# A randomized trial comparing concise and standard consent forms in the START trial

**DOI:** 10.1371/journal.pone.0172607

**Published:** 2017-04-26

**Authors:** Christine Grady, Giota Touloumi, A. Sarah Walker, Mary Smolskis, Shweta Sharma, Abdel G. Babiker, Nikos Pantazis, Jorge Tavel, Eric Florence, Adriana Sanchez, Fleur Hudson, Antonios Papadopoulos, Ezekiel Emanuel, Megan Clewett, David Munroe, Eileen Denning, the INSIGHT START Informed Consent Substudy Group

**Affiliations:** 1 Department of Bioethics, NIH Clinical Center, Bethesda, Maryland, United States of America; 2 Department of Hygiene, Epidemiology & Medical Statistics, Medical School, National & Kapodistrian University of Athens, Athens, Greece; 3 MRC Clinical Trials Unit at University College London, London, United Kingdom; 4 NIAID Division of Clinical Research, NIH, Bethesda, Maryland, United States of America; 5 Division of Biostatistics, University of Minnesota, Minneapolis, Minnesota, United States of America; 6 Genentech Research and Early Development, South San Francisco, California, United States of America; 7 Department of Clinical Sciences, Institute of Tropical Medicine, Antwerp, Belgium; 8 Washington ICC and Terry Beirn CPCRA CTU, Washington VA Medical Center, Washington DC, United States of America; 9 Forth Department of Internal Medicine, Medical School, National & Kapodistrian University of Athens, Athens, Greece; 10 Department of Medical Ethics and Health Policy, University of Pennsylvania, Philadelphia, Pennsylvania, United States of America; 11 University of New South Wales, Sydney, Australia; 12 START Community Advisory Board (CAB), University of Minnesota, Minneapolis, Minnesota, United States of America; University of Liverpool, UNITED KINGDOM

## Abstract

**Background:**

Improving the effectiveness and efficiency of research informed consent is a high priority. Some express concern about longer, more complex, written consent forms creating barriers to participant understanding. A recent meta-analysis concluded that randomized comparisons were needed.

**Methods:**

We conducted a cluster-randomized non-inferiority comparison of a standard versus concise consent form within a multinational trial studying the timing of starting antiretroviral therapy in HIV+ adults (START). Interested sites were randomized to standard or concise consent forms for all individuals signing START consent. Participants completed a survey measuring comprehension of study information and satisfaction with the consent process. Site personnel reported usual site consent practices. The primary outcome was comprehension of the purpose of randomization (pre-specified 7.5% non-inferiority margin).

**Results:**

77 sites (2429 participants) were randomly allocated to use standard consent and 77 sites (2000 participants) concise consent, for an evaluable cohort of 4229. Site and participant characteristics were similar for the two groups. The concise consent was non-inferior to the standard consent on comprehension of randomization (80.2% versus 82%, site adjusted difference: 0.75% (95% CI -3.8%, +5.2%)); and the two groups did not differ significantly on total comprehension score, satisfaction, or voluntariness (p>0.1). Certain independent factors, such as education, influenced comprehension and satisfaction but not differences between consent groups.

**Conclusions:**

An easier to read, more concise consent form neither hindered nor improved comprehension of study information nor satisfaction with the consent process among a large number of participants. This supports continued efforts to make consent forms more efficient.

**Trial registration:**

Informed consent substudy was registered as part of START study in clinicaltrials.gov #NCT00867048, and EudraCT # 2008-006439-12

## Introduction

Informed consent is a central tenet of ethical clinical research. The ideal of informed consent—a process of providing potential participants with relevant information that they understand and use to make informed and voluntary decisions—is, however, often not realized. Written informed consent documents have become increasingly long, complex, and hard to read [1–8]. Studies reveal that comprehension of study information varies widely among research participants and is often limited, especially understanding of randomization [9–10]. Systematic reviews of studies evaluating informed consent improvement strategies conclude that the most promising strategies for improving comprehension are extended discussions [11–12] and modified consent forms [12].

Prior studies that modified consent forms to evaluate improvement in comprehension had mixed results [13–18]. These studies also had three major limitations: they used small sample sizes (range 44–317), most were conducted in simulated settings, and most enrolled healthy volunteers rather than patients with illness.

To address these limitations and definitively evaluate whether simplified consent forms improve comprehension, we conducted a large, multi-national prospective randomized trial. This trial was a substudy of the Strategic Timing of AntiRetroviral Treatment (START) trial, which is investigating immediate versus deferred initiation of antiretroviral therapy in HIV-infected persons with CD4+ cell counts above 500 cells/mm^3^ [19]. Using a cluster randomized design, participating sites were randomized to use either a standard or concise consent form for all participants consenting to START. We evaluated participants’ comprehension of study information and satisfaction with the consent process.

## Methods

### Study design

Cluster randomization at the site level was used to avoid possible contamination from staff using two different consent forms for START. Details of the study design and characteristics of sites and participants have been published previously [20].

### Consent forms

The START team developed the “standard” consent template. The informed consent substudy team prepared a second “concise” consent template in collaboration with the START team. The concise version used simplified sentences and words, reduced repetition, and conveyed information using tables and bulleted lists. Both template consent documents contained all required elements of informed consent [21,22]. Both also included language informing potential START participants that they were participating in an informed consent substudy, would be asked to complete a substudy survey after signing consent, and that they could decline to complete the survey without consequence to their participation in START or their regular medical care. The “concise” template was shorter (1,821 words versus 5,927 -standard consent) and had a lower reading grade level (Flesch Kincaid Grade Level (FKGL), 9.2 versus 10.3 for the standard template) [23]. Sample consent language is shown in Box 1 and S1 File. Protocol and Consents.

**BOX 1. SAMPLE CONSENT LANGUAGE (*Full Template in S1 File. Protocol and consent)***

**CONCISE TEMPLATE RISK SECTION**

**WHAT ARE THE RISKS OF BEING IN THIS STUDY?**

All HIV medicines have some side effects.

No one knows whether people in the **DEFERRED** or **EARLY** group will have

fewer risks overall. There may also be risks that we do not know about now.

We will tell you if we learn about new risks or any other information that might be important to you.

**Possible risks of being in the DEFERRED group**:

- A drop in CD4 cell count that could increase the chance of developing HIV symptoms or AIDS.
- An increased chance of infecting others with HIV because of virus in your blood.

**Possible risks of being in the EARLY group**:

- More side effects than people who take medicines for a shorter time.
- Difficulty sticking to a schedule when taking medicines for a long time.
- Increased chance that the HIV virus will become resistant to the HIV medicines you are on.

**Other possible study risks are**:

- Side effects because of an interaction of HIV medicines with other medicines you might be taking, including herbal or alternative medicines.
- Pain, bleeding, bruising, feeling lightheaded, anxious, or rarely fainting or an infection when blood is drawn. Some people feel anxious while waiting for test results.
- Discomfort from some of the questions we ask you.

(*Word count 211, FKGL 7.0*)

**STANDARD TEMPLATE RISK SECTION**

**Possible risks of both ways of treating HIV in the study**

Each way of treating HIV disease in this study may be associated with possible benefits and risks. It is not known in the long run which of these strategies will be less risky.

Early group: The long-term risks of using HIV medicines are not clear, especially in people with higher CD4+ cell counts like those joining this study. People who take HIV medicines over many years will probably have more side effects than people who take HIV medicines for shorter periods of time. Also, you may find it hard to take HIV medicines according to your doctor or nurse’s directions for many years, which may lead to your HIV virus becoming resistant to some medicines used to treat it. Using more HIV medicines and staying on HIV medicines for a long time might also lead to HIV resistance. Because of this, you may have fewer HIV medicines you can use when the risk of disease is high.

Deferred group: The long-term risks of **not** using HIV medicines are not clear. People who do not take HIV medicines will probably have a drop in their CD4+ cell count. There may be a greater chance of developing symptoms of AIDS or other serious illnesses if the CD4+ cell count drops too much. People who do not take HIV medicines may also have an increase in the amount of HIV virus in their blood (“viral load”). People with high viral loads may be more likely to be able to pass the virus to others.

It is also possible for the HIV virus to develop resistance to any anti-HIV drug. It is not known if either way of treating HIV in this study will lead to resistance to more HIV medicines over time.

It is possible that someone could inadvertently find out that you are infected with HIV if you are taking HIV medicines and someone in your household or at work notices you taking them.

**Risks of HIV medicines**

All HIV medicines can cause side effects. Your doctor or nurse will discuss with you the risks of the specific HIV medicines that you take. These risks are not specific to this study; they are associated with taking these medicines whether you are in the study or not.

**Risks of medicine interactions (where one medicine affects how another works)**

For your safety, you must tell your doctor or nurse about all medicines, including prescription, over-the-counter (non-prescription), herbal or alternative medicines, and dietary supplements you are taking. This is because there may be serious side effects when other medicines are taken with HIV medicines. Also, please let your nurse or doctor know before you enroll in any other studies while on this study.

**Risk of transmitting HIV**

Using HIV medicines does not necessarily affect your ability to transmit HIV to other people. You should continue to use precautions to make sure you do not infect someone else. Your study doctor or nurse will tell you about how to protect yourself and other people

**Risks of blood drawing**

The risks of having blood taken include pain, bleeding, bruising, lightheadedness, fainting and rarely infection or a blood clot where the needle enters the body. You may feel some anxiety while waiting for your test results to be available. You will have blood tests like those in this study done as part of your usual care, even if you decide not to be in this study.

(*Word count 616, FKGL 9.8*)

All START sites were invited and were eligible if at least one other site offered consent in the same primary language. Ineligible sites and those that chose not to participate used the standard consent form and were not part of the substudy analysis. Both consent forms were submitted to the site’s institutional review board (IRB) or research ethics committee (REC) for approval. S2 File. List of approving IRBs/RECs. The START consent substudy team asked sites to make as few changes as possible to consent forms and to ask their IRBs/RECs to do likewise. The substudy and both consent forms were approved as part of the START protocol for each participating site. S1 File. Protocol and consents.

After approval, sites were centrally randomized 1:1 to use either the standard or concise consent form for all participants at their site. Randomization was stratified by the primary language of consent at the site in blocks of two to ensure balance between consent forms within each language. Each participant was presented with the consent form to which the site had been randomized, either standard or concise. Participants who signed consent for START between 09 April 2009 and 29 December 2013 were included in this substudy.

### Data collection

Data were collected through three surveys: 1) participant-completed survey, 2) staff-completed survey for each participant, and 3) site survey. Individuals who signed consent for START were included in the substudy whether or not they ultimately were randomized in the START trial. Basic demographic information (gender, year of birth, self-identified race, education level, CD4 count) were collected for each participant signing START consent.

The participant survey measured comprehension, satisfaction, and voluntariness using 40 multiple choice questions divided into two parts. Part A included 24 questions about participants’ experience of the consent process, satisfaction with the form, and how they made their enrollment decision. Part B included 16 questions measuring comprehension of study information, including questions about study purpose and procedures; randomization; and possible risks, side effects and benefits of participating. The survey format had been previously piloted in consent studies conducted by the NIH Clinical Center Department of Bioethics [17, 24] but comprehension questions were study-specific. Sites were instructed to administer the survey to participants right after they signed START consent before they were randomized or offered other substudies. This survey was typically self-administered, but when required, read to participants.

The staff survey for each participant was completed by the research staff member who obtained the participant’s START consent signature. The staff respondent answered three multiple choice questions, identifying his/her role, whether the consenter was the only person who discussed the study with the participant, and describing how the consent form was used in the discussion (e.g. read aloud, used as a guide or reference, or only to obtain a signature). Two additional questions asked for an estimate of time spent explaining START and the number of team members who discussed START with this participant before START consent was signed.

The consent substudy site survey included 13 multiple choice questions regarding the usual consent practices at that site, including how often consent forms were provided to participants before the clinic visit at which they actually signed START consent, the average amount of time usually spent at the site explaining START, how participant understanding was usually assessed, and how many other HIV studies were conducted at the site over the previous year. The START study site investigator completed this survey.

### Study outcome and hypotheses

The primary outcome was the proportion of participants who correctly answered the following question about randomization: “How will the researchers decide whether a participant is in the EARLY or the DEFERRED group?” Response options were: 1) “what each participant wants,” 2) “a random method like flipping a coin,” 3) “what they think is best for each participant,” 4) “based on whether the participant can pay,” or 5) “I do not know.” The key secondary endpoint was total comprehension score (range 0 to 16), equal to the number of correct answers to comprehension questions. Other secondary endpoints were composite scores of the satisfaction questions (sum, possible score 0 to 8) and the voluntariness questions (sum, possible score 0 to 4). Single-question items evaluated included: “How satisfied are you with the process of learning about the START trial?” and “Could you have refused to join the START study if you had wanted to?”

Our hypothesis was that comprehension of randomization and composite comprehension score among those in the concise group would be at least as good as that of the standard consent group (non-inferiority). A secondary hypothesis was that the concise group would report higher satisfaction with the consent process than the standard group.

### Statistical methods

The original protocol targeted 34 to 58 sites with a total 850–1450 participants. However, eligible sites were enthusiastic, resulting in 154 sites recruiting an average of 27 participants per site. In a planned START sample size re-estimation in 2013, power was recalculated using pooled data from consent groups; 82% of participants had answered the randomisation question correctly with a coefficient of variation 0.116. We re-calculated power using a higher coefficient of variation (0.15) to account for heterogeneity due to unequal cluster (site) size; randomizing 154 sites gave 84% power to determine non-inferiority based on a 7.5% absolute margin (lower bound of the two-sided 95% confidence interval (CI) for the absolute difference in the proportion of participants correctly answering the randomization question (primary outcome), concise-standard, above -7.5%).

Primary analyses included all participants who completed the substudy questionnaire after signing START consent. For primary (and other binary) outcomes randomized groups were compared using multi-level logistic models with random site effects, and group differences summarized using odds ratios and absolute differences in proportions (concise versus standard consent). The absolute difference was estimated from the corresponding multi-level logistic model coefficients back-transformed to probabilities assuming that all adjusting covariates were at the reference level (categorical) or their overall mean (continuous). Corresponding standard errors were estimated by the delta method as implemented in Stata’s nonlinear combination of coefficients command. Randomized comparisons were performed unadjusted (other than including site as a random effect: primary analysis), and adjusted for all other participant and site factors significantly associated with the outcome (secondary analysis). Comprehension (range: 0–16) and satisfaction (range: 0–8) scores were modeled using quantile (median) regression with variance corrected for clustering within sites; voluntariness score (range: 0–4) was modeled using ordinal logistic regression with variance corrected for clustering within sites. Final models for voluntariness were re-fitted using multi-level logit regression with random site effects after dichotomizing voluntariness score (4 vs <4) as there was evidence for violation of the proportionality odds assumption. Participant and site characteristics were investigated for their independent predictive value in multivariable analyses. All analyses were performed using STATA, version 13 (StataCorp, College Station, TX, USA).

## Results

Of 215 total START sites, 157 chose to participate in the substudy; 154 obtained consent for at least one person and are included in these analyses. The 77 sites randomized to standard consent were similar to the 77 sites randomized to concise consent. Table 1. Site characteristics by consent group.

**Table 1:** Site characteristics by consent group.

	Sites randomized to STANDARD consent	Sites randomized to CONCISE consent	Total sites	Total participants	p value
Number of sites	77 (100)	77 (100)	154 (100)	4229 (100)	
Site Location					p = 0.85
% North America	23 (29.9)	20 (26.0)	43 (27.9)	564 (13.3)	
% Europe (Israel)	30 (39.0)	36 (46.8)	66 (42.9)	1108 (26.2)	
% South America	11 (14.3)	11 (14.4)	22 (14.3)	1401 (33.1)	
% Oceania	7 (9.1)	4 (5.2)	11 (7.1)	107 (2.5)	
% Asia	4 (5.2)	5 (6.5)	9 (5.8)	256 (6.1)	
% Africa	2 (2.6)	1 (1.3)	3 (1.9)	793 (18.8)	
Median number of participants per site, Interquartile range (IQR), and full range	17, IQR = 9 to 36, range 1–326	14, IQR = 7 to 26, range 1–313	15, IQR = 8 to 29 range: 1–326		p = 0.25
# of other HIV studies at sites^1^					p = 0.19
≤6	31 (40.2)	45 (58.4)	76 (49.4)	1617 (38.2)	
> 6	46 (59.7)	32 (41.6)	78 (50.6)	2612 (61.8)	
# (%) sites that usually/always mail consent form ahead of visit	42 (54.5)	42 (54.5)	84 (54.5)	2161 (51.1)	p >0.99
Average amount of time explaining START					p = 0.26
< 1 hour	36 (46.8)	44 (57.1)	80 (51.9)	2278 (53.9)	
1 hour or more	41 (53.2)	33 (42.9)	74 (48.1)	1951 (46.1)	
How well average participant understands study before consent					P = 0.07
Very well	61 (79.2)	69 (89.6)	130 (84.4)	3095 (73.2)	
Slightly well	16 (20.8)	7 (9.1)	23 (14.9)	1130 (26.7)	
Not well at all	0 (0.0)	1 (1.3)	1 (0.6)	4 (0.1)	

^1^The last four variables on this table are summarized from responses to the substudy site survey that was completed once at each participating site.

**Fig 1:**
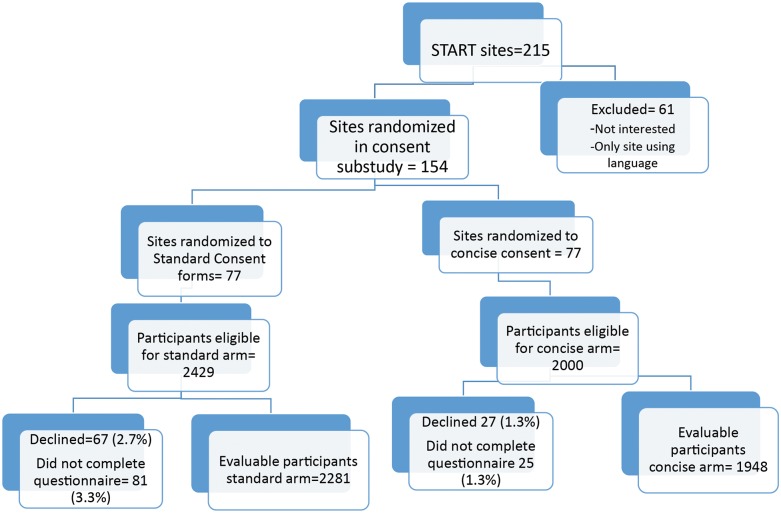
START informed consent substudy participants.

Overall, 2429 participants were at sites randomized to standard consent and 2000 at sites randomized to concise consent. Of these, 94 individuals explicitly declined to complete the survey (67 Standard, 27 Concise); an additional 106 did not complete the survey (81 Standard, 25 Concise), leaving an evaluable cohort of 4229, a 95% response rate (Fig 1. START Informed consent substudy participants). Respondents had a median age of 35 years (IQR 28–44), CD4 cell count 631 cells/mm^3^ (IQR: 559–746), and 24% were female. Participants randomized to standard consent were similar to those in the concise group (Table 2. Characteristics of Informed Consent Substudy Participants by Consent Group) and substudy participants were similar to all START participants [19].

**Table 2:** Characteristics of informed consent substudy participants by consent group.

	Participants at sites randomized to STANDARD forms	Participants at sites randomized to CONCISE forms	Total participants (%)
Evaluable Consent substudy participants	2281	1948	4229
# randomized in START^1^ [N (%)]	1904 (83.5)	1524 (78.2)	3428 (81.1)
Median (IQR) participant age, years	35.0 (28–43)	35.0 (28–44)	35.0
Female [N (%)]	562 (24.6)	448 (23.0)	1010 (23.9)
Formal education completed [N (%)]			
Less than high school	707 (31.0)	533 (27.4)	1240 (29.3)
HS graduate or equivalent	497 (21.8)	385 (19.8)	882 (20.9)
Completed vocational training	222 (9.7)	160 (8.2)	382 (9.0)
Some college or university	407 (17.8)	374 (19.2)	781 (18.5)
Completed university (bachelor’s degree)	333 (14.6)	394 (20.2)	727 (17.2)
Any post-graduate education	110 (4.8)	98 (5.0)	208 (4.9)
Median (IQR) CD4 count, cells/mm^3^	637 (564, 758)	622 (550, 732)	631
Those with previous research participation [N (%)]	236 (10.3)	264 (13.6)	500 (11.8)
Those who knew START doctors/study team for 6 months or more [N (%)]	420 (18.4)	404 (20.7)	824 (19.5)
Time known about HIV infection [N (%)]			
< 1 month	262 (11.5)	171 (8.8)	433 (10.2)
1 to 6 months	609 (26.7)	569 (29.2)	1178 (27.9)
More than 6 months but < 1 year	344 (15.1)	264 (13.6)	608 (14.4)
1 year to < 5 years	737 (32.3)	632 (32.4)	1369 (32.4)
5 or more years	315 (13.8)	304 (15.6)	619 (14.6)

All participants who completed the participant questionnaire (evaluable) were included in the substudy analysis, whether or not they were ultimately randomized to START; IQR: Interquartile Range

### Comprehension

There was no significant difference in the proportion of correct responses to the randomization question between individuals at sites randomized to standard consent and those at sites randomized to concise consent forms (82.0% (1870/2281) versus 80.2% (1563/1948) respectively; site-adjusted risk difference concise-standard +0.8% (95% CI -3.7% to 5.2%). Similarly, total comprehension score was not different between the two consent groups, with a median of 13 out of 16 for each group, (site adjusted difference 0.0 (95% CI -1.7 to +1.7, p≥0.99)). Table 3. Primary and Secondary Outcomes by Consent group.

**Table 3:** Primary and secondary outcomes by consent group.

	Total	Participants at sites randomized to STANDARD forms	Participants at sites randomized to CONCISE forms	Estimated (95% CI) difference (CONCISE vs. STANDARD)	p-value
# (%) correctly answered randomization question	3433 (81.2)	1870 (82.0)	1563 (80.2)	0.75% (-3.72%, 5.21%)^\\^	0.743
Median (IQR) Comprehension Score	13.0 (10.0,15.0)	13.0 (10.0, 15.0)	13.0 (10.0, 15.0)	0.0 (-1.7, 1.7)^2^	>0.999
Median (IQR) Satisfaction score	7.0 (6.0, 8.0)	7.0 (6.0, 8.0)	7.0 (6.0, 8.0)	0.0 (-0.3, 0.3)^2^	>0.999
Median (IQR) Voluntariness score	4.0 (3.0, 4.0)	4.0 (3.0, 4.0)	4.0 (3.0, 4.0)	-3.41% (-7.71%, 0.90%)^3^	0.121
# (%) Very or somewhat satisfied with the process of learning about START	4183 (98.9)	2256 (98.9)	1927 (98.9)	0.12% (-0.49%, 0.73%)^1^	0.698
# (%) who said they could have refused to join START	3634 (85.9)	2014 (88.3)	1620 (83.2)	0.54% (-1.75%, 2.82%)^1^	0.645

1: Difference in percentage;

2: Difference in median;

3: Difference in the percentage reporting the maximum score.

All estimates and p-values from univariable models with adjustments for the clustering of START participants within sites

Results from the corresponding multivariable model are presented in Table 4. Factors predicting correct response to randomization question. Despite no difference between consent groups, individuals from sites that had conducted more previous HIV studies and sites that always mailed the consent form ahead of the clinic visit answered the randomization question correctly more often. Furthermore, on adjusted analysis correct answers to the randomization question were independently more likely from individuals who were younger, white, better educated, from Australia or Africa, took the self-administered questionnaire rather than having it read to them, and were ultimately randomized in START. Despite the independent influence of education, race, site country, and the number of HIV studies at the site, there were no statistically significant interactions between these variables and consent assignment.

**Table 4:** Factors predicting correct response to randomization question.

	Total N	Number (%) with correct answer	Adjusted Risk (odds ratio)	Confidence intervals	P Value	Risk Difference	95% C.I.
**Consent Group**							
Standard	2281	1870 (82%)	1			0	
Concise	1948	1563 (80.2%)	1.16	0.87, 1.55	0.305	1.8	-1.7, 5.2
**Randomized to START**							
No	801 (18.9%)	603 (75.3)	0.76	0.62, 0.94	0.01	-3.7	(-6.4, -0.4)
Yes	3428 (81.1%)	2830 (82.6)	1				
**Site Country**							
North America	564 (13.3)	449 (79.6)	1.47	0.97, 2.23	0.07	4.1	(-0.4, 8.6)
Europe/Israel*	1108 (26.2)	919 (82.9)	1			0	
South America/Mexico	1401 (33.1)	1140 (81.4)	1.48	0.99, 2.21	0.06	4.2	(-0.2, 8.6)
Oceania	107 (2.5)	102 (95.3)	2.8	1.0, 7.85	0.05	8.7	(1.6, 15.9)
Asia	256 (6.1)	206 (80.5)	2.09	0.76, 5.77	0.153	7	(-0.6, 14.6)
Africa	793 (18.8)	617 (77.8)	3.52	1.61, 7.67	0.002	9.9	(3.9, 15.7)
**Race/ethnicity**							
Black	1273 (30.1)	959 (75.3)	0.38	0.28, 0.51	<0.0001	-16.5	(-24.1, -8.9)
Hispanic	721 (17.0)	567 (78.6)	0.44	0.32, 0.60	<0.0001	-13.4	(-20.7, -6.2)
Asian	285 (6.7)	226 (79.3)	0.3	0.12, 0.72	<0.0007	-21.6	(-41.9, -1.4)
White*	1775 (42.0)	1534 (86.4)	1			0	
Other	175 (4.1)	147 (84.0)	0.66	0.40, 1.09	0.104	-5.9	(-13.9, 2.2)
**Educational Level At Baseline**							
Less than high school/Less than a year*	1240 (29.3)	910 (73.4)	1			0	
High school graduate or equivalent	882 (20.9)	688 (78.0)	1.4	1.09, 1.79	0.009	3.7	(0.6, 6.7)
Completed vocational training	382 (9.0)	306 (80.1)	1.49	1.05, 2.11	0.026	4.2	(0.3, 8.2)
Some college/some university	781 (18.5)	677 (86.7)	2.92	2.18, 3.91	<0.0001	9	(4.9, 13.1)
Bachelor's degree / University degree	727 (17.2)	654 (90.0)	3.55	2.58, 4.88	<0.0001	9.9	(5.5, 14.3)
Any post-graduate education	208 (4.9)	194 (93.3)	5.56	3.06, 10.13	<0.0001	11.5	(6.4, 16.6)
**Other HIV Studies At Site (Current/Past)**							
0	45 (1.1)	31 (68.9)	0.35	0.13, 0.98	0.047	-17.9	(-40.1, 4.3)
1–3	825 (19.5)	598 (72.5)	0.58	0.38, 0.88	0.01	-8.1	(-14.8, -1.4)
4–6	747 (17.7)	594 (79.5)	0.66	0.43, 1.0	0.053	-5.9	(-12.3, 0.5)
7–10	1045 (24.7)	882 (84.4)	0.9	0.61, 1.33	0.597	-1.4	(-6.4, 3.7)
>10*	1567 (37.1)	1328 (84.7)	1			0	
**Data Collection Method**							
Participant self-administered*	3646 (86.2)	2973 (81.5)	1			0	
Staff read to participant	583 (13.8)	460 (78.9)	1.52	1.14, 2.02	0.004	4.4	(1.3, 7.6)
**Age At Consent**							
per 10 years older			0.87	0.80, 0.95	0.002	-1.8	(-3.0, -0.5)
**Number of staff who explain the study at the site**							
1 to 2	3535 (83.6)	2882 (81.5)	1			0	
3 to 4	624 (14.8)	512 (82.1)	0.91	0.63, 1.31	0.608	-1.2	(-5.9, 3.5)
5+	70 (1.7)	39 (55.7)	0.31	0.12, 0.84	0.021	-20.6	(-43.6, 2.4)
**Consent mailed before signing**							
Always*	1710 (40.4)	1470 (86.0)	1			0	
Usually/Sometimes/Rarely/Never	2519 (63.1)	1963 (77.9)	0.67	0.49, 0.90	0.009	-5.8	(-10.3, -1.2)
**Site participated in START pilot phase**							
Pilot phase site	2570 (60.8)	2154 (83.8)	1			0	
Not pilot phase site	1659 (39.2)	1279 (77.1)	0.73	0.55, 0.99	0.041	-4.2	(-8.7, 0.2)

On adjusted analysis there remained no evidence of difference between groups on comprehension scores, yet scores were independently significantly higher (p<0.05) in individuals who were white and better educated, and at sites with more previous HIV studies, where site leaders explained the study and staff thought participants understood the study very well. Participants’ comprehension scores were lower at sites which rarely or never mailed the consent form ahead of the clinic visit. A statistically significant (Global test p = 0.007) interaction effect was found, indicating that, among Asians, individuals in the concise group had an average 1.2 (95% CI: 0.2–2.2) units lower comprehension score. In all other races, differences between standard and concise groups were not statistically significant.

### Satisfaction and voluntariness

There was no statistically significant difference between the standard and concise cohorts on the composite satisfaction score (median 7 for each group, p >0.99, maximum possible 8). The secondary hypothesis, that the concise group would have higher satisfaction scores was therefore not upheld. However, significantly fewer participants in the concise group found the form too long or too detailed (too long- concise 10.7%, standard 19.2%); too detailed- concise 10.9%, standard 18.3%) (Both p<0.001).

On multivariable analysis, greater satisfaction was independently associated (p<0.05) with race (other than Asian or Black), education, region (not Africa or Australia), consent form in English, and by whether both written participant information and consent forms were provided. No statistically significant interactions were seen between groups on satisfaction scores and education, race, site country, or language. There was no significant difference between consent groups in the proportion of individuals who were very or somewhat satisfied with the process of learning about START (site-adjusted risk differences concise-standard 0.12%; 95%CI: -0.49%, 0.73%, Table 3. Primary and Secondary Outcomes by Consent group). Results from multivariable analysis were similar.

There was also no evidence of difference between standard and concise groups on composite measures of voluntariness (median 4 in each group; adjusted difference in the percentage reporting the maximum score -3.4% (-7.7, +0.9) p = 0.12)). Voluntariness scores were higher, on multivariable analysis, in older individuals, those who knew the START team for >6 months, at sites that had enrolled <10 patients, where the principal investigator explained START and where the survey was read to the participant (all p<0.05). Voluntariness scores were substantially and significantly lower in Asia (p<0.001). When the question “Could you have refused to join START” was analyzed separately, there was no evidence of difference between randomized groups either in univariable or in multivariable analyses (site-adjusted risk differences concise-standard 0.54%; 95%CI: -1.75%, 2.82%; Table 3. Primary and Secondary Outcomes by Consent group).

## Discussion

With over 4000 participants at 154 sites in 21 countries around the world, this is the largest randomized study to date evaluating standard versus concise consent forms on research participants’ comprehension and satisfaction with informed consent. These data convincingly show that shortening research consent forms and lowering reading levels neither impairs nor improves informed consent comprehension or satisfaction. Three major points are worth emphasizing.

First, despite widespread opinions that simpler, shorter consent forms might enhance [24] or compromise comprehension [25], neither understanding of randomization nor overall comprehension of study information was different between standard and concise consent form groups in this cohort. Among previous studies with smaller cohorts that modified consent forms, most were not able to show significant improvement in understanding, [15, 17, 26–28] with some notable exceptions [29–30]. Concurrently, absent evidence, it was difficult to respond to concerns that shortening consent forms would pose risks to participants by lowering comprehension [25]. Our multinational study with a large cohort demonstrated no improvement or decrement in comprehension of study information between those given a standard lengthy consent form and those given a concise form restructured for ease of reading. Satisfaction with consent also did not differ between groups, although satisfaction was high overall.

Second, many factors independently affect comprehension and satisfaction with consent. Comprehension was higher at sites that sent consent forms to prospective participants ahead of their consent visit. Participants who received educational information in addition to the consent form were more satisfied with the process. These findings suggest simple strategies that research teams could adopt to improve participant comprehension and satisfaction. Further research to evaluate the extent to which consistently sending materials ahead of time improves comprehension and providing educational information affects satisfaction or comprehension would be valuable.

Comprehension of randomization and overall comprehension was also independently influenced by age and education, as has been previously shown [17, 28, 31–32]. Notably, despite better comprehension of randomization than in most published studies,[10] one in five participants enrolled in START without understanding that their treatment assignment was going to be determined through a random process. Overall comprehension was better at sites with previous HIV research experience, suggesting possible benefits of staff experience or training. Participants seemed satisfied with the consent process regardless of the length and complexity of the forms. Investigators and review bodies should be reassured that changing the consent form did not negatively alter participants’ reported satisfaction or voluntariness, and significantly fewer concise consent group participants rated the forms as too long or too complex.

Third, making consent forms more concise and readable may have other important advantages. Evidence shows that participants don’t always read consent forms, are less likely to read long forms, [33–35] and that reading them can take a long time [36]. Further, consent forms often contain more information than the brain can normally absorb [37]. Changes to US regulations governing consent for research (Title 45 CFR 46, the Common Rule) include stricter requirements regarding information to be included in research consent forms and the manner in which information should be presented, with an explicit goal of making consent forms more meaningful, and not “… unduly long…” [38]. FDA guidance also advises IRBs to review consent form content, length, and presentation and avoid forms that are long and complex with high reading levels [39].

Consent forms serve several important functions besides informing prospective study participants. Readable consent forms can serve as an enduring record of study plans for investigators and institutions and for participants who can use the form to seek advice from others or to refer to over time [40]. Shorter forms could reduce the time that review committees spend on consent forms, and enable investigators to spend more time discussing and emphasizing important points, although both of these should be tested empirically.

Our study had several limitations. First, participant comprehension and satisfaction with consent is affected by discussions with staff, other participants, and other sources of information; however, randomizing sites enrolling large numbers of participants was meant to isolate the effects of the consent form. Second, shortening and simplifying the consent form was difficult due to sponsor and review committee concerns about regulatory compliance and the possible risks of providing less information, a challenge also experienced by others [41]. Lowering the reading level was particularly challenging, resulting in a small difference in reading level between templates, and higher grade levels for both templates than recommended for consent forms [5]. Questions used for evaluating comprehension and satisfaction have been previously utilized, but have not been psychometrically validated. Differences in responses from the small subset of participants to whom staff read the questionnaires (versus self-administration) may reflect a desire to please the staff.

## Conclusions

In this, the largest randomized study to date of informed consent forms, participant comprehension of study information and satisfaction with the process did not vary with the length and complexity of the consent forms. A concise consent form may have important practical and other advantages, and the results of this study indicate that the use of one does not compromise understanding, satisfaction, or voluntariness in the informed consent process.

## Supporting information

S1 File Protocol and consents. START Original protocol (START Version 1.0, including Informed Consent Substudy protocol), final protocol (START Version 2.0), summary of changes. And original statistical analysis plan (dated 26 Oct 2013). There were no subsequent changes made to this document.
(PDF)

S2 File List of approving IRBs/RECs. List of approving IRBs/RECs with institution and FWA numbers.
(DOCX)

S3 File CONSORT Checklist CONSORT 2010 checklist of information to include when reporting a randomised trial. (DOC)

S4 File CONSORT Flow Chart. Participant Flow.
(DOC)

S5 File Previous publication. Denning et al. Reported consent processes and demographics: a substudy of the INSIGHT Strategic Timing of AntiRetroviral Treatment (START) trial. *HIV Medicine* 2015.
(PDF)

S6 File Full list of START investigators. (DOCX)

## References

[1] Baker MT, Taub HA. Readability of informed consent forms for research in a Veterans Administration medical center. *JAMA*. 1983; 250(19):2646–8. 6632164

[2] LoVerde ME, Prochazka AV, Byyny RL. Research consent forms: continued unreadability and increasing length. *J Gen Intern Med*. 1989; 4(5):410–2. 2795264 10.1007/BF02599693

[3] Tarnowski KJ, Allen DM, Mayhall C, Kelly PA. Readability of pediatric biomedical research informed consent forms. *Pediatrics*. 1990;85(1):58–62 2296494

[4] Grossman SA, Piantadosi S, Covahey C. Are informed consent forms that describe clinical oncology research protocols readable by most patients and their families? *J Clin Oncol*. 1994; 12(10):2211–5 10.1200/JCO.1994.12.10.2211 7931491

[5] Paasche-Orlow MK, Taylor HA, Brancati FL. Readability standards for informed-consent forms as compared with actual readability. *N Engl J Med*. 2003 20;348(8):721–6 10.1056/NEJMsa021212 12594317

[6] Sharp SM. Consent documents for oncology trials: does anybody read these things? *Am J Clin Onco*l. 2004;27(6):570–5 15577434 10.1097/01.coc.0000135925.83221.b3

[7] Beardsley E, Jefford M, Mileshkin L. Longer consent forms for clinical trials compromise patient understanding: so why are they lengthening? *J Clin Oncol*. 2007; 25(9):e13–4 10.1200/JCO.2006.10.3341 17369564

[8] Albala I, Doyle M, Appelbaum PS. The evolution of consent forms for research: a quarter century of changes. IRB: *Ethics & Human Research*. 2010;32(3):7–11 20590051

[9] Mandava A, Pace C, Campbell B, Emanuel E, Grady C. The quality of informed consent: mapping the landscape. A review of empirical data from developing and developed countries. *J Med Ethics*. 2012; 38(6):356–65 10.1136/medethics-2011-100178 22313664 PMC4825806

[10] Tam NT, Huy NT, Thoa le TB, Long NP, Trang NT, Hirayama K, Karbwang J. Participants' understanding of informed consent in clinical trials over three decades: systematic review and meta-analysis. *Bull World Health Organ*. 2015 1;93(3):186–98 10.2471/BLT.14.141390 25883410 PMC4371493

[11] Flory J, Emanuel E. Interventions to improve research participants' understanding in informed consent for research: a systematic review. *JAMA*. 2004 10 6;292(13):1593–601. 10.1001/jama.292.13.1593 15467062

[12] Nishimura A, Carey J, Erwin PJ, Tilburt JC, Murad MH, McCormick JB. Improving understanding in the research informed consent process: a systematic review of 54 interventions tested in randomized control trials. *BMC Med Ethics*. 2013 7 23;14–28. 10.1186/1472-6939-14-28 PMC3733934 23879694

[13] Paris A, Brandt C, Cornu C, et al: Informed consent document improvement does not increase patients’ comprehension in biomedical research. *Br J Clin Pharmacol* 2010, 69(3):231–237. 10.1111/j.1365-2125.2009.03565.x 20233193 PMC2829692

[14] Guarino P Elbourne D, Carpenter J, Peduzzi P, Consumer involvement in consent document development: a multicenter cluster randomized trial to assess study participants' understanding. *Clinical Trials* 2006; 3:19–30 10.1191/1740774506cn133oa 16539087

[15] Coyne CA, Xu R, Raich P, Plomer K, Dignan M, Wenzel LB, Fairclough D, Habermann T, Schnell L, Quella S, Cella D; Eastern Cooperative Oncology Group. Randomized, controlled trial of an easy-to-read informed consent statement for clinical trial participation: a study of the Eastern Cooperative Oncology Group. *J Clin Oncol*. 2003 1;21(5):836–42 12610182 10.1200/JCO.2003.07.022

[16] Murphy DA, O’Keefe ZH, Kaufman AH: Improving comprehension and recall of information for an HIV vaccine trial among women at risk for HIV: Reading level simplification and inclusion of pictures to illustrate key concepts. *AIDS Educ Prev* 1999, 11(5):389–399. 10555623

[17] Stunkel L, Benson M, McLellan L, Sinaii N, Bedarida G, Emanuel E, Grady C: Comprehension and informed consent: assessing the effect of a short consent form. *IRB*: *Ethics and Human Research* 2010, 32(4):1–9 20853797 PMC4819424

[18] Enama ME, Hu Z, Gordon I, Costner P, Ledgerwood JE, Grady C; the VRC 306 and 307 Consent Study Teams. Randomization to standard and concise informed consent forms: Development of evidence-based consent practices. *Contemp Clin Trials*. 2012, 33(5): 895–902 22542645 10.1016/j.cct.2012.04.005 PMC3408575

[19] INSIGHT START Study Group, Lundgren JD, Babiker AG, Gordin F, Emery S, Grund B, Sharma S, Avihingsanon A, Cooper DA, Fätkenheuer G, Llibre JM, Molina JM, Munderi P, Schechter M, Wood R, Klingman KL, Collins S, Lane HC, Phillips AN, Neaton JD. Initiation of Antiretroviral Therapy in Early Asymptomatic HIV Infection. *N Engl J Med*. 2015 8 27;373(9):795–807. 10.1056/NEJMoa1506816 26192873 PMC4569751

[20] Denning E, Sharma S, Smolskis M, Touloumi G, Walker S, Babiker A, Clewett M, Emanuel E, Florence E, Papadopoulos A, Sánchez A, Tavel J, and Grady C, for the International Network for Strategic Initiatives in Global HIV Trials (INSIGHT) START Study Group. Reported consent processes and demographics: a substudy of the INSIGHT Strategic Timing of AntiRetroviral Treatment (START) trial *HIV Medicine* (2015), 16 (Suppl. 1), 24–29 10.1111/hiv.12230 PMC4341940 25711320

[21] Department of Health and Human Services and Office for Human Research Protections. Code of Federal Regulations: Title 45, public welfare; Part 46, protection of human subjects (http://www.hhs.gov/ohrp/humansubjects/guidance/45cfr46.html). 45CFR. 46.116 11686173

[22] International Conference on Harmonization Good Clinical Practice Guideline E6, Section 4.8.10. http://www.fda.gov/downloads/Drugs/…/Guidances/ucm073122.pdf 10.1080/105294199277860 10386329

[23] Kincaid JP, Fishburne RP, Rogers RL, Chissom BS. Derivation of new readability formulas (Automated Readability Index, Fog Count, Flesch Reading Ease) for Navy enlisted personnel Research Branch Report 8–75. Memphis Naval Air Station, 1975

[24] Lorell BH, Mikita JS, Anderson A, Hallinan Z, Forrest A. Informed consent in clinical research: Consensus recommendations for reform identified by an expert interview panel. *Clin Trials* 2015 12;12(6):692–5 10.1177/1740774515594362 26178662 PMC4657389

[25] Emanuel EJ, Grady C. Is longer always better? *Hastings Center Rep* 2008; 38: 10–11 18584850

[26] Davis TC, Holcombe RF, Berkel HJ, Pramanik S, Divers SG: Informed consent for clinical trials: a comparative study of standard versus simplified forms. *J Natl Cancer Inst* 1998, 90(9):668–674. 9586663 10.1093/jnci/90.9.668

[27] Taub HA, Baker MT, Kline GE, Sturr JF: Comprehension of informed consent information by young-old through old-old volunteers. *Exp Aging Res* 1987, 13(4):173–178 10.1080/03610738708259321 3505870

[28] Matsui K, Lie R, Turin T, Kita Y. A Randomized Controlled Trial of Short and Standard-Length Consent Forms for a Genetic Cohort Study: Is Longer Better. *J Epidemiol* 2012;22(4):308–316 10.2188/jea.JE20110104 22447213 PMC3798649

[29] Dresden GM, Levitt MA. Modifying a standard industry clinical trials consent form improves patient information retention as part of the informed consent process. *Acad*. *Emerg Med* 2001; 8:246–252. 11229946 10.1111/j.1553-2712.2001.tb01300.x

[30] Beardsley E, Jefford M, Mileshkin L. Longer consent forms for clinical trials compromise patient understanding: so why are they lengthening? *J Clin Oncol*. 2007 3 20; 25(9):e13–4 10.1200/JCO.2006.10.3341 17369564

[31] Joffe S, Cook EF, Cleary PD et al Quality of informed consent: a new measure of understanding among research subjects. *J Natl Cancer Inst* 2001; 93:139–147. 11208884 10.1093/jnci/93.2.139

[32] Taub HA, Baker MT, Sturr JF. Informed consent for research. Effects of readability, patient age, and education. *J Am Geriatr Soc*. 1986 8; 34(8):601–6. 3722678 10.1111/j.1532-5415.1986.tb05766.x

[33] Ittenbach R, Senft E, Huang G, Corsmo J, Sieber J. Readability and understanding of informed consent among participants with low incomes: a preliminary report. *J Emp Res Human Res Ethics*. 2015, 10(5): 444–448. 10.1177/1556264615615006 26564942

[34] Sharp SM. Consent documents for oncology trials: does anybody read these things? Am J Clin Oncol 2004; 27: 570–575 15577434 10.1097/01.coc.0000135925.83221.b3

[35] Plaut VC, Bartlett RP. Blind Consent? A Social Psychological Investigation of Non-Readership of Click-Through Agreements. *Law and Human Behavior* pp 1–23 First online: 16 6 2011 22849415 10.1037/h0093969

[36] Hochhauser M. How long does it take to read a consent form? *SOCRA Source*: *A publication of the Society of Clinical Research Associates*. 2008; 58: 62–65

[37] Hochhauser M. Memory overload: the impossibility of informed consent. Appl Clin Trials 11 1 2005 Available at http://www.appliedclinicaltrialsonline.com/memory-overload-impossibility-informed-consent.

[38] Final revisions to the Common Rule. Federal policy for the protection of human subjects. https://www.hhs.gov/ohrp/regulations-and-policy/regulations/finalized-revisions-common-rule/index.html#. Accessed February 7, 2017.

[39] FDA Informed consent information sheet, guidance for IRBs, investigators, and sponsors. 2014; http://www.fda.gov/downloads/RegulatoryInformation/Guidances/UCM405006.pdf. Accessed Jan 15, 2016

[40] Resnik D. Do informed consent documents matter? *Contemporary Clinical Trials*. 2009; 30: 114–115 10.1016/j.cct.2008.10.004 18977313 PMC2670580

[41] Kass N, Taylor H, Ali J, Hallez K, Chaisson L. A pilot study of simple interventions to improve informed consent in clinical research: Feasibility, approach, and results. *Clinical Trials*. 2015; 12(1): 54–66. 10.1177/1740774514560831 25475879 PMC4344898

