# Supplementary material for: A randomized trial comparing concise and standard consent forms in the START trial
Source: PLoS One. 2017 Apr 26;12(4):e0172607. doi: 10.1371/journal.pone.0172607 (PMC5406127; doi:10.1371/journal.pone.0172607)
Supplement: S1 File — START Original protocol (START Version 1.0, including Informed Consent Substudy protocol), final protocol (START Version 2.0), summary of changes. And original statistical analysis plan (dated 26 Oct 2013). There were no subsequent changes made to this document. (PDF) [file pone.0172607.s001.pdf]

Re: 16-02237: A Randomized Trial Comparing Concise and Standard Consent Forms in the START Trial

This supplement contains the following items:

1. Original protocol (START Version 1.0, including Informed Consent Substudy protocol), final protocol (START Version 2.0), summary of changes.
2. Original statistical analysis plan (dated 26 Oct 2013). There were no subsequent changes made to this document.

Strategic Timing of AntiRetroviral Treatment (START)

Sponsored by:
The University of Minnesota
Minneapolis, Minnesota, USA

In collaboration with four International Coordinating Centers (ICCs) of the INSIGHT Network:

Copenhagen HIV Programme (CHIP) -- Copenhagen, Denmark
Medical Research Council (MRC) Clinical Trials Unit -- London, United Kingdom
National Centre in HIV Epidemiology and Clinical Research (NCHECR), University of New South Wales -- Sydney, Australia
The Institute for Clinical Research at the Veterans Affairs Medical Center -- Washington, D.C., USA

Funded by:
Division of AIDS, The National Institute of Allergy and Infectious Diseases (NIAID), National Institutes of Health (NIH)
Agence Nationale de Recherches sur le SIDA et les Hépatites Virales (ANRS, France)
Bundesministerium für Bildung und Forschung (BMBF, Germany)
NEAT - European AIDS Treatment Network
Australian National Health and Medical Research Council (NHMRC)
Department of Bioethics, The Clinical Center, NIH
Division of Clinical Research, NIAID, NIH
National Cancer Institute (NCI), NIH
National Heart, Lung, and Blood Institute (NHLBI), NIH
National Institute of Mental Health (NIMH), NIH
National Institute of Neurological Disorders and Stroke (NINDS), NIH

Pharmaceutical support provided by:
Abbott Laboratories, Inc.
Bristol-Myers Squibb
Gilead Sciences, Inc.
GlaxoSmithKline, Inc.
Merck & Co., Inc.
Tibotec Pharmaceuticals, Ltd.

Protocol Co-Chairs: Abdel Babiker, Sean Emery, Fred Gordin, Jens Lundgren

The START protocol is being managed and conducted by the International Network for Strategic Initiatives in Global HIV Trials (INSIGHT) with primary support by the U.S. National Institutes of Health through grants from the Division of AIDS, NIAID, and other NIH institutes to the University of Minnesota.

The University of Minnesota will serve as the sponsor for the study and will subcontract with four ICCs that will be responsible for implementation of Good Clinical Practice (GCP) and for oversight of the conduct of the trial at clinical research sites. The University of Minnesota is a constitutional entity under the laws of the State of Minnesota and assumes liability only to the extent provided under the Minnesota Tort Claims Act, Minnesota Statutes, Section 3.736.

The legal representative for the START trial in Europe is the Copenhagen HIV Programme (CHIP).

TABLE OF CONTENTS

1 SYNOPSIS 1

2 BACKGROUND AND RATIONALE 7

2.1 Primary Study Hypothesis 7

2.2 Background on Which Study Hypothesis Was Generated 7
2.2.1 Treatment Guidelines 7
2.2.2 Summary of Potential Risks and Benefits 9

2.3 Rationale for Selected Study Design 10
2.3.1 CD4+ Cell Count Entry Criteria and CD4+ Count Deferral Criteria for Control Group 10
2.3.2 Broad Entry Criteria 10
2.3.3 Composite Endpoint of AIDS* and Non-AIDS 11
2.3.4 Major Secondary Outcomes 11

2.4 Summary 12

3 METHODOLOGY 14

3.1 Study Design 14

3.2 Study Objectives 15
3.2.1 Primary Objective 15
3.2.2 Secondary Objectives 15
3.2.3 Other Objectives 16

3.3 Primary Study Endpoint 17

3.4 Randomization 17

3.5 Sample Size and Statistical Considerations 17

3.6 Participant Selection 19
3.6.1 Inclusion Criteria 19
3.6.2 Exclusion Criteria 20

3.7 Study Plan 20
3.7.1 Treatment Strategies 20
3.7.2 Concomitant Medications 21
3.7.3 Baseline Screening 22
3.7.4 Participant Follow-up 23
3.7.5 Participant Relocation to a New Site 25
3.7.6 Stored Samples and Future Research 25

3.8 Event Reporting 25
3.8.1 AIDS and Non-AIDS Events 26
3.8.2 Deaths 26
3.8.3 Pregnancy Outcomes 26
3.8.4 Reporting Serious Events 26
3.8.5 Serious Adverse Event Reporting Required by the European Union... 27

4 CLINICAL MANAGEMENT ISSUES 28

4.1 Management of ART 28
4.1.1 Use of Potent Combination ART 28
4.1.2 Factors Affecting Choice of an ART Regimen 28
4.1.3 Antiretroviral Drug Product Information 28
4.1.4 Approved Sources for Antiretroviral Drugs 28
4.1.5 Intolerance of an Antiretroviral Drug 29
4.1.6 Drug Toxicity and Grading 29
4.1.7 Use of Antiretroviral Drugs in Women of Child-bearing Potential 29
4.1.8 Abacavir Hypersensitivity Reaction 29
4.1.9 Resistance Testing 30
4.1.10 Management of Virological Failure 30
4.1.11 Discontinuation of ART 30
4.1.12 Documentation of Reasons for Initiation or Change in ART 31
4.1.13 Use of Drugs for Prophylaxis for Opportunistic Diseases 31
4.1.14 Provision of Antiretroviral Drugs at Study Completion 31

4.2 CD4+ Cell Count and Viral Load Monitoring 31

4.3 Pregnancy and Breastfeeding 32

4.4 HIV Transmission Counseling 32

4.5 Study Withdrawal 32

4.6 Co-enrollment in Other Studies 33

5 EVALUATION 34

5.1 Data Analysis 34

5.2 Data Monitoring 35
5.2.1 Monitoring of Pilot Phase of the Study 35
5.2.2 Monitoring of Second Phase of the Study (Definitive Study) 36

6 PROTECTION OF HUMAN SUBJECTS & OTHER ETHICAL CONSIDERATIONS 38

6.1 Local Review of Protocol and Informed Consent 38

6.2 Ethical Conduct of the Study 38

6.3 Informed Consent of Study Participants 38

6.4 Confidentiality of Study Participants 38

7 OTHER IMPORTANT DOCUMENTS AND POLICIES 39

7.1 Reference Documents 39

7.2 Data Collection and Monitoring 39

7.3 Publications and Presentations 39

8 SUBSTUDIES 40

8.1 Genomics Substudy 41
8.1.1 Background and Rationale 41
8.1.2 Purpose 41
8.1.3 Study Design 41
8.1.4 Sample Size and Statistical Considerations 42
8.1.5 Participant Selection 42
8.1.6 Enrollment and Data Collection 42
8.1.7 Follow-up 42
8.1.8 Specimen Collection 42
8.1.9 Human Subjects 43
8.1.10 Confidentiality 43
8.1.11 Access to Stored Specimens Collected for the START Genomics Substudy 44
8.1.12 Access to Individual Test Results 44

8.2 Neurology Substudy 46
8.2.1 Background and Rationale 46
8.2.2 Methodology 48
8.2.3 Clinical Management Issues 55
8.2.4 Evaluation 57
8.2.5 Human Subjects 59

8.3 Informed Consent Substudy 60
8.3.1 Background and Rationale 60
8.3.2 Purpose 60
8.3.3 Study Hypothesis 61
8.3.4 Study Design 61
8.3.5 Eligibility Criteria for Sites 61
8.3.6 Randomization of Participating Sites 62
8.3.7 Data Collection 62
8.3.8 Differences between Standard Consent and Concise Consent for START 63

8.3.9 Study Outcome Measures ... 63
8.3.10 Sample Size Estimation ... 64
8.3.11 Data Analysis ... 65
8.3.12 Administration of the Questionnaire ... 65
8.3.13 Confidentiality of the Questionnaire Responses ... 65
8.3.14 Study Alternative for Sites and START Participants ... 66
8.3.15 Human Subjects ... 66
8.3.16 Benefits ... 66
8.3.17 Risks ... 66

APPENDICES

A. Sample Consent Forms
  A1. START Main Study Sample Consent
  A2. START Study Specimen Storage (Plasma & Urine) Sample Consent
  A3. START Study Specimen Storage (Biopsy Tissue & Blood) at Diagnosis of New Malignancy Sample Consent
  A4. START Genomics Substudy Sample Consent
  A5. START Genomics Substudy Sample Withdrawal Letter
  A6. START Neurology Substudy Sample Consent
  A7. START Informed Consent Substudy: Sample Concise Consent for Main Study
B. Summary of Background Data for START Design
C. Time and Events Schedule
D. INSIGHT Executive Committee and START Protocol Team
E. Primary Endpoint Definition
F. Guidelines for Resistance Testing
G. Sample Size Justification
H. References on INSIGHT Website
I. List of Acronyms

REFERENCES

1 Synopsis

Purpose

The purpose of this randomized study is to determine whether immediate initiation of antiretroviral treatment (ART) is superior to deferral of ART until the CD4+ declines below 350 cells/mm^3 in terms of morbidity and mortality in HIV-1 (subsequently referred to as HIV) infected persons who are antiretroviral naïve with a CD4+ count above 500 cells/mm^3.

The study will proceed in two phases: (1) a pilot phase, involving at least 900 participants; and (2) a definitive phase, expanding enrollment to an estimated 4,000 participants. Upon completion of the pilot phase, a recommendation will be made to the primary funder (DAIDS, NIAID, NIH) concerning whether the study should be expanded and prolonged into a definitive study. Successful completion of the pilot phase requires enrollment of at least 900 participants in 1 year by 70 designated sites supported by DAIDS. Additional sites, funded by organizations other than DAIDS, will also participate in the pilot and definitive phase.

Rationale

Section 2 and Appendix B provide a detailed summary of the background and rationale for START. With the exception of data from a small subgroup of participants who were not taking ART at the time of entry into the Strategies for Management of AntiRetroviral Therapy (SMART) study, evidence from randomized trials does not exist to guide decisions about the initiation of highly active ART regimens for HIV-infected individuals with CD4+ cell counts > 200 cells/mm^3. Current guidelines are based largely on data from cohort studies and recommend initiating ART between 201 and 350 cells/mm^3. ART is usually not initiated until the CD4+ cell count is < 350 cells/mm^3 for several reasons:

- Small absolute (as opposed to relative) risk differences in the rates of AIDS associated with earlier ART use and therefore uncertainty about the risk-benefit ratio;
- Uncertainty about the cost-effectiveness of early ART even if the risk-benefit ratio is favorable;
- Concerns about serious complications associated with ART, e.g., cardiovascular, renal, and liver disease, that might negate the expected benefits of ART;
- Side effects of ART that impact quality of life;
- Concern that there could be waning adherence with long-term use of ART and consequent development of HIV resistance; and
- The potential availability of even better treatments in the future that would be easier to take and would more durably suppress the virus and carry a lower risk of the development of resistance.

Recent data from epidemiological studies, however, indicate that the risk of AIDS is graded and persists at CD4+ levels > 500 cells/mm^3. Furthermore, for a given CD4+ count, the risk of AIDS appears to be lower in patients who have started ART than in

those who are ART-naïve. In addition, rates of serious non-AIDS diseases – cardiovascular, renal and hepatic disease and non-AIDS malignancies – are lower at higher CD4+ counts. Data from the SMART study, a treatment interruption trial that enrolled 5,472 participants with a CD4+ count > 350 cells/mm^3, are consistent with these epidemiological data. In SMART, there was a higher rate of both AIDS and non-AIDS events in the episodic compared to the continuous ART arm. This adverse treatment effect was evident among subgroups of participants on ART at entry, participants who previously took ART but stopped before randomization, and participants who were ART naïve. However, the latter two subgroups of participants were small.

Collectively, these data indicate that morbidity and mortality risk reduction with earlier use of ART may be greater than previously estimated. Furthermore, it is possible that any increased risk of non-AIDS morbidity and mortality associated with use of ART may be more than counter-balanced by a reduced risk due to HIV RNA suppression, higher CD4+ count, or other beneficial effects of ART. If this is the case, the potential risk reduction for a composite outcome of AIDS and non-AIDS events could be sizable even in a target population at relatively low risk of AIDS. The reason for this is that the rate of non-AIDS events is likely much greater than AIDS events at higher CD4+ cell counts. By itself, a large percentage risk reduction in AIDS, as might be expected with early ART, might not be of sufficient magnitude in terms of absolute excess risk to warrant early intervention. However, if the reduction in risk for AIDS is coupled with a larger absolute risk reduction for serious non-AIDS events, as might be expected even if ART only had a modest positive effect on these higher incidence events, then early ART would be indicated.

While the potential for reducing morbidity and mortality with ART is great, current data available are insufficient and do not inform whether the benefits of initiating ART at CD4+ cell counts above 500 cells/mm^3 outweigh the risks. It is critical to evaluate risks and benefits of early ART with a randomized trial.

START Schematic

HIV-infected individuals who are ART-naïve with CD4+ count > 500 cells/mm^3

Early ART Group	Deferred ART Group
Initiate ART immediately following randomization	Defer ART until the CD4+ count declines to < 350 cells/mm^3 or AIDS develops
N=450 in pilot phase and estimated as N=2,000 for definitive trial	N=450 in pilot phase and estimated as N=2,000 for definitive trial

Design
START is an international randomized trial comparing early ART versus deferred ART. Participants will be randomized in a 1:1 allocation ratio to the early or deferred ART group. The primary composite endpoint is the development of a serious AIDS event ("AIDS*"), a serious non-AIDS event ("non-AIDS"), or death from any cause.

Serious AIDS events (or AIDS*) include most traditional opportunistic conditions but exclude non-fatal esophageal candidiasis and chronic *Herpes simplex* (see Appendix E for a complete list of conditions). Non-fatal esophageal candidiasis and chronic *Herpes simplex* are not counted in the primary endpoint of serious AIDS events because they are more common than most other opportunistic events at higher CD4+ counts and usually do not cause significant limitations for people in whom they occur.

In this protocol, the term "AIDS" (without an asterisk) denotes all opportunistic conditions, including non-fatal esophageal candidiasis and chronic *Herpes simplex* (see Appendix E). All AIDS conditions are included as a secondary endpoint in START.

The following serious non-AIDS conditions are the components of the primary composite endpoint referred to as serious non-AIDS events, or "non-AIDS":

- Cardiovascular disease (CVD) (myocardial infarction, stroke, coronary revascularization);
- End-stage renal disease (ESRD) (initiation of dialysis, renal transplantation);
- Decompensated liver disease; and
- Non-AIDS-defining cancers (excluding basal and squamous cell skin cancers).

Basal and squamous cell skin cancers are included as components of a secondary endpoint – all non-AIDS-defining cancers.

Key secondary endpoints are the components of the primary composite endpoint. Other important secondary endpoints include all-cause mortality, fatal or non-fatal AIDS, drug resistance, quality of life, health-care utilization and cost of care, and HIV transmission risk behavior. In a subsample of participants, neurocognitive function will be assessed. Plasma will be stored to facilitate future biomarker comparisons between treatment groups and nested case-control studies.

In order to obtain definitive information concerning impact of early ART on the primary endpoint as compared to deferred ART, it is estimated that 4,000 participants will need to be randomized and followed for up to 6 years. Because there is uncertainty about the rates of AIDS* and non-AIDS in this target population, sample size, duration of follow-up and the target number of events will be re-estimated by the protocol team before enrollment is completed. This sample re-estimation will consider the overall (both treatment groups combined) primary event rate and the relative proportion of primary events that are AIDS* and non-AIDS.

Participant Selection
As a general guideline, participants who are considered for enrollment should be in reasonably good health. The participant should be able, in the clinician's opinion, to adhere to the protocol (i.e., be willing to accept and adhere to the data collection schedule and assigned treatment strategy).

Inclusion Criteria

- Signed informed consent
- HIV infection documented by a plasma HIV RNA viral load, rapid HIV test or any licensed[1] ELISA test; and confirmed by another test using a different method such as a rapid HIV test, Western Blot, HIV culture, HIV antigen, or HIV pro-viral DNA at any time prior to study entry.
- Age ≥ 18 years
- Karnofsky performance score ≥ 80 (an indication that the participant can perform normal activities)
- Perceived life expectancy of at least 6 months
- For women of child-bearing potential, willingness to use contraceptives as described in the product information of the ART drugs they are prescribed
- Two consecutive CD4+ cell counts > 500 cells/mm^3 at least 2 weeks apart within 60 days before randomization

[1] The term “licensed” refers to an FDA-approved kit or, for sites located in countries other than the United States, a kit that has been certified or licensed by an oversight body within that country. Confirmation of the initial test result must use a test method that is different than the one used for the initial assessment.

Exclusion Criteria

- Any previous use of ART or IL-2
- Diagnosis of any clinical AIDS event before randomization (including esophageal candidiasis and chronic *Herpes simplex* infection)
- Presence of HIV progression such as oral thrush, unexplained weight loss, or unexplained fever
- Cardiovascular event (myocardial infarction, angioplasty, coronary-artery bypass grafting, stroke) within 6 months before randomization
- Non-AIDS-defining cancer, excluding basal and squamous cell skin cancer, within 6 months before randomization
- Dialysis within 6 months before randomization
- History of decompensated liver disease
- Current imprisonment, or compulsory detention (involuntary incarceration) for treatment of a psychiatric or physical illness
- Current pregnancy or breastfeeding (a negative serum or urine pregnancy test is required within 14 days before randomization for women of child-bearing potential)

Procedures

Within 60 days before randomization, participants will attend screening visits to establish eligibility and collect baseline data. Following randomization, participants who are assigned early ART will commence taking the ART regimen that was specified prior to randomization. All participants must receive a regimen listed on the INSIGHT website table "Antiretroviral Components Required for the Initial Regimen in START" based on U.S. Department of Health and Human Services (DHHS) guidelines. This table will be updated as guidelines change (see Appendix H). To ensure that participants receive a required regimen, a number of different ART regimens will be provided to sites through the INSIGHT Central ART Repository.

Following randomization, participants will be seen at 1 month, 4 months and every 4 months thereafter for follow-up data collection. Visits will include a targeted medical history, a brief medical examination, and determination of CD4+ cell count and HIV RNA level. Plasma will be stored at baseline and each follow-up visit for future HIV-related research.

ART will be prescribed for participants in the deferred ART group when the CD4+ cell count declines to < 350 cells/mm^3 and is confirmed within 4 weeks; when an AIDS-defining event develops; or when certain other conditions or symptoms occur as specified by local guidelines.

Participants in the deferred ART group who initiate ART must receive a regimen listed on the current INSIGHT website table "Antiretroviral Components Required for the Initial Regimen in START."

Primary Endpoint

Time to AIDS*, non-AIDS, or death from any cause (first event) (see Appendix E)

Major Secondary Endpoints
- AIDS* or death from AIDS
- Non-AIDS or death not attributable to AIDS

Other Secondary Outcomes
- All-cause mortality
- Non-AIDS
- CVD (myocardial infarction, stroke, coronary revascularization)
- ESRD (initiation of dialysis, renal transplantation)
- Decompensated liver disease
- Non-AIDS malignancy, excluding basal and squamous cell skin cancers
- Non-AIDS malignancy, including basal and squamous cell skin cancers
- AIDS
- Bacterial pneumonia
- Adverse events
- Hospitalization
- Quality of life
- Health-care utilization and cost of care
- HIV transmission risk behavior
- HIV drug resistance
- Pulmonary embolism or deep vein thrombosis
- New-onset diabetes mellitus
- Coronary artery disease requiring drug treatment
- Congestive heart failure
- Peripheral arterial disease
- Change in estimated glomerular filtration rate (eGFR) and development of proteinuria
- Blood pressure and blood lipids
- Electrocardiogram (ECG) abnormalities
- Use of blood pressure- or lipid-lowering treatment or aspirin
- Change in neurocognitive function (in a subset of participants)

2 Background and Rationale

2.1 Primary Study Hypothesis

Untreated HIV infection is associated with an increased risk of both AIDS* and non-AIDS events. Therefore, among asymptomatic participants with a CD4+ count greater than 500 cells/mm^3, immediate use of ART that results in suppression of HIV RNA levels and increases in CD4+ cell counts and potentially other beneficial effects will delay the development of AIDS*, non-AIDS, and death from any cause.

2.2 Background on Which Study Hypothesis Was Generated

2.2.1 Treatment Guidelines

The 2008 guidelines for HIV-infected adults and adolescents from the U.S. DHHS recommend initiating ART for patients with an AIDS-defining illness or with a CD4+ count < 350 cells/mm^3.[1] These guidelines indicate that ART may be considered in some patients with a CD4+ count > 350 cells/mm^3, but note the following: "Existing data are inadequate to recommend initiation of antiretroviral therapy in all patients with a CD4 T-cell count > 350 cells/mm^3. Any theoretical potential benefits could be outweighed by unknown risks or by patient-specific preferences." These guidelines note that no randomized trial definitively addresses the optimal time to initiate ART.

European guidelines are similar to DHHS guidelines, recommending treatment for symptomatic HIV infection and when the CD4+ is < 350 cells/mm^3. These guidelines indicate that for some participants, treatment may be offered if the CD4+ count is between 350 and 500 cells/mm^3, and that treatment should be deferred when the CD4+ is > 500 cells/mm^3.[2]

Data from untreated cohorts indicate that the 6-month rate of AIDS continues to decrease with higher CD4+ counts in the 200-500 cells/mm^3 range and is much lower for individuals with a CD4+ cell count ≥ 350 than for individuals with counts 200-349 cells/mm^3.[3] Likewise, data from cohorts that follow patients after the initiation of ART indicate that the hazard of AIDS declines following initiation of ART, even at high CD4+ cell counts, e.g., > 350 cells/mm^3.[4] Taken together, these data suggest that initiation of ART at a CD4+ count > 350 cells/mm^3 would lead to a substantial relative reduction in AIDS events. However, among patients being followed in clinical practice, ART is usually not initiated until the CD4+ cell count is < 350 cells/mm^3 for several reasons: (1) small absolute (as opposed to relative) risk differences after 3 and 5 years in the rates of AIDS or death associated with earlier ART use,[4,5] and therefore uncertainty about the cost-effectiveness of early ART; (2) concerns about serious complications associated with ART, e.g., cardiovascular, renal, and liver disease, that might negate the expected benefits of ART; (3) side effects of ART that impact quality of life; (4) concern that there could be waning adherence with long-term use of ART and consequent development of HIV resistance; and (5) the potential availability of better treatments in the future that

would more durably suppress the virus and carry a lower risk of the development of resistance.

Many of the reasons stated above led to the assumption that, even though earlier use of ART would likely reduce morbidity and mortality in the short term, in the long term it might not. This assumption was made in the absence of any data on clinical outcomes from randomized trials that could prove or disprove it.

New data indicate that the reasons for delaying ART are weaker than before and suggest that use of ART earlier (at higher CD4+ counts) than recommended by guidelines could be beneficial. Data from the SMART study, a clinical trial that randomized 5,472 participants to episodic (DC group) versus continuous ART (VS group), indicate that episodic use of ART according to CD4+ count thresholds (stop ART when the CD4+ count is > 350 and re-initiate ART when the CD4+ count declines to < 250 cells/mm^3) across a broad range of CD4+ cell counts is associated with increased risk of non-AIDS events such as cardiovascular, liver, and renal disease and non-AIDS-defining cancers.[6,7] Furthermore, when continuous ART was reinitiated for those in the episodic ART group, this increased risk of the non-AIDS events was reduced.[8] While most of the participants in SMART were taking ART at entry, the increased risk of non-AIDS diseases associated with deferring use of ART to a CD4+ cell count of 250 cells/mm^3 appears to apply to ART-naïve participants and others not taking ART at entry. For example, for the small subgroup of participants in SMART who were ART-naïve or who had not taken ART for at least 6 months prior to randomization, the HR (DC/VS) for non-AIDS events defined similarly to that proposed for START was 7.0 (95% CI: 1.6-31.4).[9] Data from cohorts also suggest that initiating ART at higher CD4+ counts than recommended by guidelines may be beneficial. In observational studies the rates of many non-AIDS events are higher among patients with lower CD4+ cell counts.[10,11,12] A recent review summarizes data on the effects of HIV on serious diseases other than AIDS.[13] In addition, findings from one large cohort (NA ACCORD) suggest that all-cause mortality may be reduced by initiating ART between 351 and 500 cells/mm^3 as compared to deferring it to ≤ 350 cells/mm^3.[14] In that study all-cause mortality was approximately 70% greater after adjustment among patients who deferred ART to ≤ 350 cells/mm^3 (median CD4+ at initiation of 275 cells/mm^3 and 25th and 75th percentiles 210 and 317 cells/mm^3) versus those who initiated ART between 351 and 500 cells/mm^3 (median CD4+ at initiation of ART was 421 cells/mm^3 and 25th and 75th percentiles 386 and 459 cells/mm^3). Due to the low power of the SMART subgroup analysis and the potential for bias (e.g., confounding by indication) in the cohort studies, the data cited above suggest, but do not prove, that treatment of individuals with high CD4+ counts could have a substantial beneficial effect. This can only be firmly established by a large, randomized clinical trial.

On the other hand, cohort studies also indicate that the risk of CVD may increase with use of some ART.[15,16,17,18] These apparently inconsistent findings from the SMART and the D:A:D studies could both be true – immunodeficiency, pro-inflammatory effects of untreated HIV or other factors may be associated with an increased risk of CVD while

use of some ART may also increase the risk of CVD. Until the risks and benefits of ART with respect to CVD and other major clinical outcomes are quantified in a randomized trial, uncertainty will remain as to the merits of initiating ART at CD4+ counts > 350 cells/mm^3.

In order to quantify reliably the risk and benefits of ART at CD4+ counts > 350 cells/mm^3, it is important to define strategies for using ART that are clearly distinguishable and for which there is substantial uncertainty about the risk difference. Once defined, risks and benefits of the ART strategies can be reliably determined by randomly allocating a large number of participants to each strategy. In the section that follows, the potential risks and benefits of the two strategies are summarized. A more detailed review of the data on potential risks and benefits is provided in Appendix B.

2.2.2 Summary of Potential Risks and Benefits

For prolonging disease-free survival, potential benefits of an early treatment strategy of initiating ART at a CD4+ cell count > 500 cells/mm^3 are:
- Maintenance of a higher CD4+ cell count
- Lower risk of AIDS and non-AIDS conditions

Potential risks of the early ART strategy are:
- More side effects
- Increased risk of exhausting drug options (e.g., due to resistance)
- Increased risk of some non-AIDS events.

For prolonging disease-free survival, potential benefits of a deferred ART strategy in keeping with current guidelines that stipulate that ART be initiated when the CD4+ cell count declines to < 350 cells/mm^3 are:
- Drugs that are safer, easier to take, and more potent and/or less susceptible to resistance development could be available when ART is eventually initiated
- Delay in any risk of adverse events of ART

Potential risks of this strategy include:
- Longer period of time at lower CD4+ cell counts and higher HIV RNA levels
- Higher risk of AIDS and serious non-AIDS conditions while ART is deferred

In addition to these potential benefits and risks of early ART for patients with a CD4+ cell count > 500 cells/mm^3, there is a potential broader public health benefit of early ART. As noted in Appendix B, even if transmission risk behaviors do not decrease among patients taking early ART, reduced HIV transmission would be predicted based on HIV RNA decline with ART.

2.3 Rationale for Selected Study Design

2.3.1 CD4+ Cell Count Entry Criteria and CD4+ Count Deferral Criteria for Control Group

The immediate and deferred ART strategies need to be sufficiently different in their definition so that a difference in clinical outcomes between them is plausible. Thus, the CD4+ cell count entry and deferral criteria were selected to result in groups with substantially different exposure to ART over the planned follow-up period.

At entry, participants will have a CD4+ count > 500 cells/mm^3. ART will be initiated in the deferred ART group when the CD4+ declines to < 350 cells/mm^3. The deferral strategy was chosen with consideration of current guidelines[1,2] and recent data from SMART[6,9] that indicate that initiation of ART should take place closer to 350 than 200 cells/mm^3. If the majority of participants enrolled have a CD4+ cell count between 500 and 650 cells and CD4+ declines on average 50-60 cells/mm^3 per year in the absence of ART, the majority of participants in the deferred ART group are likely to remain off ART for at least 2-3 years of follow-up.[19]

As a consequence of the CD4+ entry and ART deferral criteria, and considering the rate of CD4+ increase following initiation of ART and the magnitude of the increase starting at lower versus higher CD4+ levels, these two strategies for initiating ART will result in treatment groups with substantially different CD4+ cell counts and HIV RNA levels over a 5-year follow-up period.

2.3.2 Broad Entry Criteria

With few exceptions, any healthy HIV-infected adult with a CD4+ cell count > 500 cells/mm^3 at two consecutive visits within 60 days before randomization who has not taken ART or interleukin-2 (IL-2) is eligible to enroll. Participants may have had a prior CD4+ count ≤ 500 cells/mm^3, and this will be recorded. The participant may have any HIV RNA level. HIV RNA level predicts the rate of CD4+ decline.[20,21] Thus, on average, participants with higher HIV RNA levels in the deferred ART group are likely to meet the CD4+ threshold for ART (350 cells/mm^3) more rapidly than those participants with lower HIV RNA levels. Restricting enrollment to participants with high HIV RNA could result in less distinguishable treatment groups in terms of ART use and miss an opportunity to assess whether risk of serious non-AIDS diseases associated with HIV RNA, even at low levels, can be reduced with ART. While excluding participants with high HIV RNA levels would, on average, increase the difference in ART exposure between groups, HIV RNA does not explain a large fraction of the variability in the CD4+ decline.[21] Thus, rapid decline in CD4+ is still likely for some participants. The exclusion of participants with high HIV RNA would eliminate a large subgroup of participants for which there is uncertainty about when to start ART and limit ability for risk stratification. These broad entry criteria are likely to result in the inclusion of a group of participants at variable risk for the composite endpoint. This will permit risk stratification to be carried out at the completion of the study to determine how ART should most effectively be used in subgroups considering absolute risk reductions and cost effectiveness.

2.3.3 Composite Endpoint of AIDS* and Non-AIDS

The ultimate goal of the treatment of HIV disease is to prevent morbidity and mortality. Thus, the primary and secondary endpoints of this study are clinical outcomes rather than surrogate markers.

The primary endpoint of the START study is a composite outcome of AIDS*, non-AIDS, and death from any cause (see Appendix E). This composite outcome includes major morbid events that are life threatening and impact quality of life.

It is hypothesized that ART will positively influence both AIDS* and non-AIDS risk in the CD4+ range to be studied. Based on epidemiological studies, ART is expected to reduce the risk of AIDS* more than the risk of non-AIDS. However, the incidence of non-AIDS is expected to be greater than the incidence of AIDS*. Thus, the absolute risk reduction is expected to be similar for AIDS* and non-AIDS events.

2.3.4 Major Secondary Outcomes

2.3.4.1 Secondary Clinical Outcomes

A number of secondary outcomes will be assessed at interim and final analyses to allow for the careful weighing of the benefits and risks of early ART. These clinical outcomes include the separate components of the composite outcome; all-cause mortality; all AIDS events (including esophageal candidiasis and chronic *Herpes simplex*); all non-AIDS-defining cancers (including basal and squamous cell skin cancers); bacterial pneumonia; pulmonary embolism; deep vein thrombosis; new-onset diabetes mellitus; coronary artery disease requiring drug treatment; congestive heart failure; peripheral vascular disease and adverse events.

2.3.4.2 Quality of Life, Health-Care Utilization and Cost of Care

Quality of life will be assessed using the self-administered Medical Outcomes Study Short-Form 12 Item Survey (SF-12, version 2) and a visual analog scale for current health (zero to 100, with zero being worst possible health).[22,23,24] A comparison of the costs and cost effectiveness of early versus deferred ART will be a critical component of the START study. The eventual implications of START for HIV care may rest on cost-effectiveness analysis. The majority of the costs of medical care among study participants will probably be those of ART, because hospitalizations and other expensive clinical interventions (e.g., nursing home care) are predicted to be quite uncommon in the study population. ART costs will be assessed from detailed information on usage in the two study arms and can be compared in a number of sensitivity analyses using different drug pricing systems (e.g., Average Wholesale Price [AWP], Public Health Service [PHS] price, and generic price). Geographic differences will be considered. Non-ART drugs will likely be only 5% of ART costs, and therefore, will not be collected in detail. Health-care utilization (hospitalization, clinic visits, nursing home care, and home care) will be reported and then used to estimate costs. Indirect costs of medical illness will also be estimated.

2.3.4.3 Transmission Risk Behavior

Information will also be collected on high-risk behaviors for transmitting HIV. Epidemiological data indicate that risk of sexual transmission of HIV is closely related to plasma viral load.[25,26] Thus, early ART may effectively lower risk of transmission if risk behaviors are similar in the two treatment groups.

2.3.4.4 HIV Drug Resistance

The development of resistance to ART and possible subsequent loss of drug options will be assessed as an important secondary outcome in the study. This outcome will be evaluated by collecting samples of blood prior to changes in ART due to elevated HIV RNA level and by collecting results from locally performed resistance tests. The former will be used to carry out batch resistance testing at a central laboratory during the trial. These tests will not be done in real time, and results will not be given to the investigator or participant. Key mutations that are associated with viral resistance will be determined using information periodically updated by the International AIDS Society.[27] The early and deferred ART groups will be compared for the accumulation of major mutations to each class of ART.

2.3.4.5 Markers of CVD risk

Assessments will be made of blood lipids, smoking, blood pressure, resting electrocardiographic (ECG) abnormalities, incidence of diabetes mellitus, use of medication to lower blood pressure and lipids, and the use of aspirin, to assist in the evaluation of cardiovascular risks and benefits of early treatment. For all consenting participants, plasma will be stored for central measurement of inflammatory and coagulation markers.

2.3.4.6 Neurocognitive Performance

A validated battery of tests will be administered to a subset of consenting participants in each treatment group to evaluate change in neurocognitive function.

2.4 Summary

For HIV-infected individuals with a CD4+ cell count > 500 cells/mm^3 who are not taking ART, there is genuine uncertainty whether the risk of morbidity and mortality from non-AIDS conditions that may result from ongoing viral replication and consequent general immune activation or immunodeficiency at higher CD4+ levels can be modified with ART. The findings from SMART suggest that risks of AIDS* and non-AIDS events could be reduced by initiating ART among patients at CD4+ cell counts > 350 cells/mm^3. However, it is unclear whether the findings from SMART can be generalized to ART-naïve patients or to patients with counts > 500 cells/mm^3. Epidemiological studies also suggest that reduced risks of AIDS and of some non-AIDS conditions would result from early ART. However, as in other illnesses, it is hazardous to assume that people’s risk of disease can be modified with ART as predicted by the epidemiological data.

The risk of non-AIDS events is higher than the risk of AIDS* events in these patients. START will, therefore, assess these risks and determine the effectiveness of early ART

in reducing the rate of a composite outcome that includes both AIDS* and non-AIDS. START will expand our understanding of how immunodeficiency and immune activation influence the risk of development of both AIDS* events and, more importantly, non-AIDS events at high CD4+ levels.

If START establishes that ART is beneficial among individuals with a CD4+ cell count > 500 cells/mm^3 for a broad range of health outcomes, the earlier use of ART in many parts of the world would be based on cost-effectiveness analyses, based both on the effectiveness of ART in the patients being treated and any effects in reducing the rate of ongoing transmission.

The potential for large reductions in morbidity and mortality with early ART is great; there is substantial uncertainty as to whether the risk reduction suggested by the epidemiological data and SMART results will be realized; and there is a high probability of being able to enroll and follow patients and carry out the planned early intervention. Therefore, the timing for a randomized trial is ideal.

3 Methodology

3.1 Study Design

This is a multicenter, international, randomized trial for HIV-infected adults comparing initiation of ART at a CD4+ cell count > 500 cells/mm^3 (early ART) versus initiation of ART at a CD4+ cell count of < 350 cells/mm^3 (deferred ART) for a composite outcome of AIDS*, non-AIDS, and death from any cause (see Appendix E).

In addition to the composite outcome, two important secondary outcomes will be evaluated: (1) AIDS* or death from AIDS; and (2) non-AIDS or death not attributable to AIDS, including death of unknown cause. Other secondary outcomes include components of the major endpoints, HIV drug resistance, quality of life, cost effectiveness, and HIV transmission risk behaviors.

Because there is uncertainty about the feasibility of enrollment of ART-naïve participants with CD4+ cell counts > 500 cells/mm^3, a 1-year pilot phase will be conducted. During this pilot phase, 70 sites supported by DAIDS will aim to enroll at least 900 participants. Sites with support from outside DAIDS will also participate in the pilot phase. Upon successful completion of the pilot phase, a decision on the definitive study will be made by DAIDS.

With few exceptions, consenting, asymptomatic, ART-naïve participants 18 years of age or older with a CD4+ cell count > 500 cells/mm^3 are eligible for randomization to the trial.

Participants in the early ART arm will commence ART immediately following randomization and continue taking it for the duration of the trial. The deferred ART group, the control arm, is defined in keeping with current treatment guidelines that indicate that treatment should commence after the CD4+ declines to < 350 cells/mm^3 or if an AIDS-defining diagnosis occurs. In addition, when certain other conditions or symptoms occur as specified by local guidelines, ART may be initiated. In this study, when the CD4+ declines to < 350 cells/mm^3 for a participant in the deferred arm, the CD4+ count will be immediately confirmed, and if the second CD4+ count is also < 350 cells/mm^3, ART will be initiated.

For participants in both treatment groups, the initial regimen used must be chosen from those listed on the INSIGHT website table "Antiretroviral Components Required for the Initial Regimen in START." This table is based on current DHHS guidelines and will be updated as guidelines change (see Appendix H).

As noted above, the primary objective of the pilot phase of START is to establish the feasibility of enrolling ART-naïve participants with CD4+ cell counts > 500 cells/mm^3. In addition, completeness of follow-up and adherence to the assigned treatment will be carefully considered in determining whether to continue with the definitive phase of the study. Objectives for the definitive phase are stated in the following sections.

3.2 Study Objectives

3.2.1 Primary Objective

To determine whether early ART is superior to deferred ART in delaying the occurrence of a composite outcome consisting of AIDS*, non-AIDS, or death from any cause.

3.2.2 Secondary Objectives

a. To compare early ART to deferred ART for each component of the primary composite endpoint:
   - AIDS* or death from AIDS
   - Non-AIDS or death not attributable to AIDS

b. To compare early ART to deferred ART for the following secondary outcomes:
   - All-cause mortality
   - Non-AIDS
   - CVD (myocardial infarction, stroke, coronary revascularizaton)
   - ESRD (initiation of dialysis, renal transplantation)
   - Decompensated liver disease
   - Non-AIDS malignancy, excluding basal and squamous cell skin cancers
   - Non-AIDS malignancy, including basal and squamous cell skin cancers
   - AIDS
   - Bacterial pneumonia
   - Adverse events
   - Hospitalization
   - Quality of life
   - Health-care utilization and cost of care
   - HIV transmission risk behavior
   - HIV drug resistance
   - Pulmonary embolism or deep vein thrombosis
   - New-onset diabetes mellitus
   - Coronary artery disease requiring drug treatment
   - Congestive heart failure
   - Peripheral arterial disease
   - Change in estimated GFR and development of proteinuria
   - Blood pressure and blood lipids
   - ECG abnormalities
   - Use of blood pressure- or lipid-lowering treatment or aspirin
   - Change in neurocognitive function (in a subset of participants)

c. To compare early ART with deferred ART for the primary composite outcome and other major clinical outcomes in subgroups defined by the following characteristics measured at baseline:
   - Age
   - Gender

- Race/ethnicity
- Presence and levels of risk factors for serious non-AIDS conditions (e.g., smoking, estimated GFR (eGFR), hepatitis co-infection, diabetes mellitus, estimated CVD risk, lipids, blood pressure, presence of resting ECG abnormalities, age, and gender)
- Baseline CD4+ cell count
- Baseline HIV RNA level
- Geographic region
- Calendar date of enrollment
- ART regimen specified prior to randomization

3.2.3 Other Objectives

For a robust comparison of the early and deferred ART groups, the groups must differ by a substantial amount in exposure to ART during follow-up. The first three objectives listed below are aimed at understanding whether protocol assumptions are valid and at evaluating adherence to the protocol.

a. To compare the early and deferred ART groups for:
   - ART use over follow-up
   - HIV RNA levels over follow-up
   - CD4+ cell counts over follow-up

b. To describe the early and deferred groups with respect to:
   - Initial ART regimen used
   - HIV RNA and CD4+ cell count response to the initial ART regimen
   - Number of changes in ART regimen over all follow-up and during the first year of treatment
   - Non-adherence to treatment strategy (e.g., discontinuation of ART in early ART group or initiation of ART too early in the deferred group)

c. Among participants in the deferred group, to estimate the rate of decline of CD4+ cell counts until the CD4+ declines to < 350 cells/mm^3, to estimate the fraction of participants who develop a primary event prior to starting ART, to estimate time to first ART initiation, and to estimate the fraction of participants who initiate ART before the CD4+ declines to < 350 cells/mm^3

d. Among participants in both treatment groups, to study predictors of AIDS* and non-AIDS

e. To estimate the prevalence and levels of non-AIDS risk factors at baseline (e.g., smoking, eGFR, hepatitis co-infection, diabetes mellitus, estimated CVD risk, lipids, blood pressure, presence of resting ECG abnormalities, age, and gender)

f. To evaluate the informed consent process by comparing comprehension of study requirements for those consented with a concise versus standard consent (in a subset of sites)

3.3 Primary Study Endpoint

The primary composite endpoint is defined in Appendix E and includes the following three major components:

- AIDS* or death from AIDS
  Opportunistic events consistent with the 1993 CDC expanded surveillance definition plus additional events associated with immunosuppression in the patient population targeted for enrollment. Esophageal candidiasis and chronic *Herpes simplex* infection will be counted as primary endpoints only if they result in death.

- Non-AIDS
  - CVD: myocardial infarction, stroke, coronary revascularization
  - ESRD: initiation of dialysis, renal transplantation
  - Decompensated liver disease
  - Non-AIDS-defining cancers, excluding basal and squamous cell skin cancers. Basal and squamous cell skin cancer will be counted as a primary endpoint only if they result in death.

- Death not attributable to AIDS, including death of unknown cause

The primary outcome of START and each of the major components of the primary outcome will be evaluated as the time to the first occurrence of an event above. Other major endpoints are referred to in the Study Objectives (section 3.2).

3.4 Randomization

Eligible participants will be randomized in a 1:1 ratio to either the early ART group or the deferred ART group. Randomization will be stratified by clinical site.

3.5 Sample Size and Statistical Considerations

In the pilot phase of this study, at least 900 participants (450 in the early ART group and 450 in the deferred group) will be enrolled from 70 sites supported by DAIDS.

For the definitive study on early ART, it is estimated that 4,000 participants will be required. This is based on estimates of the expected event rates in the early and deferred ART groups using data from the CASCADE Collaboration and UK CHIC[28] with computer simulations that account for the distribution of CD4+ cell counts projected for START. These estimates and other assumptions used in calculating sample size are described in detail in Appendix G and summarized below:

a. The primary analysis will be intention to treat using a Cox model with a single indicator for treatment group and with strata corresponding to geographic regions (North America, South America, Europe, Australasia, and Africa).

b. Type I error is 0.05 (2-sided) and power = 0.90. Power was set at 90% for the primary endpoint, in part, to ensure that there would be adequate power to address the components of the composite endpoint, non-AIDS and fatal AIDS or non-fatal AIDS*.

c. Participants will be enrolled over a 3-year period (2 additional years after the one year pilot phase) and followed for a minimum of 3 years resulting in an average follow-up of 4.5 years and a total study duration of 6 years.

d. The CD4+ cell count at entry will be between 501 and 600 cells/mm^3 for 70% of participants, between 601 and 700 cells/mm^3 for 20%, and > 700 cells/mm^3 for 10%.

e. In the deferred ART group, the rate of the primary endpoint is 2.8 per 100 person years over the follow-up period. Non-AIDS events will be four times more common than non-AIDS deaths, and approximately 35% of reported AIDS events and 10% of reported non-AIDS events will not meet probable or confirmed criteria (i.e., the criteria that have to be achieved to count the reported event as an endpoint). In SMART, the rates of confirmed or probable fatal AIDS or non-fatal AIDS* and of serious non-AIDS, as defined in START, were 0.4 and 2.0 per 100 person-years, respectively, in the VS group.[29]

f. Based on the computer simulations described in Appendix G, early ART is predicted to reduce the primary endpoint rate by 28.8% compared to deferred ART. This reduction in the hazard assumes: (1) AIDS* events represent 23% of the events in the deferred arm, and early ART will reduce this hazard by 43%; and (2) non-AIDS events and deaths not attributable to AIDS will represent 77% of events in the deferred ART group, and early ART will reduce this hazard by 24%. The smaller percentage risk reduction anticipated for non-AIDS events and deaths not attributable to AIDS takes into account that some of the deaths will be due to causes that are unrelated to HIV and ART. For example, 18% of deaths in SMART were due to substance abuse, accidents or violence.[6]

g. The estimated treatment differences take into account likely levels of adherence to the early ART strategy (e.g., participants followed in CASCADE who started ART were considered “on ART” irrespective of adherence or future discontinuation of their ART).

h. Adherence to the deferred strategy was also considered. It was assumed that 70% of participants would adhere to the deferral CD4+ threshold of 350 cells/mm^3 and 30% would initiate ART earlier – 10% before the CD4+ declined to 400 cells/mm^3 and 20% while the CD4+ was between 350 and 400 cells/mm^3.

i. A loss to follow-up rate of 2.7 per 100 person-years (equivalent to a 15% cumulative lost to follow-up after 6 years) is assumed.

j. Based on these assumptions, 3,822 participants (1,911 participants in each treatment group) are required. The number of primary events required is 369.

k. While many of the underlying assumptions are conservative, for some assumptions there is much uncertainty because of the absence of data (e.g., inadequate or no data on rates of non-AIDS morbidity). Considering this, a sample size of 4,000 participants (2,000 in each treatment group) and a target of 370 primary events has been established as the initial goal. This may be modified following sample size re-estimation.

Power was also estimated for the components of the primary endpoint. With the above assumptions, and with 4,000 participants and 370 primary events, power is 0.74 to detect a 43% reduction in the hazard of AIDS*, and power is 0.73 to detect a 24% reduction in the hazard of non-AIDS and deaths not attributable to AIDS.

3.6 Participant Selection

A heterogeneous group of participants with respect to baseline characteristics is important in a trial like START, and the broad inclusion and exclusion criteria specified below reflect that. It will permit risks and benefits of early versus deferred ART to be assessed in a variety of participants and will ensure that the trial reflects as much as possible the type of patients seen in clinical practice for whom there is uncertainty about when to begin lifelong ART.

As a general guideline, individuals who are considered for enrollment should be in reasonably good health. They should be able, in the clinician's opinion, to adhere to the protocol (i.e., be willing to accept and adhere to the data collection schedule and the assigned treatment strategy).

3.6.1 Inclusion Criteria

- Signed informed consent
- HIV infection documented by plasma HIV RNA viral load, a rapid HIV test or any licensed[1] ELISA test; and confirmed by another test using a different method such as a rapid HIV test, Western Blot, HIV culture, HIV antigen, or HIV pro-viral DNA at any time prior to study entry.
- Age $\geq$ 18 years
- Karnofsky performance score ≥ 80 (an indication that the participant can perform normal activities)
- Perceived life expectancy of at least 6 months

[1] The term “licensed” refers to an FDA-approved kit or, for sites located in countries other than the United States, a kit that has been certified or licensed by an oversight body within that country. Confirmation of the initial test result must use a test method that is different than the one used for the initial assessment.

- For women of child-bearing potential, willingness to use contraceptives as described in the product information of the ART drugs they are prescribed
- Two consecutive CD4+ cell counts > 500 cells/mm^3 at least 2 weeks apart within 60 days before randomization

3.6.2 Exclusion Criteria

- Any previous use of ART or IL-2
- Diagnosis of any clinical AIDS event before randomization (including esophageal candidiasis and chronic *Herpes simplex* infection)
- Presence of HIV progression such as oral thrush, unexplained weight loss, or unexplained fever
- Cardiovascular event (myocardial infarction, angioplasty, coronary-artery bypass grafting, stroke) within 6 months before randomization
- Non-AIDS-defining cancer, excluding basal and squamous cell skin cancer, within 6 months before randomization
- Dialysis within 6 months before randomization
- History of decompensated liver disease
- Current imprisonment, or compulsory detention (involuntary incarceration) for treatment of a psychiatric or physical illness
- Current pregnancy or breastfeeding (a negative serum or urine pregnancy test is required within 14 days before randomization for women of child-bearing potential)

3.7 Study Plan

The management of participants throughout the course of the trial will be guided by their assigned strategy. Throughout follow-up, when ART is used, it should be a potent combination ART regimen that is expected to provide durable suppression of HIV RNA levels.

3.7.1 Treatment Strategies

Before a participant is randomized, a potent combination ART regimen must be prespecified by the study clinician. The initial regimen used must be one from the table "Antiretroviral Components Required for the Initial Regimen in START" on the INSIGHT website (see Appendix H). The list of regimens in this table is based on current DHHS guidelines and will be updated following any changes in those guidelines.

After randomization, participants in the two groups will use potent combination ART as follows:

a. Participants assigned to the **early** ART group will start the prespecified ART regimen as soon as possible.

b. Participants assigned to the **deferred** ART group will defer ART until one of the following conditions occurs:

- CD4+ cell count declines to < 350 cells/mm^3 and is confirmed by a repeat CD4+ measurement within 4 weeks, or
- AIDS develops, or
- Conditions specified by local guidelines occur that indicate initiation of potent combination ART, e.g., symptoms indicative of HIV progression such as oral thrush, unexplained weight loss, or unexplained fever.

After discussion with their clinicians, participants in the deferred ART group who develop any of the conditions noted above will start an ART regimen selected from the table “Antiretroviral Components Required for the Initial Regimen in START” on the INSIGHT website (see Appendix H). The regimen selected may differ from the regimen prespecified for these participants prior to randomization, but should be started as soon as possible within 4 weeks of confirmation of any of the conditions noted above.

Women assigned to the **deferred** ART group who become pregnant before the CD4+ cell count declines to < 350 cells/mm^3 may be prescribed ART as recommended by local treatment guidelines, even if the ART regimen chosen differs from those listed on the INSIGHT website table.

More information on choice and management of ART and provision of antiretroviral drugs throughout the trial is found in section 4.1 below.

3.7.2 Concomitant Medications

There are no restrictions on concomitant medications for participants in either group. However, it is important to consider the potential for drug interactions between ART and concomitant medications, including agents prescribed for prophylaxis against opportunistic infections. Links to current information on drug interactions can be found on the INSIGHT website (see Appendix H).

The use of targeted categories of concomitant medications prescribed for participants in START will be ascertained at baseline and annual follow-up visits. Targeted categories will include but are not limited to:

- Agents for prophylaxis against opportunistic infections
- Lipid-lowering drugs
- Drugs for the treatment of diabetes mellitus
- Antihypertensive agents
- Other cardiovascular drugs, including aspirin
- Hormones (used for contraception or for therapy)
- Corticosteroids (used at doses above physiologic replacement)
- Immunomodulators

3.7.3 Baseline Screening

All consenting participants will have the following information and measurements collected within 60 days before randomization unless otherwise noted. All measurements are done locally unless otherwise noted.

- Demographics, including education
- Documentation of HIV infection
- CD4+ cell count and CD4%: two measurements at least 2 weeks apart, with the earliest within 60 days before randomization
- Karnofsky score
- For women of child-bearing potential, a pregnancy test (serum or urine) done in the clinic must be documented to be negative within 14 days before randomization
- Targeted health history including date of first diagnosis of HIV infection, likely mode of HIV infection, history of non-AIDS events, and pregnancy history
- Brief clinical evaluation including weight, height, sitting blood pressure, pulse, and smoking status
- Nadir CD4+ cell count and CD4% and maximum HIV RNA level available in the medical record from any time in the past
- Up to three most recent (before the above baseline measurements) CD4+ cell counts, CD4%s, and HIV RNA measurements available in the medical record
- Findings from previous genotypic or other form of HIV resistance testing (such as virtual phenotype and/or phenotypic resistance testing), if performed and available (see section 4.1.9 and Appendix F)
- Selected concomitant medications
- Quality of life assessment
- Use of alcohol and recreational drugs
- HIV transmission risk behavior assessment
- Health-care utilization
- HIV RNA measurement
- Additional laboratory assessments (participants should be asked to abstain from food, except water, for at least 8 hours prior to providing blood for glucose and lipid measurements):
  - Complete blood count (CBC): hemoglobin, hematocrit, white blood cell count (WBC) with differential and platelets
  - CD8+ T-cell count and CD8%
  - Renal function: serum creatinine to estimate GFR[30]
  - Liver function: alanine aminotransferase (ALT), aspartate aminotransferase (AST), alkaline phosphatase, total bilirubin and albumin
  - Glucose
  - Lipids: total cholesterol, low density lipoprotein (LDL), high density lipoprotein (HDL), triglycerides
  - Dipstick urinalysis for measurement of protein
- Documentation of hepatitis B and C status: hepatitis B surface antigen, core antibody and surface antibody; hepatitis C antibody and, if available, genotype and viral load. Documented positive tests at any time in the past or documented negative tests in the 6 months before randomization may be used.

- In a subset of participants, a resting ECG (at sites with a study-provided ECG machine and a certified technician)
- ART regimen to be prescribed for the participant if randomized to the early group and whether the regimen will be obtained locally or through the INSIGHT Central ART Repository
- Stored plasma (sufficient for eight 1-mL transport tubes) for future HIV-related research (e.g., HIV resistance testing, CVD biomarkers)
- Stored urine (sufficient for six 1-mL transport tubes) for future HIV-related research
- In a subset of participants, neuropsychological tests, a questionnaire to screen for depression (Center for Epidemiologic Studies Depression Scale [CES-D]), and additional demographic data
- In a subset of participants, assessment of comprehension of study requirements
- In a subset of participants, six mL of whole blood will be collected for DNA extraction in order to perform future genetic testing related to HIV.

In participants who will be prescribed abacavir, HLA-B*5701 screening test results for abacavir hypersensitivity must be available before prescription.

3.7.4 Participant Follow-up

Participants will be seen for routine follow-up clinical evaluation in accordance with standard INSIGHT procedures and schedules. The total duration of the definitive trial is estimated to be 6 years (a 3-year enrollment period with participants followed for a minimum of 3 years after the last participant is enrolled). All participants will be followed to a common closing date that will be determined to coincide with attainment of the target number of primary endpoints. The follow-up period may be extended following a sample size re-estimation that will be performed before the end of the planned enrollment.

If a decision is made following the pilot phase not to continue with the study, the common closing date will likely be soon after that, about 1.5 years after beginning the study.

The follow-up schedule for data collection is the same in both groups. Data collection visits will occur at 1 and 4 months after randomization, and every 4 months thereafter until the study closes. In addition to the data collection described below for scheduled data collection visits, certain events that are described in section 3.8 are to be reported as soon as clinical sites become aware of them.

a. The following information will be collected at every follow-up visit unless otherwise noted:
   - Targeted health history and brief clinical evaluation including weight, sitting blood pressure and pulse
   - ART regimen currently prescribed, if applicable
   - Laboratory assessments:
     - CD4+ cell count, CD4% and HIV RNA level, CD8+ T-cell count and CD8%
     - Interim CD4+ cell counts, CD4% and HIV RNA levels obtained since last visit

- Results of locally performed antiretroviral resistance tests, if available
- Self-reported adherence to ART
- Stored plasma (sufficient for four 1-mL transport tubes) for future HIV-related research

b. The following additional information will be collected at months 1, 4 and 8 and every 12 months after randomization:
   - Laboratory assessments:
     - Renal function: creatinine
   - Urine dipstick for proteinuria
   - Stored urine (sufficient for six 1-mL transport tubes) for future HIV-related research

c. The following additional information will be collected at month 4 and every 12 months after randomization:
   - Quality of life assessment
   - HIV transmission risk behavior assessment

d. The following additional information will be collected every 12 months after randomization:
   - Smoking status
   - Selected concomitant medications
   - Laboratory assessments (participants should be asked to abstain from food, except water, for at least 8 hours prior to providing blood for glucose and lipid measurements):
     - CBC: hemoglobin, hematocrit, WBC with differential and platelets
     - Liver function: ALT, AST, alkaline phosphatase, total bilirubin and albumin
     - Glucose
     - Lipids: total cholesterol, LDL, HDL, triglycerides
   - In a subset of participants, a resting ECG (at sites with a study-provided ECG machine and a certified technician)
   - Health-care utilization
   - Use of alcohol and recreational drugs
   - Pregnancy history

e. The following additional information will be collected at months 4 and 8 and every 12 months after randomization for a subset of consenting participants:
   - Neuropsychological tests
   - A questionnaire to screen for depression (CES-D).

f. The following additional information will be collected during follow-up as it occurs:
   - Changes in the ART regimen and reasons
   - Documentation of clinical events, death, and pregnancy outcomes (see section 3.8)

- For consenting participants with a newly diagnosed malignancy, a biopsy sample of malignant tissue (a fresh frozen sample or paraffin block, pathology slide(s), if available), and a 20-mL whole blood sample obtained prior to treatment of the malignancy, if available
- Each time the ART regimen is switched or stopped due to an elevated HIV RNA level (or presence of resistance mutations), a 4-mL plasma sample for later central batch HIV resistance testing. The plasma sample should be collected prior to changing the ART regimen.

Most of the procedures above apply to follow-up visits for the purpose of data collection. More frequent follow-up visits may be conducted to ensure participant safety or for the purposes of providing routine care.

3.7.5 Participant Relocation to a New Site

If a participant relocates to a site not participating in START, data collection of items that are considered to be part of routine care (e.g., HIV RNA level, CD4+ cell count, clinical events) will continue if the participant provides a signed release of information. The *START Protocol Instructions Manual* provides guidance on how to collect data for participants who relocate and/or transfer to another START clinical site.

3.7.6 Stored Samples and Future Research

Specimens will be collected before randomization, at follow-up visits, and at the occurrence of events described above. These specimens will be stored at a central specimen repository for later use in HIV-related research concerning the effects of HIV and ART on AIDS and non-AIDS conditions. Proposed research utilizing these specimens will be reviewed and approved by the INSIGHT Executive Committee. Results of research tests on individual specimens will not be given to participants or their clinicians, but aggregate research results will be made available.

Instructions for drawing, processing, storing and shipping specimens are in the *INSIGHT Laboratory Manual*.

3.8 Event Reporting

For both treatment groups, for the duration of the trial, certain events are to be reported as soon as clinical sites become aware of them. The case report forms and documentation requirements are found in the *START Protocol Instructions Manual*. The INSIGHT Endpoint Review Committee (ERC) has established objective criteria for each event comprising the primary endpoint and for major secondary endpoints; these criteria are also found in the *START Protocol Instructions Manual.* The ERC is responsible for reviewing each reported primary event to determine the level of diagnostic certainty. Events which are judged as confirmed or probable will be included in the primary analysis.

All major clinical events, including the primary endpoint and its components, regardless of relationship to ART, will be summarized for the Data and Safety Monitoring Board (DSMB).

All events described below, including all AIDS and non-AIDS events that a participant experiences will be reported from study enrollment until study completion.

3.8.1 AIDS and Non-AIDS Events

Events comprising the primary endpoint of the START study will be reported on case report forms for participants in both treatment groups for the duration of the trial, irrespective of changes in ART use. In addition, AIDS events that are not part of the primary endpoint must also be reported, namely non-fatal esophageal candidiasis and *Herpes simplex* infection lasting more than 30 days.

Similarly, all non-AIDS-defining events that are part of the primary endpoint must also be reported for all participants for the duration of the trial. In addition to the non-AIDS events included as components of the primary endpoint, bacterial pneumonia, pulmonary embolism, deep vein thrombosis, new-onset diabetes mellitus, coronary artery disease requiring drug treatment, congestive heart failure, and peripheral arterial disease must also be reported.

All events should be reported immediately after a working diagnosis of the event has been made. All events that a participant experiences during the study will be reported. This includes recurrences of previously reported diagnoses.

3.8.2 Deaths

Death from any cause is part of the primary endpoint of START. Deaths must be reported on case report forms for all study participants for the duration of the trial. Deaths must be reported immediately following site awareness that a study participant has died.

3.8.3 Pregnancy Outcomes

For women who become pregnant during the study, pregnancy outcomes will be reported on a case report form.

3.8.4 Reporting Serious Events

Selected serious events are reported on a specific case report form, even if they have also been reported on other event-specific case report forms. If these events are judged by the investigator to be related to ART and unexpected according to ART labeling, they will be reported in an expedited manner. These events are:

- Deaths
- Events that are life-threatening
- Events requiring hospitalization
- Events requiring prolongation of hospitalization

- Events resulting in significant disability or incapacity
- Congenital abnormalities/birth defects (see section 3.8.3)
- Other important medical events that may jeopardize the participant or may require intervention to prevent one of the outcomes listed above
- All Grade 4 events (not limited to a laboratory abnormality) not already reportable in one of the above categories.

These events will be graded for severity according to the DAIDS Table for Grading the Severity of Adult and Pediatric Adverse Events, Version 1.0, December 2004 (also known as the DAIDS AE Grading Table; see Appendix H) and used to monitor the rate of adverse events in the two treatment groups during the study.

All abacavir hypersensitivity reactions (see section 4.1.8), regardless of grade, are also defined as serious events for the purposes of this protocol, and are reported in an expedited fashion as such. These also require reporting on a separate case report form directly to GlaxoSmithKline, the manufacturer of abacavir).

For participants who are receiving study-supplied ART at the end of the study, these events must continue to be reported in an expedited manner while those participants are receiving ART supplied by the study. Procedures for reporting these events are described in the START *Protocol Instructions Manual*.

3.8.5 Serious Adverse Event Reporting Required by the European Union

In accordance with the EU Directive 2001/20/EC (http://europa.eu/eur-lex/pri/en/oj/dat/2001/l_121/l_12120010501en00340044.pdf) and member state requirements, the sponsor, through its legal representative in Europe, will ensure that all relevant information about serious adverse events is recorded and reported to the central and/or concerned member state authorities and IECs as appropriate and in compliance with requirements for expedited reporting. The sponsor will ensure that investigators recognize their responsibility for reporting of serious adverse reactions and set up a system to allow the pharmaceutical companies providing study drugs (Marketing Authorization Holders) to report serious adverse reactions according to their obligations. Data collected for reporting of the events in section 3.8.4 will be used to construct the required reports to European authorities.

4 Clinical Management Issues

The following clinical management guidelines apply to both the early and deferred groups.

4.1 Management of ART

4.1.1 Use of Potent Combination ART

All participants taking ART in START must be prescribed potent combination therapy, except for the reasons noted in section 3.7.1. A regimen listed on the table “Antiretroviral Components Required for the Initial Regimen in START” on the INSIGHT website must be prescribed for the initial ART regimen for participants in either arm (see Appendix H).

The University of Minnesota, the sponsor of START, has a financial interest via royalty income in abacavir, one of the treatment drugs available for use in this study.

4.1.2 Factors Affecting Choice of an ART Regimen

Clinicians should take into consideration the following factors when choosing an ART regimen:

- Gender-related issues
- Other co-morbidities, including co-infection with hepatitis B or C
- Adherence issues
- Use of alcohol and recreational drugs
- Social circumstances
- Co-administration of antiretroviral drugs with overlapping toxicities
- Co-administration of concomitant medications with the potential for drug-drug interactions with particular drugs/classes of drug that are used in combination ART
- Presence of resistance and cross-resistance

4.1.3 Antiretroviral Drug Product Information

The package insert or product information for each antiretroviral drug should be consulted for information on dosing, contraindications, and drug interactions. Links to product information and drug interactions are listed on the INSIGHT website (see Appendix H.)

4.1.4 Approved Sources for Antiretroviral Drugs

Clinicians may only prescribe antiretroviral drugs for participants in START that have approval or tentative approval by the FDA or the European Medicines Agency (EMEA).

- The INSIGHT Central ART Repository will stock selected approved antiretroviral drugs donated by pharmaceutical companies. Further information about antiretroviral drug availability and distribution from the INSIGHT Central ART Repository can be found in the *START Protocol Instructions Manual* and on the INSIGHT website (see Appendix H).

- Antiretroviral drugs, including generic formulations, that meet the approval criteria above and are obtained from local sources may also be used in START.

Antiretroviral drugs used for the initial or subsequent ART regimens in START may be obtained from either the INSIGHT Central ART Repository or from local sources, as long as the drugs meet the approval criteria above.

4.1.5 Intolerance of an Antiretroviral Drug

In case of intolerance of one or more antiretroviral drugs in the initial regimen, the clinician is encouraged (but not mandated) to prescribe another preferred regimen from the table "Antiretroviral Components Required for the Initial Regimen in START" on the INSIGHT website (see Appendix H).

4.1.6 Drug Toxicity and Grading

All participants taking ART should be closely monitored for signs and symptoms of drug toxicity. For management of toxicities, clinicians should refer to product information available on the INSIGHT website (see Appendix H).

Symptoms and laboratory findings should be graded using the DAIDS AE Grading Table. This table is found in the *START Protocol Instructions Manual* and on the INSIGHT website (see Appendix H).

Changes in ART due to treatment toxicities may be made at any time.

4.1.7 Use of Antiretroviral Drugs in Women of Child-bearing Potential

For women of child-bearing potential, clinicians should adhere to recommendations in current product information of the antiretroviral drugs prescribed regarding counseling in the use of contraception and monitoring for pregnancy. For example, current product information for efavirenz, a Pregnancy Category D drug with risk for teratogenicity, states that "barrier contraception should always be used in combination with other methods of contraception (e.g., oral or other hormonal contraceptives)." Current product information is available on the INSIGHT website (see Appendix H).

4.1.8 Abacavir Hypersensitivity Reaction

Participants known to carry HLA-B*5701 must not receive abacavir. Participants for whom abacavir will be prescribed must be tested for the HLA-B*5701 allele; only those who are negative for the allele may be prescribed abacavir. In any participant treated with abacavir, the clinical diagnosis of suspected hypersensitivity reaction remains a clinical diagnostic decision. Even in the absence of the HLA-B*5701 allele, it is important to discontinue abacavir permanently and not re-challenge with abacavir if a hypersensitivity reaction cannot be ruled out on clinical grounds, due to the potential for a severe or even fatal reaction. Suspected cases of abacavir hypersensitivity of any severity (grade) must be reported in an expedited manner, and an additional case report form is required.

4.1.9 Resistance Testing

When initiating ART in either treatment group, resistance testing may be helpful to guide therapy changes; national or local treatment guidelines should be consulted.[31,32] Additional information is provided in Appendix F.

The decision to do resistance testing immediately prior to initiating ART should consider: (1) the regimen to be prescribed; (2) availability of previous resistance tests (e.g., closer to the time of seroconversion); and (3) risk of having transmitted drug resistance based on local data.

At each follow-up visit, the results of any locally obtained resistance testing will be recorded.

Plasma specimens will also be stored at baseline, 1 month, 4 months, and every 4 months thereafter for possible resistance testing. In addition, plasma specimens will also be collected from participants each time the ART regimen is switched or stopped due to an elevated HIV RNA level (or presence of resistance mutations). On a periodic basis, genotypic resistance testing may be performed on a sample of stored plasma specimens to assess prevalence of drug-related mutations.

Real-time resistance testing will not be provided as part of the START protocol.

4.1.10 Management of Virological Failure

If a participant experiences virological failure on any ART regimen, local guidelines should be followed to select antiretroviral drugs that will fully suppress HIV replication. Clinicians should consider the full spectrum of available and qualifying drugs.

In cases of multiple virological failures, participant adherence, the number of potential drug options available, and the risks and benefit of ART should be considered. For these participants, the use of drugs through expanded-access programs is permitted. There are no restrictions on use of ancillary therapies for HIV or use of other concomitant medications.

4.1.11 Discontinuation of ART

Treatment half-lives of the drugs taken should be considered any time ART is discontinued for any reason (e.g., for toxicity, intercurrent illness, elective surgery). In general, discontinuation of ART is not recommended, but it is recognized that there may be circumstances when it occurs. Differential half-lives of antiretroviral agents may have therapeutic implications for interruptions in therapy, particularly when participants are maximally suppressed. For instance, the prolonged half-life of the non-nucleoside reverse transcriptase inhibitors (NNRTIs) (efavirenz and nevirapine) could lead to unintentional monotherapy for a short period of time if all components of a potent NNRTI-containing regimen are discontinued at the same time. This may be even more significant in some intracellular reservoirs where the half-life of the NNRTIs may be even longer. The implications of these pharmacokinetic phenomena are unclear;

however, one small study that evaluated a 4-week interruption versus continuous therapy reported the emergence of resistance in five participants in the intermittent arm.[33] In SMART, among patients in the DC group taking an NNRTI and with an HIV RNA level ≤ 400 copies/mL at entry, those who interrupted all antiretroviral drugs simultaneously as compared to stopping ART drugs in a staggered manner or after replacing the NNRTI with another drug were less likely to achieve re-suppression when re-starting the NNRTI regimen. There was also a trend for more NNRTI mutations to develop when a simultaneous interruption strategy was used.[34]

4.1.12 Documentation of Reasons for Initiation or Change in ART

Whenever ART is first initiated, and every time ART is changed for participants in either group (including discontinuation and subsequent re-initiation), the relevant information must be reported on the appropriate case report form. The reason(s) for (re)initiation, change or discontinuation of ART, including treatment-limiting toxicities, will also be recorded on the case report form.

4.1.13 Use of Drugs for Prophylaxis for Opportunistic Diseases

Just as for ART, local clinical practice guidelines should be consulted regarding choice of agents for prophylaxis for opportunistic diseases, if and when such prophylaxis is indicated.

4.1.14 Provision of Antiretroviral Drugs at Study Completion

Once the active follow-up phase of START has been completed, ART will be obtained by prescription from the participant's clinician. It is anticipated that at the end of START a closeout period of approximately 6 months will be required to complete all data collection. During this time period, ART from the INSIGHT Central ART Repository may be used, and sites will be expected to transition all participants to other sources of ART. During this time period adverse events will continue to be reported. Stopping ART is not recommended.[1,6] All participating sites must have a plan for providing ART to participants at the end of the study.

4.2 CD4+ Cell Count and Viral Load Monitoring

CD4+ cell counts should be performed by laboratories that participate in proficiency testing programs.

Participants may be monitored more closely than data collection for this protocol requires. The frequency of non-study visits and measurements of CD4+ cell count and viral load will depend on the participant's clinical status and recent CD4+ cell count and viral load status. A high plasma HIV RNA level is associated with a more rapid drop in CD4+ count.[20] Thus, participants in the deferred group with high viral loads should be monitored more closely. ART should be initiated as recommended in section 3.7.1.

Participants randomized to the deferred arm whose CD4+ cell count drops to less than 350 cells/mm^3 should have a repeat CD4+ measurement within 4 weeks to confirm this

decrease. If the decrease is confirmed, the clinician should prescribe ART to be started by the participant as soon as possible within 4 weeks of confirmation of the decrease.

Following the initiation of ART (immediately following randomization for the early ART arm or later in follow-up for the deferred arm), local guidelines should be followed for monitoring CD4+ cell count and HIV RNA level. Most guidelines recommend that HIV RNA level be measured within 2-8 weeks after ART initiation. For participants on stable ART, HIV RNA levels should be determined every 3-4 months. For START, HIV RNA levels are required at least every 4 months.

4.3 Pregnancy and Breastfeeding

Women are not eligible for enrollment into the study during their pregnancy or while they are breastfeeding. However, they may be randomized after delivery if they are ART naive. World Health Organization (WHO) guidelines recommend that women with HIV infection avoid breastfeeding "when replacement feeding is acceptable, feasible, affordable, sustainable and safe." [35]

For women of child-bearing potential, a serum or urine pregnancy test must be performed before randomization and whenever pregnancy is suspected. For women to be prescribed efavirenz, a pregnancy test should be performed as close to the start of treatment as feasible and no more than 14 days beforehand.

Women in either treatment group who become pregnant during follow-up should be managed according to principles found in national[1,36,37] and international guidelines.[35, 33] This requires a careful assessment of the mother's HIV disease stage, antiretroviral experience, gestational age, and the risks and benefits of specific antiretroviral regimens or other interventions.

Pregnant women are still considered active study participants and will be counted in their assigned randomized group for primary analyses. After delivery and breastfeeding, women will continue their assigned treatment strategy. Women will be counseled concerning breastfeeding based on national and/or WHO guidelines. Outcomes of pregnancy will be assessed.

Study staff are encouraged to register pregnancies that occur on study prospectively with *The Antiretroviral Pregnancy Registry*. A link to this registry is available on the INSIGHT website (see Appendix H.)

4.4 HIV Transmission Counseling

All participants should receive counseling regarding prevention of HIV transmission to others as indicated.

4.5 Study Withdrawal

Participants may withdraw from the study at any time at their request and resume participation at any time upon re-consent. A participant may be withdrawn if:

- He/she relocates and data can no longer be collected.
- He/she is imprisoned, or involuntarily incarcerated for medical reasons. In this case, no data will be collected during the imprisonment or involuntary incarceration. However, once released from imprisonment or involuntary incarceration, an individual may resume participation in the group to which he or she was originally assigned upon re-consent.
- The study is discontinued.

All participants should otherwise be followed according to protocol. Even if a participant chooses not to adhere to the treatment assignment or data collection schedule, every effort should be made to follow participants for the primary composite endpoint until the end of the study.

4.6 Co-enrollment in Other Studies

There are no restrictions on co-enrollment in other studies as long as co-enrollment does not interfere with a participant's assigned treatment strategy in START.

5 Evaluation

5.1 Data Analysis

The primary analysis will be by intention to treat, comparing the early and deferred groups. Thus, all participants will be included in the primary treatment comparison, irrespective of whether they adhered to their assigned ART strategy. Time-to-event methods, including stratified log-rank tests, proportional hazards regression analysis, and Kaplan-Meier cumulative event curves, will be used to summarize the primary endpoint (i.e., time to the first event) and major secondary outcomes.[38] As part of these analyses, the proportionality of the hazards over time will be assessed. The primary analysis will be based on a Cox model with a single indicator for treatment group and with strata corresponding to geographic region (North America, South America, Europe, Australasia, and Africa).

Subgroup analyses for the primary endpoint and major secondary outcomes will be performed to determine whether the treatment effect (early versus deferred) differs qualitatively across various baseline-defined subgroups. Subgroup analyses will be performed by age, gender, race/ethnicity, geographic region, the presence of risk factors for serious non-AIDS conditions, baseline CD4+ cell count, baseline HIV RNA level, calendar date of enrollment in order to assess the effect of different treatment patterns that may emerge, and the ART regimen prespecified at the time of randomization. The latter subgroup analysis will permit evaluation of major outcomes for those who receive an NNRTI-based treatment as immediate ART versus deferral of ART (compared to those who would have been given an NNRTI-based regimen immediately had they been randomly assigned to immediate ART), and for those who receive a protease inhibitor (PI)-based regimen as immediate treatment versus deferral. The consistency of the treatment difference across participating countries will also be assessed. An overall test of heterogeneity will provide evidence of whether the magnitude of the treatment difference varies across baseline subgroups.

The goal of the study is to enroll participants at variable risk of serious non-AIDS diseases. Thus, the characteristics of participants enrolled will be closely monitored to ensure good representation of participants with major risk factors for serious non-AIDS conditions. Descriptive analyses will also be carried out summarizing ART regimens used by the early and deferred groups. These analyses will be carried out by geographic region and by calendar period of enrollment and follow-up. The initial ART regimen used will be compared to the regimen specified prior to randomization.

The CD4+ inclusion criteria for the study and the CD4+ count threshold for initiation of ART in the deferred strategy were defined to result in two treatment groups that differ substantially in terms of ART exposure, and consequently, HIV RNA level and CD4+ cell count. As part of the ongoing monitoring of this study, time to initiation of ART in the deferred group will be monitored and HIV RNA levels and CD4+ cell counts will be compared for the early and deferred groups. Initiations of ART in the deferred group that are not in keeping with the protocol will be closely monitored. Likewise,

discontinuation of ART in the early and deferred groups will be closely monitored. Kaplan-Meier life-table methods will be used to estimate the cumulative percent of participants in the deferred group who initiate therapy after different periods of follow-up. Follow-up levels of HIV RNA and CD4+ count will be summarized in a number of ways. Follow-up time spent in various categories will be compared. Longitudinal measurements of viral load and CD4+ cell count will be summarized using measured levels (or log transformed) and using repeated binary assessments (e.g., viral load below 50 copies/mL).

In addition, within each treatment group a number of analyses aimed at predicting changes in CD4+ cell count, viral load, and clinical responses will be carried out. These will include analyses of predictors of HIV RNA suppression and CD4+ cell count increases following initiation of ART.

Analyses aimed at understanding whether differences in CD4+ cell count and HIV RNA levels explain differences in the primary endpoint and its components will be carried out. These models will employ Poisson regression models and Cox models with time-varying covariates. Account will be taken of the measurement error associated with CD4+ count and HIV RNA that could attenuate associations with risk of the primary endpoint.

5.2 Data Monitoring

The trial will be conducted under the direction of the INSIGHT Executive Committee and the START study protocol team. Members of the Executive Committee and protocol team are given in Appendix D. The Executive Committee, the protocol team (except those who prepare the confidential analyses), and all participating investigators will be blinded to interim results on clinical outcomes.

An independent DSMB, supported by NIAID, will meet as often as required, but at least annually, to review the general conduct of the trial and to review interim analyses of the major clinical outcomes. The monitoring plan for the review of the primary endpoint and its two major components is described below.

5.2.1 Monitoring of Pilot Phase of the Study

During the pilot phase of the study, the primary focus of DSMB reviews will be enrollment. At each review, the DSMB will be asked to assess whether enrollment to the trial is on course. This assessment will consider time requirements for protocol registration and other factors that may slow initiation of enrollment at some sites. The DSMB will also review follow-up visit attendance and treatment adherence.

The goal of the pilot phase of the study is to enroll at least 900 participants by 70 sites supported by DAIDS. If this goal is not achieved, power will be recalculated after the first year with the estimated reduced sample size considering the planned enrollment period (3 years) and follow-up (at least 3 years after the last participant is enrolled) for the definitive study, the actual enrollment rate during the pilot phase, including enrollment by sites supported from outside DAIDS, and the time it takes to open

additional sites. If a projected enrollment sufficient to provide power of at least 70% is not thought to be achievable within a reasonable period of recruitment (currently considered to be 3 years), then consideration will be given to stopping the study.

5.2.2 Monitoring of Second Phase of the Study (Definitive Study)

If a joint decision is made by the University of Minnesota and DAIDS to support the definitive phase of the trial, additional clinical sites, including sites not supported by DAIDS, will be invited to participate along with sites that enrolled participants during the pilot phase of the study. Follow-up of all participants enrolled during the pilot phase will continue. A sample size re-estimation will be carried out by the protocol team before the target enrollment of 4,000 participants is achieved to ensure that the planned sample size is adequate. The DSMB will be asked to review the plan for the definitive study based on the sample size re-estimation. For the sample size re-estimation, the protocol team will use only pooled endpoint data, i.e., the number and rate of AIDS* and non-AIDS events for both treatment groups combined. The protocol team will use these pooled event data and other relevant data sources to estimate the rates of AIDS*, non-AIDS, and deaths due to other causes (including unknown causes).

The DSMB will be asked to recommend early termination or modification only when there is clear and substantial evidence of a treatment difference. As a guideline, the Lan-DeMets spending function analog of the O'Brien-Fleming boundaries will be used to monitor the primary endpoint comparison.[39,40] The DSMB will be asked not to stop the study early unless there is evidence of a significant treatment difference based on the spending function boundary for the primary endpoint and each of the two major components of the primary endpoint – AIDS*, and non-AIDS or deaths not attributed to AIDS – are consistent (in the same direction, for example, $Z > 1.5$ for each outcome).

The DSMB will also review other relevant data that might impact the design of START, e.g., data from other completed trials, and cohorts with similarly defined target populations.

At each DSMB review, beginning with the review prior to the end of enrollment when sample size is re-estimated, futility analyses will be presented to the DSMB by the unblinded statisticians based on conditional and unconditional power. *Conditional* power incorporates the observed results by treatment group thus far (and uses the originally assumed treatment effect for future data) to calculate the conditional probability of obtaining a significant result by the end of the trial. In contrast, *unconditional* power does not take into consideration the observed treatment difference. It uses a revised estimate of the event rate in the deferred ART arm based on the observed data, the planned duration of follow-up, and the originally assumed treatment effect to calculate what the real power was at the beginning of the trial. Participants will be followed to a common closing date a minimum of 3 years after the last participant is enrolled. Thus, participant follow-up will range from 3 to 6 years, with an average follow-up of approximately 4.5 years.

Conditional and unconditional power estimates are used for two different purposes. Conditional power tells us whether we are likely to get a significant result, whereas unconditional power tells whether a null result would still be meaningful. For example, suppose the unconditional power were only 40%. Even if the true treatment benefit were as originally hypothesized, there would be a 60% chance of missing it. Therefore, a null result would not rule out the originally hypothesized treatment benefit. On the other hand, if unconditional power were high — say 90% — then a null result would effectively rule out the originally hypothesized treatment benefit.

As a guideline, we recommend that the DSMB first consider unconditional power. If unconditional power is less than 70%, the DSMB should then consider conditional power. If conditional power, given the observed data and assuming the originally hypothesized treatment effect thereafter, is less than 20%, consideration should be given to stopping the trial. We recommend early termination for futility only if both conditional and unconditional power estimates are low, i.e., only if a null result is both likely and not meaningful. It is possible that unconditional power is low in the presence of a very large treatment effect of early ART. Hence, there is a need to also consider conditional power. Such a scenario would not be grounds for stopping for futility because conditional power would probably still be high, indicating that a null result is unlikely.

In summary, the DSMB for START will be provided with the aforementioned guidelines but be expected to use their expert and independent judgment concerning early termination. It is recognized that there are a number of considerations in determining whether a trial should be stopped early. For that reason, we propose guidelines to the DSMB, not rules. This line of thinking was summed up well by Canner in his reflections on the Coronary Drug Project: "…no single statistical decision rule or procedure can take the place of the well-reasoned consideration of all aspects of the data by a group of concerned, competent, and experienced persons with a wide range of scientific backgrounds and points of view".[41]

6 Protection of Human Subjects & Other Ethical Considerations

6.1 Local Review of Protocol and Informed Consent

Prior to the initiation of the study at each clinical research site, the protocol, all informed consent forms and the participant Information materials will be submitted to and approved by the site's IRB or IEC. Likewise, any future amendments to the study protocol will be submitted and approved by each site's IRB or IEC. After IRB/IEC approval, sites must register for START before screening potential participants, and must register for any protocol amendments. Protocol registration procedures are described in the START *Protocol Instructions Manual*.

6.2 Ethical Conduct of the Study

The study will be conducted according to the Declaration of Helsinki in its current version (2004); the requirements of Good Clinical Practice (GCP) as defined in Guidelines, EU Clinical Trials Directive (2001/20/EC), and EU GCP Directive (2005/28/EC); Human Subject Protection and Data Protection Acts; the US Office for Human Research Protections (OHRP); or with the local law and regulation, whichever affords greater protection of human subjects.

6.3 Informed Consent of Study Participants

All study participants must sign all applicable approved informed consent forms (see samples in Appendix A) prior to any study-related procedures.

6.4 Confidentiality of Study Participants

The confidentiality of all study participants will be protected in accordance with GCP Guidelines and national regulations.

7 Other Important Documents and Policies

7.1 Reference Documents

Study procedures are described in detail in the *START Protocol Instructions Manual.*

7.2 Data Collection and Monitoring

Study data will be collected on standardized case report forms. It is assumed that most data will be collected during participant visits to health-care providers. In some instances, it may be necessary to obtain and abstract hospital records. Written permission from the participant is generally required. Study data and case report forms will be made available to site monitoring personnel. Monitoring may be performed by staff from the INSIGHT International Coordinating Centers or Site Coordinating Centers or by contractors of the primary funder.

At a minimum, all items referenced in the protocol as being relevant to the research study will be recorded in the participant's research record in accordance with standard procedures. In addition, all items specifically required by the protocol will be recorded on case report forms. Items that are recommended but not required by the protocol may or may not be recorded on case report forms.

7.3 Publications and Presentations

Publications and presentations related to data obtained from this study will adhere to the INSIGHT Publications and Presentations Policy on the INSIGHT website (see Appendix H).

8 Substudies

This section describes three substudies. Not all sites will be participating in these substudies. At the sites conducting the Genomics and/or Neurology substudies, participant participation in either substudy is optional and requires a separate consent. At sites conducting the Informed Consent substudy, all participants are part of the substudy by virtue of going through the informed consent process at that site. Participants are informed that, by signing the consent form for START, they are also participating in this substudy.

8.1 Genomics Substudy

8.1.1 Background and Rationale

Despite progress in the treatment of HIV/AIDS and greater understanding of the pathophysiology of HIV infection, clinicians and researchers continue to observe unexplained differences among individuals in both the progression of untreated HIV infection and response to ART. Some of these differences can be attributed to individual participant behavior (e.g., adherence to therapy) and/or diversity of the infecting virus (e.g., viral replicative capacity), but a significant portion is likely related to individual (host) differences that are determined by individual genetics.

One example of this is the 32 base-pair deletion in the CCR5 gene that in a homozygous state confers protection against HIV infection.[42,43,44] Another example is the association of human leukocyte antigen, HLA B*5701, with abacavir hypersensitivity reaction.[45] While these findings require further understanding before routine clinical application, the first example shows how knowledge of host genetics may lead to a targeted treatment strategy based on a subject's individual risk of disease progression, and the second example shows how knowledge of genetics can lead to targeted therapy to maximize efficacy and minimize toxicity.

These examples illustrate the promise of genetic knowledge in the management of HIV/AIDS, but the next step is to refine current genetic associations while continuing to discover new associations. In order to utilize current and future advances in genetic technology to better understand the role of host genetics in the pathophysiology and treatment of HIV infection, there is a need to link genetic data with clinical outcomes data in a large, well-characterized, HIV-infected population.

8.1.2 Purpose

The purpose of this study is to obtain a whole blood sample from which DNA will be extracted to study validated (present and future) genetic variants that determine the risk of the various primary and secondary outcomes assessed in START. Known genetic variants include for example HLA/killer cell immunoglobulin-like receptors (KIRs), genomic factors controlling viral replication, and genetic associates of dyslipidemia. Also, the material may be used to assess validated (present and future) genetic variants associated with tolerability of all the various medical interventions used in the study. Finally, the material will be used, via genomewide analysis, for appropriate control for population structures in this trial. These analyses may be done in participants included in START specifically or may be merged with similar studies from other sources.

8.1.3 Study Design

This nonrandomized multicenter protocol is designed to obtain a whole blood sample from participants who are planning to participate in START. The specimen will be archived for currently unspecified human genetic and other related analyses.

8.1.4 Sample Size and Statistical Considerations

All of the participants in START may participate in this substudy, so the potential sample size is the same as that for the main protocol.

8.1.5 Participant Selection

Enrollment is open to all participants in START. Consent to participation in START is required to provide a source of follow-up data to use in conjunction with the human genetic or other related assays in investigating questions of interest.

Inclusion Criteria

- Consent to randomization in START.
- Signed informed consent for the substudy (see Appendix A4).

Exclusion Criteria
None.

8.1.6 Enrollment and Data Collection

Following consent to the START study, a participant will be asked to sign the START Genomics substudy informed consent. It is strongly encouraged that participants consent to the Genomics substudy and provide a blood sample prior to randomization in START. It is, however, acceptable for participants to enroll in the Genomics substudy any time after consent and randomization in the main study.

Participants who consent to the START Genomics substudy will have the following collected:

- Six (6) mL of whole blood, frozen and shipped as directed in the *START Genomics Substudy Protocol Instructions Manual.*

Participation in the START Genomics substudy is considered complete when the START Genomics substudy enrollment form is received at the INSIGHT Statistical and Data Management Center (SDMC). This form will collect the official date of enrollment, which is the date the blood is drawn.

8.1.7 Follow-up

There are no follow-up requirements for this substudy.

8.1.8 Specimen Collection

Sites will ship frozen whole blood specimens to a central specimen repository designated in the *START Genomics Substudy Protocol Instructions Manual* for long-term storage. At a future point, DNA will be extracted from either a subset of specimens or all specimens. Additional future plans might include separation of plasma on samples for either a subset of participants or all participants.

8.1.9 Human Subjects

This protocol must receive the approval of the participating site's IRB or IEC prior to implementation. All participants must sign an informed consent form (see sample in Appendix A6).

Participants who wish to withdraw consent for the storage and use of specimens collected in this protocol may do so at any time by contacting a member of the research staff and expressing their intention to withdraw consent in writing. If a participant withdraws consent, the specimen and any products derived from the specimen will not be used in further analysis and every effort will be made to destroy them. However, results of laboratory tests that have been performed prior to the withdrawal of consent will continue to be available for future analyses and will not be destroyed. A participant who has withdrawn consent and wishes to reconsider may reenroll in the protocol by repeating the informed consent and enrollment process.

In some situations, it may be important for a participant who has already provided one blood sample under this protocol to provide an additional sample (for example, in the event that specimen transport problems or labeling errors render the original specimen unacceptable). If a new specimen must be drawn, participants must repeat the informed consent and enrollment process.

8.1.10 Confidentiality

The confidentiality of all study participants will be protected in accordance with standard IRB/IEC policies and procedures in addition to those procedures specified in this protocol document.

The privacy of individual participants will be protected by the data management system described in the *START Genomics Substudy Protocol Instructions Manual.* After approval of a concept or study requiring use of genetic material, whole blood collected under this protocol will be processed to yield host DNA or other products, hereafter referred to as human biological material (HBM). Processed HBM will be labeled with a generated identification number (GID) assigned by the INSIGHT SDMC. This GID will be used in place of the START participant identification number (PID) in all data transactions concerning individual results of tests on the processed HBM. Thus, results of genetic tests on individual participants cannot be connected to a particular participant without access to both the participant's GID and the participant's PID. Data linking the PID to the GID will be stored only at the SDMC in an encrypted file. Data security will be maintained by minimizing the number of individuals at the SDMC with access to both identifiers.

Results of laboratory tests performed on HBM obtained in connection with this protocol will be stored in databases at the SDMC, indexed by the GID. These databases will not contain the participant PID or any other clinical data. The table for linking PIDs with GIDs will be accessible to a minimal number of individuals at the SDMC.

Further details of the data management practices intended to safeguard individual participant genetic information are given in the *START Genomics Substudy Protocol Instructions Manual.*

The following paragraph concerning the Certificate of Confidentiality applies only to sites located within the United States:

In addition, a Certificate of Confidentiality has been obtained for START to help protect the privacy of participants by withholding their names and other identifying characteristics, including processed DNA and results of genetic testing, from all persons not connected with the conduct of research.

8.1.11 Access to Stored Specimens Collected for the START Genomics Substudy

Stored specimens, including HBM processed from the donated whole blood specimens, will not be sold to third parties.

Tests using stored samples that are consistent with the purpose of this substudy may be proposed by any START investigator who follows established INSIGHT policies and procedures. Before testing is initiated, the proposal must be approved by the INSIGHT Executive Committee and the IRB governing the SDMC.

Whether in the form of whole blood or blood products (e.g., extracted host DNA), the stored HBM will be used for studies relevant to the health of persons with HIV infection, including its epidemiology, diagnosis, pathogenesis, complications, treatment and prognosis. As previously stated, tests will include validated (present and future) genetic variants that determine the risk of the various primary and secondary outcomes assessed in START. Known genetic variants include, for example, HLA/KIR, genomic factors controlling viral replication, and genetic associates of dyslipidemia. Also, the material may be used to assess validated (present and future) genetic variants associated with tolerability of the various medical interventions used in the study. Finally, the material will be used, via genomewide analysis, for appropriate control for population structures in this trial. These analyses may be done in participants included in START specifically or may be merged with similar studies from other sources.

8.1.12 Access to Individual Test Results

Due to the experimental nature of the assays involved and the inherent difficulty of interpreting their clinical significance in individual cases, individual test results will not be provided to participants, investigators, clinical site research staff or health-care providers. Summaries of clinically relevant findings from genetic tests will be disseminated to all study participants through individual clinical units and the INSIGHT website. In extraordinary circumstances when knowledge generated from genetic tests on HBM may have profound and unequivocal implications for the health of study participants, every reasonable effort will be made to offer study participants the genetic test repeated outside of the study. However, original study results cannot be provided to participants or clinicians under any circumstances.

Data generated from HBM for START will not be made available to employers, insurance companies, or other third parties that are not directly involved in the clinical research.

8.2 Neurology Substudy

8.2.1 Background and Rationale

The impact of HIV infection upon neurocognitive function in participants who have early HIV disease with high CD4+ cell counts is notable for the paucity of studies that directly address this issue, and more particularly the issue of whether ART during this period can prevent or reverse the development or progression of neurocognitive impairment during this phase of HIV infection. However, the conflation of data from studies of primary HIV infection (PHI) and studies of neurocognitive performance, cerebrospinal fluid (CSF) and histopathological findings in patients with early HIV disease indirectly provide important evidence that HIV infection involves the brain early in the course of the disease, establishes a state of chronic central nervous system (CNS) immunoactivation, and may be detrimental to brain integrity. The following discussion is largely confined to those studies that include HIV-infected patients with early disease and CD4+ cell counts of 350 cells/mm^3 or greater.

PHI may be associated with a number of different neurological presentations including meningoencephalitis, meningitis, seizures and cranial neuropathies.[46,47,48] During PHI, HIV is detected in the CSF [49] and the CSF HIV viral load is highest in those patients with neurological symptoms.[49] An important marker that suggests that early damage occurs within the CNS during PHI is neurofilament protein (NFL), which is elevated in some patients during PHI.[50] Similarly, CSF levels of quinolinic acid, an endogenous neurotoxin, are elevated in asymptomatic disease.[51]

Following PHI, HIV remains detectable in the CSF throughout the course of the disease in most patients[52] including those with CD4+ cells > 500 cells/mm.[48,53,54] The source of ongoing HIV replication within the CNS may result from infected CD4+ T cells trafficking through the brain and/or from productive infection of perivascular and meningeal macrophages, given the respective appellations “transitory” and “autonomous” CNS infection by Spudich et al. The distinction between the two proposed mechanisms is important as transitory CNS infection appears to occur in early disease with associated rapid CSF decay kinetics following treatment with ART, similar to those seen in plasma. Patients with more advanced HIV disease are likelier to have more autonomous CNS infection with slower associated decay kinetics following ART.[54,55]

There is evidence to show that the CNS adaptive immune response is integral to the control of HIV infection in the brain following PHI: data support that important contributions are made by both humoral[56]and cellular immunity.[57,58,59,60] McCrossan et al recently proposed that control is conferred largely by cellular immunity principally involving CD8+ cells. In their histopathological study of patients with early HIV disease (including patients with CD4+ cell counts as high as 824 cells/mm^3) they reported that there was a significant correlation between the proportion of CD8+ cells and the HIV proviral load in brain tissues suggesting that the level of immune surveillance increases as the level of HIV proviral DNA increases. McCrossan further hypothesized that with

progressive *peripheral* immunosuppression the integrity of the CNS surveillance afforded by cellular immunity wanes, and hence the brain is vulnerable to upregulated local CNS infection and increasing numbers of infected monocyte/macrophages trafficking into the CNS.[60] This hypothesis is supported by a number of studies wherein patients do achieve improved neurocognitive function despite receiving highly active antiretroviral therapy (HAART) regimens that do not penetrate the CSF well.[61,62,63,64,65,66] A commonality among some of these studies is that following commencement of ART, neurocognitive performance improved in association with significant decreases in plasma HIV viral load[62,65] or a significant decrease in both HIV viral load and an increase in CD4+ cell counts.[63]

Predictors of clinical neurocognitive disease progression in early HIV disease have been described in one longitudinal study from the pre-HAART era of 76 patients who seroconverted a median of 11 months (range, 1 month to 4 years) prior to study entry.[67] Within 5 years of estimated seroconversion, approximately 10% of those patients whose study baseline CD4+ cell counts were > 400 cells/mm^3 had developed HIV-related neurocognitive impairment (NCI). Conversely, 40% of those patients whose CD4+ cell counts were < 400 CD4 cells/mm^3 counts at study baseline were neurocognitively impaired over the same time period. Plasma viral loads greater than 30,000 copies/mL also independently predicted more rapid neurocognitive decline. The few patients treated with two drugs were significantly less likely to become impaired.[67]

Accurate estimates of the prevalence of impairment of neurocognitive function in otherwise healthy untreated patients with ≥ 500 CD4+ cell counts/ mm^3 are not available. Notably, however, studies of patients who were stratified according to HIV disease stage, rather than by CD4+ cell count, show that asymptomatic patients *per se* do experience mild neurocognitive deficits, [68,69,70] compared to HIV negative controls. Nonetheless, even mild neurocognitive impairment may be associated with diminished functioning in day-to-day life and an increased risk of unemployment.[71]

In summary, these findings demonstrate that the CNS is involved very early in the course of HIV infection. Control of CNS infection appears largely predicated upon intact peripheral immunity, and the decline of peripheral immunity would be delayed by early ART. Patients with early disease and high CD4+ cell counts may have mild neurocognitive impairment that, in turn, may affect aspects of their everyday life including vocational capacity. Predictors of neurocognitive decline in early HIV disease are a high plasma viral load and CD4+ cell counts < 400 cells/mm^3. The anticipated fall in HIV viral load and concomitant rise in CD4+ cell counts afforded by early ART would theoretically improve neurocognitive performance in this early disease patient group or, at the least, prevent neurocognitive decline.

Purpose

The purpose of the study is to determine whether immediate initiation of ART in ART-naïve persons with a CD4+ count > 500 cells/mm^3 is superior with respect to neurocognitive function to deferring ART initiation until CD4+ counts decline to below 350 cells/mm^3.

Study Hypothesis
We hypothesize that early ART results in improved neurocognitive function compared to deferred ART.

8.2.2 Methodology

8.2.2.1 Study Design

This is a substudy of START. It is planned to co-enroll a total of 600 participants over 3 years at selected, geographically diverse sites. Randomization (1:1 ratio) to the early or deferred treatment groups will be determined by the START study. Participants will be followed to the common closing date of START. This is estimated to be 6 years after the beginning of enrollment (3 years of enrollment and a minimum of 3 years follow-up for each participant). Before enrollment ends, sample size of the main START study will be re-assessed and sample size recalculated. At the same time, sample size for the Neurology substudy will be re-calculated.

START Neurology Substudy Schematic

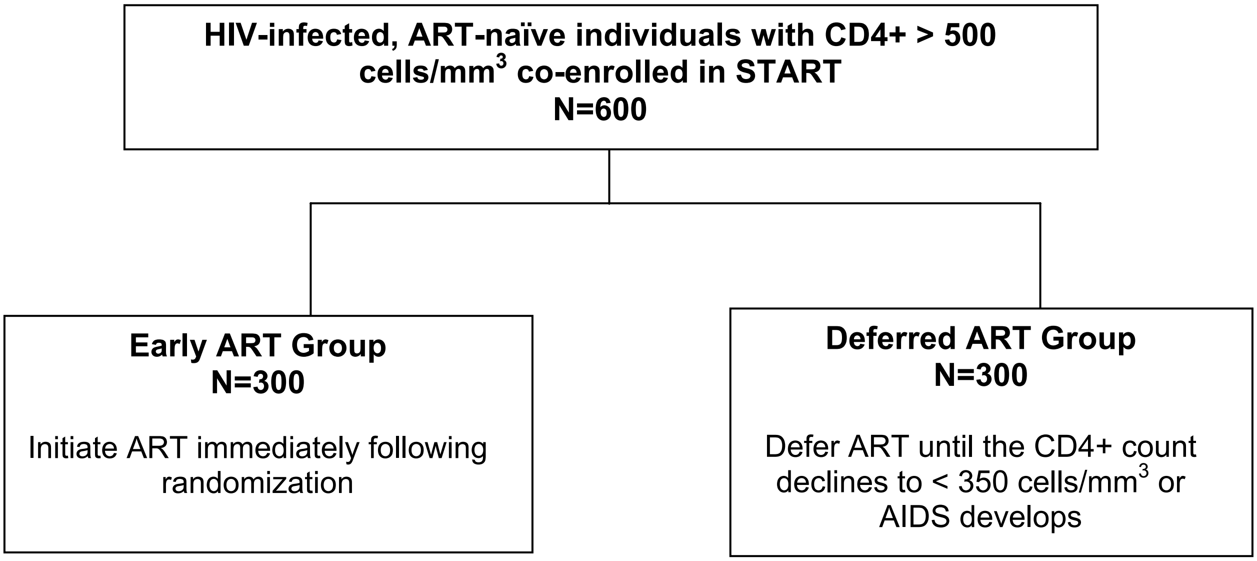

Data collection: Baseline, months 4, 8, 12, and annually thereafter.

8.2.2.2 Data Collection

To be collected at baseline (within 60 days prior to randomization), months 4, 8, and 12, and annually thereafter:

a. The quantitative neurocognitive performance (QNPZ-8) score, derived from a test battery consisting of
   - Grooved Pegboard test
   - Color Trails 1 test

- Color Trails 2 test
- WAIS-III Digit Symbol test
- Finger Tapping test
- Hopkins Verbal Learning test – revised (HVLT-R), Learning and Delayed Recall
- Semantic Verbal Fluency test (category fluency)

b. Center for Epidemiologic Studies Depression Scale (CES-D), a tool to screen for depression.

Additionally, to be collected at baseline:

- Extended demographics, including years of education, occupation, employment status, income, and area of residence (urban or rural)

8.2.2.3 Study Objectives

Primary Objective

To compare the early group with the deferred group for changes in neurocognitive function, as measured by the QNPZ-8 score.

Major Secondary Objectives

a. To compare the early and deferred ART groups for:
   - Changes in QNPZ-8 scores through the first year
   - Changes in neurocognitive function, as measured by each of the neuropsychological tests in the QNPZ-8 test battery, through the first year and overall
   - Proportions of participants with neurocognitive impairment
   - Changes in neurocognitive deficits as measured by the average deficit score (ADS)
   - Proportions of participants with depression as measured by the CES-D, and for changes in CES-D scores

b. To compare the early and deferred ART groups for changes in neurocognitive function in subgroups of participants defined by demographics (age, gender, education), geographic location, CD4+ cell count, HIV RNA level, estimated level of CNS penetration of the planned baseline ART regimen, drug classes in the planned baseline ART regimen, level of neurocognitive function at baseline, and other baseline characteristics, and to assess homogeneity of the treatment difference with respect to these baseline characteristics.

c. To determine the association of changes in neurocognitive function with baseline characteristics, including demographics (age, gender, education, occupation, employment status, income, urban or rural area of residence), geographic location, CD4+ cell count, HIV RNA level, perceived general health, estimated level of CNS penetration of the planned baseline ART regimen, drug classes in the planned

baseline ART regimen, level of neurocognitive function at baseline, dominant versus non-dominant hand, and others.

d. To determine whether changes in neurocognitive function are associated with CD4+ and HIV RNA levels through follow-up, perceived general health, duration of exposure to certain drug classes, estimated level of CNS penetration of the ART regimen, and other time-varying factors.

e. To study the association of factors measured at baseline with neurocognitive function at baseline. These factors include demographics, geographic location, CD4+ cell count, HIV RNA level, health history, perceived general health, and others.

f. To describe associations between the neuropsychological tests at baseline, and associations between the tests when measuring change in neurocognitive functioning through follow-up.

8.2.2.4 Study Endpoints and Outcome Measures

Neuropsychological Test Battery
The neuropsychological quantitative performance test battery to be used is comprised of eight neuropsychological tests that assess neurocognitive functioning across a range of cognitive abilities, shown in Table 1. Tests are described in more detail below.

Table 1. Neuropsychological Tests

Domain	Test
Attention/speed of information processing	Color Trails 1
Executive function	Color Trails 2
Verbal learning	HVLT-R, Learning
Verbal memory	HVLT-R, Delayed Recall
Language fluency	Semantic Verbal Fluency
Fine motor skills/ complex perceptual	Grooved Pegboard
Motor speed	Finger Tapping
Speed of information processing	WAIS-III Digit Symbol

Grooved Pegboard Test (Both Hands)[72]
The purpose of the test is to assess psychomotor speed and coordination. The Grooved Pegboard is a manipulative dexterity test that consists of 25 holes with randomly positioned slots in a 5 x 5 matrix. Pegs, which have a key along one side, must be rotated to match the hole before they can be inserted. This test requires more complex visual-motor coordination than most pegboards.
Score: Time to completion in seconds.

Color Trails Test: Color Trails 1 and Color Trails 2[73]
These are tests of speed for attention, sequencing, mental flexibility, and visual search and motor function. The tests are paper and pencil based. Color Trails 1 requires the participant to rapidly connect encircled numbers scattered on a page in sequence from 1 to 25. In Color Trails 2, each number is presented twice, once with a pink background and once with a yellow background, and on this part the participant rapidly connects encircled numbers in sequence, but alternates between pink and yellow colors.
Score: Time to completion in seconds, for each part.

WAIS-III Digit Symbol Test[74]
This is a test of psychomotor speed, concentration, and graphomotor abilities that requires the respondent to match symbols to numbers as quickly as possible, using a visual reference.
Score: Number correct in 120 seconds.

Hopkins Verbal Learning Test – Revised (HVLT-R): Learning and Delayed Recall [75]
The HVLT-R provides information on the ability to learn and immediately recall verbal information across trials, as well as the ability to recall this information after a delay. In the learning phase, a list of 12 words (four words from each of three semantic categories) is presented to the participant over three trials. In each trial, the same list is presented, and the participant is asked to recall as many items as possible from the list in any desired order. A 20-minute delay follows the administration of the three trials, after which the subject is asked to recall the list (delayed recall phase). In order to minimize practice effects that may result from repeated administrations, six alternate forms of the test are available.
Score, Total Learning: Number correct across the three learning trials (sum).
Score, Delayed Recall: Number of words correctly recalled after the 20 minute delay.

Finger Tapping Test (Both Hands)[76]
This is a test of motor speed. Using a specially adapted tapper, the participant is instructed to tap as rapidly as possible using the index finger. The number of taps is counted in five consecutive 10-second trials.
Score: Average number of taps across five trials excluding highest and lowest.

Semantic Verbal Fluency Test[77]
This test assesses speed of processing, memory, initiation, and inhibition, without requiring motor skills. It is a timed task requiring the production of words in a specific category in the subject's native language. Subjects are given 60 seconds to produce as many words as possible within a specific category (e.g. Animals).
Primary score: Number of correct words within 60 seconds.

Composite Outcome Measures

QNPZ-8 Score
The QNPZ-8 test battery consists of the eight neuropsychological tests described above. First, test scores will be standardized to *z-scores*, by subtracting the mean and

dividing by the standard deviation of test scores in reference populations. Best available normative data will be used, matched for each participant by age, education, sex, and race/ethnicity, when appropriate, and as available for each test. For the Finger Tapping and Grooved Pegboard tests, the average of the z-scores for the dominant and non-dominant hands will be used in calculating the QNPZ-8 score. For the HVLT-R, the z-scores for Learning and for Delayed Recall will be entered separately when calculating the QNPZ-8 score. The participant's eight individual z-scores will then be averaged to produce a quantitative neurocognitive performance z-score (QNPZ-8 score).

QNPZ-8 scores above zero denote above-average neurocognitive function and scores below zero denote below-average neurocognitive function compared to the reference population.

Neurocognitive Impairment
For the purpose of this study, neurocognitive impairment is defined as:

a. decrease in the QNPZ-8 score from the participant's baseline by at least 0.5,
   or
b. decrease in z-scores in two or more independent tests to at least 1 below baseline. The two tests can not be both Grooved Pegboard and Finger Tapping, since these tests both measure fine motor skills.

ADS

For each of the eight neuropsychological tests, the deficit score is calculated as follows:

Table 2. Calculation of Deficit Scores Based on Z-Scores

Deficit Score	Z-Score
0 (normal)	$\geq$ -1
1 (mild)	-1.5 to less than -1
2 (mild-moderate)	-2 to less than -1.5
3 (moderate)	-2.5 to less than -2
4 (moderate-severe)	-3 to less than -2.5
5 (severe)	< -3

The ADS is the average of the deficit scores for the eight neuropsychological tests. Higher scores denote more impairment. Assuming that the reference populations standardize the z-scores correctly to mean 0 and standard deviation 1, then participants who score more than 1 standard deviation below the population average on two of the eight tests would have an ADS of at least 2/8. In calculating the ADS, low performance on some tests is not cancelled out by high performance on other tests, as could be the case with the QNPZ-8.

CES-D

The CES-D[78] is a screening tool for depression. It is a 20-item pen and paper questionnaire designed to assess the frequency and severity with which symptoms of

depression are experienced. Questions refer to the past week. Participants rate each item on a 4-point scale. The CES-D score is obtained by summing the scores for the 20 items (scores for items 4, 8, 12, and 16 are reversed). CES-D scores range from 0 to 60. Scores of 16 to 26 are considered indicative of mild depression and scores of 27 or more indicative of major depression. The CES-D has been widely used in research and has been translated into a number of different languages.

CNS Penetration

The level of CNS penetration for a given ART regimen will be calculated as the CNS penetration effectiveness score (CPE) by Letendre et al.[79] In this algorithm, CNS penetration scores are assigned to each antiretroviral drug and the CPE score for a regimen is calculated as the sum of scores for the individual drugs. If newer algorithms are published, the best available will be used.

General Health

The participants' perception of their general health will be assessed by two items on a questionnaire, on a 5-point Likert scale and on a visual analog scale. These data are part of the QOL assessments in the main START study, and will be collected at baseline, month 4, and annually.

Primary Endpoint

Change in QNPZ-8 score

Secondary Endpoints

- Change in test scores for each of the eight neuropsychological tests – Grooved Pegboard, Color Trails 1 and 2, Finger Tapping, HVLT-R Learning, HVLT-R Delayed Recall, Semantic Verbal Fluency, and WAIS-III Digit Symbol
- Change in ADS
- Proportion of participants with neurocognitive impairment

8.2.2.5 Sample Size Calculations

A total sample size of 600 is considered sufficient to detect an average treatment difference of 0.13 in mean change in the QNPZ-8 scores from baseline, where the treatment difference is averaged over the 4, 8, and 12 month visits in the first year and annual visits thereafter, with a power of 80% at a 5% significance level. The difference of 0.13 in mean change in the QNPZ-8 scores would not be clinically meaningful for an individual participant but would be clinically meaningful for a participant group. Table 3 displays the sample sizes necessary under several scenarios. The sample size of 600 assumes a standard deviation of 0.7 for change in QNPZ-8 scores from baseline to any follow-up visit and is inflated by 10% to account for missed visits and loss to follow-up. With this sample size, a treatment difference of 0.1 would be detected with 58% power.

The sample size calculations were performed for a balanced design, assuming that the QNPZ-8 scores follow a longitudinal mixed model with exchangeable within-participant correlation structure and normally distributed random effects. The correlation structure implies that the between-participant standard deviations of change in QNPZ-8 scores from baseline to a given follow-up visit are equal through follow-up within each treatment group. For simplicity, the SD in Table 3 is expressed as standard deviation of change from baseline to any follow-up visit. The model used for the sample size calculations assumes that every participant completes the month-48 visit, as an approximation for the projected mean follow-up of 4.5 years in the main START study.

Table 3. Total Sample Size for Detecting Average Treatment Differences Between the Early and Deferred Groups in Mean Change in QNPZ-8 Scores from Baseline, Averaged over Months 4, 8, 12, 24, 36, and 48; power 80 %, significance level 5%

Difference Early vs. Deferred	Total N (N inflated by 10%)			
	SD=0.5	SD=0.6	SD = 0.65	SD = 0.7
Δ = 0.10	474 (521)	682 (750)	798 (878)	926(1019)
Δ = 0.13	282 (310)	404 (444)	474 (521)	**550 (605)**
Δ = 0.15	212 (233)	304 (334)	356 (392)	414 (455)

The assumption of SD=0.7 for the standard deviation of the *change in QNPZ-8 scores* from baseline was based on the following considerations.

a. In the SMART study, QNPZ-5 scores were obtained at baseline and month 6 for 258 participants. The QNPZ-5 scores were based on four of the QNPZ-8 tests, excluding the HVLT-R, Semantic Verbal Fluency, and the WAIS-III Digit Symbol tests, plus the Timed Gait test. The standard deviation of change in QNPZ-5 scores (pooled across the two treatment groups) was 0.52, with a 95% confidence interval of 0.48 – 0.57. The value of SD=0.7 was chosen conservatively to guard against model misspecification, including misspecification of the within-participant correlation structure. Also, the somewhat different composition of the neuropsychological test battery compared to the QNPZ-5 may result in a different, possibly higher SD of change for the QNPZ-8.

b. In a different study, four neuropsychological tests were administered at baseline and month 12, including the Grooved Pegboard, Finger Tapping, Timed Gait, and the Digit Symbol Modalities tests. QNPZ-4 scores were calculated from the standardized test results. The standard deviation of changes in QNPZ-4 scores from baseline to month 12 was 0.7, 95% confidence interval of 0.6 – 0.9 in 108 HIV-infected patients (Richard Price, personal communication).

The protocol team will monitor the standard deviation of change in QNPZ-8 scores and the within-participant correlation structure, pooled across treatment groups. Sample size will be recalculated concurrently with the sample size recalculation for the main START study.

8.2.2.6 Sample Size, Enrollment and Site Selection

Enrollment and Site Selection

Enrollment into the Neurology substudy is planned to start concurrently with the main START study. The Neurology substudy aims to co-enroll participants from a limited number of sites and to co-enroll a high proportion of eligible participants at these sites. Limiting the number of participating sites makes it easier to standardize the administration of the neuropsychological tests and also is more cost effective.

It is planned to assess feasibility and re-calculate sample size for the Neurology substudy concurrently with the main START study, taking into account the observed standard deviation of change in QNPZ-8 scores and the expected duration of the main study.

8.2.2.7 Participant Selection

A total of 600 participants will be enrolled at selected sites that are geographically diverse and ensure a demographically diverse population. At these sites, it is planned to co-enroll a high proportion of the eligible START study participants.

Inclusion Criteria
- Simultaneous co-enrollment in the START study
- Signed informed consent (see Appendix A6)
- Age $\geq$ 18 years

Exclusion Criteria
Participant is unable to perform necessary tests in the protocol, in the clinician's judgment.

8.2.3 Clinical Management Issues

8.2.3.1 Administration of the Neuropsychological Tests

The research staff who will administer the neuropsychological tests will be well trained in the standardized format in which these tests should be given. Research staff will participate in follow-up training to ensure correct administration of the tests as necessary. The tests are simple to administer and the total time per study visit will take about 50 minutes.

Table 4. Expected Time Needed for Tests and the CES-D

Test	Time
Grooved Pegboard (both hands)	8 min
Color Trails 1 and 2	3-8 min
WAIS-III Digit Symbol	5 min
HVLT-R, Learning	10 min
HVLT-R, Delayed Recall	3-5 min
Semantic Verbal Fluency	3 min
Finger Tapping (Both Hands)	8 min
CES-D	5 min
**Total**	**45 - 52 min**

The ethos that will underpin the training of the clinicians who will administer these tests is that the tests are serving as measures of the health of the participants' neurocognitive and motor performance. Thus, any discussions about these tests with the participants should impart the notion that we are seeking to define their neurocognitive health rather than their neurocognitive impairment.

All efforts will be made to make the participants feel comfortable and relaxed during the testing. If participants become fatigued or anxious during the testing they will be given an opportunity to rest and the testing may be ceased prematurely at the participant's request. During the administration of the tests, participants will be positively encouraged irrespective of their performance on the tests.

The test scores for the individual tests will be collected on standardized case report forms. The z-scores, ADS scores, and CES-D scores will be calculated at the INSIGHT statistical center. Calculated test scores will not be available in real-time, and will not be returned to the clinical sites. Participants in the Neurology substudy should continue to receive screening for cognitive impairment as per local standard of care. If concerns arise during the administration of tests or the CES-D questionnaire, it is recommended that participants be referred for diagnosis and treatment according to local guidelines.

8.2.3.2 Study Withdrawal

Participants may withdraw from the study at any time at their request, as described in section 4.5 of the START protocol. A participant can withdraw from the Neurology substudy and still be followed in the START study. If a participant withdraws from the main START study, the participant will be withdrawn from the Neurology substudy.

Otherwise, participants should be followed according to protocol, even if a participant chooses not to adhere to the treatment assignment or data collection schedule.

8.2.4 Evaluation

8.2.4.1 Data Analysis

All analyses comparing the treatment groups will be by intent to treat. Comparisons between treatment groups will be stratified by geographical area of enrollment, unless sample sizes are too small or stratification is inappropriate for other reasons.

Primary Analysis
For the primary objective, mean changes in QNPZ-8 scores from baseline will be compared between the early and deferred groups using all measurements through follow-up in a longitudinal mixed-effects model, stratified by geographical area of enrollment. The model will also include “month of visit” as categorical covariate, to adjust for a possible learning effect that would occur equally in both treatment groups.

Secondary Analyses
For each of the component tests of the QNPZ-8, mean changes from baseline in unadjusted test scores and in z-scores will be compared between the early and deferred groups using all measurements through follow-up in longitudinal mixed-effects models. The slopes in these endpoints through year 1 and through follow-up will also be compared between treatment groups, using longitudinal mixed-effects models.

Additionally to the standard longitudinal mixed-effects models that assume Gaussian distributions for the random effects, changes in tests scores through follow-up will be compared between treatment groups using O’Brien’s rank-sum test for multiple endpoints.[80] The two treatment groups will also be compared for changes in test scores from baseline to last visit, and for area under the curve from baseline through follow-up for the QNPZ-8 and each of the component tests.

The early and deferred ART arms will be compared for changes in test scores (QNPZ-8, z-scores, and unadjusted test scores) within subgroups of participants defined by demographics (age, sex, race/ethnicity, education, occupation, employment status, income, urban or rural area of residence), geographic location, CD4+ cell count, HIV RNA level, and other baseline characteristics using longitudinal mixed models. Presence of differential treatment effects across subgroups will be assessed by tests for interaction between subgroup and treatment group indicators in these longitudinal models.

In order to assess presence of a lateral effect on neurocognitive functioning, for finger tapping and grooved pegboard, the interaction between treatment group indicator and indicator for dominant versus non-dominant hand will be assessed in longitudinal models with double-repeated measures (within participant, measures are repeated over time, and across hands). If there is evidence for such an interaction, it will be investigated as to whether it changes over time, for example, by fitting an additional slope parameter. Change in test scores will also be compared between the dominant and non-dominant hands within the treatment groups.

The effect of classes of antiretroviral drugs on neurocognitive functioning will be assessed by comparing the early versus deferred ART treatment effect between subgroups of participants: those whose planned initial ART regimen (determined prior to randomization) contains the drug class under investigation, and those whose planned initial regimen does not contain the drug class. This means, the effect of the drug class will be assessed through the interaction effect between treatment and subgroup indicator. Also, ART regimens will be classified by their estimated level of CNS penetration. To test the hypothesis that higher CNS penetration protects neurocognitive functioning, the interaction effect between CNS penetration and treatment group indicator will be assessed in longitudinal models.

The early and deferred groups will be compared for proportions of participants with neurocognitive impairment using longitudinal models for binary data, for example, generalized estimating equations (GEE). The ADS is an alternative measure of neurocognitive impairment. The early and deferred groups will be compared for changes in ADS using longitudinal mixed models for ordinal data. Subgroup analyses will be performed if sample size is sufficient.

The association of changes in neuropsychological test scores with baseline factors and time-varying factors will be assessed by including these factors as independent variables in longitudinal mixed models. The association will be assessed within the early and deferred groups separately and also pooled across treatment groups if there is no evidence for differential opposite-treatment effects. Follow-up time spent at various levels of the time-varying factors will be tabulated to support the interpretation of the above analyses. For example, to help interpret the association of changes in neurocognitive functioning with the estimated CNS penetration levels of the ART regimens, the CNS penetration level will be included as a time-updated covariate in longitudinal models, and also exposure time to ART regimens of different CNS penetration levels (or no ART) will be tabulated.

Additionally, changes in test scores will be summarized through follow-up (e.g., as area under the curve), and linear regression methods will be used to investigate predictors that can be identified at baseline.

Linear regression methods will be used to assess the association of baseline factors with neuropsychological test scores at baseline in cross-sectional analyses.

Alcohol use, recreational drug use, and depression influence neurocognitive functioning. Alcohol use and recreational drug use (collected in the main START study), and depression scores will be tabulated through follow-up to provide supporting information for the treatment comparisons and to describe the substudy population. Baseline values will be assessed as explanatory factors for changes in neurocognitive functioning. Treatment groups will be compared for changes in CES-D scores using longitudinal mixed models.

8.2.4.2 Data Monitoring

Data for the Neurology substudy will be monitored by the DAIDS DSMB, and study results will be reviewed simultaneously with the main START study.

The protocol team will monitor the standard deviation of change in QNPZ-8 scores and the within-participant correlation structure of the QNPZ-8 scores, pooled across treatment groups, to adjust the sample size if necessary. Additionally, the standard deviation of change in test scores, pooled across treatment groups, will be monitored for each of the component tests of the QNPZ-8, as one measure to control the quality of test administration and to identify need for re-training. The protocol team will be blinded to all other follow-up data until the closing of the START study.

8.2.5 Human Subjects

This protocol must receive the approval of the participating site's IRB or IEC prior to implementation. All study participants must sign an informed consent form (see sample in Appendix A6).

8.3 Informed Consent Substudy

8.3.1 Background and Rationale

Informed consent is widely accepted as an integral part of ethical clinical research[81,82] and includes three distinct elements: (1) disclosure of information to prospective research participants, (2) participant understanding of the information, and (3) a voluntary decision by the participant to enroll in the research. Yet, the goal of well-informed individuals making voluntary choices about research participation is often imperfectly realized. Empirical data suggest that comprehension of important study information, especially side effects and randomization of treatment, although variable, can be unacceptably low.[83,84,85,86,87,88,89] In addition, some participants do not seem to understand that participation is their choice or that they can leave a study at any time.[88,90,91,92,93,94] Somewhat paradoxically, as more evidence emerges that comprehension is inadequate, written consent documents have become increasingly long and complex. Legal, ethical, regulatory, and risk reduction authorities have all had a hand in adding language to consent documents in the interests of “protecting” the human participant as well as the research institution or sponsor.

Many agree that long and complex written documents are unlikely to enhance understanding, a goal of informed consent. Some groups have attempted to improve the consent process by improving the readability of consent documents.[95,96,97,98,99,100] Studies that compared typical consent forms to more simplified designs (usually lower in reading level and sometimes shorter) have found either equivalency between the regular and simplified forms[95,96,97] or statistically and clinically significant improvements in comprehension with a simpler form.[98,99] Studies that measured satisfaction found that subjects clearly preferred the simplified forms.[96,97]

The INSIGHT START study presents an opportunity to compare participant understanding after being randomized to either a standard consent form or a more concise consent form, across a variety of languages and international research sites. The main outcome measure will be prospective participant comprehension of the study specific information, and secondary outcome measures will include participant satisfaction with the consent process, and the resources required for this process. Demonstrating that one form is superior to the other would suggest a new standard or reinforce the current approach to writing consent forms. A demonstration of equivalent results would permit investigators to use the most efficient and easiest of equally effective alternatives.

8.3.2 Purpose

The purpose of this substudy is to evaluate understanding of study information and satisfaction with the consent process among research participants of the START protocol, after receiving information from one of two different types of consent form: a standard consent and a concise consent.

8.3.3 Study Hypothesis

Comprehension of those in the concise consent group will be at least as good as for those in the standard consent group. Satisfaction with the process will be higher for the concise group.

8.3.4 Study Design

This is a randomized study to evaluate and compare the following two groups in START:
- A **standard group** in which participants receive the standard detailed consent form (see Appendix A1) and discuss the study with one or more members of the research team.
- A **concise group** in which participants receive a shorter, less-complex, consent form (see Appendix A7) written at a lower reading level, and discuss the study with one or more members of the research team.

Both the standard and the concise forms contain all of the required elements of informed consent according to the federal regulations found at 45CFR46.116 and 21CFR50.25, and include information needed to make a decision about participating in START.

Consent documents and the questionnaire assessing responses will be translated into the primary language(s) spoken at participating sites.

START Informed Consent Substudy Schematic

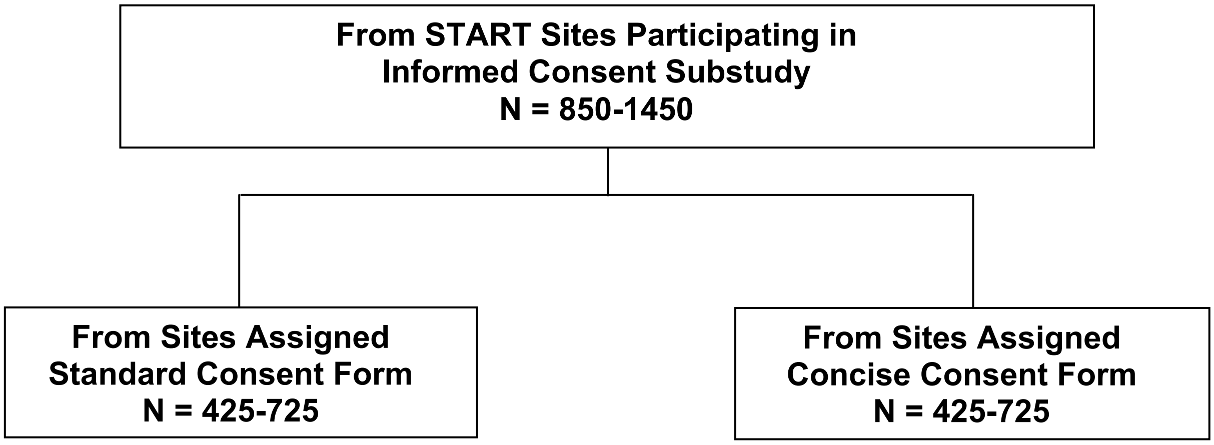

Data collection: Following consent and prior to randomization in main START study

8.3.5 Eligibility Criteria for Sites

- Sites will choose whether or not to participate in the Informed Consent substudy. Interested sites will complete a site questionnaire.

- Sites that choose to participate in the Informed Consent substudy must be registered for both the main START study and the Informed Consent substudy. They must also register to any amendments of either the main study or the substudy.
- There must be at least two willing sites whose primary language is the same.
- Sites may be excluded after review by substudy investigators in cases where the IRB/ IEC stipulates revisions to either consent document that significantly alter the length or readability, making comparison difficult.

8.3.6 Randomization of Participating Sites

For scientific reasons, to minimize contamination, the unit of randomization will be the site rather than the individual participant. Sites will have the option of participating in the consent substudy. Sites that choose not to participate in the substudy will use the standard consent template. Sites that choose to participate in the substudy will submit both the standard and concise forms to their IRB. All reviewing IRBs/IECs will be informed that the consent documents are part of a randomized assessment of understanding and asked to consider minimizing stipulated changes to the consent documents. After IRB/IEC approval, the site will register to START and the Informed Consent substudy and will submit both consents for approval, as per usual procedures. The Informed Consent substudy team will review any changes made to consent documents to ensure that the two documents remain sufficiently different. Similar processes will be followed for any amendments to the consent if the protocol changes.

After registration, the site will be randomized to either the standard form or the concise form. A stratified randomization scheme will be used. Eligible sites will be grouped by primary language of participants, and sites will be centrally assigned to use the concise or standard consent (1:1 allocation) by block randomization within stratum. The site will be notified of their consent form assignment when they receive notification from the SDMC that they are open to randomize participants to START.

The substudy will randomize by site in order to enhance the ability to compare the two consent documents and decrease the possibility that those obtaining consent will alter their usual process because of the substudy. Additionally, randomization by site will minimize the logistical burden at the site because the same consent form and process will be utilized for each participant.

8.3.7 Data Collection

Each randomized site will only use the consent document to which it was assigned for all participants at that site.

Each participant will complete a self-administered questionnaire immediately after giving consent to START but before START randomization. The questionnaire will take approximately 15 to 20 minutes to complete. Consent substudy objectives will be measured using responses to this questionnaire. Research participants' comprehension, especially of study purpose, risks and benefits, randomization, and their right to withdraw will be measured by answers to a series of knowledge questions.

The secondary outcomes will be measured using questions related to satisfaction with the consent process and voluntariness of participation decisions.

The number of individuals completing the questionnaire and the number who refused or failed to complete it will be summarized. Basic information available for those individuals who sign consent at each site (e.g., CD4+ cell count, limited demographic information, whether or not eligible, and whether or not randomized) will be tabulated to assess any differences between the two groups.

For each consented participant, site staff will record the approximate amount of time spent in the consent process, who obtained consent (e.g., the site leader, study coordinator, etc.) and the extent to which the person who obtained consent used the written document. In addition, the site leader will be asked to complete a brief questionnaire describing general site and consent process characteristics.

In addition, the number and type of changes made to the final IRB/IEC approved consent document from each participating site will be compared for length and readability to the sample consent distributed to each site.

8.3.8 Differences between Standard Consent and Concise Consent for START

Both the standard and the concise consent forms present information needed to make a decision about participating in START and contain all of the required elements of informed consent according to the federal regulations found at 45CFR46.116 and 21CFR50.25. These required elements include: statement that activity is research, the purpose and procedures, anticipated risks, possible benefits, alternatives, whom to contact for information, how confidentiality will be protected, how research injury will be handled, and that participation is voluntary. Both forms also include “additional” elements related to unforeseeable risks, circumstances under which participation would be terminated, additional costs, approximate number of participants, and that significant new findings will be provided to participants.

The concise form attempts to present this information in a way that reduces repetition, simplifies explanations and terminology, reduces the reading level, shortens the form, and uses formatting, tables, bullets, etc. to facilitate understanding. It is approximately one-third the length of the standard form.

8.3.9 Study Outcome Measures

The primary endpoint will be the proportion of participants giving correct answers to questions about randomization. An important secondary endpoint will be based on mean scores on the comprehension section of the instrument. Comprehension will include questions on study purpose and procedures, randomization, risks and side effects, and right to refuse. A comprehension score will be based on the number of correct answers to the knowledge questions.

Other secondary endpoints will be measured by a composite score of the satisfaction questions, a composite score of the voluntariness questions, and an evaluation of IRB/IEC required changes to the consent documents.

8.3.10 Sample Size Estimation

Sample size was estimated taking into account the between-site variance in the proportion responding correctly to questions about randomization, the number of participants to be enrolled at each site, the expected percent of participants given the standard consent who will understand the randomization plan, and the expected difference in the proportion understanding the randomization plan between the standard and concise consent form groups.

Cluster randomized trials require a larger sample size than trials with individual randomization. Compared to an individual randomized trial, the sample size must be inflated by an amount that depends on the cluster size and the intra-site correlation coefficient. The intra-site correlation is the amount of site to site variability compared to the total variability.

There is limited information available on site to site variability in the comprehension of randomization, the primary response variable for the study. Data from a consent substudy of a large international trial, Evaluation of Subcutaneous Proleukin® in a Randomized International Trial (ESPRIT), conducted in 2003 was used to estimate sources of variability in the comprehension of randomization. The percent of participants responding correctly to two questions about randomization for sites that enrolled at least ten participants was assessed. Seventeen sites enrolled at least ten participants and on average 50% of the 426 participants at these sites answered both questions correctly. There was considerable variability in the response among sites with the percent of participants responding correctly ranging from 18% to 87%. The estimated coefficient of variation for true proportions between sites was 0.30; the estimated intra-site correlation coefficient was 0.09 (personal communication, Deborah Wentworth).

Sample size for the START substudy is estimated using an approach described by Hayes and Bennett.[101] They use the between-site coefficient of variation (standard deviation divided by mean) to estimate sample size. Their approach is equivalent to use of the intra-site correlation coefficient. If a coefficient of variation of 0.30 is assumed and each site enrolls 25 participants, then 58 sites (29 assigned to use the concise consent and 29 assigned to use the standard consent) are required to detect a difference of 15% of participants responding accurately to the questions concerning randomization (50% versus 65%) with power of 0.80 at the two-sided level of significance of 0.05 (i.e., a total of 1,450 participants). If the coefficient of variation is 0.20, then a total of 34 sites are needed with these assumptions (850 participants). For comparison, if participants were individually randomized, a total of 340 participants would be required.

Given the uncertainty about the coefficient of variation and the percent of participants given the standard consent who will understand the randomization plan, sample size will be re-estimated after the pilot phase of START during which about 30 sites are expected to participate in the consent substudy. The revised sample size will be based on the percent of participants in the standard consent group comprehending the randomization process and the estimated coefficient of variation for the standard consent group.

8.3.11 Data Analysis

The number and characteristics of sites assigned to use the standard and concise consent will be summarized. Baseline site characteristics will include primary language of participants and site staff, projected enrollment for pilot phase of START, and the background of the person administering the consent (e.g., physician, nurse, coordinator). Interested sites for which the Informed Consent substudy or sample informed consents were not approved by local ethics committees will also be summarized.

For sites participating in the Informed Consent substudy, demographic characteristics (e.g., age, gender, ethnicity) of participants consented will be compared. In addition, the number of participants at each site who refused to complete the post-consent questionnaire will be tabulated. Median (IQR) site size (number of participants consented with completed questionnaires) for each treatment group will be summarized.

Logistic regression appropriate for cluster randomized trials (hierarchical models) will be used to compare the standard and concise consent groups for the primary outcome. Odds ratios (concise versus standard consent) and 95% confidence intervals will be cited. Mean levels of continuous secondary outcomes will be compared using methods that account for the within-site correlation. The intra-site correlation coefficient will be cited for the primary and each secondary outcome.

The primary analysis will include all participants who completed the post-consent questionnaire. In a secondary analysis, participants who were consented and did not complete the questionnaire will be considered. In these analyses, participants will be counted as if they did not comprehend the consent questions.

8.3.12 Administration of the Questionnaire

Individual participants will complete the questionnaire immediately after consenting to START, and return it to the research team when completed.

8.3.13 Confidentiality of the Questionnaire Responses

Questionnaires will be coded with the study PID. No other identifying information will be included on the questionnaire. If copies of the questionnaire are kept at the site after data are transmitted, they will be aggregated and kept separate from the participant records.

8.3.14 Study Alternative for Sites and START Participants

A site can choose not to participate in the Informed Consent substudy. Participants at each participating site will be asked to complete a questionnaire after they give consent; however, individual participants can refuse to complete the questionnaire.

8.3.15 Human Subjects

The Informed Consent substudy will be approved by the participating site's IRB/IEC prior to implementation.

Participants will be informed in the consent document that their site is participating in a consent substudy. They will be asked to complete the questionnaire as part of that study, and will be informed that they can decline without affecting their participation in START.

8.3.16 Benefits

Participants will not benefit directly from participation in this substudy, but may contribute to a better understanding of the informed consent process and development of improvements for the future.

8.3.17 Risks

There are no anticipated risks to the participants from completing the substudy questionnaire.

APPENDIX A: SAMPLE CONSENT FORMS

A1. START Main Study Sample Consent

A2. START Study Specimen Storage (Plasma & Urine) Sample Consent

A3. START Study Specimen Storage (Biopsy Tissue & Blood) at Diagnosis of New Malignancy Sample Consent

A4. START Genomics Substudy Sample Consent

A5. START Genomics Substudy Sample Withdrawal Letter

A6. START Neurology Substudy Sample Consent

A7. START Informed Consent Substudy: Sample Concise Consent for Main Study

APPENDIX A1: START MAIN STUDY SAMPLE CONSENT

University of Minnesota: SPONSOR
NIAID: PRIMARY FUNDER

Protocol Title:
Strategic Timing of AntiRetroviral Treatment
(START)

A Multicenter Study of the
International Network for Strategic Initiatives in Global HIV Trials
(INSIGHT)

Short Title of the Study: START

CONSENT FOR PARTICIPATING IN AN NIH-FUNDED RESEARCH TRIAL

SITE LEADER: ___________________________________ PHONE: __________

ALL SITE INSTRUCTION THAT IS INCLUDED IN A TEXT BOX SHOULD BE REMOVED FROM THE SITE'S INFORMED CONSENT FOR SUBJECTS

OHRP Requirements to be read by the sites:

PLEASE NOTE THAT THIS SAMPLE LANGUAGE DOES NOT PREEMPT OR REPLACE LOCAL IRB REVIEW AND APPROVAL. INVESTIGATORS ARE REQUIRED TO PROVIDE THE LOCAL IRB WITH A COPY OF THIS SAMPLE LANGUAGE ALONG WITH THE LANGUAGE INTENDED FOR LOCAL USE. LOCAL IRBS ARE REQUIRED TO WEIGH THE UNIQUE RISKS, CONSTRAINTS, AND POPULATION CONSIDERATIONS AS A CONDITION OF ANY APPROVAL. ANY DELETION OR SUBSTANTIVE CHANGE OF INFORMATION CONCERNING RISKS OR ALTERNATIVE TREATMENT MUST BE JUSTIFIED BY THE INVESTIGATOR, APPROVED BY THE LOCAL IRB, AND NOTED IN THE IRB MINUTES. JUSTIFICATION AND IRB APPROVAL OF SUCH CHANGES MUST BE FORWARDED TO THE INTERNATIONAL COORDINATING CENTER.. SPONSOR-APPROVED CHANGES IN THE PROTOCOL MUST BE APPROVED BY THE LOCAL IRB BEFORE USE UNLESS INTENDED FOR THE ELIMINATION OF APPARENT IMMEDIATE HAZARD. NEW INFORMATION SHALL BE SHARED WITH EXISTING SUBJECTS AT NEXT ENCOUNTER, WITH ALL NEW SUBJECTS PRIOR TO INVOLVEMENT, OR AS THE LOCAL IRB MAY OTHERWISE ADDITIONALLY REQUIRE.

INTRODUCTION

You are being asked to take part in the START study because you are infected with HIV, the virus that causes AIDS, and because you have never taken medicines to treat your HIV infection. This study is being done to find out the best time to start taking HIV medicines. The study will look at whether starting HIV medicines early before most guidelines suggest doing so (in other words, when your CD4+ cell count is fairly high) may help you stay healthy longer than waiting for your CD4+ to drop to the point at which most guidelines recommend you start treatment. Before you can decide whether or not to take part in this study, we would like to explain the purpose of the study, how it may help you, any risks to you, and what is expected of you.

YOUR PARTICIPATION IS VOLUNTARY

This consent form gives you information about the study that will be discussed with you. Once you understand the study, and if you agree to take part, you will be asked to sign this consent form. You will be given a copy to keep.

Before you learn about the study, it is important that you know the following:

- Your participation is entirely voluntary;
- You may decide not to take part or to withdraw from the study at any time without losing the benefits of your routine medical care.

This study is being funded primarily by the National Institute of Allergy and Infectious Diseases (NIAID), U.S. National Institutes of Health (NIH), through a grant to the University of Minnesota, which is the lead institution in the INSIGHT group. The University of Minnesota is the sponsor of this study. This study is also being conducted with additional funding from the Agence Nationale de Recherches sur le SIDA et les Hépatites Virales (ANRS, France); Bundesministerium für Bildung und Forschung (BMBF, Germany); the Australian National Health and Medical Research Council (NHMRC), the Department of Bioethics at The Clinical Center at NIH, NEAT - European AIDS Treatment Network; National Heart, Lung, and Blood Institute; National Institute of Mental Health (NIMH); National Institute of Neurological Disorders and Stroke (NINDS); and the Division of Clinical Research (NIAID). Additional support is being provided by Abbott Laboratories, Inc.; Bristol-Myers Squibb; Gilead Sciences, Inc.; GlaxoSmithKline, Inc.; Merck & Co., Inc, and Tibotec Pharmaceuticals, Ltd.

[The following language should be inserted at sites which are participating in the Informed Consent substudy of START.]

By reading this consent, if you choose to sign it, you are also taking part in a substudy about the consent form itself. We are trying to learn which of two types of consent form is best to help people understand what the START study is about and what they have to do if they join the study. Everybody at this site gets the same consent form that you are reading now. Several sites are doing this substudy. Some of them will use this consent form, and some of them will use the other consent being studied. You will be asked to complete a questionnaire about how well you understand the START study and how you felt about the consent process. You can refuse to complete this questionnaire and still be in the START study.

Site instruction:<br>*If your site is participating in the Informed Consent substudy, please work with your IRB to modify the consent language as little as possible from this template. This will strengthen the validity of the comparison of the two consents.*

WHY IS THIS STUDY BEING DONE?

There are many available guidelines for when people who are infected with HIV should start taking HIV medicines. Most of these agree that if the number of your CD4+ cells (cells in your blood which help fight infection) drops below 350 cells/mm^3, or if you have symptoms of AIDS, you should start taking HIV medicines. The data supporting this recommendation are stronger for those with CD4+ cell counts < 200 cells/mm^3 than for those with CD4+ counts between 200 and 350 cells/mm^3. If your CD4+ cell count is above 350 cells/mm^3, most guidelines suggest that you do not need to start HIV medicines right away. However, there is very little evidence to prove that this is always a good choice.

It is unusual to develop symptoms of AIDS when your CD4+ cell count is above 350, although it does happen in some people. Studies have shown that other serious illnesses, like heart attacks, liver disease, or kidney failure, also happen less often in people whose CD4+ cell count is higher. In these studies, the chance of having one of these serious illnesses appears to increase as your CD4+ cell count drops. In some studies, people who took HIV medicines and kept their CD4+ cell counts high had fewer of these serious illnesses than people not taking HIV medicines. We do not completely understand why this happens.

We are doing this study to find out if the chances of getting one of these serious illnesses or of getting AIDS are less if you start taking HIV medicines at a time when your CD4+ cell count is still fairly high instead of waiting to take HIV medicines at a CD4+ count recommended by the guidelines.

We will also try to learn more about how this strategy of starting HIV medicines early might affect other things, such as your chances of developing other illnesses or resistance to HIV medicines (where the HIV virus changes so that some medicines no longer work against it), how often you need to see a doctor, the cost of your medical care, and your general health and satisfaction with your life.

HOW MANY PEOPLE WILL TAKE PART IN THE STUDY?
We expect that we will need about 4,000 people from around the world to answer this question. Our plan is to enroll about 900 people in the first year. The protocol team will then determine whether we will need more people in the study than the 4,000 we expect to need. The primary study funder will then decide whether the study will be able to continue.

HOW LONG WILL YOU BE IN THE STUDY?
An independent Data and Safety Monitoring Board (DSMB) will review results of the study at least once per year. The DSMB is made up of doctors and other people with a good understanding of HIV and clinical trials, and they are responsible for reviewing the study results to make sure the study continues to be safe and likely to answer the study question. The DSMB may decide to stop the study when they review it if they do not think the study will be able to reach an answer. If the DSMB, the study sponsor, or the study's primary funder decides to end the study after the first year, your participation would end about 6 months later.

On the other hand, if the decision is to continue the study, then you will continue in the study and we will enroll the rest of the people needed to complete the study. Right now we think it will take about 3 years to enroll the study, and at least 3 years of follow-up after that to complete the study. You will continue to be followed in the study group to which you were assigned at the beginning, until the study comes to an end.

HOW WILL THE STUDY WORK?
If you qualify for this study, you will be assigned by chance (like flipping a coin) to one of two treatment groups. You will have an equal (1 to 1) chance of being assigned to either of the two groups. These are the groups:

1) If you are assigned to the Early treatment group, you will start taking HIV medicines right away after enrollment. This is the experimental (research) group.

2) If you are assigned to the Deferred treatment group, you will wait to start HIV medicines until your CD4+ cell count drops to below 350 cells/mm^3, or until you develop AIDS or other symptoms of HIV infection. This is what is recommended by the guidelines and is the current standard of care. This is also called the "control group."

No matter which group you are assigned to, the specific HIV medicines you take will be decided by you and your doctor. Your first HIV medicine regimen will be chosen from a list of the best regimens that doctors currently recommend for people starting treatment. If you need to change HIV medicines during the study, you and your doctor will decide what HIV medicines are best to take next.

The University of Minnesota, the sponsor of this research, gets royalties (payments) from the use of abacavir, one of the HIV medicines that can be used in this study. The sponsor will not tell you or your doctor if you should take abacavir. Only you and your doctor will decide which HIV medicines you will take.

People in the Deferred treatment group can start HIV medicines at any time on the study, if you and your doctor agree that there is a good reason to start before your CD4+ cell count drops to below 350 cells/mm^3. You may develop medical problems that might be related to the HIV virus or your general health. It may be better for you to start HIV medicines for these reasons.

[If applicable: While you are taking part in this study, you may be asked to take part in some smaller related research studies. You may refuse to take part in these smaller studies and still be in this main study.]

WHAT DO YOU HAVE TO DO IF YOU ARE IN THIS STUDY?

Screening

Site Instruction:
It is suggested that you tell potential participants how long screening and follow-up visits are likely to take.

You will have at least two clinic visits to see if you are eligible to be in the study before you can be randomized to one of the two study groups. The first visit must be within 60 days (about 2 months) before you are enrolled, and requires that 5-10 milliliters (mL) (1-2 teaspoons) of blood be drawn to measure your CD4+ cell count.

The second visit must be at least 2 weeks after the first visit before you are randomized. Your study doctor or nurse will ask questions about your health and your HIV infection and will give you a short medical examination. At this visit, you will have about 60-75 mL (about 4-5 tablespoons) of blood drawn. Some of the blood will be used to measure the amount of HIV in your blood, for another CD4+ cell count, for a CD8+ cell count (another type of blood cell that is affected by HIV), and for other routine tests. Some blood will be tested to see if you are infected with hepatitis B or hepatitis C (liver diseases caused by viruses), if you have not already had these tests. We will also do tests to check how your liver and kidneys are working, along with testing the amount of sugar and the types of lipids (fats) in your blood. For the blood tests for sugar and lipids done at this second visit we will ask that you not have anything to eat and only water to drink for at least 8 hours before the visit. You will also be asked for a urine sample, which will be tested to see if it has proteins in it which may indicate you have a kidney problem. These results will be shared with you at your next study visit or when they become available.

Site instruction:
If your site does not have a study-supplied ECG machine and a certified technician, please remove the text in italics in the next paragraph.

At this second visit *you will also have an electrocardiogram (ECG), a routine test that allows the doctor to look at the rhythm of your heart. This involves lying on a table and having 12 small electrodes stuck to your skin for at least 5 minutes while the test is done.* You will be asked what medicines you are taking. You will be asked questions about how often you see the doctor or go to the hospital. You will be asked to answer a questionnaire about how you feel physically and emotionally (a “quality of life” questionnaire). You will be asked about sexual or other behaviors that may infect other people with HIV, and you will receive counseling about how to avoid infecting others. You will be asked whether and how much you smoke, and about your use of alcohol and other drugs. Your answers to these questions will not affect your medical care or your participation in the study.

Your doctor will discuss HIV medicines with you. You and your doctor will decide which specific HIV medicines you will take if you are randomized to start treatment right away. Your doctor will go over the possible side effects of your treatment and how you should take your HIV medicines.

If you and your doctor decide you should take a medicine containing abacavir (Ziagen®, Epzicom®, Kivexa®, or Trizivir®), you will have another blood test to see if you carry the HLA-B*5701 marker. People who have this marker are more likely to have a bad reaction to abacavir. Your doctor will need to have the result of this test before your start taking abacavir, and if you have the marker you will not take abacavir.

If you have had a test showing what medicines your HIV virus might not respond to ("resistance testing"), the results of this test will be recorded for the study.

If you are a woman who can get pregnant, you will have a pregnancy test done using either blood (about 1 teaspoon) or a urine sample. This will be done at the clinic within 14 days before you are randomized. You will be asked about any pregnancies you have had. If you are pregnant or breastfeeding you cannot be in this study, as it would be unsafe for you to do so.

We would like to collect about an additional 15-30 mL (1-2 tablespoons) of blood and a small urine sample from you to be used for additional tests in the future, looking at information important to HIV research. There is a separate consent form for this that your study doctor or nurse will discuss with you. Whether or not you decide to agree to this additional blood and urine collection, you may still be in this study.

Follow-up visits
No matter which group you are assigned to, you will return to the clinic at about 1 month and 4 months after you are randomized, and at least every 4 months after that until the end of the study. At these visits, your study doctor or nurse will ask you about your general health and whether you are having any side effects from your HIV medicines (if you are taking these). You will have a brief physical exam and about 5-10 mL (about 1-2 teaspoons) of blood will be drawn. This blood will be used to measure the amount of HIV in your blood and your CD4+ cell count. You will also be asked for a urine sample to be tested for protein. These results will be shared with you at your next study visit or when they become available. If you are taking HIV medicines, you will also be asked about how much of your prescribed medicine you have taken.

If you have had a test showing what medicines your HIV virus might not respond to ("resistance testing"), the results of this test will be recorded for the study.

If you have consented to have blood stored for this study, at each study visit you will have some blood stored at a central laboratory in the United States. If you have consented to have urine stored for this study, at visits in the first year of the study, and once a year after that, 6 mL (about two teaspoons) of urine will be stored at a central laboratory in the United States. The blood and urine will be used for future studies of the HIV virus, the immune system and complications of HIV medicines. This is described in the separate consent for blood and urine storage.

At your Month 4 visit and at every annual visit, you will be asked to answer the “quality of life” questionnaire about how you feel physically and emotionally. You will also be asked about sexual or other behaviors that may infect other people with HIV and receive counseling about how to avoid infecting others.

For visits in the first year of the study, and once a year after that, you will have another 10-15 mL (about a tablespoon) of blood drawn to see how your kidneys are working.

Once a year at your study visit you will also have an extra 30-45 mL (2-3 tablespoons) of blood drawn to test how your liver is working and to look at the amount of sugar and types of lipids in your blood. You will be asked to not eat anything for at least 8 hours before these tests for sugar and lipids, and to not drink anything except water during that time.

Site instruction:<br>*If your site does not have a study-supplied ECG machine and a certified technician, please remove the text in italics in the next paragraph.*

At this annual visit, *you will have another ECG*. You will be asked what medicines you are taking. You will be asked about your smoking status. If you are a woman who can get pregnant, you will be asked if you have been pregnant, and if so, how that pregnancy turned out. You will be asked questions about how often you see the doctor or go to the hospital. You will be asked about the use of alcohol and other drugs. Your answers to these questions will not affect your medical care or your participation in the study.

It is very important to let your study doctor or nurse know right away if you are sick or injured or in the hospital. This is important for your safety, and also for the study to learn more about illnesses that happen to people with HIV who are treated early (or defer treatment) for their HIV infection. Your study doctor or nurse will ask you for permission to get medical records from your other doctors or from the hospital (if you were in the hospital). You will be asked to sign a “Release of Medical Information” form to allow other doctors or hospitals to share this information with the study team.

You should tell your study nurse or doctor before you take any other medicines or dietary supplements or enroll in other clinical trials. This is important because some medicines should not be taken together. Your study doctor or nurse will help figure out what medicines and supplements are safe for you to take.

If you have consented to have blood stored for this study, we will also collect a 10-mL sample of blood (about 2 teaspoons) if you change HIV medicines because of the amount of HIV virus in your blood or because resistance mutations have been detected. You will be asked for this sample before you start taking your new HIV medicines. This blood will be used sometime in the future to confirm whether your HIV virus had

stopped responding to the HIV medicines you were taking. This is described in the separate consent for blood storage.

You doctor may decide to see you more often than required for the study based on your needs.

HOW WILL YOU GET HIV MEDICINES FOR THE STUDY?
When it is time for you to take HIV medicines – right away if you are in the early group, or later on in the deferred group – your study doctor or nurse will help you find out if you can get HIV medicines through your insurance or some other program or if the study will provide HIV medicines to you.

WHAT IF YOU MOVE?
If you move or transfer your medical care to another doctor, the study staff would like to continue to collect information about your health. If you give permission, your study doctor or nurse will contact your new doctor and ask him or her to provide information about your health. The types of information your new doctor will be asked for are routine things, such as results of laboratory tests (for example, CD4+ cell count and viral load), what medicines you are taking, and whether you have been sick. When you move, you will be asked to sign a “Release of Medical Information” form to allow your new doctor to share this information with the study team. Your new doctor may also ask you to sign a release form.

WHAT ARE THE RISKS AND/OR DISCOMFORTS OF THIS STUDY?
This section describes risks or discomforts that you may experience if you decide to be in this study. There may be additional risks to you (or to your baby, if you are a woman and become pregnant while on the study) that we have no way of knowing about right now. If additional risks or benefits are found during the study, your study doctor or nurse will let you know about them right away.

Possible risks of both ways of treating HIV in the study
Each way of treating HIV disease in this study may be associated with possible benefits and risks. It is not known in the long run which of these strategies will be less risky.

<u>Early group</u>: The long-term risks of using HIV medicines are not clear, especially in people with higher CD4+ cell counts like those joining this study. People who take HIV medicines over many years will probably have more side effects than people who take HIV medicines for shorter periods of time. Also, you may find it hard to take HIV medicines according to your doctor or nurse’s directions for many years, which may lead to your HIV virus becoming resistant to some medicines used to treat it. Using more HIV medicines and staying on HIV medicines for a long time might also lead to HIV resistance. Because of this, you may have fewer HIV medicines you can use when the risk of disease is high.

<u>Deferred group</u>: The long-term risks of **not** using HIV medicines are not clear. People who do not take HIV medicines will probably have a drop in their CD4+ cell count.

There may be a greater chance of developing symptoms of AIDS or other serious illnesses if the CD4+ cell count drops too much. People who do not take HIV medicines may also have an increase in the amount of HIV virus in their blood ("viral load"). People with high viral loads may be more likely to be able to pass the virus to others.

Risks of HIV medicines
All HIV medicines can cause side effects. Your doctor or nurse will discuss with you the risks of the specific HIV medicines that you take. These risks are not specific to this study; they are associated with taking these medicines whether you are in the study or not.

It is also possible for the HIV virus to develop resistance to any anti-HIV drug. It is not known if either way of treating HIV in this study will lead to resistance to more HIV medicines over time.

It is possible that someone could inadvertently find out that you are infected with HIV if you are taking HIV medicines and someone in your household or at work notices you taking them.

Risks of medicine interactions (where one medicine affects how another works)
For your safety, you must tell your doctor or nurse about all medicines, including prescription, over-the-counter (non-prescription), herbal or alternative medicines, and dietary supplements you are taking. This is because there may be serious side effects when other medicines are taken with HIV medicines. Also, please let your nurse or doctor know before you enroll in any other studies while on this study.

Risk of transmitting HIV
Using HIV medicines does not necessarily affect your ability to transmit HIV to other people. You should continue to use precautions to make sure you do not infect someone else. Your study doctor or nurse will tell you about how to protect yourself and other people.

Risks of blood drawing
The risks of having blood taken include pain, bleeding, bruising, lightheadedness, fainting and rarely infection or a blood clot where the needle enters the body. You may feel some anxiety while waiting for your test results to be available. You will have blood tests like those in this study done as part of your usual care, even if you decide not to be in this study.

WHAT ABOUT PREGNANCY AND BREASTFEEDING?
If you are a woman who can become pregnant, you should know that there are some HIV medicines that are risky to take during pregnancy. If your doctor or nurse thinks that one of these medicines is the best choice for you to treat your HIV infection, she or he will talk to you about these risks and about contraception and other options for HIV medicines. In order to be in this study, you must be willing to use appropriate birth

control (contraception) if you are taking HIV medicines. For some medicines, this may mean you need to use two types of birth control at the same time.

Pregnant women cannot join the study since we do not know whether these treatment strategies are safe for them or their unborn babies. If you are a woman who is able to become pregnant, you must have a negative pregnancy test before you join this study. If you become pregnant during the study, tell your study doctor or nurse right away. You may go to another doctor to help manage your pregnancy if you choose to do so. You will be asked to continue your study visits.

Women who are breastfeeding cannot join the study since the effects on the baby from the HIV medicines that are in breast milk are not known.

WHAT ARE THE BENEFITS OF THIS STUDY?
If you take part in this study, there may be a direct benefit to you, but no guarantee can be made. It is also possible that you may receive no benefit from being in this study. What we learn from this study may help us to improve the treatment of other people who are infected with HIV.

Benefits of taking HIV medicines
While not every doctor necessarily agrees with all of the treatment guidelines, very few doubt that when properly used, HIV medicines can bring about an increase in CD4+ cell count and decrease in viral load in most people. So, if you are taking HIV medicines in this study, you may have an increase in your CD4+ cell count and a decrease in your viral load. Because of this, you might stay well for a longer time, be less likely to develop symptoms of HIV infection or infections associated with HIV, and may be less likely to be able to transmit HIV to other people.

Benefits of not taking HIV medicines
If you are not taking HIV medicines, you will not have any of the side effects that HIV medicines can cause. You also will not have to cope with any of the other issues of HIV medicines, like timing when to eat so you can take your medicines.

Benefits of being in a research study
By being in a research study, you may find out sooner about treatments, services, or other things that could help you live with your HIV infection than you would if you were not in a research study. You might have access to tests or treatments that would not otherwise be available to you. You might also feel good about yourself for participating in research that might improve not only your own health but also the health of other people, including people who are close to you.

Benefit to others
In addition to any direct benefit you may receive, your participation in this research may lead to benefits for other people infected with HIV. By finding out the best way to use HIV medicines, this research could lead to better treatment for HIV.

WILL HIV MEDICINES BE PROVIDED AFTER THE STUDY?
After you complete this study, if you and your doctor decide it is best for you to take HIV medicines, you will get HIV medicines in one of two ways. If the study gave you medicines, you will continue to get HIV medicines through the study for up to 6 months after the study is completed. After this time, neither the primary study funder of this study (DAIDS) nor the drug companies that make HIV medicines will provide HIV medicines to you.
This 6-month time period will give your doctor time to arrange for you to get your medicines another way. If you got your medicines through your insurance or national health program during the study, you will need to continue to get your medicines this way after the study.

Site Instruction:
Please include specific information about how your site will provide ART to participants after the study ends.

WHAT IF THERE ARE NEW FINDINGS?
You will be told about any new information learned during the study that might cause you to change your mind about staying in the study. At the end of the study, you will be told when study results may be available and how to learn about them.

WHAT IF YOU DON'T WANT TO BE IN THE STUDY ANY LONGER?
If you enroll in this study, you may decide to stop participating at any time. Withdrawing from this study will not affect the benefits of your regular medical care. However, if you are receiving HIV medicines provided by the study, you will not continue to be given HIV medicines through the study after you withdraw. Your doctor or nurse will help you find another way to get HIV medicines.

CAN YOUR STUDY PARTICIPATION BE STOPPED WITHOUT YOUR CONSENT?
You may be taken off of HIV medicines or started on HIV medicines during the study before you normally would be if your doctor believes it is the best thing for you. You may be taken off the entire study without your consent if:

- Your study doctor decides that continuing in the study would harm you;
- The study is cancelled by the sponsor (the University of Minnesota), the National Institute of Allergy and Infectious Diseases (NIAID), the INSIGHT Executive Committee, regulatory authorities in your country, or your site's Institutional Review Board (IRB)/Ethics Committee(IEC);
- You are in jail or prison; or
- Other administrative reasons.

WHAT OTHER CHOICES DO YOU HAVE BESIDES THIS STUDY?
Before you decide to take part in this study, the study doctor or nurse will talk with you about the other options that are available to you. Possible options include:

- Not taking any treatment for your HIV infection at this time;
- Taking HIV medicines using current standards and guidelines.

WHAT ARE THE COSTS TO YOU?
During the study, *<site should insert specific information about what HIV medicines will be provided for free, if any>.* You, your insurance company, or some other third-party payer must pay for all other medicines, including HIV medicines not listed above and medicines needed to prevent or treat other illnesses. We will provide all clinical and professional services, lab work, and other tests that are part of this study and not part of your regular care at no cost to you.

Site Instruction:<br>*If the above information is not correct for your country/site, please revise the text to explain to the subject any costs to them that may result from participating in the study and obtaining their ART.*

HOW IS YOUR PRIVACY PROTECTED?
Researchers will take every reasonable step to protect the privacy of your health information and to prevent misuse of this information. You will not be identified by name or any other way in any publication about this study. You will be identified only by a code, and personal information from your records will not be released without your written permission. We will collect dates of your study visits and of hospitalizations and certain illnesses so that we can answer the study questions as accurately as possible. We will use your initials as a check on the number code assigned to you to make sure all of your information stays together. If you wish, you may ask your study doctor or nurse to use any 3-letter code instead of your initials and this will not affect your participation in the study or your regular medical care.

[The following paragraph is for U.S. sites only]
In addition to these efforts to keep your information private, the START study and its substudies are covered by a Certificate of Confidentiality from the U.S. Department of Health and Human Services. This certificate means that researchers cannot be forced to give information collected as part of this study to people who are not involved with the study, such as the court system. However, this certificate has limited protection rights. You should know that it does not stop the doctor in charge of this study from taking appropriate steps to prevent serious harm to yourself or others.

[The following paragraph is for international sites only]
Efforts will be made to keep your personal information private, but we cannot guarantee complete confidentiality. Your personal information may be released if required by law. Any publication of this study will not use your name or identify you personally.

[The following paragraph is for all sites}
Your medical and research records may be reviewed by the *[insert the name of the site]* ethics committee (institutional review board, IRB), the U.S. National Institutes of Health

(NIH), the U.S. Office for Human Research Protections (OHRP), and the research staff and monitors, and their designees. Also, the research staff at *[insert the name of the site]* is required to make sure that people not involved with this study do not have access to your research and medical records while collecting personal information about you. They will keep your files in a locked cabinet in a safe place and will handle your personal information very carefully. This will also help to protect your privacy.

WHAT IF YOU ARE INJURED?
If you are injured because of being in this study, *[insert the name of the clinic]* will give you immediate necessary treatment for your injuries. The cost for this treatment will be charged to you or your insurance company. You will then be told where you may receive additional treatment for injuries. There is no program for monetary compensation. You do not give up any of your legal rights by signing this form.

Site Instruction:
If the information is not correct for your country/site, please revise the information to inform the subject of the following: 1. what treatment will be provided to the subject; 2. who will pay for the treatment; 3. if there is any plan for compensation for research-related injury issues, such as lost wages, etc.

WHAT IF YOU HAVE PROBLEMS OR QUESTIONS?
If you ever have questions about this study or in case of research-related injuries, you should contact *[insert the name of the study doctor at your site]* at *[insert the telephone number]*. If you have questions about research subject's rights you can call *[insert the name and title of the appropriate country- or site-specific person]* at *[insert the telephone number]*.

SIGNATURE PAGE FOR CONSENT TO PARTICIPATE IN START MAIN STUDY

If you have read the informed consent (or if you have had it explained to you) and understand the information, and you voluntarily agree to join this study, please sign your name below.

____________________ Participant's name (typed or printed)	____________________ Participant's signature	________ Date
	**OR**	
____________________ Participant's legal guardian or representative	____________________ Legal guardian's signature	________ Date

____________________ Witness's name (typed or printed)	____________________ Witness's signature	________ Date

NOTE: This consent form with the original signatures MUST be retained on file by the principal investigator. A copy must be given to the participant. A copy should be placed in the participant's medical record, if applicable.

A witness to the participant's signature is strongly encouraged.

APPENDIX A2: START STUDY SPECIMEN STORAGE (PLASMA AND URINE) SAMPLE CONSENT

University of Minnesota: SPONSOR
NIAID: PRIMARY FUNDER

Protocol Title:
Strategic Timing of AntiRetroviral Treatment
(START)

A Multicenter Study of the
International Network for Strategic Initiatives in Global HIV Trials
(INSIGHT)

Short Title of the Study: START

CONSENT FOR THE STORAGE OF SPECIMENS OBTAINED WHILE PARTICIPATING IN AN NIH-FUNDED RESEARCH TRIAL

SITE LEADER: ____________________________________ PHONE: __________

ALL SITE INSTRUCTION THAT IS INCLUDED IN A TEXT BOX SHOULD BE REMOVED FROM THE SITE'S INFORMED CONSENT FOR SUBJECTS

INTRODUCTION

You have decided to take part in START, a research study being funded primarily by the National Institute of Allergy and Infectious Diseases (NIAID), US National Institutes of Health (NIH), through a grant to the University of Minnesota, which is the lead institution in the INSIGHT group. The University of Minnesota is the sponsor of START. While you are in this research study, we would like to take some samples of blood and urine from you that might be useful for future research. You are being asked to agree to the storage of these samples and to their use for future research. This consent form gives you information about the collection, storage, and use of your samples. The study staff will talk with you about this information. Please ask if you have any questions.

If you agree to the storage of your samples for future research, you will be asked to sign this consent form. You will get a copy to keep. You do not have to agree to storing samples or sign this consent form in order to be in the main START study.

HOW WILL THE STUDY GET THE SAMPLES FROM YOU?

If you agree to allow the researchers to take additional samples for storage, you will have 15-30 mL (1 to 2 tablespoons) of blood drawn from a vein by a needle at baseline and each study follow-up visit (at 1 month, 4 months, and every 4 months after you start the study). You will also give a urine sample at baseline and at 1, 4, 8, and 12 months, and every 12 months thereafter. You will give this sample by urinating into a container

that your study doctor or nurse will give you. These additional samples will be kept and used for future research.

You will also be asked for a 10-mL (about 2 teaspoons) blood sample if you change HIV medicines because of the amount of HIV virus in your blood or because resistance mutations have been detected. This blood will be used sometime in the future to confirm whether your HIV virus had stopped responding to the HIV medicines you were taking. You and your doctor or nurse will not get the results of these tests.

HOW WILL YOUR SAMPLES BE USED?
Your samples will be used to learn more about HIV infection and its complications. The research may include studies to understand how HIV causes disease and complications and how to best treat or prevent HIV infection and its complications. Samples may also be used to study other problems that are very important to people with HIV infection, such as liver disease, diabetes, or heart disease. Testing may include studies of HIV, studies of other infections that affect people with HIV (for example, hepatitis viruses), studies of your cells, proteins, and other chemicals in your body.

The researchers do not plan to contact you or your doctor or nurse with any results from these studies done on your stored samples. This is because research tests are often done with experimental procedures, and, in general, results from only one research study should not be used to make a decision on how to treat your disease. Your samples will not be sold or used directly to produce commercial products. Research studies using your samples will be reviewed by the National Institutes of Health and a special committee at the institution where the researcher wants to test your samples (an Institutional Review Board or Ethics Committee).

WILL YOUR SAMPLES BE USED FOR STUDIES OF YOUR GENES (DNA)?
These samples will not be used to study your genes (DNA). Tests could possibly be done on the genes of the HIV virus you are infected with.

HOW LONG WILL YOUR SAMPLES BE KEPT?
There is no time limit on how long your samples will be stored.

HOW WILL YOUR SAMPLES BE STORED?
Your samples will be stored at special facilities in the United States that are designed to store samples safely and securely. The storage facilities are designed so that only researchers approved by the study team and the National Institutes of Health can use the samples for future testing. The employees at these facilities who will store and track your specimens will not have information that identifies you by name. An Institutional Review Board (Ethics Committee) will oversee the storage facilities to protect you and other research participants from harm.

DOES STORAGE OF YOUR SAMPLES BENEFIT YOU?

There are no direct benefits to you. The benefit of doing research on stored samples includes learning more about HIV infection and its complications, in order to help people who have HIV.

WHAT ARE THE RISKS?
There are no risks to your health related to storing your samples. Possible risks of having blood drawn include pain, bleeding, bruising, lightheadedness, fainting, and rarely, infection or a blood clot where the needle enters the body. Possible risks to your privacy are described in the next section.

HOW WILL THE STUDY PROTECT YOUR PRIVACY?
In order to keep your information private, your samples will be labeled with a code that can be traced back only to your research clinic, not to you personally. Your personal information (name, address, phone number) will be protected by the research clinic. When researchers are given your stored samples to study, they will not be given your personal information. The results of future tests will not be included in your health records unless you are notified of a special test result and you ask that your test result be sent to another doctor or nurse. Every effort will be made to keep your personal information confidential.

Site Instruction:
If there are any other country/site-specific organizations or personnel that might have access to your subjects' research records, please add them to the above text.

<u>For sites in the US only:</u>
In addition to the efforts of the study staff to help keep your personal information private, we have gotten a Certificate of Confidentiality from the U.S. Federal Government. This certificate means that researchers cannot be forced to tell people who are not connected with this study, such as the court system, about your participation. However, this certificate has limited protection rights. You should know that it does not stop the doctor in charge of this study from taking appropriate steps to prevent serious harm to yourself or others.

WHAT ARE YOUR RIGHTS?
Allowing your samples to be stored is voluntary. You may decide not to have samples stored and still be in the START study. If you decide now that your samples can be stored for future research, you may change your mind at any time. You must contact your study doctor or nurse and let him/her know that you do not want your samples used for future research. Your samples will then no longer be used.

WHAT DO YOU DO IF YOU HAVE QUESTIONS?
For questions about the storage of your samples, contact *[insert the name of the investigator]* at *[insert telephone number]*. For questions about your rights related to the

storage of your samples for research, contact *[insert the name or title of person on the Institutional Review Board]* at *[insert telephone number].*

SIGNATURE PAGE FOR CONSENT FOR STORAGE OF PLASMA AND URINE SPECIMENS OBTAINED WHILE PARTICIPATING IN START

Please read the statements below carefully, and think about your choice. Write your initials on the line in front of your choice for each statement. No matter what you decide, it will not affect your health care or your participation in the START study.

I agree to have blood (15 to 30 mL [1 to 2 tablespoons]) taken at baseline and each study visit; and urine taken at baseline and at study visits at months 1, 4, 8, and 12, and every 12 months afterward; both blood and urine will be stored and used for future research related to HIV infection and its complications. I also agree to have 10 mL (about 2 teaspoons) of blood taken before I change HIV medicines, if the change is because of the amount of HIV virus in my blood or because resistance mutations have been detected. This blood will be used in the future for tests to confirm whether the virus was resistant to the HIV medicines I was taking. I understand that I will not receive these results. I understand that no tests of my genes (DNA) will be done on this blood.

_____Yes
_____No

If you have read this informed consent (or if you have had it explained to you) and understand the information, and you voluntarily agree to have blood and urine stored for this study, please print and sign your name below.

Participant's name (typed or printed)	Participant's signature	Date
	**OR**	
Participant's legal guardian or representative	Legal guardian's signature	Date

Witness's name (typed or printed)	Witness's signature	Date

NOTE: This consent form with the original signatures MUST be retained on file by the site investigator. A copy must be given to the participant. A copy should be placed in the participant's medical record, if applicable.

A witness to the participant's signature is strongly encouraged.

APPENDIX A3: START STUDY SPECIMEN STORAGE (BIOPSY TISSUE AND BLOOD) AT DIAGNOSIS OF NEW MALIGNANCY SAMPLE CONSENT

University of Minnesota: SPONSOR
NIAID: PRIMARY FUNDER

Protocol Title:
Strategic Timing of AntiRetroviral Treatment
(START)

A Multicenter Study of the
International Network for Strategic Initiatives in Global HIV Trials
(INSIGHT)

Short Title of the Study: START

CONSENT FOR THE STORAGE OF BIOPSY TISSUE AND BLOOD OBTAINED AT DIAGNOSIS OF NEW MALIGNANCY WHILE PARTICIPATING IN AN NIH-FUNDED RESEARCH TRIAL

SITE LEADER: ___________________________________ PHONE: __________

ALL SITE INSTRUCTION THAT IS INCLUDED IN A TEXT BOX SHOULD BE REMOVED FROM THE SITE'S INFORMED CONSENT FOR SUBJECTS

INTRODUCTION

You have decided to take part in START, a research study being funded primarily by the National Institute of Allergy and Infectious Diseases (NIAID), U.S. National Institutes of Health (NIH), through a grant to the University of Minnesota, which is the lead institution in the INSIGHT group. The University of Minnesota is the sponsor of this study. If you develop a cancer or malignancy during the course of the START study, you are being asked now to agree to donate a biopsy tissue and a blood sample for future research. You are being asked to agree to the storage of these samples and to their use for future research. This consent form gives you information about the collection, storage, and use of your samples. The study staff will talk with you about this information. Please ask if you have any questions.

If you agree to the storage of your samples for future research, you will be asked to sign this consent form. You will get a copy to keep. You do not have to agree to storage of samples or sign this consent form in order to be in the main START study.

WHEN WILL YOUR TISSUE AND BLOOD BE OBTAINED?

If you have a tissue biopsy to determine if you have a cancer or malignancy we are asking for permission to have some of the tissue taken and stored for future research.

Only tissue in excess of that required for decision-making will be stored. If it is determined that your doctor needs more of your tissue for additional studies, the tissue that is stored may be given back to your doctor.

You are also being asked to donate a blood sample of approximately 20 mL (about 4 teaspoons) for storage so that scientists will also be able to look for any reasons that may explain the cancer. It is best to draw the blood sample before you have received treatment for your cancer or malignancy.

If, during the course of treatment by your doctor, it is necessary to perform additional biopsy procedures for diagnostic reasons, you will be asked at that time to consent to having a portion of that sample sent for storage. No additional blood will be drawn.

Information about you, such as your sex and age, that is not unique to you will be stored along with your sample. This information may help scientists better understand any tests they may do on your stored samples.

HOW WILL YOUR TISSUE AND BLOOD BE STORED?
Your samples will be stored at special facilities in the United States that are designed to store samples safely and securely. The name of these facilities is the AIDS Cancer Specimen Resource (ACSR). This bank has been set up by the National Cancer Institute (NCI) to store tissues and biological fluids from HIV-positive and HIV-negative persons. ACSR specimens are available for scientists studying cancers associated with HIV disease.

HOW WILL YOUR TISSUE AND BLOOD BE USED?
Your samples will be used to learn more about HIV infection, cancer, and other complications. The research may include studies to understand how HIV causes disease and complications and how to best treat or prevent HIV infection and its complications.

The researchers do not plan to contact you or your doctor or nurse with any results from studies done on your stored samples. This is because research tests are often done with experimental procedures, and, in general, results from only one research study should not be used to make a decision on how to treat your disease. Your samples will not be sold or used directly to produce commercial products.

Research studies using your samples will be reviewed by the National Cancer Institute. The studies will also be reviewed by a special committee (an Institutional Review Board or Ethics Committee) at the institution where the researcher wants to test your samples.

WILL YOUR SAMPLES BE USED FOR STUDIES OF YOUR GENES (DNA)?
Studies to detect certain genes associated with cancers or the development of cancers may be done on your stored specimens with appropriate approval by the National Cancer Institute and the Institutional Review Board or Ethics Committee at

the institution where the researcher wants to test your samples. The results of these studies will not identify you by name, but may be shared with other investigators and published in scientific journals.

HOW LONG WILL YOUR SAMPLES BE KEPT?
There is no time limit on how long your samples will be stored.

DOES STORAGE OF YOUR TISSUE AND BLOOD SAMPLES BENEFIT YOU?
It may be that there will be no direct benefit to you by participating in storing your tissue and blood samples. However, there may be possible benefit to medical knowledge and it is hoped that the information gained from these procedures will help in the treatment of HIV-infected individuals in the future.

WHAT ARE THE RISKS?
There are no risks to your health in storing your samples. Possible risks of having blood drawn include pain, bleeding, bruising, lightheadedness, fainting, and rarely, infection or a blood clot where the needle enters the body.

WHAT ARE THE COSTS?
There are no additional costs to you for storage of your tissue and blood samples.

HOW WILL THE STUDY PROTECT YOUR PRIVACY?
In order to keep your information private, your samples will be labeled with a code that can be traced back only to your research clinic, not to you personally. Your personal information (name, address, phone number) will be protected by the research clinic. When researchers are given your stored samples to study, they will not be given your personal information. The results of future tests will not be included in your health records. Every effort will be made to keep your personal information confidential.

> *Site Instruction:*
> *If there are any other country/site-specific organizations or personnel that might have access to your subjects' research records, please add them to the above text.*

For sites in the US only:
In addition to the efforts of the study staff to help keep your personal information private, we have gotten a Certificate of Confidentiality from the U.S. Federal Government. This certificate means that researchers cannot be forced to tell people who are not connected with this study, such as the court system, about your participation. However, this certificate has limited protection rights. You should know that it does not stop the doctor in charge of this study from taking appropriate steps to prevent serious harm to yourself or others.

WHAT ARE YOUR RIGHTS?

Allowing your samples to be stored is voluntary. You may decide not to have samples stored and still be in the START study. If you decide now that your samples can be stored for future research, you may change your mind at any time. You must contact your study doctor or nurse and let him/her know that you do not want your samples used for future research. Your samples will then no longer be used.

WHAT DO YOU DO IF YOU HAVE QUESTIONS?

For questions about the storage of your samples, contact *[insert the name of the investigator]* at *[insert telephone number]*. For questions about your rights related to the storage of your samples for research, contact *[insert the name or title of person on the Institutional Review Board]* at *[insert telephone number].*

SIGNATURE PAGE FOR CONSENT TO THE STORAGE OF BIOPSY TISSUE AND BLOOD OBTAINED AT DIAGNOSIS OF NEW MALIGNANCY WHILE PARTICIPATING IN START

Site Instruction:<br>*This is only a suggested signature page. Sites may use their own signature page.*

Please read the statements below carefully, and think about your choice. Write your initials on the line in front of your choice for each statement. No matter what you decide, it will not affect your health care or your participation in the START study.

If, during the START study, I develop a new cancer or malignancy, I agree to donate a tissue sample from any biopsy that is taken and a blood sample (20 mL [4 teaspoons]) to be stored and to be used for future research related to HIV infection and its complications. I understand that I will not receive these results. I understand that this agreement may include tests of my genes (DNA).

_____Yes

_____No

If you have read this informed consent (or if you have had it explained to you) and understand the information, and you voluntarily agree to have biopsy tissue and blood stored for this study, please print and sign your name below.

Participant's name (typed or printed)	Participant's signature	Date
	**OR**	
Participant's legal guardian or representative	Legal guardian's signature	Date

Witness's name (typed or printed)	Witness's signature	Date

NOTE: This consent form with the original signatures MUST be retained on file by the site investigator. A copy must be given to the participant. A copy should be placed in the participant's medical record, if applicable.

A witness to the participant's signature is strongly encouraged.

APPENDIX A4: START GENOMICS SUBSTUDY SAMPLE CONSENT

University of Minnsota: SPONSOR
NIAID: PRIMARY FUNDER

Protocol Title:
Genomics: A Substudy of

Strategic Timing of AntiRetroviral Treatment
(START)

A Multicenter Study of the
International Network for Strategic Initiatives in Global HIV Trials
(INSIGHT)

Short Title of the Study: START Genomics Substudy

CONSENT FOR PARTICIPATION IN A SUBSTUDY OF AN NIH-FUNDED RESEARCH TRIAL

SITE LEADER: ___________________________________ PHONE: __________

ALL SITE INSTRUCTION THAT IS INCLUDED IN A TEXT BOX SHOULD BE REMOVED FROM THE SITE'S INFORMED CONSENT FOR SUBJECTS

INTRODUCTION
You are being asked to take part in this substudy because you are infected with HIV, the virus that causes AIDS, and because you have joined the START study. The START study is being funded primarily by the National Institute of Allergy and Infectious Diseases (NIAID), U.S. National Institutes of Health (NIH), through a grant to the University of Minnesota, which is the lead institution in the INSIGHT group. The University of Minnesota is the sponsor of this study. This substudy is being done to look at the genetics of people with HIV and of the specific HIV virus infecting them, to see how these might affect the health or treatment of people with HIV.

YOUR PARTICIPATION IS VOLUNTARY
This consent form gives you information about the substudy that will be discussed with you. Once you understand the substudy, and if you agree to take part, you will be asked to sign this consent form. You will be given a copy to keep.

Before you learn about the substudy, it is important that you know the following:
- Your participation is entirely voluntary;
- You can refuse to take part in this substudy and still be in the main START study;
- If you agree to this substudy and then decide to stop participating in the substudy, you may do so at any time for any reason. If you stop participating in this substudy,

you can still be in the main START study, and you will not lose any of the benefits of your regular medical care.

WHY DO A GENOMICS SUBSTUDY IN START?

Research from other medical problems like diabetes, high blood pressure, and hepatitis C infection has shown that people may react differently to either illness or treatment, based on their genes. Genes are inherited and control things like hair color and height. Everyone's genes are a little different. The purpose of this substudy is to collect one blood specimen and store it for researchers who will do genetic testing (testing on your genes) and other related tests in the future. These tests, when linked with your health information from the START study, will help us find out how the genetic make-up of people affects HIV infection and its treatment. For example, some people who carry a specific version of a gene may have serious side effects after taking abacavir (Ziagen®) while others who have a different version of the gene will probably not have that side effect. If your doctor or nurse wants to prescribe abacavir for you, you will have a test for this gene done as part of being in the START study, since we know that this can make a difference in what HIV medicines your study doctor or nurse prescribes to you.

By studying your DNA or genes, this substudy may help scientists learn more about people's ability to fight off HIV infection and its complications. It may also help them learn about how people respond differently to HIV treatment, to find out why some people get different illnesses and side effects of medicines, and how to best treat HIV and other HIV-related conditions. Any future research done on the blood collected for this substudy will be related to HIV disease, the treatment of HIV, or the infections and other health problems common to people who are infected with HIV.

HOW MANY PEOPLE WILL TAKE PART IN THE GENOMICS SUBSTUDY?

Any person enrolled in START may take part in this substudy. We expect about 4,000 people from around the world to enroll in START.

HOW LONG WILL YOU BE IN THE SUBSTUDY?

Your participation in this substudy will end when your blood specimen arrives at the storage facility in usable condition. If it does not arrive in usable condition, we will ask you to sign another consent form and donate another blood specimen.

Your blood will be used for research to be done in the future. If you later decide that you do not want your blood to be used for future research, it will not be used, and every effort will be made to destroy it. Test results from the blood will not be given to you in any case.

Your participation in the START study will continue until the START study ends or you withdraw your consent to be in it. Any health information from the START study linked to your blood specimen for this substudy will only be used for research.

WHAT DO YOU HAVE TO DO IF YOU ARE IN THE GENOMICS SUBSTUDY?

The research staff will collect one blood specimen of about 6 mL (a little more than 1 teaspoon). This will be done by using a needle to get blood from a vein in your arm. This procedure will only take a few minutes of your time. This will happen only one time.

HOW WILL YOUR BLOOD BE USED?
Your blood will be used only to learn more about HIV infection and about health problems common to people who are infected with HIV. This may include tests to better understand why some people with HIV get sicker faster than others and why medicines might work better in one group of HIV-infected people compared with other groups.

Researchers involved with this blood collection project do not know yet exactly which tests will be done.

You and your study doctor or nurse will not get any results from the tests done on your blood collected in this substudy. These tests will only be used for research and may not apply to your clinical care. If these tests show a promising result that has a strong and clear effect on the health condition of other research participants, every reasonable effort will be made to provide you with additional genetic testing outside of this substudy.

Your blood sample collected for this substudy will become the property of the START study. Your blood will not be sold or used to make commercial products. It will not be tested for any specific research study unless the plan for using your blood is approved by the INSIGHT Executive Committee, the DAIDS (National Institutes of Health/NIH), and a special committee at the researcher's institution (an Institutional Review Board or Ethics Committee).

Researchers will write reports about new findings and results that they learn about from doing future tests on your blood. These reports will be shared with participating sites. These findings will also be submitted for publication in scientific or medical journals to share with you and the public. Any publications about this research will not use your name or identify you personally.

HOW LONG WILL YOUR BLOOD BE KEPT?
Your blood specimen will be stored as long as funding is available for storage and testing.

HOW WILL YOUR BLOOD BE STORED?
Your blood specimen will be stored safely and securely at a special facility in the United States called a specimen repository. This facility follows strict procedures so that only approved researchers can use the stored specimen for future testing. The employees at this facility who will store and track your blood specimen will not have information that identifies you by name.

[Alternative to Previous Paragraph for International Sites Only]

Your blood specimen will be stored safely and securely at a special facility called a specimen repository. The repository may be located within the United States. This facility follows strict procedures so that only approved researchers can use the stored specimen for future testing. The employees at this facility who will store and track your blood specimen will not have information that identifies you by name.

HOW IS YOUR PRIVACY PROTECTED?
Researchers will take every reasonable step to protect the confidentiality of your health information and to prevent misuse of this information. They will also make sure your blood sample is handled with care at the storage facility and that your privacy is protected. For example, your research records will be identified by a code. Your blood sample and results of any genetic testing will be identified by a second code. Only a few statisticians associated with the START study will have access to both codes in order to analyze the test results. These statisticians will not have access to any information that can identify you.

[For U.S. Sites Only]
In addition to these efforts to keep your information confidential, the START study is covered by a Certificate of Confidentiality from the U.S. Department of Health and Human Services. This certificate means that researchers cannot be forced to give information collected as part of this substudy to people who are not involved with the substudy, such as the court system. However, this certificate has limited protection rights. You should know that it does not stop the doctor in charge of this substudy from taking appropriate steps to prevent serious harm to yourself or others. Federal and state laws also help protect research participants and others who have genetic testing done.

[For International Sites Only]
Efforts will be made to keep your personal information confidential, but we cannot guarantee complete confidentiality. Your personal information may be released if required by law. Any publication of this substudy will not use your name or identify you personally.

[For All Sites]
Your medical and research records may be reviewed by the *[insert the name of the site]* ethics committee (institutional review board, IRB), the U.S. National Institutes of Health (NIH), the U.S. Office for Human Research Protections (OHRP), and the research staff and monitors, and their designees. Also, the research staff at *[insert the name of the site]* is required to make sure that people not involved with this substudy do *not* have access to your research and medical records while collecting personal information about you. They will keep your files in a locked cabinet in a safe place and will handle your personal information very carefully. This will also help to protect your privacy.

WHAT ARE THE BENEFITS OF THE GENOMICS SUBSTUDY?
There are no benefits to you for participating in this substudy. Information learned from the blood collected as a part of this substudy may help others with HIV in the future.

WHAT ARE THE RISKS OF THE SUBSTUDY?
There are few risks involved with your participation in this project. Drawing blood may result in a little pain and slight bruising where the needle goes into your skin. You may also faint, feel lightheaded, bleed, or develop a small blood clot where the needle goes into your skin. Very rarely, your skin may get infected. Another small but unlikely risk is the possibility of others finding out about your participation in this substudy.

CAN YOUR GENOMICS SUBSTUDY PARTICIPATION BE STOPPED WITHOUT YOUR CONSENT?
Your study doctor or nurse may need to take you off the substudy early without your permission if:
- The substudy is cancelled by the sponsor (the University of Minnesota), the National Institute of Allergy and Infectious Diseases (NIAID), the INSIGHT Executive Committee, regulatory authorities in your country, or your site's Institutional Review Board (IRB)/Ethics Committee(IEC);
- You are in jail or prison.

WHAT OTHER CHOICES DO YOU HAVE BESIDES THIS SUBSTUDY?
You can choose not to be in this substudy and still be in the START study. Please talk to your study doctor or nurse about this and other choices available to you.

WHAT IF YOU ARE INJURED?
If you are injured from having your blood drawn for this substudy, you will receive proper medical care. The cost for such medical care will be paid by you or by another party. There is no program for compensation through this substudy. You will not be giving up any of your legal rights by signing this consent form.

Site Instruction:<br>*If the information is not correct for your country/site, please revise the information to inform the subject of the following: 1. what treatment will be provided to the subject; 2. who will pay for the treatment; 3. if there is any plan for compensation for research-related injury issues, such as lost wages, etc.*

WHAT ARE THE COSTS TO YOU?
There is no cost to you to be in this substudy. The substudy will cover all costs for storage of your blood and for future tests.

WHAT ARE YOUR RIGHTS AS A RESEARCH PARTICIPANT?
Allowing your blood to be collected, stored, and tested at a later date is voluntary. Your decision will not affect your right to take part in the main START study or affect your receipt of medical care.

If you sign the consent that your blood can be stored for research to be done at a later date, including studies of your genes, you can change your mind at any time. If you

change your mind, you must write a letter to *[insert the name of the principal investigator*] at the *[insert the name and address of the site]* to let them know that you do not want your blood specimen used for future research. A sample letter will be given to you as a guide to help you express your request in writing.

When *[insert the name of the principal investigator]* receives your letter, the research staff will contact you to come to the clinic to sign and date in this original informed consent to verify your decision. A second copy of this consent will be given to you as proof that we received your request. If we do not hear from you within 30 days after getting your letter to withdraw from this plan, we will send your request to the storage facility.

If you decide to withdraw consent for this substudy, your blood sample and any parts separated from it will not be used. Every effort will be made to destroy your blood sample and any parts separated from it. If some testing has already been done on your blood sample, the results from this testing will remain as part of this research. The research staff at the *[insert the name of the site]* will notify you of the date your blood specimen and any of its parts were destroyed. You have the right to change your mind and to re-enroll in this substudy. If you change your mind, you must sign a new consent form and donate another blood specimen.

WHOM DO YOU CALL IF YOU HAVE QUESTIONS OR PROBLEMS?
For questions about this substudy or about the storage or use of your blood, and in case of any research-related injury,contact:
- *[name of the investigator or other study staff]*
- *[telephone number of above]*

For questions about your rights as a research participant contact:
- *[name or title of person on the ethics committee (Institutional Review Board, IRB) or other organization appropriate for the site]*
- *[telephone number of above]*

SIGNATURE PAGE FOR START GENOMICS SUBSTUDY CONSENT

Site Instruction:
This is only a suggested signature page. Sites may use their own signature page.

You agree to donate a blood sample for the START Genomics Substudy to be stored and used for future genetic research related to HIV infection, its complications, and related illnesses. You understand that the testing and research on your blood sample will be done at a later date. You also understand that you and your study doctor or nurse will not get results from any testing done on your blood sample. Further, you understand that your blood sample may be stored for a long time.

Unless you decide at some point in the future to submit a request in writing to no longer take part in this substudy, you agree to let the START study researchers use your blood sample for approved HIV-related genetic testing and research whether or not you are still alive. You also agree to give permission/authorization for the use and disclosure of your personal health information as described in this consent form for the purposes of this research.

If you have read this consent form (or had it explained to you) and all of your questions were answered, and you agree to take part in this substudy, please print and sign your name, and give the date of your signature below. By doing so, you also confirm receiving the *sample study withdrawal letter*.

Participant's name (typed or printed)	Participant's signature	Date

OR

Participant's legal guardian or representative	Legal guardian's signature	Date

Witness's name (typed or printed)	Witness's signature	Date

NOTE: This consent form with the original signatures MUST be retained on file by the site investigator. A copy must be given to the participant. A copy should be placed in the participant's medical record, if applicable.

A witness to the participant's signature is strongly encouraged.

APPENDIX A5: START GENOMICS SUBSTUDY SAMPLE WITHDRAWAL LETTER

**SAMPLE STUDY WITHDRAWAL LETTER
FOR STUDY PARTICIPANTS WHO CHOOSE AT A LATER DATE
TO STOP TAKING PART IN THE START GENOMICS SUBSTUDY AND
NOT TO HAVE THEIR BLOOD SPECIMEN USED FOR FUTURE GENETIC TESTING**

NOTE FOR THE STUDY PARTICIPANT: PLEASE KEEP A COPY OF THIS LETTER FOR YOUR RECORDS ALONG WITH A COPY OF YOUR SIGNED CONSENT FORM.

Date of Letter

Name of Site Leader at the Participating Site
Name of Facility
Name of Department
Address (Street Number and Name)
Address (City, State, Country, Postal Code)

Dear Dr. (Name of Site Leader):

I am a study participant for the START Genomics Substudy. I have decided to stop participating in this research. Therefore, I would like to have my blood sample destroyed and not used for future genetic testing.

I understand that any information collected from testing my blood sample before you received this request will remain a part of the research study and kept confidential. I also understand that if I decide to be a part of this study again, I must sign a new consent form and donate another blood sample.

Please let me know when my blood sample is destroyed.

Thank you for respecting my decision.

Cordially,

Your signature

APPENDIX A6: START NEUROLOGY SUBSTUDY SAMPLE CONSENT

University of Minnesota: SPONSOR
NIAID: PRIMARY FUNDER

Protocol Title:
Neurology: A Substudy of
Strategic Timing of AntiRetroviral Treatment
(START)

A Multicenter Study of the
International Network for Strategic Initiatives in Global HIV Trials
(INSIGHT)

Short Title of the Study: START Neurology Substudy

CONSENT FOR PARTICIPATION IN A SUBSTUDY OF AN NIH-FUNDED RESEARCH TRIAL

SITE LEADER: ___________________________________ PHONE: __________

ALL SITE INSTRUCTION THAT IS INCLUDED IN A TEXT BOX SHOULD BE REMOVED FROM THE SITE'S INFORMED CONSENT FOR SUBJECTS

INTRODUCTION

You are being asked to take part in this substudy because you are infected with HIV, the virus that causes AIDS, and because you have joined the START study. This substudy is being done to look at whether there are changes in the function of the brain depending on whether you start HIV medicines early or wait until the guidelines suggest doing so.

YOUR PARTICIPATION IS VOLUNTARY

This consent form gives you information about the study that will be discussed with you. Once you understand the study, and if you agree to take part, you will be asked to sign this consent form. You will be given a copy to keep.

Before you learn about this substudy, it is important that you know:

- Your participation is completely voluntary;
- You can refuse to take part in this substudy and still be in the main START study;
- If you agree to this substudy and then decide to stop participating in the substudy, you may do so at any time for any reason. If you stop participating in this substudy, you can still be in the main START study, and you will not lose any of the benefits of your regular medical care.

If you agree to take part in this substudy, you will be asked to sign this consent form. The doctor in charge of this substudy at this location is *[insert the name of the Site Leader]*. He or she will keep the original copy of this consent to place in your medical record. You will also receive a copy to keep.

This study is being funded by DAIDS, National Institute of Allergy and Infectious Diseases (NIAID); the National Institute of Neurological Disorders and Strokes (NINDS); and the National Institute of Mental Health (NIMH), all in the U.S. National Institutes of Health (NIH), through a grant to the University of Minnesota, which is the lead institution in the INSIGHT group. The University of Minnesota is the sponsor of this study.

WHY IS THE NEUROLOGY SUBSTUDY BEING DONE?
HIV can affect how well the brain functions and may cause problems like poor concentration and memory: this is called “HIV-related cognitive impairment.” HIV-related cognitive impairment usually only happens when a person’s CD4+ count is less than 200 cells/mm^3, and HIV medicines have been proven to help to reverse memory and concentration problems in this situation.

When CD4+ counts are much higher, like over 500 cells/mm^3, HIV may cause changes in brain function in some people that are so small that the people don’t even notice them. We don’t know whether starting HIV medication early helps to treat or prevent these small changes; it may be that it’s perfectly safe to wait until your CD4+ cells drop to the point at which most guidelines recommend that you start treatment (350 cells/mm^3 or fewer). There is very little information to help answer this question. Therefore this substudy will look at whether starting HIV medicines earlier or waiting until the CD4+ cell count drops below 350 cells/mm^3 makes any difference to brain function in people with HIV.

HOW MANY PEOPLE WILL TAKE PART IN THE NEUROLOGY SUBSTUDY?
We expect that we will need about 600 people to answer this question.

HOW LONG WILL YOU BE IN THE SUBSTUDY?
You will continue to be followed in the substudy until the main START study comes to an end. Right now we think it will take about 6 years to complete the study.

WHAT DO YOU HAVE TO DO IF YOU ARE IN THIS SUBSTUDY?

Screening
After you consent to the main START study and this substudy, your study doctor or nurse will do eight tests that measure your memory, speed of thinking, concentration, movement, and coordination. You will be asked to fill out a short questionnaire about any depression you may be having. The tests and questionnaire will take about an hour for you to complete.

The tests include:
- Placing pegs in holes on a board – this tests your speed of thinking and coordination

- Connecting colored circles on a piece of paper (two versions) – these test your movement and coordination, your speed of thinking, and your concentration
- Tapping your index fingers – this tests your movement and coordination
- Matching symbols and numbers – this tests your movement and coordination, your speed of thinking, and your concentration
- Remembering a list of words that will be read out loud to you – this tests your concentration, your memory, and your speed of thinking
- To list words of the same kind, for example, words describing food – this tests your speed of thinking, and your concentration

At this first visit only, you will also be asked about how many years of schooling you have had, whether you live in a town or a rural area, and about your occupation and income level.

Follow-up visits
You will return to the clinic for study visits at months 4, 8, and 12, and every 12 months after that. At each of these visits, your doctor or study nurse will do exactly the same tests that were done at your first visit, as described above. You will be asked to fill out the same short questionnaire about any depression you may be having. At each study visit, the tests and questionnaire will take about an hour for you to complete.

WILL YOU GET THE RESULTS OF THE TESTS DONE IN THIS SUBSTUDY?
The results of the tests done in this substudy will not be available to you. This is because these tests are never used alone to diagnose HIV-related cognitive impairment or other brain disorders. Your study doctor or nurse will explain and recommend available treatment options, should you have or develop serious brain problems.

Your study doctor or nurse will go over your responses to the depression questionnaire to make sure you are getting appropriate care if you are having any depression.

WHAT ARE THE RISKS AND/OR DISCOMFORTS OF BEING IN THIS SUBSTUDY?
You may become tired during the tests of brain function. If this happens, it’s fine for you to take a break for a while and then continue.

WHAT ARE THE BENEFITS OF BEING IN THIS SUBSTUDY?
If you take part in this substudy, there may be a direct benefit to you from having your brain function looked at regularly, but no guarantee can be made. It is also possible that you may receive no benefit from being in this study. Information learned from this study may help others who have HIV.

WHAT ABOUT PREGNANCY AND BREASTFEEDING?
There are no risks related to pregnancy or breastfeeding.

WHAT IF THERE ARE NEW FINDINGS?
You will be told about any new information learned during this substudy that might cause you to change your mind about staying in it. At the end of the main START

study, you will be told when substudy results may be available and how to learn about them.

WHAT IF YOU WANT TO WITHDRAW FROM THE NEUROLOGY SUBSTUDY?
If you enroll in this substudy, you may decide to stop participating at any time. Withdrawing from this study will not affect your medical care, and you can continue to be in the main START study.

CAN YOUR SUBSTUDY PARTICIPATION BE STOPPED WITHOUT YOUR CONSENT?
You may be taken off the Neurology substudy without your consent if:

- Your study doctor decides that continuing in the substudy would harm you;
- The substudy is cancelled by the sponsor (the University of Minnesota), the National Institute of Allergy and Infectious Diseases (NIAID), the INSIGHT Executive Committee, regulatory authorities in your country, or your site's Institutional Review Board (IRB)/Ethics Committee(IEC);
- You are in jail or prison
- Other administrative reasons.

WHAT ARE THE ALTERNATIVES TO BEING IN THIS SUBSTUDY?
You can choose not to be in this substudy. Please talk to your study doctor or nurse about this and other choices available to you.

ARE THERE ANY COSTS TO YOU?
There is no cost to you to be in this substudy. The substudy will cover all costs for doing the tests described earlier in the consent.

WHAT IF YOU ARE INJURED?
There is very little chance that you would be injured by participating in this substudy. If you are injured as a result of participating in this substudy, you will receive proper medical care. The cost for such medical care will be paid by you or by another party. There is no program to compensate you through this substudy. You will not be giving up any of your legal rights by signing this consent form.

Site Instruction:
If the information is not correct for your country/site, please revise the information to inform the subject of the following: 1. what treatment will be provided to the subject; 2. who will pay for the treatment; 3. if there is any plan for compensation for research-related injury issues, such as lost wages, etc.

HOW IS YOUR PRIVACY PROTECTED?
Researchers will take every reasonable step to protect the privacy of your health information and to prevent misuse of this information. For example, your research records will be identified by a code. You will not be identified by name or any other way in any publication about this substudy.

[The following paragraph is for U.S. sites only]
In addition to these efforts to keep your information private, the START study and its substudies are covered by a Certificate of Confidentiality from the U.S. Department of Health and Human Services. This certificate means that researchers cannot be forced to give information collected as part of this substudy to people who are not involved with the substudy, such as the court system. However, this certificate has limited protection rights. You should know that it does not stop the doctor in charge of this substudy from taking appropriate steps to prevent serious harm to yourself or others.

[The following paragraph is for international sites only]
Efforts will be made to keep your personal information private, but we cannot guarantee complete confidentiality. Your personal information may be released if required by law. Any publication of this substudy will not use your name or identify you personally.

[The following paragraph is for all sites}
Your medical and research records may be reviewed by the *[insert the name of the site]* ethics committee (institutional review board, IRB), the U.S. National Institutes of Health (NIH), the U.S. Office for Human Research Protections (OHRP), and the research staff and monitors, and their designees. Also, the research staff at *[insert the name of the site]* is required to make sure that people not involved with this substudy do not have access to your research and medical records while collecting personal information about you. They will keep your files in a locked cabinet in a safe place and will handle your personal information very carefully. This will also help to protect your privacy.

WHOM DO YOU CALL IF YOU HAVE QUESTIONS OR PROBLEMS?
For questions about the Neurology substudy or in case of a research-related injury contact:

- *insert name of the investigator or other study staff*
- *insert telephone number of above*

For questions about your rights as a research participant, contact:

- *insert name or title of person on the ethics committee (Institutional Review Board, IRB) or other organization appropriate for the site*
- *insert telephone number of above*

SIGNATURE PAGE FOR THE START NEUROLOGY SUBSTUDY CONSENT

Site Instruction:<br>*This is only a suggested signature page. Sites may use their own signature page.*

You have already agreed to join the main START study, and you can still be in the main START study even if you do not want to join this substudy.

If you have read this informed consent (or if you have had it explained to you) and understand the information, and you voluntarily agree to join ***the START Neurology Substudy***, please sign your name below.

____________________	____________________	__________
Participant's name (typed or printed)	Participant's signature	Date
	**OR**	
____________________	____________________	__________
Participant's legal guardian or representative	Legal guardian's signature	Date

____________________	____________________	__________
Witness's name (typed or printed)	Witness's signature	Date

NOTE: This consent form with the original signatures MUST be retained on file by the principal investigator. A copy must be given to the participant. A copy should be placed in the participant's medical record, if applicable.

A witness to the participant's signature is strongly encouraged.

APPENDIX A7: START INFORMED CONSENT SUBSTUDY: SAMPLE CONCISE CONSENT FOR MAIN STUDY

University of Minnesota: SPONSOR
NIAID: PRIMARY FUNDER

Protocol Title:
Strategic Timing of Anti-Retroviral Treatment
(START)

A Multicenter Study of the
International Network for Strategic Initiatives in Global HIV Trials
(INSIGHT)

Short Title of the Study: START

CONSENT FOR PARTICIPATION IN AN NIH-FUNDED RESEARCH TRIAL

SITE LEADER: ___________________________________ PHONE: __________

ALL SITE INSTRUCTION THAT IS INCLUDED IN A TEXT BOX SHOULD BE REMOVED FROM THE SITE'S INFORMED CONSENT FOR SUBJECTS

OHRP Requirements to be read by the sites:

PLEASE NOTE THAT THIS SAMPLE LANGUAGE DOES NOT PREEMPT OR REPLACE LOCAL IRB REVIEW AND APPROVAL. INVESTIGATORS ARE REQUIRED TO PROVIDE THE LOCAL IRB WITH A COPY OF THIS SAMPLE LANGUAGE ALONG WITH THE LANGUAGE INTENDED FOR LOCAL USE. LOCAL IRBS ARE REQUIRED TO WEIGH THE UNIQUE RISKS, CONSTRAINTS, AND POPULATION CONSIDERATIONS AS A CONDITION OF ANY APPROVAL. ANY DELETION OR SUBSTANTIVE CHANGE OF INFORMATION CONCERNING RISKS OR ALTERNATIVE TREATMENT MUST BE JUSTIFIED BY THE INVESTIGATOR, APPROVED BY THE LOCAL IRB, AND NOTED IN THE IRB MINUTES. JUSTIFICATION AND IRB APPROVAL OF SUCH CHANGES MUST BE FORWARDED TO THE INTERNATIONAL COORDINATING CENTER. SPONSOR-APPROVED CHANGES IN THE PROTOCOL MUST BE APPROVED BY THE LOCAL IRB BEFORE USE UNLESS INTENDED FOR THE ELIMINATION OF APPARENT IMMEDIATE HAZARD. NEW INFORMATION SHALL BE SHARED WITH EXISTING SUBJECTS AT NEXT ENCOUNTER, WITH ALL NEW SUBJECTS PRIOR TO INVOLVEMENT, OR AS THE LOCAL IRB MAY OTHERWISE ADDITIONALLY REQUIRE.

Site instruction:
If your site is participating in the Informed Consent Substudy, please work with your IRB to modify the consent language as little as possible from this template. This will strengthen the validity of the comparison of the two consents.

We invite you to join this HIV/AIDS research study. It is up to you whether or not you want to join this study. Please ask questions and take as much time as you need to decide.

WHY ARE WE DOING THIS RESEARCH?
We want to find out whether it is better for people with HIV infection to start taking HIV medicines as soon as they know they have HIV or to wait until current guidelines recommend

that they start HIV medicines. To find this out, people like you who are HIV infected but have not taken HIV medicines will be put into one of two groups through a random process- like flipping a coin. If you join, you have an equal chance of being in either group:

- The **DEFERRED** group (half of those who join) will start HIV medicines according to current guidelines. HIV medicines would start when you develop AIDS or HIV symptoms or when CD4 cells fall below 350 cells/mm^3. CD4 cells are immune cells that normally fight infection, but fall over time in HIV infection.

- The **EARLY** group (half of those who join) will start taking HIV medicines right away.

Whether you are in the **DEFERRED** or **EARLY** group, you and your doctor will decide which specific HIV medicines you will take from a list of standard HIV medicines.

The University of Minnesota, the sponsor of this research, gets royalties (payments) from the use of abacavir, one of the HIV medicines that can be used in this study. The sponsor will not tell you or your doctor if you should take abacavir. Only you and your doctor will decide which HIV medicines you will take.

We will also study whether the cost of medical care, general health, and satisfaction with life differs between the **DEFERRED** and **EARLY** group. And, we will study whether the virus changes and becomes resistant to some HIV medicines. We are testing two different forms of written information to find out which is easier to understand. You will get one of these written forms.

We plan to enroll 4000 people with HIV infection and follow them for 3 to 6 years.

WHAT WILL HAPPEN DURING THE RESEARCH?

Before the study begins
During 2 clinic visits, we will do some tests to see if you qualify:

- <u>Visit 1</u>: Draw a small amount of blood to measure how many CD4 cells you have.

- <u>Visit 2</u>: (at least 2 weeks later)
    - Ask questions about your health, medical history, sexual behaviors, use of alcohol and drugs, and quality of life.
    - Check to see how healthy you are by doing a physical examination, taking a small amount of blood and urine, *and doing a routine test of your heart (an ECG)*. You should not eat or drink anything except water for 8 hours before this clinic visit. We will give you the results of these tests when they are ready.
    - If you are a woman, test your blood or urine to see if you are pregnant.

Site instruction:
If your site does not have a study-supplied ECG machine and a certified technician, please remove the text in italics in the preceding list.

After the study starts
Whether you are in the **DEFERRED** or **EARLY** group, you will come to the clinic 1 and 4 months after you start the study and then about every 4 months until the study ends. Each time we will ask questions about your health, medicines, and side effects.

At the beginning of the Study	At 1 and 4 months, and every 4 months after	Additional tests done once a year
• *Optional* Consent Questionnaire<br>• Assigned to either the **DEFERRED** or the **EARLY** group.	• Blood tests for HIV viral load and CD4 count<br>• Physical exam<br>• Health-related Questionnaires (at month 4 and once a year)<br>• *Optional* blood to store for future research<br>• Urine test for general health and *optional* urine to store for future research (once a year after the first year)	• Blood tests to check general health<br>• *ECG of your heart*

Site instruction:
If your site does not have a study-supplied ECG machine and a certified technician, please remove the text in italics in the preceding table.

At any time during the study - Please talk to your study doctor or nurse *as soon as possible if*:

- *You are sick or hurt or in the hospital for any reason.*
- *You want to join any other research study*
- *You take any other medicines, including over-the-counter, herbal, or alternative medicines.*
- *You move or transfer your care to another doctor.*
- *You become pregnant*.

With your permission, we will contact your doctor(s) to ask about any medicines you are taking or any illnesses, hospitalizations, or pregnancies you have had.

FOR WOMEN: PREGNANCY AND BREASTFEEDING
You cannot join if you are pregnant or breastfeeding. We will test to see if you are pregnant. To be in the study you must use appropriate birth control. Please discuss this with your doctor.

If you do become pregnant during the study, please tell the study doctor or nurse *right away* because your HIV medicines may need to be changed. We will ask you to continue your study visits.

WHAT ARE THE RISKS OF BEING IN THIS STUDY?
All HIV medicines have some side effects. No one knows whether people in the **DEFERRED** or **EARLY** group will have fewer risks overall. There may also be risks that we do not know about now. We will tell you if we learn about new risks or any other information that might be important to you.

Possible risks of being in the DEFERRED group:
- A drop in CD4 cell count that could increase the chance of developing HIV symptoms or AIDS.
- An increased chance of infecting others with HIV because of virus in your blood.

Possible risks of being in the EARLY group:
- More side effects than people who take medicines for a shorter time.
- Difficulty sticking to a schedule when taking medicines for a long time.
- Increased chance that the HIV virus will become resistant to the HIV medicines you are on.

Other possible study risks are:
- Side effects because of an interaction of HIV medicines with other medicines you might be taking, including herbal or alternative medicines.
- Pain, bleeding, bruising, feeling lightheaded, anxious, or rarely fainting or an infection when blood is drawn. Some people feel anxious while waiting for test results.
- Discomfort from some of the questions we ask you.

WHAT ARE THE BENEFITS OF BEING IN THIS STUDY?
Although taking HIV medicines can help people with HIV infection, we do not know whether you will benefit more from starting HIV medicines **EARLY** or taking them at the **DEFERRED** time**.** This study will help us learn how to treat future patients with HIV.

WHAT CHOICES DO YOU HAVE OTHER THAN BEING IN THIS STUDY?
You do not have to join this research study if you do not want to. If you join, you can quit at any time. If you choose not to join or to quit, it will not affect your regular medical care. If you decide not to join, please talk with your doctor about whether or not to take HIV medicines.

CAN YOUR STUDY PARTICIPATION BE STOPPED EVEN IF YOU DON'T AGREE?
The study doctor can take you out of this study if continuing in the study would harm you, if you go to prison, or if the study is stopped by the primary funder, the sponsor, the INSIGHT Executive Committee, review committees (IRB/REC) or government authorities. Once a year, an expert group will review the study and will recommend stopping it if the risk is higher than expected in either the **DEFERRED** or **EARLY** group.

WHAT WILL WE PAY FOR?

We will pay for the services, lab work, and other tests that are part of this study but not part of your regular care. During the study, your HIV medicines will either be paid for through your insurance company or national program or the study will provide them.

WHAT WILL HAPPEN AT THE END OF THE STUDY?
If you stop participating or the study ends, you and your doctor will decide whether you should take HIV medicines. If you stop early, the study will not be able to continue to provide your medicines. If you finish the study and we are providing your HIV medicines, we will continue to provide them for up to 6 months while you and your doctor find another way to get your medicines.

WHO WILL BE ABLE TO SEE YOUR MEDICAL INFORMATION?
We will protect the privacy of your medical information as much as legally possible, and release your records only with your written permission. We will label your study records with a code number and your initials, and you will not be identified in any publications about this research. However, your records may be seen by:

- People in the US government agencies that fund or oversee this research, for example, the U.S. National Institutes of Health (NIH).
- Study monitors who make sure the study is being conducted correctly.
- Independent groups (IRB/REC) that make sure the study is ethically acceptable.

[The following paragraph is for US sites only]
"We have a Certificate of Confidentiality from the US Government. This means that law enforcement officers, the courts, and others cannot force us to give them information about you. However, this does not prevent the study team from taking appropriate steps to prevent serious harm to you or to others.

WHAT IF YOU ARE INJURED AS PART OF THE STUDY?
We will provide treatment right away if you are hurt because of the research. The costs may be charged to you or your insurance company. We will give you information about where you can get additional treatment. You do not give up any of your legal rights by signing this form.

Site Instruction:
If the information is not correct for your country/site, please revise the information to inform the subject of the following: 1. what treatment will be provided to the subject; 2. who will pay for the treatment; 3. if there is any plan for compensation for research-related injury issues, such as lost wages, etc.

WHO CAN YOU TALK TO ABOUT THIS STUDY?
Please contact (*site PI and contact information*) if you have any questions or concerns about this research study or contact *(name and contact info)* if you have concerns about your rights as a research participant or you are injured as part of this study.

SIGNATURE PAGE FOR CONSENT TO PARTICIPATE IN START MAIN STUDY

If you have read the consent form or had the information explained to you, and you agree to join the START study, please sign below.

____________________ Participant's name (typed or printed)	____________________ Participant's signature	__________ Date
	**OR**	
____________________ Participant's legal guardian or representative	____________________ Legal guardian's signature	__________ Date

____________________ Witness's name (typed or printed) — ____________________ Witness's signature — __________ Date

NOTE: This consent form with the original signatures MUST be retained on file by the principal investigator. A copy must be given to the participant. A copy should be placed in the participant's medical record, if applicable.

A witness to the participant's signature is strongly encouraged.

APPENDIX B: SUMMARY OF BACKGROUND DATA FOR START DESIGN

CD4+ Cell Count, HIV RNA Level, and Risk of AIDS
Findings from the Multicenter AIDS Cohort Study (MACS) established that single measurements of HIV RNA and CD4+ count were important determinants of AIDS or death and that HIV RNA level predicted the rate of CD4+ decline. Data from larger epidemiological studies like the CASCADE Collaboration extended these findings by estimating 6-month risk of AIDS according to current CD4+ and HIV RNA levels. Predicted 6-month risk of AIDS among untreated patients aged 45 years with a CD4+ count of 500 cells/mm^3 ranged from 0.5% (HIV RNA = 3000 copies/mL) to 1.9% (HIV RNA = 300,000 copies/mL). Likewise, for patients in the same age group with an HIV RNA level of 100,000 copies/mL, the predicted 6-month risk of AIDS ranged from 1.0% (CD4+ = 350 cells/mm^3) to 0.5% (CD4+ = 500 cells/mm^3). Both current CD4+ count and current HIV RNA level were significant determinants of 6-month risk of AIDS.[3]

Other studies have provided evidence of a graded relationship between latest (or current) levels of CD4+ count and risk of AIDS diseases even among ART-naïve patients with CD4+ cell count ≥ 350 cells/mm^3.[3,102,103,104,105] Data for 17,609 ART-naïve individuals from the United Kingdom Collaborative HIV Cohort Study (UK CHIC) suggest that the graded relationship between AIDS or death and CD4+ count is evident among those with latest CD4+ cell counts ≥ 500/mm^3. In that cohort, estimates of absolute rates of AIDS or death are 24.5 (95% CI: 21.1 – 27.9), 15.5 (95% CI: 12.2 – 18.8) and 9.7 (95% CI: 7.0 – 12.4) per 1,000 person-years for patients with latest CD4+ count 350-499, 500-649 and ≥ 650/mm^3, respectively.

The risks for these groups with CD4+ counts above the current threshold for initiating ART are not negligible for individuals for whom there is the realistic hope of living close to a normal lifespan with use of ART. Based on these data, it is reasonable to hypothesize that for those with a CD4+ count > 500 cells/mm^3 (the target population for START), early initiation of ART will lead to a reduced risk of AIDS as compared to deferral of ART to CD4+ counts < 350 cells/mm^3 where AIDS risk is increased.

Furthermore, for a given CD4+ count, the risk of AIDS or death is lower in patients who have started ART than in those who are ART-naïve. Among individuals in the UK CHIC cohort, the corresponding rates of AIDS or death for those who started ART are 13.8 (95% CI: 11.7-15.9), 10.0 (95% CI: 7.8 – 12.2) and 9.1 (95% CI: 7.0-11.2) per 1,000 person-years for patients with latest CD4+ cell counts of 350-499, 500-649 and ≥ 650/mm^3, respectively. Overall, for those in the UK CHIC cohort, there was a reduced risk of AIDS or death for those on ART compared to those who were not (HR=0.58; 95% CI: 0.51-0.68) after adjustment for current CD4+ count (personal communication, Andrew Phillips). This reduced risk with ART was also observed in a EuroSIDA investigation. The lower risk of AIDS at high CD4+ counts among those on ART is also consistent with the finding from the MACS that AIDS events that occurred at higher CD4+ levels were associated with higher viral load levels.[106]

In the SMART trial, most (~80%) patient-time during follow-up was spent at CD4+ cell counts ≥ 350/mm^3.[6,107] Despite the relatively high CD4+ counts in the SMART study, there was a highly significant increased risk of AIDS or death in the patients assigned episodic ART (the DC group) compared to the viral suppression (VS) group (HR=2.6; 95% CI: 1.9-3.7). The difference in risk between the DC and VS groups was greater for fatal or non-fatal AIDS events (HR=3.6; 95% CI: 2.2-5.5) than for AIDS or death from any cause. The greater risk of AIDS or death in the DC compared to the VS group was explained in part by their lower CD4+ counts and higher HIV-RNA levels during follow-up.

These data suggest, but do not prove, that initiation of ART might reduce the rate of AIDS or death by an amount greater than would be predicted by the CD4+ cell count rise alone. The lower rates of AIDS or death on ART may be related to the lower viral load and the resulting reduction in immune activation,[108,109] other factors such as qualitative aspects of immunodeficiency not encapsulated in the CD4+ count,[110] and/or immunosuppression.[111] The effects of generalized immune activation and dysfunction induced by HIV infection are not well understood, and it is possible that these factors could be related to the development of non-AIDS events as well as AIDS events.

Relationship of CD4+ Count and Risk of Non-AIDS Events

While the potential reduction in risk of AIDS events is an important motivation for a trial of early ART, serious morbidity and mortality among patients with CD4+ count greater than 350 cells/mm^3 is dominated by conditions other than AIDS.[112,113,114,115,116,117] Until recently, non-AIDS conditions have been associated with other established risk factors and use of ART, but not HIV. Lau et al have found that in patients with CD4+ count > 200 cells/mm^3, risk of death from non-AIDS causes is greater than risk of death from AIDS causes.[118] The difference is more marked the higher the CD4+ count. Consistent with these data, of the 85 deaths that occurred in SMART, only 7 (8%) were from AIDS diseases. The risk of experiencing a pre-defined composite endpoint consisting of cardiovascular, hepatic, or renal disease outcomes was higher in the DC group compared with the VS group -- HR (DC/VS) = 1.7 (95% CI: 1.1-2.5).[6] The HR of non-AIDS death was 1.9 (95% CI: 1.1 – 2.9).[107] Further evidence that untreated HIV may result in an increased risk of death from non-AIDS diseases comes from EuroSIDA. In that large epidemiological investigation, the rate of non-AIDS-related death declined over the calendar time period corresponding to the introduction of effective ART across Europe.[115,119]

In D:A:D, the rates of death from hepatic causes, non-AIDS-defining malignancies[120] and deaths from non-AIDS causes were higher among those with lower levels of latest CD4+ cell counts. These relationships appear to be present even in those with a CD4+ cell count ≥ 350 cells/mm^3. Thus, the higher risk is not limited to those with very severe immunodeficiency. Similar findings have been reported from the CASCADE cohort, where cause-specific relative hazards per 100 /mm^3 higher CD4+ count of 0.89 (95% CI: 0.85 – 0.94) for risk of death from non-AIDS malignancy and 0.89 (95% CI: 0.83 - 0.92) for death from hepatic causes have been reported.[116] These relationships of risk

with CD4+ count are not so marked as for AIDS diseases, e.g., HR for death from AIDS is 0.76 (95% CI: 0.74 – 0.77) in CASCADE, but nevertheless are appreciable and attain high levels of statistical significance. Similarly, in the FIRST study, a study of initial ART in 1,397 patients, a 100-cell higher CD4+ cell count was associated with a 0.56 relative hazard for AIDS events (95% CI: 0.50-0.62) and a relative hazard of 0.86 (95% CI: 0.77-0.96) for non-AIDS morbidity and mortality.[121]

Cancer incidence has also been found to be increased in HIV-uninfected, immunosuppressed patients after kidney transplant.[122] A recent meta-analysis of HIV/AIDS studies and transplant studies concluded that both populations had an increased risk of many cancers and risk was similar.[123] Similarly, a report from the large Adult and Adolescent Spectrum of Disease and HIV Outpatient Study concluded that the incidence of many types of non-AIDS defining cancer were higher in HIV-infected persons compared to the United States general population.[124] Further, risk of lung cancer has been found to be higher in HIV infected, compared with uninfected, individuals even after adjustment for smoking.[125,126,127,128] A large study in U.S. veterans reported that hepatocellular carcinoma and non-Hodgkin's lymphoma risk was greater in HIV-positive than HIV-negative veterans. For hepatocellular carcinoma, the higher risk appears to be explained in part by co-infection with hepatitis C and alcohol abuse/dependence.[129] In SMART, the rate of non-AIDS malignancies was 30% higher among DC as compared to VS participants but this increased hazard was not significant (HR=1.3; 95% CI:0.7-2.1).[7]

Possible Positive and Negative Effects of ART on Non-AIDS Diseases
Liver disease has been reported to be the most common non-AIDS cause of death in HIV-infected patients receiving ART.[11] The balance in risks between known increased risk induced by some antiretroviral drugs[130,131] and the decreased risk of progressive liver diseases that may be associated with a reduction in immunodeficiency is not clear. While the impact of co-infection with hepatitis B and hepatitis C viruses on this risk/benefit is also unclear, data from SMART indicate that the potential for benefit is great. Risk of non-AIDS death in SMART was three- to fourfold higher among co-infected patients.[132]

The risk/benefit of early ART for CVD is also uncertain. In the large D:A:D cohort, an increased risk of myocardial infarction with increasing cumulative use of protease inhibitors was found (relative rate of 1.16 per year of protease inhibitor exposure).[16] This increased risk was estimated with high precision due to the 345 myocardial infarctions observed (95% CI: 1.10-1.23). Some of this effect appears to be explained by lipid levels. In D:A:D, the relative rate of myocardial infarction per year of protease inhibitor exposure was reduced to 1.10 (95% CI: 1.04-1.18) after adjustment for lipids. An increased risk of myocardial infarction or CVD associated with protease inhibitors has also been reported in other cohort studies.[133,134] Likewise, in two studies that compared HIV-infected individuals with the general population, myocardial infarction incidence was higher among those who were HIV infected.[135,136] In one of these studies, the increased risk was associated with protease inhibitors. The SMART and D:A:D group have also reported an increase risk of myocardial infarction associated wit use of abacavir.[17,18]

In SMART, there was a higher risk of CVD in the DC compared to the VS arm that was of borderline statistical significance (HR= 1.6; 95% CI 1.0 – 2.5; p=0.05). There is no evidence to suggest that this is related to the generally lower CD4+ counts in these patients. Consistent with this, the current CD4+ cell count does not show a strong association with risk of myocardial infarction in the D:A:D study. The higher risk in the DC arm in SMART could relate to changes in lipid profile resulting from stopping ART as there was an associated marked lowering in HDL cholesterol as well as in LDL cholesterol, resulting in an unfavorable total cholesterol-to-HDL ratio.[137] HDL cholesterol levels are reduced by HIV infection[138,139]. A possible mechanism involving impaired cholesterol efflux from macrophages by which the lower HDL cholesterol may increase risk of CVD in HIV-infected individuals has been described.[140] More generally, pro-atherogenic effects of HDL in the presence of inflammation have been the subject of several reviews.[141,142] This increased risk of CVD in the DC compared to the VS group was reduced when continuous ART was re-initiated (HR= 1.1; 95% CI: 0.7-1.8; p=00.64).

Impaired endothelial function may promote the development of atherosclerosis and other end-organ diseases through a number of mechanisms including its effects on the vascular wall, platelet and leukocyte adhesion, and coagulation.[143] Using stored specimens for SMART participants, four inflammatory and two coagulation markers were investigated as possible explanations for the increased risk of all-cause mortality, which was largely non-AIDS, and CVD in the DC compared to the VS group. Study entry levels of interluekin-6 (IL-6) and D-dimer were strongly related to all-cause mortality. Unadjusted ORs (highest versus lowest quartile) were 8.3 (95% CI 3.3-20.8; p<0.0001) and 12.4 (95% CI 4.2-37.0; p<0.0001), respectively. Furthermore, IL-6 and D-dimer levels increased one month after randomization by 30% and 16% in the DC group and by 0% and 5% in the VS group (p<0.0001 for both); the increase in the DC group was related to HIV-RNA levels at one month (p<0.0001).[144] IL-6 levels, an inflammatory marker produced in response to several factors including infection, are higher among HIV-infected than HIV-negative individuals.[145] D-dimer, a fibrin degradation product, has also been reported to be higher in HIV-infected as compared to HIV-negative individuals. In addition, D-dimer levels decreased in ART-naïve patients following the initiation of treatment.[146] HIV may increase risk of CVD and other end-organ diseases by activating inflammatory pathways in the vascular wall.[147]

HIV infection is associated with several types of renal dysfunction, including HIV-associated nephropathy (HIVAN), immune complex kidney disease and acute renal failure.[148,149] In a large cohort of women, the prevalence of proteinuria based on a urine dipstick examination was higher among women with higher HIV RNA levels and lower CD4+ count (≤ 200 versus > 200 cells/mm^3). Further, proteinuria was associated with an increased risk of a doubling of creatinine levels during follow-up.[150] A recent report in HIV-infected South Africans suggests that there may be a direct effect of HIV on renal disease as the prevalence of HIVAN was high among untreated individuals with HIV infection who did not have other risk factors for renal disease.[151] HIVAN is more commonly diagnosed in patients of Black African ethnic origin and appears to have

declined since the introduction of more potent ART regimens.[149,152,153,154] The impact of ART on renal disease other than HIVAN is less clear.[155] This may be due to associations with nephrotoxicity of some antiretroviral medications and with drugs used to prevent or treat complications of HIV infection.[148] Taken together, these studies indicate that HIV may increase the risk of renal disease. Whether early use of ART may prevent disease or slow progression is uncertain.

In summary, a clear picture on the likely risk/benefits of early ART in terms of hepatic, cardiovascular and renal disease does not emerge when considering together the data from epidemiological studies, laboratory studies, and SMART. A trial should provide definitive information on these risks and benefits.

CD4+ Cell Count and HIV RNA Response to ART

The majority of adults and children initiating ART now achieve a viral load < 50 copies/mL by 12 months.[156,157,158] Since the introduction of combination ART, this percentage has increased over time to a level (nearly 90% in some cohorts) at which future improvements will be difficult to achieve. On average, among patients achieving virologic suppression, there is a rapid rise in CD4+ cell count in the first several months of treatment followed by a continued slower rise.[159,160] With continued suppression of HIV RNA levels, CD4+ cell counts on average approach normal levels with long-term treatment.[161] However, achievement of normal levels likely requires several additional years for those starting ART with CD4+ counts < 350 compared to to > 350 cells/mm^3. In one report, median CD4+ counts after 7 years of uninterrupted ART were 660, 780, and 870 cells/mm^3, for those starting ART with counts 200-350, 350-500, and > 500 cells/mm^3, respectively.[162] Thus, if there is a continuum of risk between CD4+ count and AIDS and non-AIDS diseases as the epidemiological data indicate, patients who start ART at lower CD4+ counts will spend more time in CD4+ categories associated with a greater risk of morbidity and mortality. Further, about 15-20% of patients do not achieve a good immunological response (at least 25 cells) even with viral suppression after the first 6-9 months of ART,[163,164,165] and this poor immunologic response is associated with an increased risk of AIDS or death.[164,166] The consequences of this poor response on risk of AIDS and non-AIDS would be expected to be greater among patients who start ART at lower CD4+ counts. In addition, the immune response to immunization has been shown to be related to nadir CD4+ count, suggesting that immune competence may be compromised in those who defer ART to < 350 cells/mm^3.[167]

The CD4+ cell count response to different ART regimens is similar even though the virologic response varies. In three trials of protease inhibitor- and NNRTI-based regimens, CD4+ cell count increases were similar for the treatment groups even though NNRTI-based regimens (largely efavirenz) were superior in terms of virologic response to protease inhibitor-based regimens (largely nelfinavir and ritonavir-boosted regimens).[168,169,170,171] Similarly, a trial of lopinavir-ritonavir versus nelfinavir found that patients assigned lopinavir-ritonavir had a superior virologic response but a similar immunologic response as compared to patients assigned a nelfinavir-based regimen.[172] A recent review also noted that the recovery of CD4+ cell counts following the initiation

of ART appears independent of ART regimen but that comparisons over the long term were complicated by frequent changes in ART.[173]

These data indicate that a strategy of initiating ART at > 500 as compared to < 350 cells/mm^3 is likely to result in large CD4+ cell count differences between treatment groups over many years and beyond the planned duration of the study. During the initial years of follow-up, CD4+ cell counts will increase in the early ART group on average and decline in the deferred ART group. Even after those in the deferred group initiate ART, it will likely take several years to approach the average count achieved for a comparable patient (similar baseline count) in the early ART group. Also, the consequences of not achieving a robust immune response will be greater in terms of CD4+ count among those in the deferred ART arm.

Data indicate that new regimens that might be available to the deferred arm, and not the immediate ART arm, are unlikely to result in large differences in viral suppression or CD4+ cell count recovery compared with existing regimens. With the ART strategies to be studied, large differences in ART exposure, HIV RNA levels, and CD4+ count are expected over the entire follow-up period. These are ideal conditions to understand the risk and benefits of beginning ART at CD4+ counts > 500 versus waiting until the CD4+ declines to < 350 cells/mm^3.

CD4+ Decline among ART-Naïve Individuals
The CD4+ cell counts that define patient eligibility and the rate of CD4+ decline among those enrolled and randomized to the deferred group are important design parameters. Together, the entry CD4+ and rate of decline of CD4+ during follow-up define the average period of time the randomized groups differ with respect to use of ART. During this period of decline in CD4+ count among the deferred group before ART is initiated, it is hypothesized that risk of AIDS and non-AIDS conditions will increase. During this same time period, for the immediate ART group, it is hypothesized that risk of AIDS and non-AIDS diseases will decrease as a consequence of using ART that increases CD4+ cell count and suppresses HIV RNA levels. Any adverse effects of ART would also be more evident during this time period in the early ART group than later in follow-up when a larger percentage of those assigned to the deferred arm are also taking ART.

CD4+ declines among naïve patients depend on HIV RNA levels and are highly variable.[20] For example, a recent analysis of two cohorts indicated that the annual rate of decline was 55.9 cells (95% CI: 47.3-64.5) for those with HIV RNA levels at baseline of 10,001-40,000 copies/mL and 77.7 cells (95% CI: 68.2-87.3) for those with HIV RNA levels > 40,000 copies/mL.[21] This level and variability of CD4+ decline has been observed in other cohorts[174,175] and the decline has been found to be greater among older patients.[175] Of particular relevance to the proposed design, in the UK CHIC study, among patients with CD4+ cell count between 500 and 650 cells/mm^3 and with a median HIV RNA level of 15,800 copies/mL, the median time to use of ART or a CD4+ cell count < 350 cells/mm^3 was 2.5 years.

With consideration of the variability in the rate of decline, current guidelines indicate that the CD4+ cell count is usually the most important consideration in decisions to initiate ART and that counts among ART-naïve patients should be determined every 3 to 6 months.[1] In START, CD4+ cell count will be monitored at least every 4 months for both the deferred and early ART groups.

Risk Factors for Non-AIDS Conditions

The etiology of each of the non-AIDS conditions – CVD, renal disease, liver disease, and non-AIDS-defining cancers – is multifactorial. For each disease, there are established risk factors, many of which are modifiable. HIV and ART may be additional risk factors for these diseases.

Older age is associated with an increased risk of each of these diseases. As HIV populations with access to ART age, these events will increase in absolute numbers. Smoking is an established risk factor for many diseases including cardiovascular, renal and liver disease and many cancers.[176] For CVD, the major risk factors are blood cholesterol level, blood pressure, smoking, overweight/obesity, diets high in fat and cholesterol, and diabetes mellitus.[177,178] These risk factors are additive in their effects on CVD risk, and for blood pressure and blood cholesterol, risk of a cardiovascular event is not restricted to those with hypertension or hypercholesterolemia. Risk equations for predicting long-term risk of CVD morbidity and mortality have been developed taking into account actual levels of blood pressure and blood cholesterol.[179]

Risk factors for renal disease are similar to those for CVD. Blood pressure and diabetes mellitus are major risk factors. Smoking and elevated blood cholesterol are also associated with an increased risk of renal disease.[180] African-Americans have a greater risk of kidney disease than other race groups.[181] Traces of protein on a casual urine dipstick and above average creatinine levels (e.g., 1.5-2.0 mg/dl) have been associated with long-term risk of ESRD.[182] The National Kidney Foundation in the United States has adopted a new classification scheme for chronic kidney disease as an estimated glomerular filtration rate (eGFR) < 60 mL/min per 1.73 m^2 or proteinuria.[183] Creatinine will be measured in START at baseline and all follow-up examinations in the first year and annually afterward in order to estimate GFR. The simplified Modification of Diet in Renal Disease equation will be used.[30]

Chronic hepatitis C infection is a major risk for cirrhosis, hepatocellular carcinoma, and death from liver disease.[184,185] The association between co-infection and risk of AIDS or death has not been consistent in different studies.[185,186,187] In SMART, approximately 15% of patients were co-infected with hepatitis C.[6] Risk of non-AIDS death was three- to fourfold higher among co-infected patients and the majority of these patients were co-infected with hepatitis C.[132] Chronic hepatitis B infection is associated with an increased risk of death from liver disease.[10] However, co-infection with hepatitis B virus is expected to be less common in the target population for START because of the location of sites.

If early ART is effective in reducing risk of these diseases, the public health impact may be much greater among individuals with established risk factors for each disease because a given proportional reduction in risk will have a much greater impact on absolute excess risk among individuals at higher risk. For this reason, subgroup hypotheses have been formulated that take into account risk of non-AIDS diseases.

Potential Loss of Future Drug Options with Early ART

As a consequence of incomplete HIV RNA level suppression with ART, replication in the presence of strong selective drug pressure occurs and resistance develops. The resistance that develops to a specific drug can confer cross-resistance to other drugs within the class.[188] While a second potent ART regimen can usually be constructed following virologic failure on the initial regimen, until recently subsequent durable regimens were more difficult to define. With the early potent ART regimens used, toxicities and poor adherence were common reasons for changing ART regimens.[189] Current ART is less toxic and more convenient to take, and it has proved to be highly successful in durably suppressing HIV.[190,191] Emergence of three-class resistance and exhaustion of all available active drugs has also proved to be a slow process.[192]

Nevertheless, ART is life-long, and excellent adherence is critical to reducing the risk of resistance. This is a major reason why treatment guidelines emphasize patient readiness.[1] This will also be an important consideration for the START study – patients and clinicians must be prepared to initiate ART immediately if randomized to that arm. Since drug therapy is needed in order to select for resistance, the risk of developing resistance will be higher initially in patients randomized to early ART initiation compared with those deferring use of ART.

Effect of ART on HIV Transmission

An early study suggested that zidovudine reduced HIV transmission among serodiscordant couples.[193] Two studies in Africa found an association between plasma HIV RNA level and transmission risk.[25,26] In the Rakai study of 415 serodiscordant couples in which transmission risk increased with HIV RNA level of the infected partner, no transmission events occurred in those couples in which the infected partner had a plasma HIV RNA level < 1500 copies/mL.[25] Models predict that ART could decrease HIV transmission.[194,195] This however, may depend in part on changes in HIV risk behaviors following the initiation of ART.

The association between ART usage and HIV transmission risk behavior was evaluated in a substudy of the SMART study. Overall, transmission risk behavior was similar among participants randomized to continuous ART versus episodic ART. However, in the relatively small subgroup of participants (n = 194) off ART at baseline (and therefore, similar to participants in the START study), randomization to start ART was associated with a decrease in self-reported high-risk behavior (anal or vaginal sex without a condom, self-reported needle-sharing, or incident gonorrhea, chlamydia, or syphilis). These data suggest that earlier initiation of ART may decrease the risk of HIV transmission, both by decreasing viral load and by decreasing behaviors associated with HIV transmission.[196]

APPENDIX C: TIME AND EVENTS SCHEDULE

Requirement	Baseline (≤60 days **before** participant randomized unless noted below)	≤14 days **before** participant randomized	Follow-up visits in Y1				Follow-up visits **after** Y1 (every 4 months)	
			1	4	8	12	Annual (e.g. 24, 36, etc.)	Other q4 visits
Informed consent	X							
Demographics, including education	X							
Documentation of HIV infection[1]	X							
CD4+ cell count and CD4%	X[2]		X	X	X	X	X	X
Karnofsky score	X							
Serum or urine pregnancy test[3]		X[4]						
Targeted health history and clinical evaluation[5]	X		X	X	X	X	X	X
Nadir CD4+ cell count and CD4%, maximal HIV RNA documented in the medical record at any time in the past	X							
Up to 3 most recent additional CD4+ cell counts, CD4%s and HIV RNA levels available in the medical record	X		X	X	X	X	X	X

[1] By plasma HIV RNA viral load, a rapid HIV test or any licensed ELISA test; and confirmed by an ELISA, Western Blot, HIV culture, HIV antigen, HIV pro-viral DNA or a second antibody test by a method other than ELISA at any time prior to study entry

[2] Before randomization, two consecutive CD4+ cell count and % measurements are required at least 2 weeks apart, with the earlier occurring not more than 60 days before randomization.

[3] For women of child-bearing potential. Test must be done in the clinic and read by a clinician or laboratory technician.

[4] Also required anytime pregnancy is suspected or if a woman of child-bearing potential is being prescribed efavirenz or other Pregnancy Category D drug.

[5] To include weight, height, sitting blood pressure, pulse, and smoking status (smoking status at baseline and annually only). The following clinical events are reported as they occur: AIDS diagnoses (listed in Appendix E), non-AIDS events (listed in Appendix E), bacterial pneumonia, pulmonary embolism, deep vein thrombosis, new-onset diabetes mellitus, coronary artery disease requiring drug treatment, congestive heart failure, peripheral arterial disease, serious events, initiation of and changes of ART, and pregnancy outcomes.. In addition, for participants diagnosed with malignancy while on study, fresh frozen tissue or paraffin block, pathology slide(s) of the malignancy, and pre-treatment whole blood stored for future research will also be obtained, if possible.

Requirement	Baseline (≤60 days **before** participant randomized unless noted below)	≤14 days **before** participant randomized	Follow-up visits in Y1				Follow-up visits **after** Y1 (every 4 months)	
			1	4	8	12	Annual (e.g. 24, 36, etc.)	Other q4 visits
Findings from genotyping or other form of acceptable ART resistance testing, if available	X		X	X	X	X	X	X
Recording of selected concomitant medications	X					X	X	
Pregnancy history	X					X	X	
Quality of life assessment	X			X		X	X	
Use of alcohol and recreational drugs	X					X	X	
HIV transmission risk behaviors	X			X		X	X	
Health care utilization	X					X	X	
HIV RNA level	X		X	X	X	X	X	X
CBC: hemoglobin, hematocrit, white blood cell count (WBC) with differential and platelets	X					X	X	
CD8+ cell count and CD8%	X		X	X	X	X	X	X
Renal function measurement: serum creatinine	X		X	X	X	X	X	
Liver function measurements: ALT, AST, alkaline phosphatase, total bilirubin and albumin	X					X	X	
Glucose	X					X	X	
Lipids: total cholesterol, LDL, HDL, triglycerides	X					X	X	
Dipstick urinalysis for protein	X		X	X	X	X	X	

Requirement	Baseline (≤60 days **before** participant randomized unless noted below)	≤14 days **before** participant randomized	Follow-up visits in Y1				Follow-up visits **after** Y1 (every 4 months)	
			1	4	8	12	Annual (e.g. 24, 36, etc.)	Other q4 visits
Hepatitis B and C: hepatitis B surface antigen, core antibody, surface antibody; hepatitis C antibody; and, if available, hepatitis C genotype and viral load	X[6]							
Resting ECG[7]	X					X	X	
ART regimen which participant will start if randomized to Early arm	X							
Current ART regimen			X	X	X	X	X	X
Self-reported adherence to ART			X	X	X	X	X	X
Stored plasma for future HIV-related research (separate consent)[8]	X		X	X	X	X	X	X
Stored urine for future HIV-related research (separate consent)	X		X	X	X	X	X	
Extended demographics (Neurology substudy)	X							
Neuropsychological tests (Neurology substudy)	X			X	X	X	X	
CES-D Depression Scale (Neurology substudy)	X			X	X	X	X	
Self-reported comprehension of study requirements (at participating sites)	X							

[6] Documentation of positive tests for hepatitis from any time in the past or of negative tests for hepatitis within the past six months is acceptable.
[7] At sites with a study-supplied ECG machine and a certified technician.
[8] Plasma for storage will also be collected each time the ART regimen is switched or stopped due to an elevated HIV RNA (or presence of resistance mutations). The sample will be used for HIV resistance testing to be done in batch mode at a later time.

Requirement	Baseline (≤60 days **before** participant randomized unless noted below)	≤14 days **before** participant randomized	Follow-up visits in Y1				Follow-up visits **after** Y1 (every 4 months)	
			1	4	8	12	Annual (e.g. 24, 36, etc.)	Other q4 visits
Stored whole blood for future genomics research (separate consent)	X[9]							

[9] It is strongly encouraged that consent to participate in the Genomics substudy is given prior to randomization in the main study and a blood sample provided shortly after the consent. Participants may, however, consent to participate in the Genomics substudy at any time after randomization in the main study.

APPENDIX D: INSIGHT EXECUTIVE COMMITTEE AND START PROTOCOL TEAM

The **INSIGHT Executive Committee** is responsible for the governance of the network and is comprised of leading experts in the design, administration, conduct, and analysis of HIV clinical trials. The Executive Committee includes representatives from the community and different scientific and administrative disciplines. It consists of the following members:

- James D. Neaton (Chair)
- Donald Abrams
- Abdel Babiker
- John Baxter
- Calvin Cohen
- David Cohn
- Simon Collins
- David Cooper
- Janet Darbyshire
- Wafaa El-Sadr
- Sean Emery
- Fred Gordin
- Karin Klingman
- H. Clifford Lane
- Gregg Larson
- Marcelo Losso
- Jens Lundgren
- Jeff Nadler
- Andrew Phillips
- Jo Watson

The **INSIGHT START Protocol Team** will oversee the implementation of the START study. The Protocol Team includes representatives from the community, from industry collaborators, and from different scientific and administrative disciplines. Four members of the INSIGHT Executive Committee will serve as co-chairs. Members are:

- Co-Chairs: Abdel Babiker, Sean Emery, Fred Gordin, Jens Lundgren
- Blinded statistician: Abdel Babiker
- Unblinded statisticians: Birgit Grund, Andrew Phillips, Shweta Sharma
- NIAID statistician: Michael Proschan
- Division of AIDS Medical Officer: Karin Klingman
- Division of AIDS Pharmacist: Ana Martinez
- Laboratory Representative: John Baxter
- ICC clinicians: Jose Arribas, Waldo Belloso, David Cooper, Gerd Fätkenheuer, Bernard Hirschel, Sally Hodder, Margaret Johnson, Mauro Schechter
- ICC representatives: Daniela Gey, Nick Paton, Sarah Pett, Michael Vjecha

- Leadership CORE representatives: Gregg Larson, Eileen Denning
- Community representatives: David Munroe, Siegfried Schwarze
- Pharmaceutical company representatives: Ron Falcon (Tibotec); Sandy Griffith (GlaxoSmithKline); Kristy Grimm (Bristol-Myers Squibb); François Lilienthal (Merck); Michael Norton (Abbott); Jim Rooney (Gilead)
- Other funders: Jean-Michel Molina and Bruno Hoen (ANRS); Gerd Fätkenheuer (BMBF); Richard Davey, Jr. (NIH Clinical Center); Brian Agan and Scott Wegner (Tri-Service AIDS Clinical Consortium/TACC, funded by NIAID/NIH).
- Other experts (including INSIGHT Interest Group representatives and substudy representatives not identified elsewhere): Jason Baker, Bruce Brew, Bill Burman, Richard Davey, Matthew Dolan, Greg Dore, Daniel Duprez, Ezekiel Emanuel, Christine Grady, Alan Landay, Bruno Ledergerber, Ron Mitsuyasu, Amanda Mocroft, Deenan Pillay, Richard Price, Peter Reiss, Kevin Robertson, Jürgen Rockstroh, Michael Ross, Amalio Telenti, Edwina Wright

APPENDIX E: PRIMARY ENDPOINT DEFINITION

The primary composite endpoint for START is non-fatal serious AIDS events (or "AIDS*"), non-fatal serious non-AIDS (or "non-AIDS") events, and death from any cause. It includes the following components:

- Fatal AIDS or non-fatal AIDS* events
  These include opportunistic events consistent with the 1993 CDC expanded surveillance definition plus additional events associated with immunosuppression in the participant population targeted for enrollment. Esophageal candidiasis and chronic *Herpes simplex* infection will only be counted in the primary endpoint if fatal events.

  AIDS* events include:
  - Aspergillosis (invasive)
  - Bartonellosis
  - Candidiasis of the bronchi, trachea, or lungs
  - Invasive cervical cancer
  - Chagas disease (American trypanosomiasis) of the central nervous system (CNS)
  - Cytomegalovirus virus (CMV) disease (radiculomyelitis, meningoencephalitis, or other disease)
  - CMV retinitis
  - Extrapulmonary or disseminated coccidioidomycosis
  - Cryptosporidiosis with diarrhea > 1 month
  - Cryptococcosis, meningitis or extrapulmonary
  - HIV-related encephalopathy, including AIDS Dementia Complex
  - Disseminated *Herpes zoster*
  - Extrapulmonary or disseminated histoplasmosis
  - Isosporiasis with diarrhea > 1 month
  - Kaposi's sarcoma, mucocutaneous or visceral
  - Leishmaniasis (visceral)
  - Hodgkin's lymphoma
  - Non-Hodgkin's lymphoma, all cell types
  - Primary lymphoma of the brain
  - Tuberculosis, pulmonary and extrapulmonary
  - Microsporidiosis with diarrhea > 1 month
  - *Mycobacterium avium* complex (MAC), disseminated
  - Other nontuberculous species or unidentified species of *Mycobacterium*, disseminated
  - Nocardiosis
  - Penicilliosis, disseminated
  - Extrapulmonary *Pneumocystis jirovecii*
  - *Pneumocystis jirovecii* pneumonia
  - Recurrent bacterial pneumonia (2 episodes within 12 months)

- Progressive multifocal leukoencephalopathy (PML)
- *Rhodococcus equi* disease
- Recurrent *Salmonella* septicemia (2 episodes within 12 months)
- Toxoplasmosis of the brain
- Wasting syndrome due to HIV

AIDS events include:
- All conditions defined as AIDS* above
- Esophageal candidiasis
- Chronic *Herpes simplex*

- Non-fatal serious non-AIDS events ("non-AIDS")
  - o Cardiovascular disease (CVD) (myocardial infarction, stroke, coronary revascularization)
  - o End-stage renal disease (ESRD) (initiation of dialysis, renal transplantation)
  - o Decompensated liver disease
  - o Non-AIDS-defining cancers (excluding basal and squamous cell skin cancers)

- Deaths not attributable to AIDS

The INSIGHT Endpoint Review Committee (ERC) has established objective criteria for each event and its level of diagnostic certainty. These criteria are given in the *START Protocol Instructions Manual.* The ERC is responsible for reviewing each reported event to determine the level of diagnostic certainty. Events that are judged as confirmed or probable will be included in the primary analysis.

APPENDIX F: GUIDELINES FOR RESISTANCE TESTING

When initiating ART in either treatment group, resistance testing may be helpful to guide therapy; national or local treatment guidelines should be consulted.[1, 197, 198] Resistance testing can also be used to guide treatment changes in participants experiencing virologic failure. The following provides background information and general comments regarding the use of resistance testing in managing ART.

There are two types of resistance tests: genotypic and phenotypic assays. A genotype detects resistance mutations to relevant viral genes and an interpretive algorithm is used to predict drug susceptibility. A phenotype measures the virus's ability to replicate in the presence of different drugs, with susceptibility reported as an IC_{50} fold change compared to a wild-type control. The genotypic assay is generally more sensitive in detecting early evidence of drug resistance, has a more rapid turn around time, and is usually less expensive than a phenotype.

Transmitted resistance, defined as resistance to at least one antiretroviral drug, has been reported to occur in 6-16% of newly infected patients from surveillance studies in the U.S. and Europe.[4] Resistance mutations may persist for prolonged periods in untreated chronically infected patients. Persistence of transmitted NNRTI resistance mutations is likely due to these mutations having little effect on viral fitness.

Studies suggest that suboptimal virologic responses may occur in patients with baseline resistance mutations.[1] Recent data using highly sensitive resistance assays have demonstrated that for NNRTI-based regimens, the presence of NNRTI resistance prior to starting ART is predictive of virologic failure. Resistance testing in naive patients has also been demonstrated to be cost-effective based on a prevalence of at least 5% in an ART-naïve population, given specific assumptions about regimen failure rates and its consequences to future regimen outcomes.[199]

Guidelines for resistance testing in treatment-naïve patients

The decision to perform resistance testing immediately prior to initiating ART should consider: (1) the regimen to be prescribed; (2) availability of previous resistance tests (e.g., closer to the time of seroconversion); and (3) risk of having transmitted drug resistance based on local data. Additionally, the following general points regarding baseline resistance testing should be considered:

- Resistance testing is desirable to guide initial treatment if the prevalence of transmitted resistance is > 5% in the population where the patient is believed to have been infected, though it is reasonable to note that relying on geography and estimates of time of seroconversion may be imprecise for several reasons including the mobility of populations.
- In treatment-naïve patients, a genotype is the preferred assay to detect transmitted resistance.
- Ideally, resistance testing should be performed early after the diagnosis of HIV to increase the likelihood of detecting resistance. If a resistance test was done previously, then that test can be used to guide initial treatment.

- There are settings where resistance testing may not be necessary prior to starting treatment. These include regions of very low prevalence of transmitted drug resistance, as well as a reliance on regimens that are likely to be minimally impacted by typical transmitted resistance patterns.

Guidelines for resistance testing in treatment-experienced patients
There are no restrictions on the use of resistance testing for guiding treatment changes. Prospective studies have shown a benefit with drug resistance testing in patients with virologic failure.

Clinicians should refer to current guidance on switching ART because of virologic failure or suboptimal virologic response.[1, 197, 198] Resistance testing is most helpful when performed while a patient is taking their failing regimen. A definitive resistance test result is more likely when the plasma viral load is > 1,000 copies/mL. In treatment-experienced patients, resistance testing is not as useful after patients have discontinued treatment, as drug resistant species may not be detected.

Resistance testing should not be the only consideration guiding treatment changes. Prior treatment history, adherence, drug intolerance, and pharmacokinetic issues should also be assessed. Furthermore, expert advice has been shown to be beneficial in patients with extensive prior ART experience and drug resistance.[1]

APPENDIX G: SAMPLE SIZE JUSTIFICATION

Sample size has been estimated using data from several sources including the CASCADE Collaboration and the UK CHIC Cohort. To calculate event rates in START, the rate of CD4+ count change while ART naïve and after initiation of ART was modelled using data from CASCADE. The observed CD4+ counts, which were modelled in the square root scale (suggested by Box-Cox), were assumed to differ from the individual's true (unobserved) values by random measurement errors representing intra-patient variability. Before ART initiation, a person's true square root CD4+ trajectory incorporates a linear decline with slope varying between individuals plus true fluctuations represented by a Brownian motion process.[200] A similar model was used after ART initiation, except that the trajectory incorporated two rates of increase one up to 1 year after ART initiation and the other after 1 year.

Event rates are assumed to be determined by current true CD4+ count but initiation of ART in the deferred treatment group is determined by the observed CD4+ count. At baseline, it was assumed that 70% of patients would have CD4+ counts between 501 and 600 cells/mm^3, 20% between 601 and 700, and the remaining 10% would have CD4+ counts > 700 cells/mm^3. The estimated model parameters were used to calculate the predicted (true) CD4+ counts corresponding to the observed values.
Using the estimated slopes for CD4+, computer simulations were performed to estimate time spent in different CD4+ categories. Event rates within CD4+ categories were estimated using data from the CASCADE Collaboration [3] and from the UK CHIC Cohort.[28] CD4+ was assumed to be measured every 4 months; repeated after a month if the value crossed the CD4+ threshold for initiation of ART in the deferred treatment group. To allow for non-adherence to the deferred ART strategy, it was assumed that 70% of patients would not initiate ART until CD4+ count dropped to below 350 cells/mm^3 and 30 % would initiate ART earlier – 10% before the CD4+ declined to 400 cells/mm^3 and 20% while the CD4+ was between 350 and 400 cells/mm^3.

Based on this model and the assumptions cited, the percentage of patients initiating ART in the deferred arm after 1 year is estimated as 13%; by the end of the study, 75% of patients in the deferred arm will have started ART.

The event rates from the cohort studies that were used for the simulations are shown in Tables G-1 and G-2 below.

Table G-1 summarizes event rates for ART-naïve participants from UK CHIC and CASCADE. Rates of AIDS or death from any cause (AIDS/death) can be computed for both cohorts. Rates for AIDS* (serious AIDS events including all AIDS deaths) can only be estimated from CASCADE. AIDS/death rates are similar for the two cohorts. Serious AIDS event rates are much lower because non-AIDS deaths represent a large fraction of the deaths in each CD4+ category.

Table G-1: Event Rates Pre-ART by Latest Observed CD4+ Count

Endpoint	Cohort	CD4+	Person-years	Events	Rate
AIDS or death	UK-CHIC	<200	2,449	794	32.4
		200-349	7,062	349	4.9
		350-499	8,643	212	2.5
		500-649	5,704	89	1.6
		≥ 650	5,464	53	1.0
	CASCADE	<200	1,592	448	28.1
		200-349	5,148	235	4.6
		350-499	8,242	172	2.1
		≥ 500	15,277	160	1.0
AIDS*	CASCADE	<200	1,604	371	23.1
		200-349	5,136	172	3.3
		350-499	8,235	111	1.3
		≥500	15,260	95	0.6
Non-AIDS death	CASCADE	<200	1,557	83	5.3
		200-349	5,086	42	0.8
		350-499	8,232	46	0.6
		≥ 500	15,295	48	0.3
All-cause death	CASCADE	<200	1,557	147	9.4
		200-349	5,086	47	0.9
		350-499	8,232	50	0.6
		≥ 500	15,295	57	0.4

Table G-2 summarizes event rates following the initiation of ART for participants in the CASCADE study. Similar to the data for ART-naïve participants in Table 1 rates for AIDS* are much smaller than AIDS or death because non-AIDS deaths represent a large fraction of all deaths. This fraction increases with increasing CD4+ cell count.

Table G-2: CASCADE Event Rates after Starting ART by Latest Observed CD4+ Cell Count

	Latest CD4+ Cell Count (cells/mm^3)			
**Endpoint**	**<200**	**200-349**	**350-499**	**≥500**
AIDS or Death	10.7	2.2	1.3	0.6
AIDS*	8.2	1.7	0.6	0.3
Non-OD death	2.5	0.8	0.4	0.3
All-cause death	4.3	0.8	0.4	0.3

The early and deferred ART groups are to be compared in terms to time to first occurrence of any of the components of the composite primary endpoint. It is assumed that 5% of patients with primary outcome would have multiple events of both AIDS* and non-AIDS. Rates of non-AIDS morbidity were not available and the events in the cohorts used were not adjudicated. We assumed that the rate of non-fatal non-AIDS events was 4 times the death rate from non-AIDS causes resulting in an assumed ratio of non-AIDS to AIDS* events of 3.3. We also assumed that the percentage of accepted events after adjudication was 65% for AIDS* and 90% for non-AIDS events. The sensitivity of sample size to the ratio of non-fatal non-AIDS to non-AIDS death and to different adjudication rates are illustrated below in Table G-3. Sample size is shown assuming 90% power and a type 1 error of 0.05 (2-sided). Based on this table, the target number of events was set at 370. If 275 events are achieved instead of 370, power is approximately 0.80 to detect a hazard ratio of 0.714 at the 0.05 level of significance.

Table G-3: Sensitivity of Sample Size and the Event Target to Assumptions Concerning the Ratio of Non-AIDS to AIDS* Events and Event Adjudication Estimates

Ratio: Non-fatal Non-AIDS/Non-AIDS Deaths	% AIDS* Events Adjudicated	Composite Event Rate per 100 Person Years (Deferred Arm)	Hazard Ratio (average over follow-up)	Combined Sample Size	Event Target
3	65	2.40	0.706	4,244	351
4	65	2.81	0.712	3,822	369
5	65	3.23	0.717	3.472	383
3	75	2.49	0.700	3,900	333
4	75	2.91	0.707	3,545	353
5	75	3.33	0.712	3,245	367

APPENDIX H: REFERENCES ON INSIGHT WEBSITE

The INSIGHT website (**www.insight-trials.org**) will maintain updated links to the following documents referenced in the START protocol and to other information pertinent to the study:

- The *START Protocol Instructions Manual*
- The table "Antiretroviral Components Required for the Initial Regimen in START" with a current list of preferred antiretroviral regimens for use as initial therapy in START. Previous versions of the table with applicable dates will also be posted.
- Current product information on antiretroviral drugs used in START
- Information on antiretroviral drug availability and distribution from the INSIGHT Central ART Repository
- Panel on Clinical Practices for Treatment of HIV Infection. Guidelines for the use of antiretroviral agents in HIV-1 infected adults and adolescents.
- The DAIDS Table for Grading the Severity of Adult and Pediatric Adverse Events, Version 1.0, December 2004 (or "DAIDS AE Grading Table"), as applicable for START
- *Antiretroviral Pregnancy Registry* for voluntary reporting of pregnancies that occur during START
- INSIGHT Publications and Presentations Policy

APPENDIX I: LIST OF ACRONYMS

ACSR	AIDS Cancer Specimen Resource
ADS	Average Deficit Score
AE	Adverse Event
AIDS	Acquired Immunodeficiency Syndrome (see Appendix E)
AIDS*	Modified definition of AIDS (see Appendix E)
ALT	Alanine aminotransferase
ANRS	Agence Nationale de Recherches sur le SIDA et les Hépatites Virales
ART	Antiretroviral Therapy
AST	Aspartate Aminotransferase
AWP	Average Wholesale Price
BMBF	Bundesministerium für Bildung und Forschung (German Ministry)
BP	Blood Pressure
cART	Combination Antiretroviral Therapy
CASCADE	Concerted Action on Seroconversion to AIDS and Death in Europe
CBC	Complete Blood Ccount
CDC	Centers for Disease Control and Prevention (U.S.)
CES-D	Center for Epidemiologic Studies Depression Scale
CFR	Code of Federal Regulations (U.S.)
CI	Confidence Interval
CNS	Central Nervous System
CPE	CNS Penetration Score
CRF	Case Report Form
CSF	Cerebrospinal Fluid
CVD	Cardiovascular Disease
D:A:D	Data Collection for Adverse Events of Anti-HIV Drugs
DAIDS	The Division of AIDS, NIAID, NIH (U.S.)
DC	Drug Conservation (Arm in SMART Study)
DHHS	Department of Health and Human Services (U.S.)
DNA	Deoxyribonucleic Acid
DSMB	Data and Safety Monitoring Board
DVT	Deep Vein Thrombosis
EAE	Expedited Adverse Event
ECG	Electrocardiogram
eGFR	Estimated Glomerular Filtration Rate
ELISA	Enzyme-Linked Immunosorbent Assay
EMEA	European Medicines Agency
ERC	Endpoint Review Committee
ESPRIT	Evaluation of Subcutaneous Pro-leukin in a Randomized International Trial
ESRD	End-stage Renal Disease
EU	European Union
FDA	Food and Drug Administration (U.S.)
FIRST	Flexible Initial Antiretrovirus Suppressive Therapies
GCP	Good Clinical Practice

GEE	General Estimating Equations
GFR	Glomerular Filtration Rate
GID	Generated Identification Number
HAART	Highly Active Antiretroviral Therapy
HBM	Human Biological Material
HBV	Hepatitis B Virus
HCV	Hepatitis C Virus
HDL	High Density Lipoprotein
HIV	Human Immunodeficiency Virus Type 1
HIVAN	HIV-Associated Nephropathy
HLA	Human Leukocyte Antigen
HR	Hazard Ratio
HVLT-R	Hopkins Verbal Learning Test
ICC	International Coordinating Center (INSIGHT)
IEC	Institutional Ethics Committee
IL-2	Interleukin-2
INSIGHT	International Network for Strategic Initiatives in Global HIV Trials
IRB	Institutional Review Board
LDL	Low Density Lipoprotein
MACS	Multicenter AIDS Cohort Study
mL	Milliliter
mm	Millimeter
NCI	National Cancer Institute, NIH (U.S.)
NFL	Neurofilament Protein
NIAID	National Institute of Allergy and Infectious Diseases, NIH (U.S.)
NIH	National Institutes of Health (U.S.)
NIMH	National Institute of Mental Health, NIH (U.S.)
NINDS	National Institute of Neurological Disease and Stroke, NIH (U.S.)
NNRTI	Non-nucleoside Reverse Transcriptase Inhibitor
Non-AIDS	Serious Non-AIDS Conditions (see Appendix E)
NRTI	Nucleoside/Nucleotide Reverse Transcriptase Inhibitor
OHRP	Office for Human Research Protections (U.S.)
PHI	Primary HIV Infection
PHS	Public Health Service (U.S.)
PI	Protease Inhibitor
PID	Participant Identification Number
PIM	Protocol Instructions Manual
QNPZ	Quantitative Neurocognitive Performance Z Score
RNA	Ribonucleic Acid
SAE	Serious Adverse Event
SD	Standard Deviation
SDMC	Statistical and Data Management Center (INSIGHT)
SF-12	Medical Outcomes Study Short-Form-12 Item Survey
SMART	Strategies for Management of Antiretroviral Therapy
START	Strategic Timing of Antiretroviral Treatment
SUSAR	Suspected Unexpected Serious Adverse Reactions

TACC	Tri-Service AIDS Commission, Department of Defense (U.S.)
U.K. CHIC	United Kingdom Collaborative HIV Cohort Study
U.S.	United States of America
VS	Viral Suppression (Arm in SMART Study)
WAIS-III	Wechsler Adult Intelligence Scale-III
WBC	White Blood Cell Count
WHO	World Health Organization

REFERENCES

[1] Panel on Antiretroviral Guidelines for Adults and Adolescents. Guidelines for the use of antiretroviral agents in HIV-1 infected adults and adolescents. November 3, 2008. (http://AIDSinfo.nih.gov/ContentFiles/AdultandAdolescentGL.pdf).

[2] European AIDS Clinical Society (EACS). Guidelines for the clinical management and treatment of HIV infected adults in Europe. Version 2, December 2007 (http://www.eacs.eu/)

[3] CASCADE Collaboration. Short-term risk of AIDS according to current CD4 cell count and viral load in antiretroviral drug-naïve individuals and those treated in the nonotherapy era. *AIDS* 2004;18:51-58.

[4] Egger M, May M, Chene G, et al and the ART Cohort Collaboration. Prognosis of HIV-1 infected patients starting highly active antiretroviral therapy: a collaborative analysis of prospective studies. *Lancet* 2002;360:119-129.

[5] May M, Sterne JAC, Sabin C, et al and the ART Cohort Collaboration. Prognosis of HIV-1 infected patients after initiation of HAART: collaborative analysis of prospective studies. *AIDS* 2007;21:1185-1197.

[6] The Strategies for Management of Antiretroviral Therapy (SMART) Study Group. CD4+ count - guided interruption of antiretroviral treatment. *N Engl J Med* 2006; 355:2283-2296.

[7] Silverberg, MJ, Neuhaus J, Bower M, et al. Risk of cancers during interrupted antiretroviral therapy in the SMART Study. *AIDS* 2007;21:1-7.

[8] The Strategies for Management of Antiretroviral Therapy (SMART) Study Group. Clinical outcomes in the SMART study after recommendation to re-initiate antiretroviral therapy for participants randomized to the CD4+ guided interruption strategy. *Ann Intern Med* 2008; 149:289-299.

[9] Emery S, Neuhaus J, Phillips A, et al. Clinical outcomes in antiretroviral naïve patients and those not taking antiretroviral therapy at entry to SMART *J Infect Dis* 2008;197:1133-1144.

[10] Mocroft A, Soriano V, Rockstroh J, et al. Is there evidence for an increase in the death rate from liver-related disease in patients with HIV? *AIDS* 2005; 19:2117-2125.

[11] The D:A:D Study Group. Liver-related deaths in persons infected with the human immunodeficiency virus. *Arch Intern Med* 2006;166:1632-1641.

[12] Qurishi N, Kreuzberg C, Lüchters G, et al. Effect of antiretroviral therapy on liver-related mortality in patients with HIV and hepatitis C virus infection. *Lancet* 2003; 362:1708-1713.

[13] Phillips AN, Neaton J, Lundgren J. The role of HIV in serious diseases other than AIDS. *AIDS* 2008;22:2409-2418. .

[14] Kitahata MM, Gange SJ, Moore RD. Initiating rather than deferring HAART at a CD4+ count between 351-500 cells/mm3 is associated with improved survival. 48th International Conference on Antimicrobial Agents and Chemotherapy (ICAAC 2008). Washington, DC. October 25-28, 2008. Abstract H-896b.

[15] The D:A:D Study Group. Combination antiretroviral therapy and the risk of myocardial infarction. *N Engl J Med* 2003;349:1993-2003.

[16] The D:A:D Study Group. Class of antiretroviral drugs and risk of myocardial infarction. *N Engl J Med* 2007;356:1723-1735.

[17] D:A:D Study Group. Use of nucleoside reverse transcriptase inhibitors and risk of myocardial infarction in HIV-infected patients enrolled in the D:A:D study: a multi-cohort collaboration. *Lancet* 2008;371:1417-1426..

[18] The SMART/INSIGHT and D:A:D Study Groups. Use of nucleoside reverse transcriptase inhibitors and risk of myocardial infarction in HIV-infected patients. *AIDS* 2008;22:F17-F24.

[19] The UK Collaborative HIV Cohort (CHIC) Study Steering Committee. HIV diagnosis at CD4 count above 500/mm^3 and progression to below 350/mm^3 without antiretroviral therapy. *J Acquir Immune Defic Syndr* 2007;46:275-278.

[20] Mellors JW, Muñoz A, Giorgi JV, et al. Plasma viral load and CD4+ lymphocytes as prognostic markers of HIV-1 infection. *Ann Intern Med* 1997; 126:946-954.

[21] Rodŕiguez B, Sethi AK, Cheruvu VK, et al. Predictive value of plasma HIV RNA level on rate of CD4 T-cell decline in untreated HIV infection. *JAMA* 2006; 296:1498-1506.

[22] Ware J, Jr., Kosinski M, Keller SD. A 12-Item Short-Form Health Survey: construction of scales and preliminary tests of reliability and validity. *Med Care.* Mar 1996;34:220-233.

[23] Wu AW, Huang IC, Gifford AL, et al. Creating a crosswalk to estimate AIDS Clinical Trials Group quality of life scores in a nationally representative sample of persons in care for HIV in the United States. *HIV Clin Trials.* 2005;6:147-157.

[24] Burman WJ, Grund B, Roediger MP et al. The impact of episodic CD4+ cell count-guided antiretroviral therapy on quality of life. *J Acquir Immune Defic Syndr* 2008;47:185-93.

[25] Quinn TC, Wawer MH, Sewankambo N, et al. Viral load and heterosexual transmission of human immunodeficiency virus type 1. Rakai Project Study Group. *N Engl J Med* 2000;342;921-929.

[26] Fideli US, Allen SA, Musonda R, et al. Virologic and immunologic dterminants of heterosexual transmission of human immunodeficiency virus type 1 in Africa. *AIDS Res Hum Retroviruses* 2001;17:901-910.

[27] Johnson VA, Brun-Vézinet F, Clotet B, et al. Update of the drug resistance mutations in HIV-1 : 2007. *Topics in HIV Medicine* 2007;15:119-124.

[28] Phillips AN, Gazzard B, Gilson R, et al. Rate of AIDS diseases or death in HIV infected antiretroviral therapy-naïve individuals with high CD4 count. *AIDS* 2007;21:1717-1721.

[29] Neaton JD, Grund B. Earlier initiation of antiretroviral therapy in treatment naive patients: implications of results of treatment interruption trials. *Current Opinion in HIV and AIDS* 2008;3:112-117.

[30] National Kidney Foundation: K/DOQI clinical practice guidelines for chronic kidney disease: evaluation, classification, and stratification. *Am J Kidney Dis* 2002;39: S1-S266.

[31] Hirsch MS, Brun-Vezinet F, D'Aquila R, et al. Antiretroviral drug resistance testing in adult HIV-1 infection. Recommendations of International AIDS Society-USA Panel. *JAMA* 2000;283:2417-2426.

[32] Vandamme AM, Sönnerborg A, Ait-Khaled M, et al. Updated European recommendations for the clinical use of HIV drug resistance testing. *Antivir Ther* 2004;9:829-848.

[33] Dybul M, Nis-Kraske E, Daucher M, et al. Long-cycle structured intermittent vesus continuous highly active antiretroviral therapy for the treatment of chronic infection with human immunodeficiency virus: effects on drug toxicity and on immunologic and virologic parameters. *J Infect Dis* 2003;188: 388-396.

[34] Fox Z, Phillips A, Cohen C, et al. Viral re-suppression and detection of drug resistance following interruption of a suppressive non-nucleoside reverse transcriptase inhibitor-based regimen. *AIDS* 2008;22:2279-2289.

[35] World Health Organization. Antiretroviral drugs for treatment of pregnant women and preventing HIV infection in infants: Towards Universal Access. Recommendations for a public health approach. August 7, 2006. (http://www.who.int/hiv/pub/guidelines/en/).

[36] Public Health Service. Recommendations for use of antiretroviral drugs in pregnant HIV-1 infected women for maternal health and interventions to reduce perinatal HIV-1 transmission in the United States. October 12, 2006. (http://AIDSinfo.nih.gov).

[37] Australian Antiretroviral Guidelines Panel. Australian commentary to the USA Department of Health and Human Services Antiretroviral Guidelines. October 10, 2006. (http://www.ashm.org.au/aust-guidelines/).

[38] Kalbfleisch JD, Prentice RL. The statistical analysis of failure time data. 2nd ed., New York, NY, John Wiley, 2002.

[39] O'Brien PC, Fleming TR. A multiple testing procedure for clinical trials. *Biometrics* 1979;35:549-56.

[40] Lan KKG, DeMets DL. Discrete sequential boundaries for clinical trials. *Biometrika* 1983;70:659-63.

[41] The Coronary Drug Project Research Group. 1981. Practical aspects of decision making in clinical trials: the Coronary Drug Project as a case study. *Cont Clin Trials* 1:363-376.

[42] Dean M, Carrington M, Winkler C, et al. Genetic restriction of HIV-1 infection and progression to AIDS by a deletion allele of the CKR5 structural gene. Hemophilia Growth and Development Study, Multicenter AIDS Cohort Study, Multicenter Hemophilia Cohort Study, San Francisco City Cohort, ALIVE Study. *Science* 1996; 273(5283):1856-1862.

[43] Liu R, Paxton WA, Choe S, et al. Homozygous defect in HIV-1 coreceptor accounts for resistance of some multiply-exposed individuals to HIV-1 infection. *Cell* 1996; 86(3):367-377.

[44] Samson M, Libert F, Doranz BJ, et al. Resistance to HIV-1 infection in caucasian individuals bearing mutant alleles of the CCR-5 chemokine receptor gene. *Nature* 1996; 382(6593):722-725.

[45] Mallal S, Nolan D, Witt C, et al. Association between presence of HLA-B*5701, HLA-DR7, and HLA-DQ3 and hypersensitivity to HIV-1 reverse-transcriptase inhibitor abacavir. *Lancet* 2002;359:727-732.

[46] Brew BJ. HIV Neurology. First ed. New York: Oxford University Press; 2001.

[47] Carne CA, Tedder RS, Smith A, et al. Acute encephalopathy coincident with seroconversion for anti-HTLV-III. *Lancet* 1985;2(8466):1206-8.

[48] Ho DD, Sarngadharan MG, Resnick L, et al. Primary human T-lymphotropic virus type III infection. *Ann Intern Med* 1985;103:880-883.

[49] Tambussi G, Gori A, Capiluppi B, et al. Neurological symptoms during primary human immunodeficiency virus (HIV) infection correlate with high levels of HIV RNA in cerebrospinal fluid. *CID* 2000;30(6):962-5.

[50] Abdulle S, Mellgren A, Brew BJ, et al. CSF neurofilament protein (NFL) - a marker of active HIV-related neurodegeneration. *J Neurol* 2007;254(8):1026-32.

[51] Martin A, Heyes MP, Salazar AM, et al. Progressive slowing of reaction time and increasing cerebrospinal fluid concentrations of quinolinic acid in HIV-infected individuals. *J Neuropsychiatry Clin Neurosci* 1992;4(3):270-9.

[52] Chiodi F, Keys B, Albert J, et al. Human immunodeficiency virus type 1 is present in the cerebrospinal fluid of a majority of infected individuals. *J Clin Microbiol* 1992;30(7):1768-71.

[53] Garcia F, Niebla G, Romeu J, et al. Cerebrospinal fluid HIV-1 RNA levels in asymptomatic patients with early stage chronic HIV-1 infection: support for the hypothesis of local virus replication. *AIDS* 1999;13(12):1491-6.

[54] Spudich SS, Nilsson AC, Lollo ND, et al. Cerebrospinal fluid HIV infection and pleocytosis: relation to systemic infection and antiretroviral treatment. *BMC Infect Dis* 2005;5:98.

[55] Staprans S, Marlowe N, Glidden D, et al. Time course of cerebrospinal fluid responses to antiretroviral therapy: evidence for variable compartmentalization of infection. *AIDS* 1999;13(9):1051-61.

[56] Cepok S, von Geldern G, Nolting T, et al. Viral load determines the B-cell response in the cerebrospinal fluid during human immunodeficiency virus infection. *Ann Neurol* 2007.

[57] An SF, Groves M, Gray F, Scaravilli F. Early entry and widespread cellular involvement of HIV-1 DNA in brains of HIV-1 positive asymptomatic individuals. *J Neuropathol Exp Neurol* 1999;58(11):1156-62.

[58] Gray F, Scaravilli F, Everall I, et al. Neuropathology of early HIV-1 infection. *Brain Pathol* 1996;6(1):1-15.

[59] Sinclair E, Gray F, Ciardi A, Scaravilli F. Immunohistochemical changes and PCR detection of HIV provirus DNA in brains of asymptomatic HIV-positive patients. *J Neuropathol Exp Neurol* 1994;53(1):43-50.

[60] McCrossan M, Marsden M, Carnie FW, et al. An immune control model for viral replication in the CNS during presymptomatic HIV infection. *Brain* 2006;129(Pt 2):503-16.

[61] Sacktor N, Tarwater PM, Skolasky RL, et al. CSF antiretroviral drug penetrance and the treatment of HIV-associated psychomotor slowing. *Neurology* 2001;57(3):542-4.

[62] Robertson KR, Robertson WT, Ford S, et al. Highly active antiretroviral therapy improves neurocognitive functioning. *J Acquir Immune Defic Syndr* 2004;36(1):562-6.

[63] Cohen RA, Boland R, Paul R, et al. Neurocognitive performance enhanced by highly active antiretroviral therapy in HIV-infected women. *AIDS* 2001;15(3):341-5.

[64] Price RW, Yiannoutsos CT, Clifford DB, et al. Neurological outcomes in late HIV infection: adverse impact of neurological impairment on survival and protective effect of antiviral therapy. AIDS Clinical Trial Group and Neurological AIDS Research Consortium study team. *AIDS* 1999;13(13):1677-85.

[65] Tozzi V, Balestra P, Galgani S, et al. Positive and sustained effects of highly active antiretroviral therapy on HIV-1-associated neurocognitive impairment. *AIDS* 1999;13(14):1889-97.

[66] Ferrando S, van Gorp W, McElhiney M, et al. Highly active antiretroviral treatment in HIV infection: benefits for neuropsychological function. *AIDS* 1998;12(8):F65-70.

[67] Marcotte TD, Deutsch R, McCutchan JA, et al. Prediction of incident neurocognitive impairment by plasma HIV RNA and CD4 levels early after HIV seroconversion. *Arch Neurol* 2003;60(10):1406-12.

[68] White DA, Heaton RK, Monsch AU. Neuropsychological studies of asymptomatic human immunodeficiency virus-type-1 infected individuals. The HNRC Group. HIV Neurobehavioral Research Center. *J Int Neuropsychol Soc* 1995;1(3):304-15.

[69] Heaton RK, Grant I, Butters N, et al. The HNRC 500--neuropsychology of HIV infection at different disease stages. HIV Neurobehavioral Research Center. J *Int Neuropsychol* Soc 1995;1(3):231-51.

[70] Reger M, Welsh R, Razani J, Martin DJ, Boone KB. A meta-analysis of the neuropsychological sequelae of HIV infection. *J Int Neuropsychol Soc* 2002;8(3):410-24.

[71] Heaton RK, Velin RA, McCutchan JA, et al. Neuropsychological impairment in human immunodeficiency virus-infection: implications for employment. HNRC Group. HIV Neurobehavioral Research Center. *Psychosom Med* 1994;56(1):8-17.

[72] Instruction/Owner's Manual. Grooved Pegboard. In: Lafayette Instruments 1989.

[73] D'Elia LF, Satz P, Uchiyama CL, et al. Color Trails Test. Odessa, FL. In: Psychological Assessment Resources, Inc; 1996.

[74] The Psychological Corporation. Wechsler Adult Intelligence Scale-Revised. San Antonio, TX: The Psychological Corporation; 1981.

[75] Benedict RH, Schretlen D, Groninger L, Brandt J. Hopkins Verbal Learning Test-Revised: Normative data and analysis of inter-from and test-retest reliability. *The Clinical Neuropsychologist* 1998;12(1):43-55.

[76] Reitan RM. Manual for administration of neuropsychological test batteries for adults and children. Bloomington. In: Indiana University Press; 1969.

[77] Spreen O, Strauss E. A compendium of neuropsychological tests :administration, norms, and commentary. 2nd ed., New York, Oxford University Press: 1998

[78] Radloff LS. The CES-D scale: A self-report depression scale for research in the general population. *Applied Psychological Measurement* 1977;1:385-401.

[79] Letendre S, Marquie-Beck J, Capparelli E, et al for the CHARTER Group. Validation of the CNS penetration-effectiveness rank for quantifying antiretroviral penetration into the central nervous system. *Arch Neurol* 2008;65(1):65-70.

[80] O'Brien PC. Procedures for comparing samples with multiple endpoints. *Biometrics* 1984; 40:1079-1087.

[81] Faden R, Beauchamp TA. A History and Theory of Informed Consent. Oxford University Press; 1986.

[82] Emanual EJ, Wendler D, Grady C. What makes clinical research ethical? *JAMA* 2000; 283(20):2701-11.

[83] Bergler JH, Pennington AC, Metcalfe M, Freis ED. Informed consent: how much does the patient understand? *Clin Pharmacol Ther* 1980;27(4):435-39.

[84] Howard JM, DeMets D, BHAT Research Group. How informed is informed consent? The BHAT experience. *Control Clin Trials* 1981;2:287-303.

[85] Harrison K, Vlahov D, Jones K, Charon K, Clements ML. Medical eligibility, comprehension of the consent process, and retention of ijection drug users recruited for an HIV vaccine trial. *J Acquir Immune Defic Syndr* 1995;10:386-90.

[86] van Stuijvenberg M, Suur MH, de Vos S, et al. Informed consent, parental awareness, and reasons for participating in a randomised controlled study. *Arch Dis Child* 1998;79(2):120-25.

[87] Pace C, Emanuel EJ, Chuenyam T, et al. The quality of informed consent in a clinical research study in Thailand. *IRB* 2005;27:9-17.

[88] Pace C, Talisuna A, Wendler D, et al. Quality of parental consent in a Ugandan malaria study. *Am J Public Health* 2005;95:1184-89.

[89] Moodley K, Pather M, Myer L. Informed consent and participant perceptions of influenza vaccine trials in South Africa. *J Med Ethics* 2005;3:727-32.

[90] Karim QA, Karim SSA, Coovadia HM, Susser M. Informed consent for HIV testing in a South African hospital: Is it truly informed and truly voluntary? *Am J Pub Health* 1998;488:637-40.

[91] Leach A, Hilton S, Greenwood BM, et al. An evaluation of the informed consent procedure used during a trial of a *Haemophilus influenzae* type B conjugate vaccine undertaken in The Gambia, West Africa. *Soc Sci Med* 1999;48:139-48.

[92] Lynoe N, Hyder Z, Chowdhury M, Ekstrom L. Obtaining informed consent in Bangladesh. *N Engl J Med* 2001;344:460-61.

[93] Joubert G, Steinberg H, van der Ryst E, Chikobvu P. Consent for participation in the Bloemfontein vitamin A trial: How informed and voluntary? *Am J Public Health* 2003;93:582-84.

[94] Marshall PA, Adebamowo CA, Adeyemo AA, et al. Voluntary participation and informed consent to international genetic research. *Am J Public Health* 2006;96:1989-95.

[95] Taub HA, Baker MT, Sturr JF. Informed consent for research. Effects of readability, patient age, and education. *J Am Geriatr Soc* 1986;34(8):601-6.

[96] Davis TC, Holcombe RF, Berkel HJ, Pramanik S, Divers SG. Informed consent for clinical trials: A comparative study of standard versus simplified forms. *J Natl Cancer Inst* 1998;90(9):668-74.

[97] Coyne CA, Xu R, Raich P, et al. Randomized, controlled trial of an easy-to-read informed consent statement for clinical trial participation: A study of the Eastern Cooperative Oncology Group. *J Clin Oncol* 2003;21(5):836-42.

[98] Dresden GM, Levitt MA. Modifying a standard industry clinical trial consent form improves patient information retention as part of the informed consent process. *Acad Emerg Med* 2001;8(3):246-52.

[99] Young DR, Hooker DT, Freeberg FE. Informed consent documents: Increasing comprehension by reducing reading level. *IRB* 1990;12(3):1-5.

[100] NCI Template Consent Form, available at http://www.cancer.gov/clinicaltrials/understanding/simplification-of-informed-consent-docs/page3

[101] Hayes RJ, Bennett S. Simple sample size calculation for cluster-randomized trials. *Int J Epid* 1999;28:319-326.

[102] Olsen CH, Gatell J, Ledergerber B, et al. Risk of AIDS and death at given HIV-RNA and CD4 cell counts, in relation to specific antiretroviral drugs in the regimen. *AIDS* 2005; 19 (3): 319-330.

[103] Podlekareva D, Mocroft A, Dragsted UB, et al. Factors associated with the development of opportunistic infections in HIV-1-infected adults with high CD4(+) cell counts: A EuroSIDA study. *J Infect Dis* 2006; 194 (5): 633-641.

[104] Bogaards JA, Weverling GJ, Geskus RB, et al. Low versus high CD4 cell count as starting point for introduction of antiretroviral treatment in resource-poor settings: a scenario-based analysis. *Antiviral Therapy* 2003; 8 (1): 43-50.

[105] Weber R, Friis-Moller N, Sabin C et al. HIV and non-HIV related deaths and their relationship to immunodeficiency: The D:A:D Study. Abstract 106LB, 12th Conference on Retroviruses and Opportunistic Infections. Boston, USA, 2005.

[106] Hennessey KA, Giorgi JV, Kaplan AH, et al. AIDS onset at high CD4+ cell levels is associated with high viral load. *AIDS Research and Human Retroviruses* 2000; 16:103-107.

[107] The Strategies for Management of Antiretroviral Therapy (SMART) Study Group. Inferior clinical outcome of the CD4+ count - guided antiretroviral treatment interruption

strategy in SMART: role of CD4+ counts and HIV-RNA levels during follow-up. *J Infect Dis* 2008;197:1145-1155.

[108] Lempicki RA, Kovacs JA, Baseler MW, et al. Impact of HIV-1 infection and highly active antiretroviral therapy on the kinetics of CD4+ and CD8+ T cell turnover in HIV-infected patients. *Proc Natal Acad Sci* 2000;97:13778-13783.

[109] Brenchley JM, Price DA, Schacker TW, et al. Microbial translocation is a cause of systemic immune activation in chronic HIV infection. *Nature Med* 2006;12:1365-1371.

[110] Lange CG, Valdez H, Medvik K, et al. CD4+ T-lymphocyte nadir and the effect of highly active antiretroviral therapy on phenotypic and functional immune restoration in HIV-1 infection. *Clin Immunol* 2002;102:154-161.

[111] Day CL, Kaufmann DE, Kiepiela P, et al. PD-1 expression on HIV-specfiic T cells is associated with T-cell exhaustion and disease progression. *Nature* 2006;443:350-354.

[112] Sackoff JE, Hanna DB, Pfeiffer MR and Torian LV. Causes of death among persons with AIDS in the era of highly active antiretroviral therapy: New York City. *Ann Intern Med* 2006;145:397-406.

[113] Crum NF, Riffenburgh RH, Wegner S, et al. Comparisons of causes of death and mortality rates among HIV-infected persons: analysis of the pre-, early, and late HAART (highly active antiretroviral therapy) eras. *J Acquir Immune Defic Syndr* 2006;41:194-200.

[114] Reisler RB, Han C, Burman WJ, Tedaldi EM, Neaton JD. Grade 4 events are as important as AIDS events in the era of HAART. *J Acquir Immune Defic Syndr* 2003;34:379-386.

[115] Mocroft A, Brettle R, Kirk O, et al. Changes in the cause of death among HIV positive subjects across Europe: results from the EuroSIDA study. *AIDS* 2002;16:1663-71.

[116] Smit C, Geskus R, Walker S, et al. Effective therapy has altered the spectrum of cause-specific mortality following HIV seroconversion. AIDS 2006;20:741-9.

[117] Palella FJ, Jr., Baker RK, Moorman AC, et al. Mortality in the highly active antiretroviral therapy era: changing causes of death and disease in the HIV outpatient study. *J Acquir Immune Defic Syndr* 2006;43:27-34.

[118] Lau B, Gange S, Moore RD. Risk of non-AIDS related mortality may exceed risk of AIDS-related mortality among individuals enrolling into care with CD4+ counts greater than 200 cells/mm^3. *J Acquir Immune Defic Syndr* 2006 (epublication).

[119] Mocroft A, Ledergerber B, Katlama C, et al. Decline in the AIDS and death rates in the EuroSIDA study : an observational study. *Lancet* 2003;362:22-29.

[120] The Data Collection on Adverse Events of Anti-HIV Drugs (D:A:D) Study Group. HIV-induced immunodeficiency and mortality from AIDS-defining and non-AIDS-defining malignancies. *AIDS* 2008;22:2143-2153.

[121] Baker JV, Peng G, Rapkin J, et al. CD4+ cell count and risk of non-AIDS diseases following initial treatment for HIV infection. *AIDS* 2008;22:841-848.

[122] Vajdic CM, McDonald SP, McCredie MRE, et al. Cancer incidence before and after kidney transplantation. *JAMA* 2006; 296:2823-2831.

[123] Grulich AE, van Leeuwen MT, Falster MO, Vajdic C. Incidence of cancers in people with HIV/AIDS compared with immunosuppressed transplant recipients: a meta-analysis. *Lancet* 2007;370:59-67.

[124] Patel P, Hanson DL, Sullivan PS, et al. Incidence of types of cancer among HIV-infected persons compared with the general population in the United States, 1992-2003. *Ann Intern Med* 2008;148:728-736.

[125] Clifford GM, Polesel J, Rickenbach M, et al. Cancer Risk in the Swiss HIV Cohort Study: Associations with immunodeficiency, smoking, and highly active antiretroviral therapy. *J Natl Cancer Inst* 2005;97:425 – 432.

[126] Engels EA, Brock MV, Chen J, et al. Elevated incidence of lung cancer among HIV-infected individuals. *J Clinical Oncology* 2006;24(9):1383-1388.

[127] Kirk GD, Merlo C, O'Driscoll P, et al. HIV infection is associated with an increased risk of lung cancer, independent of smoking. *CID* 2007;45:103-110.

[128] Chaturvedi A, Pfeiffer RM, Chang L, et al. Elevated risk of lung cancer among people with HIV. *AIDS* 2007; 21:207-213.

[129] McGinnis KA, Fultz SL, Skanderson M, et al. Hepatocellular carcinoma and non-Hodgkin's lymphoma: the roles of HIV, hepatitis C infection, and alcohol abuse. *J Clin Oncol* 2006;24:5005-5009.

[130] Nunez MJ, Martin-Carbonero L, Moreno V, et al. Impact of antiretroviral treatment-related toxicities on hospital admissions in HIV-infected patients. *AIDS Research and Human Retroviruses* 2006; 22: 825-829.

[131] van Leth F, Phanuphak P, Ruxrungtham K, et al. Comparison of first-line antiretroviral therapy with regimens including nevirapine, efavirenz, or both drugs, plus stavudine and lamivudine: a randomised open-label trial, the 2NN Study. *Lancet* 2004; 363:1253-1263.

[132] Tedaldi E, Peters L, Neuhaus J, et al. Opportunistic disease and mortality in patients co-infected with hepatitis B or C virus in the Strategic Management of Antiretroviral Therapy(SMART) study. *CID* 2008;47:1468-1475.

[133] Holmberg SD, Moorman AC, Williamson JM, et al. Protease inhibitors and cardiovascular outcomes in patients with HIV-1. *Lancet* 2002; 360:1747-1748.

[134] Iloeje UH, L'Italien G, Mauskoph J, et al. Protease inhibitor exposure and increased risk of cardiovascular disease in HIV-infected patients. *HIV Medicine* 2005; 6:37-4.

[135] Mary-Krause M, Cotte L, Simon A, Partisani M, Costagliola D. Increased risk of myocardial infarction with duration of protease inhibitor therapy in HIV-infected men. *AIDS* 2003; 17:2479-2486.

[136] Triant VS, Lee H, Hadigan C, Grinspoon SK. Increased acute myocardial infarction rates and cardiovascular risk factors among patients with HIV disease. *J Clin Endocrin Metab* 2007 (epublication).

[137] SMART CVD Risk Writing Group. Interruption of antiretroviral therapy and risk of cardiovascular disease in persons with HIV-1 infection: exploratory analyses from the SMART trial *Antivir Ther* 2008;13:177-187.

[138] Riddler SA, Smit E, Cole SR, et al. Impact of HIV infection and HAART on serum lipids in men. *JAMA* 2003;289:2978-2982.

[139] Rose H, Woolley I, Hoy J, et al. HIV infection and high-density lipoprotein: the effect of the disease versus the effect of treatment. *Metabolism Clinical and Experimental* 2006;55:90-95.

[140] Mujawar Z, Rose H, Morrow MP, et al. Human immunodeficiency virus impairs reverse cholesterol transport from macrophages. *PLoS Biology* 2006;4:1970-1983.

[141] Navab M, Anantharamich GM, Reddy, et al. Mechanism of disease: proatherogenic HDL – an evolving field. *Nature Clinical Practice* 2006;2:504-511.

[142] Chait A, Han CY, Oram JF, Heinecke JW. Lipoprotein-associated inflammatory proteins: markers or mediators of cardiovascular disease. *J Lipid Research* 2005;46:389-403.

[143] Cines DB, Pollak ES, Buck CA, et al. Endothelial cells in physiology and in the pathophysiology of vascular disorders. *Blood* 1998;91:3527-3561.

[144] Kuller LH, Tracy R, Belloso W, et al. Inflammatory and coagulation biomarkers and mortality in patients with HIV infection. *PLoS Medicine* 2008 5(10):e203.

[145] De Larrangas GF, Petroni A, Deluchi G, Alonso BS, Benetucci JA. Viral load and disease progression as responsible for endothelial activation and/or injury in human immunodeficiency virus-1-infected patients. *Blood Caogul Fibrinolysis* 2003;14:15-18.

[146] Wolf K, Tsakiris DA, Weber R, et al. Antiretroviral therapy reduces markers of endothelial and coagulation activation in patients with human immunodeficiency virus type 1. *JID* 2002;185:456-462.

[147] Fisher SA, Miller TL, Lipshultz SE. Impact of HIV and highly active antiretroviral therapy on leukocyte adhesion molecules, arterial inflammation, dyslipidemia, and atherosclerosis. *Atherosclerosis* 2006;185:1-11.

[148] Gupta, SK, Eustace JA, Winston JA, et al. Guidelines for the management of chronic kidney disease in HIV-infected patients: recommendations of the HIV Medicine Association of the Infectious Diseases Society of America. *Clin Infect Dis* 2005; 40:1559-1585.

[149] Kimmel PL, Barisoni L, Koop JB. Pathogenesis and treatment of HIV-associated renal disease: lesson from clinical and animal studies, molecular pathologic correlations, and genetic investigations. *Annal Intern Med* 2003;139:214-226.

[150] Szczech LA, Gange S, van der Horst C, et al. Predictors of proteinuria and renal failure among women with HIV infection. *Kidney International* 2002;61:195-202.

[151] Han TM, Naicker S, Ramdial PK, Assounga AG. A cross-sectional study of HIV-seropositive patients with varying degrees of proteinuria in South Africa. *Kidney Int'l* 2006;69:2243-2250.

[152] Cantor ES, Kimmel PL, Bosch JP. Effect of race on expression of acquired immunodeficiency syndrome-associated nephropathy. *Arch Intern Med* 1991;151:125-128.

[153] Lucas GM, Eustace JA, Sozio S, et al. Highly active antiretroviral therapy and the incidence of HIV-1-associated nephropathy: a 12-year cohort study. *AIDS* 2004; 18:541-546.

[154] Atta MG, Gallant JE, Rahman MH, et al. Antiretroviral therapy in the treatment of HIV-associated nephropathy. *Nephrol Dial Transplant* 2006;21:2809-2813.

[155] Szczech LA, Gupta SK, Habash R, et al. The clinical epidemilogy and course of the spectrum of renal diseases associated with HIV infection. *Kidney Int'l* 2004; 66:1145-1152.

[156] Moore RD, Keruly JC, Gebo KA, et al. An improvement in virologic response to highly active antiretroviral therapy in clinical practice from 1996 to 2002. *J Acquir Immune Defic Syndr* 2005; 39:195-198.

[157] Lampe FC, Smith CJ, Madge S, et al. Success of clinical care for human immunodeficiency virus infection according to demographic group among sexually infected patients in a routine clinic population, 1999 to 2004. *Arch Intern Med* 2007;167: 692-700.

[158] The Antiretroviral (ART) Collaboration. HIV treatment response and prognosis in Europe and North America in the first decade of highly active antiretroviral therapy: a collaborative analysis. *Lancet* 2006; 368:451-458.

[159] Smith CJ, Sabin CA, Lampe FC, et al. The potential for CD4 cell increases in HIV-positive individuals who control viraemia with highly active antiretroviral therapy. *AIDS* 2003; 17:963-969.

[160] Moore RD, Keruly JC. CD4+ cell count 6 years after commencement of highly active antiretroviral therapy in persons with sustained virologic suppression. *CID* 2007; 44:441-446.

[161] Mocroft A, Phillips A, Gatell J, et al. Normalization of CD4 counts in patients with HIV-1 infection and maximum virological suppression who are taking combination antiretroviral therapy: an observational cohort study. *Lancet* 2007; 370:407-413.

[162] Gras L, Kesselring AM, Griffin JT, et al. CD4 cell counts of 800 cells/mm^3 or greater after 7 years of highly active antiretroviral therapy are feasible in most patients starting with 350 cells/mm^3 or greater. *J Acquir Immune Defic Syndr* 2007;45:183-192.

[163] Pakker NG, Kroon ED, Roos MT, et al. Immune restoration does not invariably occur following long-term HIV-1 suppression during antiretroviral therapy. *AIDS* 1999;13(2):203-212.

[164] Moore DM, Hogg RS, Chan K, et al. Disease progression in patients with virological suppression in response to HAART is associated with the degree of immunological response. *AIDS* 2006;20:371-377.

[165] Moore DM. Regimen dependent variations in immunologic responses to HAART in the presence of virologic suppression. 11th International Workshop on HIV Observational Databases, Monte Carlo, 2007 (Abstract 23).

[166] The Antiretroviral Therapy (ART) Cohort Collaboration. Prognostic importance of initial response in HIV-1 infected patients starting potent antiretroviral therapy: analysis of prospective studies. *Lancet* 2003;362:679-686.

[167] Lange CG, Lederman MM, Medvik K, et al. Nadir Cd4+ T-cell count and numbers of CD28+ CD4+ T-cells predict functional responses to immunizations in chronic HIV-1 infection. *AIDS* 2003;17:2015-2023.

[168] Robbins GK, DeGrutolla V Schafer RW, et al. Comparison of sequential three-drug regimens as initial therapy for HIV-1 infections. *N Engl J Med* 2003; 249:2293-2303.

[169] INITIO Trial International Coordinating Committee. Virological and immunological outcomes at 3 years after starting antiretroviral therapy with regimens containing non-nucleoside reverse transcriptse inhibitor, protease inhibitor, or both in INITIO: open-label randomized trial. *Lancet* 2006;368:287-299.

[170] MacArthur RD, Novak RM, Peng G, et al. A comparison of three highly active antiretroviral treatment strategies consisting of non-nucleoside reverse transcriptase inhibitors, protease inhibitors, or both in the presence of nucleoside reverse transcriptase inhibitors as initial therapy (CPCRA 058 FIRST Study): a long-term randomized trial. *Lancet* 2006;368:2125-2135.

[171] Rajesh G, Spritzler J, Chan E, et al. Effect of baseline- and treatment-related factors on immunologic recovery after initiation of antiretroviral therapy in HIV-1 positive subjects: results from ACTG 384. *JAIDS* 2006;42:426-434.

[172] Walmsley S, Bernstein B, King M, et al. Lopinavir-ritonavir versus nelfinavir for the initial treatment of HIV infection. *N Engl J Med* 2002;346:2039-2046.

[173] Battegay M, Nüesch R, Hirschel B, Kaufmann GR. Immunological recovery and antiretroviral therapy in HIV-1 infection. *Lancet Infect Dis* 2006;6:280-287.

[174] Lang W, Perkins H, Anderson RE, et al. Patterns of T lymphocye changes with human immunodeficiency virus infection: from seroconversion to the development of AIDS. *J Acquir Immune Defic Syndr* 1989;2:63-69.

[175] CASCADE Collaboration. Differences in CD4 cell counts at seroconversion and decline among 5739 HIV-1 infected individuals with well-estimated dates of seroconversion. *J Acquir Immune Defic Syndr* 2003;34:76-83.

[176] Peto R, Lopez AD, Boreham J, Thun M, Heath C. Mortality from smoking in developed countries. indirect estimation from national vital statistics. *Lancet* 1992;339:1268-1278.

[177] Stamler J. Established major coronary risk factors: historical overview. In: Marmot MG, Elliott P (eds) Coronary Heart Disease Epidemiology: From Aetiology to Public Health, Oxford University Press, June 2005.

[178] Stamler J, Neaton J, Garside DB, Davigulus ML. The major adult cardiovascular diseases: a global historical perspective. In: Lauer R, Burns TL, Daniels S (eds.). Pediatric Prevention of Atherosclerotic Cardiovascular Disease. Oxford University Press, 2006.

[179] Anderson, K.M., Odell PM, Wilson PW, Kannel WB. Cardiovascular disease risk profiles. *Am Heart J* 1991;121:293-298.

[180] Klag MJ, Whelton PK, Randall BL, et al. Blood pressure and end-stage renal disease. *N Engl J Med* 1996;334:13-18.

[181] Klag MJ, Whelton PW, Randall BL, et al. End-stage renal disease in African-American and White men. 16-year MRFIT findings. *JAMA* 1997;277:1293-1298.

[182] Ishani A, Grandits GA, Grimm RH, et al. Association of single measurements of dipstick proteinuria, estimated glomerular filtration rate, and hematocrit with 25-year incidence of end-stage renal disease in the Multiple Risk Factor Intervention Trial. *J Am Soc Nephrol* 2006;17:1444-1452.

[183] National Kidney Foundation: K/DOQI clinical practice guidelines for chronic kidney disease: evaluation, classification, and stratification. *Am J Kidney Dis* 2002;39: S1-S266.

[184] Pawlotsky JM. Pathophysiology of hepatitis C virus infection and related liver disease. *TRENDS in Microbiology* 2004;12:96-102.

[185] Rockstroh JK, Mocroft A, Soriano V, et al. Influence of hepatitis C virus infection on HIV-1 disease progression and response to highly active antiretroviral therapy. *J Infect Dis* 2005;192: 992-1002.

[186] Greub G, Ledergerber B, Battegay M, et al. Clinical progression, survival and immune recovery during antiretroviral therapy in patients with HIV-1 and hepatitis C coinfection. *Lancet* 2000; 356:1800-1805.

[187] Sulkowski MS, Moore RD, Mehta SH, Chaisson RE, Thomas DL. Hepatitis C and progression of HIV disease. *JAMA* 2002; 288:199-206.

[188] Hirsch MS, Brun-Vézinet F, Clotel B, et al. Antiretroviral drug resistance testing in adults infected with human immunodeficiency virus type 1: 2003 recommendations of an International AIDS Society – USA Panel. *CID* 2003;37:113-128.

[189] D'Arminio MA, Cozzi Lepri A, Rezza G, et al. Insights into the reasons for discontinuation of the first highly active antiretroviral theapy (HAART) regimen in a cohort of antiretroviral naïve patients. *AIDS* 2000;14:499-507.

[190] Moore RD, Keruly JC, Gebo KA, Lucas GM. An improvement in virologic response to highly active antiretroviral therapy in clinical practice from 1996 through 2002. J *Acquir Immune Defic Syndr* 2005;39:195-198.

[191] Lampe FC, Gatell JM, Staszewski S, et al. Changes over time in risk of initial virological failure of combination antiretroviral therapy. *Arch Intern Med* 2006;166:521-528.

[192] UK HIV Drug Resistance Database. Long term probability of detection of HIV-1 drug resistance after starting antiretroviral therapy in routine clinical practice. *AIDS* 2005;19:487-494.

[193] Musicco M, Lazzarin A, Nicolosi A, et al. Antiretroviral treatment of men infected with human immunodeficiency virus type 1 reduces the incidence of heterosexual transmission. Italian Study Group on HIV Heterosexual Transmission. *Arch Intern Med* 1994;154:1971-1976.

[194] Law MG, Prestage G, Grulich A, Van de Ven P, Kippax S. Modeling the effect of combination antiretroviral treatments on HIV incidence. *AIDS* 2001;15:1287-1294.

[195] Montaner JSG, Hogg R, Wood E, et al. The case for expanding access to highly active antiretroviral therapy to curb the growth of the HIV epidemic. *Lancet* 2006;368:531-536.

[196] Burman W, Grund B, Neuhaus J, et al. Episodic antiretroviral therapy increases HIV transmission risk compared to continuous therapy: Results of a randomized controlled trial. *J Acquir Immun Defic Synd* 2008; 49:142-150.

[197] Hirsch MS, Brun-Vezinet F, D'Aquila R, et al. Antiretroviral drug resistance testing in adult HIV-1 infection. Recommendations of International AIDS Society-USA Panel. *JAMA* 2000;283:2417-2426.

[198] Vandamme AM, Sönnerborg A, Ait-Khaled M, et al. Updated European recommendations for the clinical use of HIV drug resistance testing. *Antivir Ther* 2004; 9:829-848.

[199] Sax PE, Islam R, Walensky RP, et al. Should resistance testing be performed for treatment-naïve HIV-infected patients? A cost-effectiveness analysis. *Clin Inf Dis* 2005; 41:1316-1323.

[200] Taylor JMG, Cumberland WG, Sy JP. A stochastic model for analysis of longitudinal AIDS data. *J Amer Stat Assoc* 1994; 89:727-736.

Strategic Timing of AntiRetroviral Treatment (START)

Sponsored by:
The University of Minnesota
Minneapolis, Minnesota, USA

In collaboration with four International Coordinating Centers (ICCs) of the INSIGHT Network:

Copenhagen HIV Programme (CHIP) -- Copenhagen, Denmark
Medical Research Council (MRC) Clinical Trials Unit -- London, United Kingdom
National Centre in HIV Epidemiology and Clinical Research (NCHECR), University of New South Wales -- Sydney, Australia
The Institute for Clinical Research at the Veterans Affairs Medical Center -- Washington, D.C., USA

Funded by:
Division of AIDS, The National Institute of Allergy and Infectious Diseases (NIAID), National Institutes of Health (NIH)
Agence Nationale de Recherches sur le SIDA et les Hépatites Virales (ANRS, France)
Bundesministerium für Bildung und Forschung (BMBF, Germany)
NEAT - European AIDS Treatment Network
Australian National Health and Medical Research Council (NHMRC)
Department of Bioethics, The Clinical Center, NIH
Division of Clinical Research, NIAID, NIH
National Cancer Institute (NCI), NIH
National Heart, Lung, and Blood Institute (NHLBI), NIH
National Institute of Mental Health (NIMH), NIH
National Institute of Neurological Disorders and Stroke (NINDS), NIH

Pharmaceutical support provided by:
Abbott Laboratories, Inc.
Bristol-Myers Squibb
Gilead Sciences, Inc.
GlaxoSmithKline, Inc.
Merck & Co., Inc.
Tibotec Pharmaceuticals, Ltd.

[illegible]
Oct 27, 2010

Protocol Co-Chairs: Abdel Babiker, Sean Emery, Fred Gordin, Jens Lundgren

The START protocol is being managed and conducted by the International Network for Strategic Initiatives in Global HIV Trials (INSIGHT) with primary support by the U.S. National Institutes of Health through grants from the Division of AIDS, NIAID, and other NIH institutes to the University of Minnesota.

The University of Minnesota will serve as the sponsor for the study and will subcontract with four ICCs that will be responsible for implementation of Good Clinical Practice (GCP) and for oversight of the conduct of the trial at clinical research sites. The University of Minnesota is a constitutional entity under the laws of the State of Minnesota and assumes liability only to the extent provided under the Minnesota Tort Claims Act, Minnesota Statutes, Section 3.736.

The legal representative for the START trial in Europe is the Copenhagen HIV Programme (CHIP).

TABLE OF CONTENTS

1 SYNOPSIS 1

2 BACKGROUND AND RATIONALE 7

2.1 Primary Study Hypothesis 7

2.2 Background on Which Study Hypothesis Was Generated 7
2.2.1 Current evidence and practice on the timing of ART initiation 7
2.2.2 Summary of Potential Risks and Benefits 9

2.3 Rationale for Selected Study Design 9
2.3.1 CD4+ Cell Count Entry Criteria and CD4+ Count Deferral Criteria for Control Group 9
2.3.2 Broad Entry Criteria 10
2.3.3 Composite Endpoint of AIDS* and Non-AIDS 10
2.3.4 Major Secondary Outcomes 11

2.4 Summary 12

3 METHODOLOGY 14

3.1 Study Design 14

3.2 Study Objectives 14
3.2.1 Primary Objective 14
3.2.2 Secondary Objectives 14
3.2.3 Other Objectives 16

3.3 Primary Study Endpoint 16

3.4 Randomization 17

3.5 Sample Size and Statistical Considerations 17

3.6 Participant Selection 19
3.6.1 Inclusion Criteria 19
3.6.2 Exclusion Criteria 19

3.7 Study Plan 20
3.7.1 Treatment Strategies 20
3.7.2 Concomitant Medications 21
3.7.3 Baseline Screening 21
3.7.4 Participant Follow-up 23
3.7.5 Participant Relocation to a New Site 24
3.7.6 Stored Samples and Future Research 25

3.8 Event Reporting ... 25
3.8.1 AIDS and Non-AIDS Events ... 25
3.8.2 Deaths ... 26
3.8.3 Pregnancy Outcomes ... 26
3.8.4 Reporting Serious Events ... 26
3.8.5 Serious Adverse Event Reporting Required by the European Union... 27

4 CLINICAL MANAGEMENT ISSUES ... 28

4.1 Management of ART ... 28
4.1.1 Use of Potent Combination ART ... 28
4.1.2 Factors Affecting Choice of an ART Regimen ... 28
4.1.3 Antiretroviral Drug Product Information ... 28
4.1.4 Approved Sources for Antiretroviral Drugs ... 28
4.1.5 Intolerance of an Antiretroviral Drug ... 29
4.1.6 Drug Toxicity and Grading ... 29
4.1.7 Use of Antiretroviral Drugs in Women of Child-bearing Potential ... 29
4.1.8 Abacavir Hypersensitivity Reaction ... 29
4.1.9 Resistance Testing ... 30
4.1.10 Management of Virological Failure ... 30
4.1.11 Discontinuation of ART ... 30
4.1.12 Documentation of Reasons for Initiation or Change in ART ... 31
4.1.13 Use of Drugs for Prophylaxis for Opportunistic Diseases ... 31
4.1.14 Provision of Antiretroviral Drugs at Study Completion ... 31

4.2 CD4+ Cell Count and Viral Load Monitoring ... 31

4.3 Pregnancy and Breastfeeding ... 32

4.4 HIV Transmission Counseling ... 32

4.5 Study Withdrawal ... 33

4.6 Co-enrollment in Other Studies ... 33

5 EVALUATION ... 34

5.1 Data Analysis ... 34

5.2 Data Monitoring ... 35

6 PROTECTION OF HUMAN SUBJECTS & OTHER ETHICAL CONSIDERATIONS ... 38

6.1 Local Review of Protocol and Informed Consent ... 38

6.2 Ethical Conduct of the Study ... 38

6.3 Informed Consent of Study Participants 38

6.4 Confidentiality of Study Participants 38

7 OTHER IMPORTANT DOCUMENTS AND POLICIES 39

7.1 Reference Documents 39

7.2 Data Collection and Monitoring 39

7.3 Publications and Presentations 39

8 SUBSTUDIES 40

8.1 Genomics Substudy 41
8.1.1 Background and Rationale 41
8.1.2 Purpose 41
8.1.3 Study Design 41
8.1.4 Sample Size and Statistical Considerations 42
8.1.5 Participant Selection 42
8.1.6 Enrollment and Data Collection 42
8.1.7 Follow-up 42
8.1.8 Specimen Collection 42
8.1.9 Human Subjects 43
8.1.10 Confidentiality 43
8.1.11 Access to Stored Specimens Collected for the START Genomics Substudy 44
8.1.12 Access to Individual Test Results 44

8.2 Neurology Substudy 46
8.2.1 Background and Rationale 46
8.2.2 Methodology 48
8.2.3 Clinical Management Issues 55
8.2.4 Evaluation 57
8.2.5 Human Subjects 59

8.3 Informed Consent Substudy 60
8.3.1 Background and Rationale 60
8.3.2 Purpose 60
8.3.3 Study Hypothesis 61
8.3.4 Study Design 61
8.3.5 Eligibility Criteria for Sites 61
8.3.6 Randomization of Participating Sites 62
8.3.7 Data Collection 62
8.3.8 Differences between Standard Consent and Concise Consent for START 63
8.3.9 Study Outcome Measures 63

8.3.10 Sample Size Estimation 64
8.3.11 Data Analysis 65
8.3.12 Administration of the Questionnaire 66
8.3.13 Confidentiality of the Questionnaire Responses 66
8.3.14 Study Alternative for Sites and START Participants 66
8.3.15 Human Subjects 66
8.3.16 Benefits 66
8.3.17 Risks 66

APPENDICES

A. Sample Consent Forms
    A1. START Main Study Sample Consent
    A2. START Study Specimen Storage (Plasma & Urine) Sample Consent
    A3. START Study Specimen Storage (Biopsy Tissue & Blood) at Diagnosis of New Malignancy Sample Consent
    A4. START Genomics Substudy Sample Consent
    A5. START Genomics Substudy Sample Withdrawal Letter
    A6. START Neurology Substudy Sample Consent
    A7. START Informed Consent Substudy: Sample Concise Consent for Main Study
B. Summary of Background Data for START Design
C. Time and Events Schedule
D. START Protocol Team
E. Primary Endpoint Definition
F. Guidelines for Resistance Testing
G. Sample Size Justification
H. References on INSIGHT Website
I. List of Acronyms

REFERENCES

1 Synopsis

Purpose

The purpose of this randomized study is to determine whether immediate initiation of antiretroviral treatment (ART) is superior to deferral of ART until the CD4+ declines below 350 cells/mm^3 in terms of morbidity and mortality in HIV-1 (subsequently referred to as HIV) infected persons who are antiretroviral naïve with a CD4+ count above 500 cells/mm^3.

Rationale

Section 2 and Appendix B provide a detailed summary of the background and rationale for START. With the exception of data from a small subgroup of participants who were not taking ART at the time of entry into the Strategies for Management of AntiRetroviral Therapy (SMART) study, evidence from randomized trials does not exist to guide decisions about the initiation of highly active ART regimens for HIV-infected individuals with CD4+ cell counts > 200 cells/mm^3. Current guidelines are based largely on data from observational studies; some of these recommend initiating ART when the CD4+ cell count is ≤ 350 cells/mm^3, while others recommend initiating ART when the CD4 cell count falls below 500 cells/mm^3. In current practice, ART is usually not initiated until the CD4+ cell count is < 350 cells/mm^3 for several reasons:

- small absolute (as opposed to relative) risk differences in the rates of AIDS associated with earlier ART use and therefore uncertainty about the risk-benefit ratio;
- uncertainty about the cost-effectiveness of early ART even if the risk-benefit ratio is favorable;
- concerns about serious complications associated with ART, e.g., cardiovascular, renal, and liver disease, that might negate the expected benefits of ART;
- side effects of ART that impact quality of life;
- concern that there could be waning adherence with long-term use of ART and consequent development of HIV resistance; and
- the potential availability of even better treatments in the future that would be easier to take and would more durably suppress the virus and carry a lower risk of the development of resistance.

Recent data from epidemiological studies indicate that the risk of AIDS is graded and persists at CD4+ levels > 500 cells/mm^3. Furthermore, for a given CD4+ count, the risk of AIDS appears to be lower in patients who have started ART than in those who are ART-naïve. In addition, rates of serious non-AIDS diseases – cardiovascular, renal and hepatic disease and non-AIDS malignancies – are lower at higher CD4+ counts. Data from the SMART study, a treatment interruption trial that enrolled 5,472 participants with a CD4+ count > 350 cells/mm^3, are consistent with these epidemiological data. In SMART, there was a higher rate of both AIDS and non-AIDS events in the episodic compared to the continuous ART arm. This adverse treatment effect was evident among subgroups of participants on ART at entry, participants who

previously took ART but stopped before randomization, and participants who were ART naïve. However, the latter two subgroups of participants were small.

Collectively, these data indicate that morbidity and mortality risk reduction with earlier use of ART may be greater than previously estimated. Furthermore, it is possible that any increased risk of non-AIDS morbidity and mortality associated with use of ART may be more than counter-balanced by a reduced risk due to HIV RNA suppression, higher CD4+ count, or other beneficial effects of ART. If this is the case, the potential risk reduction for a composite outcome of AIDS and non-AIDS events could be sizable even in a target population at relatively low risk of AIDS. The reason for this is that the rate of non-AIDS events is much greater than AIDS events at higher CD4+ cell counts. By itself, a large percentage risk reduction in AIDS, as might be expected with early ART, might not be of sufficient magnitude in terms of absolute excess risk to warrant early intervention. However, if the reduction in risk for AIDS is coupled with a larger absolute risk reduction for serious non-AIDS events, as might be expected even if ART only had a modest positive effect on these higher incidence events, then early ART would be indicated.

While the potential for reducing morbidity and mortality with ART is great, current data available are insufficient and do not inform whether the benefits of initiating ART at CD4+ cell counts above 500 cells/mm^3 outweigh the risks. It is critical to evaluate risks and benefits of early ART with a randomized trial.

START Schematic

HIV-infected individuals who are ART-naïve with CD4+ count > 500 cells/mm^3

**Early ART Group**	**Deferred ART Group**
Initiate ART immediately following randomization<br><br>N=2,000	Defer ART until the CD4+ count declines to < 350 cells/mm^3 or AIDS develops<br><br>N=2,000

Design
START is an international randomized trial comparing early ART versus deferred ART. Participants will be randomized in a 1:1 allocation ratio to the early or deferred ART group. The primary composite endpoint is the development of a serious AIDS event ("AIDS*"), a serious non-AIDS event ("non-AIDS"), or death from any cause.

Serious AIDS events (or AIDS*) include most traditional opportunistic conditions but exclude non-fatal esophageal candidiasis and chronic *Herpes simplex* (see Appendix E for a complete list of conditions). Non-fatal esophageal candidiasis and chronic *Herpes simplex* are not counted in the primary endpoint of serious AIDS events because they are more common than most other opportunistic events at higher CD4+ counts and usually do not cause significant limitations for people in whom they occur.

In this protocol, the term "AIDS" (without an asterisk) denotes all opportunistic conditions, including non-fatal esophageal candidiasis and chronic *Herpes simplex* (see Appendix E). All AIDS conditions are included as a secondary endpoint in START.

The following serious non-AIDS conditions are the components of the primary composite endpoint referred to as serious non-AIDS events, or "non-AIDS":

- Cardiovascular disease (CVD) (myocardial infarction, stroke, coronary revascularization);
- End-stage renal disease (ESRD) (initiation of dialysis, renal transplantation);
- Decompensated liver disease; and
- Non-AIDS-defining cancers (excluding basal and squamous cell skin cancers).

Basal and squamous cell skin cancers are included as components of a secondary endpoint – all non-AIDS-defining cancers.

Key secondary endpoints are the components of the primary composite endpoint. Other important secondary endpoints include all-cause mortality, fatal or non-fatal AIDS, drug resistance, quality of life, health-care utilization and cost of care, and HIV transmission risk behavior. . Plasma will be stored to facilitate future biomarker comparisons between treatment groups and nested case-control studies.

In order to obtain definitive information concerning impact of early ART on the primary endpoint as compared to deferred ART, it is estimated that 4,000 participants will need to be randomized and followed for up to 6 years. Because there is uncertainty about the rates of AIDS* and non-AIDS in this target population, sample size, duration of follow-up and the target number of events will be re-estimated by the protocol team before enrollment is completed. This sample re-estimation will consider the overall (both treatment groups combined) primary event rate and the relative proportion of primary events that are AIDS* and non-AIDS.

Participant Selection
As a general guideline, participants who are considered for enrollment should be in reasonably good health. The participant should be able, in the clinician's opinion, to adhere to the protocol (i.e., be willing to accept and adhere to the data collection schedule and assigned treatment strategy).

Inclusion Criteria

- Signed informed consent
- HIV infection documented by a plasma HIV RNA viral load, rapid HIV test or any licensed[1] ELISA test; and confirmed by another test using a different method including but not limited to a rapid HIV test, Western Blot, HIV culture, HIV antigen, or HIV pro-viral DNA at any time prior to study entry.
- Age ≥ 18 years
- Karnofsky performance score ≥ 80 (an indication that the participant can perform normal activities)
- Perceived life expectancy of at least 6 months
- For women of child-bearing potential, willingness to use contraceptives as described in the product information of the ART drugs they are prescribed
- Two CD4+ cell counts > 500 cells/mm^3 at least 2 weeks apart within 60 days before randomization

[1] The term “licensed” refers to an FDA-approved kit or, for sites located in countries other than the United States, a kit that has been certified or licensed by an oversight body within that country. Confirmation of the initial test result must use a test method that is different than the one used for the initial assessment.

Exclusion Criteria

- Any previous use of ART or IL-2
- Diagnosis of any clinical AIDS event before randomization (including esophageal candidiasis and chronic *Herpes simplex* infection)
- Presence of HIV progression such as oral thrush, unexplained weight loss, or unexplained fever at randomization
- Cardiovascular event (myocardial infarction, angioplasty, coronary-artery bypass grafting, stroke) within 6 months before randomization
- Non-AIDS-defining cancer, excluding basal and squamous cell skin cancer, within 6 months before randomization
- Dialysis within 6 months before randomization
- Diagnosis of decompensated liver disease before randomization
- Current imprisonment, or compulsory detention (involuntary incarceration) for treatment of a psychiatric or physical illness
- Current pregnancy or breastfeeding (a negative serum or urine pregnancy test is required within 14 days before randomization for women of child-bearing potential)

Procedures

Within 60 days before randomization, participants will attend screening visits to establish eligibility and collect baseline data. Following randomization, participants who are assigned early ART will commence taking the ART regimen that was specified prior to randomization. All participants must receive a regimen listed on the INSIGHT website table “Antiretroviral Components Required for the Initial Regimen in START” based on U.S. Department of Health and Human Services (DHHS) guidelines. This table will be updated as guidelines change (see Appendix H). To ensure that participants receive a required regimen, a number of different ART regimens will be provided to sites through the INSIGHT Central ART Repository.

Following randomization, participants will be seen at 1 month, 4 months and every 4 months thereafter for follow-up data collection. Visits will include a targeted medical history, a brief medical examination, and determination of CD4+ cell count and HIV RNA level. Plasma will be stored at baseline and each follow-up visit for future HIV-related research.

ART will be prescribed for participants in the deferred ART group when the CD4+ cell count declines to < 350 cells/mm^3 and is confirmed within 4 weeks; when an AIDS-defining event develops; or when certain other conditions or symptoms occur as specified by local guidelines.

Participants in the deferred ART group who initiate ART must receive a regimen listed on the current INSIGHT website table “Antiretroviral Components Required for the Initial Regimen in START.”

Primary Endpoint

Time to AIDS*, non-AIDS, or death from any cause (first event) (see Appendix E)

Major Secondary Endpoints
- AIDS* or death from AIDS
- Non-AIDS or death not attributable to AIDS

Other Secondary Outcomes
- All-cause mortality
- Non-AIDS
- CVD (myocardial infarction, stroke, coronary revascularization)
- ESRD (initiation of dialysis, renal transplantation)
- Decompensated liver disease
- Non-AIDS malignancy, excluding basal and squamous cell skin cancers
- Non-AIDS malignancy, including basal and squamous cell skin cancers
- AIDS
- Bacterial pneumonia
- Adverse events
- Hospitalization
- Quality of life
- Health-care utilization and cost of care
- HIV transmission risk behavior
- HIV drug resistance
- Pulmonary embolism or deep vein thrombosis
- New-onset diabetes mellitus
- Coronary artery disease requiring drug treatment
- Congestive heart failure
- Peripheral arterial disease
- Change in estimated glomerular filtration rate (eGFR) and development of proteinuria
- Blood pressure and blood lipids
- Electrocardiogram (ECG) abnormalities
- Use of blood pressure- or lipid-lowering treatment or aspirin
- Fractures

2 Background and Rationale

2.1 Primary Study Hypothesis

Untreated HIV infection is associated with an increased risk of both AIDS* and non-AIDS events. Therefore, among asymptomatic participants with a CD4+ count greater than 500 cells/mm^3, immediate use of ART that results in suppression of HIV RNA levels and increases in CD4+ cell counts and potentially other beneficial effects will delay the development of AIDS*, non-AIDS, and death from any cause.

2.2 Background on Which Study Hypothesis Was Generated

2.2.1 Current evidence and practice on the timing of ART initiation

Evidence from randomized controlled trials support the initiation of ART before CD4+ cell count drops to less than 200 cells/mm^3.[1]. However, there are no data from randomized clinical trials to guide the decision on the optimal time to initiate ART in asymptomatic individuals with CD4+ cell count > 200 cells/mm^3. For this reason, treatment guidelines from around the world have relied on data from observational studies in making their recommendations. Some of these guidelines recommend initiating ART when the CD4+ cell count is ≤ 350 cells/mm^3,[2,3,4,5] , while others recommend initiating ART when the CD4+ cell count falls below 500 cells/mm^3.[6, 7]

Data from untreated cohorts indicate that the 6-month rate of AIDS continues to decrease with higher CD4+ counts in the 200-500 cells/mm^3 range and is much lower for individuals with a CD4+ cell count ≥ 350 than for individuals with counts 200-349 cells/mm^3.[8] Likewise, data from cohorts that follow patients after the initiation of ART indicate that the hazard of AIDS declines following initiation of ART, even at high CD4+ cell counts, e.g., > 350 cells/mm^3.[9] Taken together, these data suggest that initiation of ART at a CD4+ count > 350 cells/mm^3 would lead to a substantial relative reduction in AIDS events. However, among patients being followed in clinical practice, ART is usually not initiated until the CD4+ cell count is < 350 cells/mm^3 for several reasons: (1) small absolute (as opposed to relative) risk differences after 3 and 5 years in the rates of AIDS or death associated with earlier ART use,[10] and therefore uncertainty about the cost-effectiveness of early ART; (2) concerns about serious complications associated with ART, e.g., cardiovascular, renal, and liver disease, that might negate the expected benefits of ART; (3) side effects of ART that impact quality of life; (4) concern that there could be waning adherence with long-term use of ART and consequent development of HIV drug resistance; and (5) the potential availability of better treatments in the future that would more durably suppress the virus and carry a lower risk of the development of resistance.

Many of the reasons stated above led to the assumption that, even though earlier use of ART would likely reduce morbidity and mortality in the short term, in the long term it might not. This assumption was made in the absence of any data on clinical outcomes from randomized trials that could prove or disprove it.

More recent data indicate that the reasons for delaying ART are weaker than before and suggest that use of ART earlier (at higher CD4+ counts) than is currently practiced could be beneficial. Data from the SMART study, a clinical trial that randomized 5,472 participants to episodic (DC group) versus continuous ART (VS group), indicate that episodic use of ART according to CD4+ count thresholds (stop ART when the CD4+ count is > 350 and re-initiate ART when the CD4+ count declines to < 250 cells/mm^3) across a broad range of CD4+ cell counts is associated with increased risk of non-AIDS events such as cardiovascular, liver, and renal disease and non-AIDS-defining cancers.[11,12] Furthermore, when continuous ART was reinitiated for those in the episodic ART group, this increased risk of the non-AIDS events was reduced.[13] While most of the participants in SMART were taking ART at entry, the increased risk of non-AIDS diseases associated with deferring use of ART to a CD4+ cell count of 250 cells/mm^3 appears to apply to ART-naïve participants and others not taking ART at entry. For example, for the small subgroup of participants in SMART who were ART-naïve or who had not taken ART for at least 6 months prior to randomization, the HR (DC/VS) for non-AIDS events defined similarly to that proposed for START was 7.0 (95% CI: 1.6-31.4).[14] Data from cohorts also suggest that initiating ART at higher CD4+ counts than is currently practiced may be beneficial. In observational studies the rates of many non-AIDS events are higher among patients with lower CD4+ cell counts.[15,16,17] A recent review summarizes data on the effects of HIV on serious diseases other than AIDS.[18] In addition, the clinical effect of starting ART at a CD4+ cell count > 350 cells/mm^3 was investigated by two large observational studies using separate multi-cohort datasets. Findings from the first study suggested mortality was reduced by about 40% when ART was initiated at a CD4+ cell count between 350 and 500 compared to < 350 cells/mm^3 while mortality was almost halved if ART was initiated at a CD4+ cell count above 500 compared to between 350 and 500 cells/mm^3.[19] However, in the second study, no evidence of an increase in the risk of AIDS or death was found when ART was started at a CD4+ count above 450 compared to between 350 and 450 cells/mm^3.[20] Due to the low power of the SMART subgroup analysis and the potential for bias (e.g., confounding by indication) in the cohort studies, the data cited above suggest, but do not prove, that treatment of individuals with high CD4+ counts could have a substantial beneficial effect. This can only be firmly established by a large, randomized clinical trial.

On the other hand, cohort studies also indicate that the risk of CVD may increase with use of some ART.[21,22,23,24] These apparently inconsistent findings from the SMART and the D:A:D studies could both be true – immunodeficiency, pro-inflammatory effects of untreated HIV or other factors may be associated with an increased risk of CVD while use of some ART may also increase the risk of CVD. Until the risks and benefits of ART with respect to CVD and other major clinical outcomes are quantified in a randomized trial, uncertainty will remain as to the merits of initiating ART at CD4+ counts > 350 cells/mm^3.

In order to quantify reliably the risk and benefits of ART at CD4+ counts > 350 cells/mm^3, it is important to define strategies for using ART that are clearly distinguishable and for which there is substantial uncertainty about the risk difference. Once defined, risks and benefits of the ART strategies can be reliably determined by randomly allocating a large number of participants to each strategy. In the section that follows, the potential risks and benefits of the two strategies are summarized. A more detailed review of the data on potential risks and benefits is provided in Appendix B.

2.2.2 Summary of Potential Risks and Benefits

For prolonging disease-free survival, potential benefits of an early treatment strategy of initiating ART at a CD4+ cell count > 500 cells/mm^3 are:

- Maintenance of a higher CD4+ cell count
- Lower risk of AIDS and non-AIDS conditions

Potential risks of the early ART strategy are:

- More side effects
- Increased risk of exhausting drug options (e.g., due to resistance)
- Increased risk of some non-AIDS events.

For prolonging disease-free survival, potential benefits of a deferred ART strategy that stipulate that ART be initiated when the CD4+ cell count declines to < 350 cells/mm^3 are:

- Drugs that are safer, easier to take, and more potent and/or less susceptible to resistance development could be available when ART is eventually initiated
- Delay in any risk of adverse events of ART

Potential risks of this strategy include:

- Longer period of time at lower CD4+ cell counts and higher HIV RNA levels
- Higher risk of AIDS and serious non-AIDS conditions while ART is deferred

In addition to these potential benefits and risks of early ART for patients with a CD4+ cell count > 500 cells/mm^3, there is a potential broader public health benefit of early ART. As noted in Appendix B, even if transmission risk behaviors do not decrease among patients taking early ART, reduced HIV transmission would be predicted based on HIV RNA decline with ART.

2.3 Rationale for Selected Study Design

2.3.1 CD4+ Cell Count Entry Criteria and CD4+ Count Deferral Criteria for Control Group

The immediate and deferred ART strategies need to be sufficiently different in their definition so that a difference in clinical outcomes between them is plausible. Thus, the CD4+ cell count entry and deferral criteria were selected to result in groups with substantially different exposure to ART over the planned follow-up period.

At entry, participants will have a CD4+ count > 500 cells/mm^3. ART will be initiated in the deferred ART group when the CD4+ declines to < 350 cells/mm^3. The deferral strategy was chosen with consideration of current practice and recent data from SMART[11,14] that indicate that initiation of ART should take place closer to 350 than 200 cells/mm^3. If the majority of participants enrolled have a CD4+ cell count between 500 and 650 cells and CD4+ declines on average 50-60 cells/mm^3 per year in the absence of ART, the majority of participants in the deferred ART group are likely to remain off ART for at least 2-3 years of follow-up.[25]

As a consequence of the CD4+ entry and ART deferral criteria, and considering the rate of CD4+ increase following initiation of ART and the magnitude of the increase starting at lower versus higher CD4+ levels, these two strategies for initiating ART will result in treatment groups with substantially different CD4+ cell counts and HIV RNA levels over a 5-year follow-up period.

2.3.2 Broad Entry Criteria

With few exceptions, any healthy HIV-infected adult with a CD4+ cell count > 500 cells/mm^3 at two visits within 60 days before randomization who has not taken ART or interleukin-2 (IL-2) is eligible to enroll. Participants may have had a prior CD4+ count ≤ 500 cells/mm^3, and this will be recorded. The participant may have any HIV RNA level. HIV RNA level predicts the rate of CD4+ decline.[26,27] Thus, on average, participants with higher HIV RNA levels in the deferred ART group are likely to meet the CD4+ threshold for ART (350 cells/mm^3) more rapidly than those participants with lower HIV RNA levels. Restricting enrollment to participants with high HIV RNA could result in less distinguishable treatment groups in terms of ART use and miss an opportunity to assess whether risk of serious non-AIDS diseases associated with HIV RNA, even at low levels, can be reduced with ART. While excluding participants with high HIV RNA levels would, on average, increase the difference in ART exposure between groups, HIV RNA does not explain a large fraction of the variability in the CD4+ decline.[27] Thus, rapid decline in CD4+ is still likely for some participants. The exclusion of participants with high HIV RNA would eliminate a large subgroup of participants for which there is uncertainty about when to start ART and limit ability for risk stratification. These broad entry criteria are likely to result in the inclusion of a group of participants at variable risk for the composite endpoint. This will permit risk stratification to be carried out at the completion of the study to determine how ART should most effectively be used in subgroups considering absolute risk reductions and cost effectiveness.

2.3.3 Composite Endpoint of AIDS* and Non-AIDS

The ultimate goal of the treatment of HIV disease is to prevent morbidity and mortality. Thus, the primary and secondary endpoints of this study are clinical outcomes rather than surrogate markers.

The primary endpoint of the START study is a composite outcome of AIDS*, non-AIDS, and death from any cause (see Appendix E). This composite outcome includes major morbid events that are life threatening and impact quality of life.

It is hypothesized that ART will positively influence both AIDS* and non-AIDS risk in the CD4+ range to be studied. Based on epidemiological studies, ART is expected to reduce the risk of AIDS* more than the risk of non-AIDS. However, the incidence of non-AIDS is expected to be greater than the incidence of AIDS*. Thus, the absolute risk reduction is expected to be similar for AIDS* and non-AIDS events.

2.3.4 Major Secondary Outcomes

2.3.4.1 Secondary Clinical Outcomes

A number of secondary outcomes will be assessed at interim and final analyses to allow for the careful weighing of the benefits and risks of early ART. These clinical outcomes include the separate components of the composite outcome; all-cause mortality; all AIDS events (including esophageal candidiasis and chronic *Herpes simplex*); all non-AIDS-defining cancers (including basal and squamous cell skin cancers); bacterial pneumonia; pulmonary embolism; deep vein thrombosis; new-onset diabetes mellitus; coronary artery disease requiring drug treatment; congestive heart failure; peripheral vascular disease; fractures; and adverse events.

2.3.4.2 Quality of Life, Health-Care Utilization and Cost of Care

Quality of life will be assessed using the self-administered Medical Outcomes Study Short-Form 12 Item Survey (SF-12, version 2) and a visual analog scale for current health (zero to 100, with zero being worst possible health).[28,29,30] A comparison of the costs and cost effectiveness of early versus deferred ART will be a critical component of the START study. The eventual implications of START for HIV care may rest on cost-effectiveness analysis. The majority of the costs of medical care among study participants will probably be those of ART, because hospitalizations and other expensive clinical interventions (e.g., nursing home care) are predicted to be quite uncommon in the study population. ART costs will be assessed from detailed information on usage in the two study arms and can be compared in a number of sensitivity analyses using different drug pricing systems (e.g., Average Wholesale Price [AWP], Public Health Service [PHS] price, and generic price). Geographic differences will be considered. Non-ART drugs will likely be only 5% of ART costs, and therefore, will not be collected in detail. Health-care utilization (hospitalization, clinic visits, nursing home care, and home care) will be reported and then used to estimate costs. Indirect costs of medical illness will also be estimated.

2.3.4.3 Transmission Risk Behavior

Information will also be collected on high-risk behaviors for transmitting HIV. Epidemiological data indicate that risk of sexual transmission of HIV is closely related to plasma viral load.[31,32] Thus, early ART may effectively lower risk of transmission if risk behaviors are similar in the two treatment groups.

2.3.4.4 HIV Drug Resistance

The development of resistance to ART and possible subsequent loss of drug options will be assessed as an important secondary outcome in the study. This outcome will be

evaluated by collecting samples of blood prior to changes in ART due to elevated HIV RNA level and by collecting results from locally performed resistance tests. The former will be used to carry out batch resistance testing at a central laboratory during the trial. These tests will not be done in real time, and results will not be given to the investigator or participant. Key mutations that are associated with viral resistance will be determined using information periodically updated by the International AIDS Society.[33] The early and deferred ART groups will be compared for the accumulation of major mutations to each class of ART.

2.3.4.5 Markers of CVD risk

Assessments will be made of blood lipids, smoking, blood pressure, resting electrocardiographic (ECG) abnormalities, incidence of diabetes mellitus, use of medication to lower blood pressure and lipids, and the use of aspirin, to assist in the evaluation of cardiovascular risks and benefits of early treatment. For all consenting participants, plasma will be stored for central measurement of inflammatory and coagulation markers.

2.4 Summary

For HIV-infected individuals with a CD4+ cell count > 500 cells/mm^3 who are not taking ART, there is genuine uncertainty whether the risk of morbidity and mortality from non-AIDS conditions that may result from ongoing viral replication and consequent general immune activation or immunodeficiency at higher CD4+ levels can be modified with ART. The findings from SMART suggest that risks of AIDS* and non-AIDS events could be reduced by initiating ART among patients at CD4+ cell counts > 350 cells/mm^3. However, it is unclear whether the findings from SMART can be generalized to ART-naïve patients or to patients with counts > 500 cells/mm^3. Epidemiological studies also suggest that reduced risks of AIDS and of some non-AIDS conditions would result from early ART. However, as in other illnesses, it is hazardous to assume that people's risk of disease can be modified with ART as predicted by the epidemiological data.

The risk of non-AIDS events is higher than the risk of AIDS* events in these patients. START will, therefore, assess these risks and determine the effectiveness of early ART in reducing the rate of a composite outcome that includes both AIDS* and non-AIDS. START will expand our understanding of how immunodeficiency and immune activation influence the risk of development of both AIDS* events and, more importantly, non-AIDS events at high CD4+ levels.

If START establishes that ART is beneficial among individuals with a CD4+ cell count > 500 cells/mm^3 for a broad range of health outcomes, the earlier use of ART in many parts of the world would be based on cost-effectiveness analyses, based both on the effectiveness of ART in the patients being treated and any effects in reducing the rate of ongoing transmission.

The potential for large reductions in morbidity and mortality with early ART is great; there is substantial uncertainty as to whether the risk reduction suggested by the epidemiological data and SMART results will be realized; and there is a high probability

of being able to enroll and follow patients and carry out the planned early intervention. Therefore, the timing for a randomized trial is ideal.

3 Methodology

3.1 Study Design

This is a multicenter, international, randomized trial for HIV-infected adults comparing initiation of ART at a CD4+ cell count > 500 cells/mm^3 (early ART) versus initiation of ART at a CD4+ cell count of < 350 cells/mm^3 (deferred ART) for a composite outcome of AIDS*, non-AIDS, and death from any cause (see Appendix E).

In addition to the composite outcome, two important secondary outcomes will be evaluated: (1) AIDS* or death from AIDS; and (2) non-AIDS or death not attributable to AIDS, including death of unknown cause. Other secondary outcomes include components of the major endpoints, HIV drug resistance, quality of life, cost effectiveness, and HIV transmission risk behaviors.

With few exceptions, consenting, asymptomatic, ART-naïve participants 18 years of age or older with a CD4+ cell count > 500 cells/mm^3 are eligible for randomization to the trial.

Participants in the early ART arm will commence ART immediately following randomization and continue taking it for the duration of the trial. Participants in the deferred ART group, the control arm, should commence treatment when the CD4+ declines to < 350 cells/mm^3 or if an AIDS-defining diagnosis occurs. In addition, when certain other conditions or symptoms occur as specified by local guidelines, ART may be initiated. In this study, when the CD4+ declines to < 350 cells/mm^3 for a participant in the deferred arm, the CD4+ count will be immediately confirmed (within 4 weeks), and if the second CD4+ count is also < 350 cells/mm^3, ART will be initiated.

For participants in both treatment groups, the initial regimen used must be chosen from those listed on the INSIGHT website table “Antiretroviral Components Required for the Initial Regimen in START.” This table is based on current DHHS guidelines and will be updated as guidelines change (see Appendix H).

3.2 Study Objectives

3.2.1 Primary Objective

To determine whether early ART is superior to deferred ART in delaying the occurrence of a composite outcome consisting of AIDS*, non-AIDS, or death from any cause.

3.2.2 Secondary Objectives

a. To compare early ART to deferred ART for each component of the primary composite endpoint:
   - AIDS* or death from AIDS
   - Non-AIDS or death not attributable to AIDS

b. To compare early ART to deferred ART for the following secondary outcomes:
   - All-cause mortality
   - Non-AIDS
   - CVD (myocardial infarction, stroke, coronary revascularizaton)
   - ESRD (initiation of dialysis, renal transplantation)
   - Decompensated liver disease
   - Non-AIDS malignancy, excluding basal and squamous cell skin cancers
   - Non-AIDS malignancy, including basal and squamous cell skin cancers
   - AIDS
   - Bacterial pneumonia
   - Adverse events
   - Hospitalization
   - Quality of life
   - Health-care utilization and cost of care
   - HIV transmission risk behavior
   - HIV drug resistance
   - Pulmonary embolism or deep vein thrombosis
   - New-onset diabetes mellitus
   - Coronary artery disease requiring drug treatment
   - Congestive heart failure
   - Peripheral arterial disease
   - Change in estimated GFR and development of proteinuria
   - Blood pressure and blood lipids
   - ECG abnormalities
   - Use of blood pressure- or lipid-lowering treatment or aspirin
   - Fractures

c. To compare early ART with deferred ART for the primary composite outcome and other major clinical outcomes in subgroups defined by the following characteristics measured at baseline:
   - Age
   - Gender
   - Race/ethnicity
   - Presence and levels of risk factors for serious non-AIDS conditions (e.g., smoking, estimated GFR (eGFR), hepatitis co-infection, diabetes mellitus, estimated CVD risk, lipids, blood pressure, presence of resting ECG abnormalities, age, and gender)
   - Baseline CD4+ cell count
   - Baseline HIV RNA level
   - Geographic region
   - Calendar date of enrollment
   - ART regimen specified prior to randomization

3.2.3 Other Objectives

For a robust comparison of the early and deferred ART groups, the groups must differ by a substantial amount in exposure to ART during follow-up. The first three objectives listed below are aimed at understanding whether protocol assumptions are valid and at evaluating adherence to the protocol.

a. To compare the early and deferred ART groups for:
   - ART use over follow-up
   - HIV RNA levels over follow-up
   - CD4+ cell counts over follow-up

b. To describe the early and deferred groups with respect to:
   - Initial ART regimen used
   - HIV RNA and CD4+ cell count response to the initial ART regimen
   - Number of changes in ART regimen over all follow-up and during the first year of treatment
   - Non-adherence to treatment strategy (e.g., discontinuation of ART in early ART group or initiation of ART too early in the deferred group)

c. Among participants in the deferred group, to estimate the rate of decline of CD4+ cell counts until the CD4+ declines to < 350 cells/mm^3, to estimate the fraction of participants who develop a primary event prior to starting ART, to estimate time to first ART initiation, and to estimate the fraction of participants who initiate ART before the CD4+ declines to < 350 cells/mm^3

d. Among participants in both treatment groups, to study predictors of AIDS* and non-AIDS

e. To estimate the prevalence and levels of non-AIDS risk factors at baseline (e.g., smoking, eGFR, hepatitis co-infection, diabetes mellitus, estimated CVD risk, lipids, blood pressure, presence of resting ECG abnormalities, age, and gender)

f. To evaluate the informed consent process by comparing comprehension of study requirements for those consented with a concise versus standard consent (in a subset of sites)

3.3 Primary Study Endpoint

The primary composite endpoint is defined in Appendix E and includes the following three major components:

- AIDS* or death from AIDS
  Opportunistic events consistent with the 1993 CDC expanded surveillance definition plus additional events associated with immunosuppression in the patient population targeted for enrollment. Esophageal candidiasis and chronic *Herpes simplex* infection will be counted as primary endpoints only if they result in death.

- Non-AIDS
    - o CVD: myocardial infarction, stroke, coronary revascularization
    - o ESRD: initiation of dialysis, renal transplantation
    - o Decompensated liver disease
    - o Non-AIDS-defining cancers, excluding basal and squamous cell skin cancers. Basal and squamous cell skin cancer will be counted as a primary endpoint only if they result in death.

- Death not attributable to AIDS, including death of unknown cause

The primary outcome of START and each of the major components of the primary outcome will be evaluated as the time to the first occurrence of an event above. Other major endpoints are referred to in the Study Objectives (section 3.2).

3.4 Randomization

Eligible participants will be randomized in a 1:1 ratio to either the early ART group or the deferred ART group. Randomization will be stratified by clinical site.

3.5 Sample Size and Statistical Considerations

It is estimated that 4,000 participants will be required to reliably address the primary study question. This is based on estimates of the expected event rates in the early and deferred ART groups using data from the CASCADE Collaboration and UK CHIC[34] with computer simulations that account for the distribution of CD4+ cell counts projected for START. These estimates and other assumptions used in calculating sample size are described in detail in Appendix G and summarized below:

a. The primary analysis will be intention to treat using a Cox model with a single indicator for treatment group and with strata corresponding to geographic regions (North America, South America, Europe, Australasia, and Africa).

b. Type I error is 0.05 (2-sided) and power = 0.90. Power was set at 90% for the primary endpoint, in part, to ensure that there would be adequate power to address the components of the composite endpoint, non-AIDS and fatal AIDS or non-fatal AIDS*.

c. Participants will be enrolled over a 3-year period (2 additional years after the one year pilot phase) and followed for a minimum of 3 years resulting in an average follow-up of 4.5 years and a total study duration of 6 years.

d. The CD4+ cell count at entry will be between 501 and 600 cells/mm^3 for 70% of participants, between 601 and 700 cells/mm^3 for 20%, and > 700 cells/mm^3 for 10%.

e. In the deferred ART group, the rate of the primary endpoint is 2.8 per 100 person years over the follow-up period. Non-AIDS events will be four times more common than non-AIDS deaths, and approximately 35% of reported AIDS events and 10% of

reported non-AIDS events will not meet probable or confirmed criteria (i.e., the criteria that have to be achieved to count the reported event as an endpoint). In SMART, the rates of confirmed or probable fatal AIDS or non-fatal AIDS* and of serious non-AIDS, as defined in START, were 0.4 and 2.0 per 100 person-years, respectively, in the VS group.[35]

f. Based on the computer simulations described in Appendix G, early ART is predicted to reduce the primary endpoint rate by 28.8% compared to deferred ART. This reduction in the hazard assumes: (1) AIDS* events represent 23% of the events in the deferred arm, and early ART will reduce this hazard by 43%; and (2) non-AIDS events and deaths not attributable to AIDS will represent 77% of events in the deferred ART group, and early ART will reduce this hazard by 24%. The smaller percentage risk reduction anticipated for non-AIDS events and deaths not attributable to AIDS takes into account that some of the deaths will be due to causes that are unrelated to HIV and ART. For example, 18% of deaths in SMART were due to substance abuse, accidents or violence.[11]

g. The estimated treatment differences take into account likely levels of adherence to the early ART strategy (e.g., participants followed in CASCADE who started ART were considered "on ART" irrespective of adherence or future discontinuation of their ART).

h. Adherence to the deferred strategy was also considered. It was assumed that 70% of participants would adhere to the deferral CD4+ threshold of 350 cells/mm^3 and 30% would initiate ART earlier – 10% before the CD4+ declined to 400 cells/mm^3 and 20% while the CD4+ was between 350 and 400 cells/mm^3.

i. A loss to follow-up rate of 2.7 per 100 person-years (equivalent to a 15% cumulative lost to follow-up after 6 years) is assumed.

j. Based on these assumptions, 3,822 participants (1,911 participants in each treatment group) are required. The number of primary events required is 369.

k. While many of the underlying assumptions are conservative, for some assumptions there is much uncertainty because of the absence of data (e.g., inadequate or no data on rates of non-AIDS morbidity). Considering this, a sample size of 4,000 participants (2,000 in each treatment group) and a target of 370 primary events has been established as the initial goal. This may be modified following sample size re-estimation.

Power was also estimated for the components of the primary endpoint. With the above assumptions, and with 4,000 participants and 370 primary events, power is 0.74 to detect a 43% reduction in the hazard of AIDS*, and power is 0.73 to detect a 24% reduction in the hazard of non-AIDS and deaths not attributable to AIDS.

3.6 Participant Selection

A heterogeneous group of participants with respect to baseline characteristics is important in a trial like START, and the broad inclusion and exclusion criteria specified below reflect that. It will permit risks and benefits of early versus deferred ART to be assessed in a variety of participants and will ensure that the trial reflects as much as possible the type of patients seen in clinical practice for whom there is uncertainty about when to begin lifelong ART.

As a general guideline, individuals who are considered for enrollment should be in reasonably good health. They should be able, in the clinician's opinion, to adhere to the protocol (i.e., be willing to accept and adhere to the data collection schedule and the assigned treatment strategy).

3.6.1 Inclusion Criteria

- Signed informed consent
- HIV infection documented by plasma HIV RNA viral load, a rapid HIV test or any licensed[1] ELISA test; and confirmed by another test using a different method, including but not limited to a rapid HIV test, Western Blot, HIV culture, HIV antigen, or HIV pro-viral DNA at any time prior to study entry.
- Age $\geq$ 18 years
- Karnofsky performance score ≥ 80 (an indication that the participant can perform normal activities)
- Perceived life expectancy of at least 6 months
- For women of child-bearing potential, willingness to use contraceptives as described in the product information of the ART drugs they are prescribed
- Two CD4+ cell counts > 500 cells/mm^3 at least 2 weeks apart within 60 days before randomization

3.6.2 Exclusion Criteria

- Any previous use of ART or IL-2
- Diagnosis of any clinical AIDS event before randomization (including esophageal candidiasis and chronic *Herpes simplex* infection)
- Presence of HIV progression such as oral thrush, unexplained weight loss, or unexplained fever at randomization
- Cardiovascular event (myocardial infarction, angioplasty, coronary-artery bypass grafting, stroke) within 6 months before randomization

[1] The term “licensed” refers to an FDA-approved kit or, for sites located in countries other than the United States, a kit that has been certified or licensed by an oversight body within that country. Confirmation of the initial test result must use a test method that is different than the one used for the initial assessment.

- Non-AIDS-defining cancer, excluding basal and squamous cell skin cancer, within 6 months before randomization
- Dialysis within 6 months before randomization
- Diagnosis of decompensated liver disease before randomization
- Current imprisonment, or compulsory detention (involuntary incarceration) for treatment of a psychiatric or physical illness
- Current pregnancy or breastfeeding (a negative serum or urine pregnancy test is required within 14 days before randomization for women of child-bearing potential)

3.7 Study Plan

The management of participants throughout the course of the trial will be guided by their assigned strategy. Throughout follow-up, when ART is used, it should be a potent combination ART regimen that is expected to provide durable suppression of HIV RNA levels.

3.7.1 Treatment Strategies

Before a participant is randomized, a potent combination ART regimen must be prespecified by the study clinician. The initial regimen used must be one from the table "Antiretroviral Components Required for the Initial Regimen in START" on the INSIGHT website (see Appendix H). The list of regimens in this table is based on current DHHS guidelines and will be updated following any changes in those guidelines.

After randomization, participants in the two groups will use potent combination ART as follows:

a. Participants assigned to the **early** ART group will start the prespecified ART regimen as soon as possible.

b. Participants assigned to the **deferred** ART group will defer ART until one of the following conditions occurs:
   - CD4+ cell count declines to < 350 cells/mm^3 and is confirmed by a repeat CD4+ measurement within 4 weeks, or
   - AIDS develops, or
   - Conditions specified by local guidelines occur that indicate initiation of potent combination ART, e.g., symptoms indicative of HIV progression such as oral thrush, unexplained weight loss, or unexplained fever.

   The intent of the deferral strategy used in START is to initiate ART as soon as possible after the CD4+ count drops below 350 cells/mm^3. Thus, CD4+ count should be monitored closely as it approaches 350 cells/mm^3 (see section 4.2).

   After discussion with their clinicians, participants in the deferred ART group who develop any of the conditions noted above will start an ART regimen selected from the table "Antiretroviral Components Required for the Initial Regimen in START" on the INSIGHT website (see Appendix H). The regimen selected may differ from the

regimen prespecified for these participants prior to randomization, but should be started as soon as possible within 4 weeks of confirmation of any of the conditions noted above.

Women assigned to the **deferred** ART group who become pregnant before the CD4+ cell count declines to < 350 cells/mm^3 may be prescribed ART as recommended by local treatment guidelines, even if the ART regimen chosen differs from those listed on the INSIGHT website table.

More information on choice and management of ART and provision of antiretroviral drugs throughout the trial is found in section 4.1 below.

3.7.2 Concomitant Medications

There are no restrictions on concomitant medications for participants in either group. However, it is important to consider the potential for drug interactions between ART and concomitant medications, including agents prescribed for prophylaxis against opportunistic infections. Links to current information on drug interactions can be found on the INSIGHT website (see Appendix H).

The use of targeted categories of concomitant medications prescribed for participants in START will be ascertained at baseline and annual follow-up visits. Targeted categories will include but are not limited to:

- Agents for prophylaxis against opportunistic infections
- Lipid-lowering drugs
- Drugs for the treatment of diabetes mellitus
- Antihypertensive agents
- Other cardiovascular drugs, including aspirin
- Hormones (used for contraception or for therapy)
- Corticosteroids (used at doses above physiologic replacement)
- Immunomodulators

3.7.3 Baseline Screening

All consenting participants will have the following information and measurements collected within 60 days before randomization unless otherwise noted. All measurements are done locally unless otherwise noted.

- Demographics, including education
- Documentation of HIV infection
- CD4+ cell count and CD4%: two measurements at least 2 weeks apart, with the earliest within 60 days before randomization
- Karnofsky score
- For women of child-bearing potential, a pregnancy test (serum or urine) done in the clinic must be documented to be negative within 14 days before randomization
- Targeted health history including date of first diagnosis of HIV infection, likely mode of HIV infection, history of non-AIDS events, pregnancy status, and history of fractures

- Brief clinical evaluation including weight, height, sitting blood pressure, pulse, and smoking status
- Nadir CD4+ cell count and CD4% and maximum HIV RNA level available in the medical record from any time in the past
- Up to three most recent (before the above baseline measurements) CD4+ cell counts, CD4%s, and HIV RNA measurements available in the medical record
- Findings from previous genotypic or other form of HIV resistance testing (such as virtual phenotype and/or phenotypic resistance testing), if performed and available (see section 4.1.9 and Appendix F)
- Selected concomitant medications
- Quality of life assessment
- Use of alcohol and recreational drugs
- HIV transmission risk behavior assessment
- Health-care utilization
- HIV RNA measurement
- Additional laboratory assessments (participants should be asked to abstain from food, except water, for at least 8 hours prior to providing blood for glucose and lipid measurements):
  - Complete blood count (CBC): hemoglobin, hematocrit, white blood cell count (WBC) with differential and platelets
  - CD8+ T-cell count and CD8%
  - Renal function: serum creatinine to estimate GFR[36]
  - Liver function: alanine aminotransferase (ALT), aspartate aminotransferase (AST), alkaline phosphatase, total bilirubin and albumin
  - Glucose
  - Lipids: total cholesterol, low density lipoprotein (LDL), high density lipoprotein (HDL), triglycerides
  - Dipstick urinalysis for measurement of protein
- Documentation of hepatitis B and C status: hepatitis B surface antigen, core antibody and surface antibody; hepatitis C antibody and, if available, genotype and viral load. Documented positive tests at any time in the past or documented negative tests in the 6 months before randomization may be used.
- In a subset of participants, a resting ECG (at sites with a study-provided ECG machine and a certified technician)
- ART regimen to be prescribed for the participant if randomized to the early group and whether the regimen will be obtained locally or through the INSIGHT Central ART Repository
- Stored plasma (sufficient for eight 1-mL transport tubes) for future HIV-related research (e.g., HIV resistance testing, CVD biomarkers)
- Stored urine (sufficient for six 1-mL transport tubes) for future HIV-related research

In participants who will be prescribed abacavir, HLA-B*5701 screening test results for abacavir hypersensitivity must be available before prescription.

3.7.4 Participant Follow-up

Participants will be seen for routine follow-up clinical evaluation in accordance with standard INSIGHT procedures and schedules. The total duration of the trial is estimated to be 6 years (a 3-year enrollment period with participants followed for a minimum of 3 years after the last participant is enrolled). All participants will be followed to a common closing date that will be determined to coincide with attainment of the target number of primary endpoints. The follow-up period may be extended following a sample size re-estimation that will be performed before the end of the planned enrollment.

The follow-up schedule for data collection is the same in both groups. Data collection visits will occur at 1 and 4 months after randomization, and every 4 months thereafter until the study closes. In addition to the data collection described below for scheduled data collection visits, certain events that are described in section 3.8 are to be reported as soon as clinical sites become aware of them.

a. The following information will be collected at every follow-up visit unless otherwise noted:
   - Targeted health history and brief clinical evaluation including weight, sitting blood pressure and pulse
   - ART regimen currently prescribed, if applicable
   - Laboratory assessments:
     - CD4+ cell count, CD4% and HIV RNA level, CD8+ T-cell count and CD8%
     - Interim CD4+ cell counts, CD4% and HIV RNA levels obtained since last visit
   - Results of locally performed antiretroviral resistance tests, if available
   - Self-reported adherence to ART
   - Stored plasma (sufficient for four 1-mL transport tubes) for future HIV-related research

b. The following additional information will be collected at months 1, 4 and 8 and every 12 months after randomization:
   - Laboratory assessments:
     - Renal function: creatinine
   - Urine dipstick for proteinuria
   - Stored urine (sufficient for six 1-mL transport tubes) for future HIV-related research

c. The following additional information will be collected at month 4 and every 12 months after randomization:
   - Quality of life assessment
   - HIV transmission risk behavior assessment

d. The following additional information will be collected every 12 months after randomization:
   - Smoking status
   - Selected concomitant medications

- New medical diagnoses
- Laboratory assessments (participants should be asked to abstain from food, except water, for at least 8 hours prior to providing blood for glucose and lipid measurements):
    - CBC: hemoglobin, hematocrit, WBC with differential and platelets
    - Liver function: ALT, AST, alkaline phosphatase, total bilirubin and albumin
    - Glucose
    - Lipids: total cholesterol, LDL, HDL, triglycerides
- In a subset of participants, a resting ECG (at sites with a study-provided ECG machine and a certified technician)
- Health-care utilization
- Use of alcohol and recreational drugs
- Pregnancy status
- History of HLA-B*5701 testing since the last annual visit (since randomization at Month 12)

e. The following additional information will be collected during follow-up as it occurs:

- Changes in the ART regimen and reasons
- Documentation of clinical events, death, and pregnancy outcomes (see section 3.8)
- For consenting participants with a newly diagnosed malignancy, a biopsy sample of malignant tissue (a fresh frozen sample or paraffin block, pathology slide(s), if available). A 20-mL whole blood sample obtained prior to treatment of the malignancy, if available, will also be collected for consenting participants in the U.S.
- Each time the ART regimen is switched or stopped due to an elevated HIV RNA level (or presence of resistance mutations), a 4-mL plasma sample for later central batch HIV resistance testing. The plasma sample should be collected prior to changing the ART regimen.
- Pregnancy outcomes (and relevant history) for female participants who become pregnant after randomization.

Most of the procedures above apply to follow-up visits for the purpose of data collection. More frequent follow-up visits may be conducted to ensure participant safety or for the purposes of providing routine care.

3.7.5 Participant Relocation to a New Site

If a participant relocates to a site not participating in START, data collection of items that are considered to be part of routine care (e.g., HIV RNA level, CD4+ cell count, clinical events) will continue if the participant provides a signed release of information. The *START Protocol Instructions Manual* provides guidance on how to collect data for participants who relocate and/or transfer to another START clinical site.

3.7.6 Stored Samples and Future Research

Specimens will be collected before randomization, at follow-up visits, and at the occurrence of events described above. These specimens will be stored at a central specimen repository for later use in HIV-related research concerning the effects of HIV and ART on AIDS and non-AIDS conditions. Proposed research utilizing these specimens will be reviewed and approved by the INSIGHT Executive Committee. Results of research tests on individual specimens will not be given to participants or their clinicians, but aggregate research results will be made available.

Instructions for drawing, processing, storing and shipping specimens are in the *START Laboratory Manual*.

3.8 Event Reporting

For both treatment groups, for the duration of the trial, certain events are to be reported as soon as clinical sites become aware of them. The case report forms and documentation requirements are found in the *START Protocol Instructions Manual*. The INSIGHT Endpoint Review Committee (ERC) has established objective criteria for each event comprising the primary endpoint and for major secondary endpoints; these criteria are also found in the *START Protocol Instructions Manual.* The ERC is responsible for reviewing each reported primary event to determine the level of diagnostic certainty. Events which are judged as confirmed or probable will be included in the primary analysis.

All major clinical events, including the primary endpoint and its components, regardless of relationship to ART, will be summarized for the Data and Safety Monitoring Board (DSMB).

All events described below, including all AIDS and non-AIDS events that a participant experiences will be reported from study enrollment until study completion.

3.8.1 AIDS and Non-AIDS Events

Events comprising the primary endpoint of the START study will be reported on case report forms for participants in both treatment groups for the duration of the trial, irrespective of changes in ART use. In addition, AIDS events that are not part of the primary endpoint must also be reported, namely non-fatal esophageal candidiasis and *Herpes simplex* infection lasting more than 30 days.

Similarly, all non-AIDS-defining events that are part of the primary endpoint must also be reported for all participants for the duration of the trial. In addition to the non-AIDS events included as components of the primary endpoint, bacterial pneumonia, pulmonary embolism, deep vein thrombosis, new-onset diabetes mellitus, coronary artery disease requiring drug treatment, congestive heart failure, and peripheral arterial disease must also be reported.

All events should be reported immediately after a working diagnosis of the event has been made. All events that a participant experiences during the study will be reported. This includes recurrences of previously reported diagnoses.

3.8.2 Deaths

Death from any cause is part of the primary endpoint of START. Deaths must be reported on case report forms for all study participants for the duration of the trial. Deaths must be reported immediately following site awareness that a study participant has died.

3.8.3 Pregnancy Outcomes

For women who become pregnant during the study, pregnancy outcomes will be reported on a case report form.

3.8.4 Reporting Serious Events

Selected serious events are reported on a specific case report form, even if they have also been reported on other event-specific case report forms. If these events are judged by the investigator to be related to ART and unexpected according to ART labeling, they will be reported in an expedited manner. These events are:

- Deaths
- Events that are life-threatening
- Events requiring hospitalization
- Events requiring prolongation of hospitalization
- Events resulting in significant disability or incapacity
- Congenital abnormalities/birth defects (see section 3.8.3)
- Other important medical events that may jeopardize the participant or may require intervention to prevent one of the outcomes listed above
- All Grade 4 events (not limited to a laboratory abnormality) not already reportable in one of the above categories.

These events will be graded for severity according to the DAIDS Table for Grading the Severity of Adult and Pediatric Adverse Events, Version 1.0, December 2004 (also known as the DAIDS AE Grading Table; see Appendix H) and used to monitor the rate of adverse events in the two treatment groups during the study.

All abacavir hypersensitivity reactions (see section 4.1.8), regardless of grade, are also defined as serious events for the purposes of this protocol, and are reported in an expedited fashion as such.

For participants who are receiving study-supplied ART at the end of the study, these events must continue to be reported in an expedited manner while those participants are receiving ART supplied by the study. Procedures for reporting these events are described in the START *Protocol Instructions Manual*.

3.8.5 Serious Adverse Event Reporting Required by the European Union

In accordance with the EU Directive 2001/20/EC (http://europa.eu/eur-lex/pri/en/oj/dat/2001/l_121/l_12120010501en00340044.pdf) and member state requirements, the sponsor, through its legal representative in Europe, will ensure that all relevant information about serious adverse events is recorded and reported to the central and/or concerned member state authorities and IECs as appropriate and in compliance with requirements for expedited reporting. The sponsor will ensure that investigators recognize their responsibility for reporting of serious adverse reactions and set up a system to allow the pharmaceutical companies providing study drugs (Marketing Authorization Holders) to report serious adverse reactions according to their obligations. Data collected for reporting of the events in section 3.8.4 will be used to construct the required reports to European authorities.

4 Clinical Management Issues

The following clinical management guidelines apply to both the early and deferred groups.

4.1 Management of ART

4.1.1 Use of Potent Combination ART

All participants taking ART in START must be prescribed potent combination therapy, except for the reasons noted in section 3.7.1. A regimen listed on the table "Antiretroviral Components Required for the Initial Regimen in START" on the INSIGHT website must be prescribed for the initial ART regimen for participants in either arm (see Appendix H).

The University of Minnesota, the sponsor of START, has a financial interest via royalty income in abacavir, one of the treatment drugs available for use in this study.

4.1.2 Factors Affecting Choice of an ART Regimen

Clinicians should take into consideration the following factors when choosing an ART regimen:

- Gender-related issues
- Other co-morbidities, including co-infection with hepatitis B or C
- Adherence issues
- Use of alcohol and recreational drugs
- Social circumstances
- Co-administration of antiretroviral drugs with overlapping toxicities
- Co-administration of concomitant medications with the potential for drug-drug interactions with particular drugs/classes of drug that are used in combination ART
- Presence of resistance and cross-resistance

4.1.3 Antiretroviral Drug Product Information

The package insert or product information for each antiretroviral drug should be consulted for information on dosing, contraindications, and drug interactions. Links to product information and drug interactions are listed on the INSIGHT website (see Appendix H).

4.1.4 Approved Sources for Antiretroviral Drugs

Clinicians may only prescribe antiretroviral drugs for participants in START that have approval or tentative approval by the FDA or the European Medicines Agency (EMEA).

- The INSIGHT Central ART Repository will stock selected approved antiretroviral drugs donated by pharmaceutical companies. Further information about antiretroviral drug availability and distribution from the INSIGHT Central ART Repository can be found in the *START Protocol Instructions Manual* and on the INSIGHT website (see Appendix H).

- Antiretroviral drugs, including generic formulations, that meet the approval criteria above and are obtained from local sources may also be used in START.

Antiretroviral drugs used for the initial or subsequent ART regimens in START may be obtained from either the INSIGHT Central ART Repository or from local sources, as long as the drugs meet the approval criteria above.

4.1.5 Intolerance of an Antiretroviral Drug

In case of intolerance of one or more antiretroviral drugs in the initial regimen, the clinician is encouraged (but not mandated) to prescribe another preferred regimen from the table "Antiretroviral Components Required for the Initial Regimen in START" on the INSIGHT website (see Appendix H).

4.1.6 Drug Toxicity and Grading

All participants taking ART should be closely monitored for signs and symptoms of drug toxicity. For management of toxicities, clinicians should refer to product information available on the INSIGHT website (see Appendix H).

Symptoms and laboratory findings should be graded using the DAIDS AE Grading Table. This table is found in the *START Protocol Instructions Manual* and on the INSIGHT website (see Appendix H).

Changes in ART due to treatment toxicities may be made at any time.

4.1.7 Use of Antiretroviral Drugs in Women of Child-bearing Potential

For women of child-bearing potential, clinicians should adhere to recommendations in current product information of the antiretroviral drugs prescribed regarding counseling in the use of contraception and monitoring for pregnancy. For example, current product information for efavirenz, a Pregnancy Category D drug with risk for teratogenicity, states that "barrier contraception should always be used in combination with other methods of contraception (e.g., oral or other hormonal contraceptives)." Current product information is available on the INSIGHT website (see Appendix H).

4.1.8 Abacavir Hypersensitivity Reaction

Participants known to carry HLA-B*5701 must not receive abacavir. Participants for whom abacavir will be prescribed must be tested for the HLA-B*5701 allele; only those who are negative for the allele may be prescribed abacavir. In any participant treated with abacavir, the clinical diagnosis of suspected hypersensitivity reaction remains a clinical diagnostic decision. Even in the absence of the HLA-B*5701 allele, it is important to discontinue abacavir permanently and not re-challenge with abacavir if a hypersensitivity reaction cannot be ruled out on clinical grounds, due to the potential for a severe or even fatal reaction. Suspected cases of abacavir hypersensitivity of any severity (grade) must be reported in an expedited manner.

4.1.9 Resistance Testing

When initiating ART in either treatment group, resistance testing may be helpful to guide therapy changes; national or local treatment guidelines should be consulted.[37,38] Additional information is provided in Appendix F.

The decision to do resistance testing immediately prior to initiating ART should consider: (1) the regimen to be prescribed; (2) availability of previous resistance tests (e.g., closer to the time of seroconversion); and (3) risk of having transmitted drug resistance based on local data.

At each follow-up visit, the results of any locally obtained resistance testing will be recorded.

Plasma specimens will also be stored at baseline, 1 month, 4 months, and every 4 months thereafter for possible resistance testing. In addition, plasma specimens will also be collected from participants each time the ART regimen is switched or stopped due to an elevated HIV RNA level (or presence of resistance mutations). On a periodic basis, genotypic resistance testing may be performed on a sample of stored plasma specimens to assess prevalence of drug-related mutations.

Real-time resistance testing will not be provided as part of the START protocol.

4.1.10 Management of Virological Failure

If a participant experiences virological failure on any ART regimen, local guidelines should be followed to select antiretroviral drugs that will fully suppress HIV replication. Clinicians should consider the full spectrum of available and qualifying drugs.

In cases of multiple virological failures, participant adherence, the number of potential drug options available, and the risks and benefit of ART should be considered. For these participants, the use of drugs through expanded-access programs is permitted. There are no restrictions on use of ancillary therapies for HIV or use of other concomitant medications.

4.1.11 Discontinuation of ART

Treatment half-lives of the drugs taken should be considered any time ART is discontinued for any reason (e.g., for toxicity, intercurrent illness, elective surgery). In general, discontinuation of ART is not recommended, but it is recognized that there may be circumstances when it occurs. Differential half-lives of antiretroviral agents may have therapeutic implications for interruptions in therapy, particularly when participants are maximally suppressed. For instance, the prolonged half-life of the non-nucleoside reverse transcriptase inhibitors (NNRTIs) (efavirenz and nevirapine) could lead to unintentional monotherapy for a short period of time if all components of a potent NNRTI-containing regimen are discontinued at the same time. This may be even more significant in some intracellular reservoirs where the half-life of the NNRTIs may be even longer. The implications of these pharmacokinetic phenomena are unclear;

however, one small study that evaluated a 4-week interruption versus continuous therapy reported the emergence of resistance in five participants in the intermittent arm.[39] In SMART, among patients in the DC group taking an NNRTI and with an HIV RNA level ≤ 400 copies/mL at entry, those who interrupted all antiretroviral drugs simultaneously as compared to stopping ART drugs in a staggered manner or after replacing the NNRTI with another drug were less likely to achieve re-suppression when re-starting the NNRTI regimen. There was also a trend for more NNRTI mutations to develop when a simultaneous interruption strategy was used.[40]

4.1.12 Documentation of Reasons for Initiation or Change in ART

Whenever ART is first initiated, and every time ART is changed for participants in either group (including discontinuation and subsequent re-initiation), the relevant information must be reported on the appropriate case report form. The reason(s) for (re)initiation, change or discontinuation of ART, including treatment-limiting toxicities, will also be recorded on the case report form.

4.1.13 Use of Drugs for Prophylaxis for Opportunistic Diseases

Just as for ART, local clinical practice guidelines should be consulted regarding choice of agents for prophylaxis for opportunistic diseases, if and when such prophylaxis is indicated.

4.1.14 Provision of Antiretroviral Drugs at Study Completion

Once the active follow-up phase of START has been completed, ART will be obtained by prescription from the participant's clinician. It is anticipated that at the end of START a closeout period of approximately 6 months will be required to complete all data collection. During this time period, ART from the INSIGHT Central ART Repository may be used, and sites will be expected to transition all participants to other sources of ART. During this time period adverse events will continue to be reported. Stopping ART is not recommended. All participating sites must have a plan for providing ART to participants at the end of the study.

4.2 CD4+ Cell Count and Viral Load Monitoring

CD4+ cell counts should be performed by laboratories that participate in proficiency testing programs.

Participants may be monitored more closely than data collection for this protocol requires. The frequency of non-study visits and measurements of CD4+ cell count and viral load will depend on the participant's clinical status and recent CD4+ cell count and viral load status. CD4+ counts can sometimes decline rapidly. Thus, for participants in the deferred group, it is important to monitor their CD4+ counts closely and begin preparing them to begin ART once the CD4+ count declines to < 400 cells/mm^3, in particular in participants with higher viral loads. ART should be initiated as recommended in section 3.7.1.

Participants randomized to the deferred arm whose CD4+ cell count drops to less than 350 cells/mm^3 should have a repeat CD4+ measurement within 4 weeks to confirm this decrease. If the decrease is confirmed, the clinician should prescribe ART to be started by the participant as soon as possible within 4 weeks of confirmation of the decrease. In some cases, clinicians may choose to initiate ART before these criteria are met. Deviations from the protocol-specified criteria must be reported on a case report form.

Following the initiation of ART (immediately following randomization for the early ART arm or later in follow-up for the deferred arm), local guidelines should be followed for monitoring CD4+ cell count and HIV RNA levels. Most guidelines recommend that HIV RNA level be measured within 2-8 weeks after ART initiation. For participants on stable ART, HIV RNA levels should be determined every 3-4 months. For START, HIV RNA levels are required at least every 4 months.

4.3 Pregnancy and Breastfeeding

Women are not eligible for enrollment into the study during their pregnancy or while they are breastfeeding. However, they may be randomized after delivery if they are ART naive. World Health Organization (WHO) guidelines recommend that women with HIV infection avoid breastfeeding "when replacement feeding is acceptable, feasible, affordable, sustainable and safe." [41]

For women of child-bearing potential, a serum or urine pregnancy test must be performed before randomization and whenever pregnancy is suspected. For women to be prescribed efavirenz, a pregnancy test should be performed as close to the start of treatment as feasible and no more than 14 days beforehand.

Women in either treatment group who become pregnant during follow-up should be managed according to principles found in national[42,43] and international guidelines.[41, 33] This requires a careful assessment of the mother's HIV disease stage, antiretroviral experience, gestational age, and the risks and benefits of specific antiretroviral regimens or other interventions.

Pregnant women are still considered active study participants and will be counted in their assigned randomized group for primary analyses. After delivery and breastfeeding, women will continue their assigned treatment strategy. Women will be counseled concerning breastfeeding based on national and/or WHO guidelines. Outcomes of pregnancy will be reported.

Study staff are encouraged to register pregnancies that occur on study prospectively with *The Antiretroviral Pregnancy Registry*. A link to this registry is available on the INSIGHT website (see Appendix H.)

4.4 HIV Transmission Counseling

All participants should receive counseling regarding prevention of HIV transmission to others as indicated.

4.5 Study Withdrawal

Participants may withdraw from the study at any time at their request and resume participation at any time upon re-consent. A participant may be withdrawn if:

- He/she relocates and data can no longer be collected.
- He/she is imprisoned, or involuntarily incarcerated for medical reasons. In this case, no data will be collected during the imprisonment or involuntary incarceration. However, once released from imprisonment or involuntary incarceration, an individual may resume participation in the group to which he or she was originally assigned upon re-consent.
- The study is discontinued.

All participants should otherwise be followed according to protocol. Even if a participant chooses not to adhere to the treatment assignment or data collection schedule, every effort should be made to follow participants for the primary composite endpoint until the end of the study.

4.6 Co-enrollment in Other Studies

There are no restrictions on co-enrollment in other studies as long as co-enrollment does not interfere with a participant's assigned treatment strategy in START.

5 Evaluation

5.1 Data Analysis

The primary analysis will be by intention to treat, comparing the early and deferred groups. Thus, all participants will be included in the primary treatment comparison, irrespective of whether they adhered to their assigned ART strategy. Time-to-event methods, including stratified log-rank tests, proportional hazards regression analysis, and Kaplan-Meier cumulative event curves, will be used to summarize the primary endpoint (i.e., time to the first event) and major secondary outcomes.[44] As part of these analyses, the proportionality of the hazards over time will be assessed. The primary analysis will be based on a Cox model with a single indicator for treatment group and with strata corresponding to geographic region (North America, South America, Europe, Australasia, and Africa).

Subgroup analyses for the primary endpoint and major secondary outcomes will be performed to determine whether the treatment effect (early versus deferred) differs qualitatively across various baseline-defined subgroups. Subgroup analyses will be performed by age, gender, race/ethnicity, geographic region, the presence of risk factors for serious non-AIDS conditions, baseline CD4+ cell count, baseline HIV RNA level, calendar date of enrollment in order to assess the effect of different treatment patterns that may emerge, and the ART regimen prespecified at the time of randomization. The latter subgroup analysis will permit evaluation of major outcomes for those who receive an NNRTI-based treatment as immediate ART versus deferral of ART (compared to those who would have been given an NNRTI-based regimen immediately had they been randomly assigned to immediate ART), and for those who receive a protease inhibitor (PI)-based regimen as immediate treatment versus deferral. The consistency of the treatment difference across participating countries will also be assessed. An overall test of heterogeneity will provide evidence of whether the magnitude of the treatment difference varies across baseline subgroups.

The goal of the study is to enroll participants at variable risk of serious non-AIDS diseases. Thus, the characteristics of participants enrolled will be closely monitored to ensure good representation of participants with major risk factors for serious non-AIDS conditions. Descriptive analyses will also be carried out summarizing ART regimens used by the early and deferred groups. These analyses will be carried out by geographic region and by calendar period of enrollment and follow-up. The initial ART regimen used will be compared to the regimen specified prior to randomization.

The CD4+ inclusion criteria for the study and the CD4+ count threshold for initiation of ART in the deferred strategy were defined to result in two treatment groups that differ substantially in terms of ART exposure, and consequently, HIV RNA level and CD4+ cell count. As part of the ongoing monitoring of this study, time to initiation of ART in the deferred group will be monitored and HIV RNA levels and CD4+ cell counts will be compared for the early and deferred groups. Initiations of ART in the deferred group that are not in keeping with the protocol will be closely monitored. Likewise,

discontinuation of ART in the early and deferred groups will be closely monitored. Kaplan-Meier life-table methods will be used to estimate the cumulative percent of participants in the deferred group who initiate therapy after different periods of follow-up. Follow-up levels of HIV RNA and CD4+ count will be summarized in a number of ways. Follow-up time spent in various categories will be compared. Longitudinal measurements of viral load and CD4+ cell count will be summarized using measured levels (or log transformed) and using repeated binary assessments (e.g., viral load below 50 copies/mL).

In addition, within each treatment group a number of analyses aimed at predicting changes in CD4+ cell count, viral load, and clinical responses will be carried out. These will include analyses of predictors of HIV RNA suppression and CD4+ cell count increases following initiation of ART.

Analyses aimed at understanding whether differences in CD4+ cell count and HIV RNA levels explain differences in the primary endpoint and its components will be carried out. These models will employ Poisson regression models and Cox models with time-varying covariates. Account will be taken of the measurement error associated with CD4+ count and HIV RNA that could attenuate associations with risk of the primary endpoint.

5.2 Data Monitoring

The trial will be conducted under the direction of the START study protocol team. Members of the protocol team are listed in Appendix D. The protocol team (except those who prepare the confidential analyses) and all participating investigators will be blinded to interim results on clinical outcomes.

An independent DSMB, supported by NIAID, will meet as often as required, but at least annually, to review the general conduct of the trial and to review interim analyses of the major clinical outcomes. The monitoring plan for the review of the primary endpoint and its two major components is described below.
A sample size re-estimation will be carried out by the protocol team before the target enrollment of 4,000 participants is achieved to ensure that the planned sample size is adequate. For the sample size re-estimation, the protocol team will use only pooled endpoint data, i.e., the number and rate of AIDS* and non-AIDS events for both treatment groups combined. The protocol team will use these pooled event data and other relevant data sources to estimate the rates of AIDS*, non-AIDS, and deaths due to other causes (including unknown causes).

The DSMB will be asked to recommend early termination or modification only when there is clear and substantial evidence of a treatment difference. As a guideline, the Lan-DeMets spending function analog of the O'Brien-Fleming boundaries will be used to monitor the primary endpoint comparison.[45,46] The DSMB will be asked not to stop the study early unless there is evidence of a significant treatment difference based on the spending function boundary for the primary endpoint and each of the two major components of the primary endpoint – AIDS*, and non-AIDS or deaths not attributed

to AIDS – are consistent (in the same direction, for example, Z > 1.5 for each outcome).

The DSMB will also review other relevant data that might impact the design of START, e.g., data from other completed trials, and cohorts with similarly defined target populations.

At each DSMB review, beginning with the review prior to the end of enrollment when sample size is re-estimated, futility analyses will be presented to the DSMB by the unblinded statisticians based on conditional and unconditional power. *Conditional* power incorporates the observed results by treatment group thus far (and uses the originally assumed treatment effect for future data) to calculate the conditional probability of obtaining a significant result by the end of the trial. In contrast, *unconditional* power does not take into consideration the observed treatment difference. It uses a revised estimate of the event rate in the deferred ART arm based on the observed data, the planned duration of follow-up, and the originally assumed treatment effect to calculate what the real power was at the beginning of the trial. Participants will be followed to a common closing date a minimum of 3 years after the last participant is enrolled. Thus, participant follow-up will range from 3 to 6 years, with an average follow-up of approximately 4.5 years.

Conditional and unconditional power estimates are used for two different purposes. Conditional power tells us whether we are likely to get a significant result, whereas unconditional power tells whether a null result would still be meaningful. For example, suppose the unconditional power were only 40%. Even if the true treatment benefit were as originally hypothesized, there would be a 60% chance of missing it. Therefore, a null result would not rule out the originally hypothesized treatment benefit. On the other hand, if unconditional power were high — say 90% — then a null result would effectively rule out the originally hypothesized treatment benefit.

As a guideline, we recommend that the DSMB first consider unconditional power. If unconditional power is less than 70%, the DSMB should then consider conditional power. If conditional power, given the observed data and assuming the originally hypothesized treatment effect thereafter, is less than 20%, consideration should be given to stopping the trial. We recommend early termination for futility only if both conditional and unconditional power estimates are low, i.e., only if a null result is both likely and not meaningful. It is possible that unconditional power is low in the presence of a very large treatment effect of early ART. Hence, there is a need to also consider conditional power. Such a scenario would not be grounds for stopping for futility because conditional power would probably still be high, indicating that a null result is unlikely.

In summary, the DSMB for START will be provided with the aforementioned guidelines but be expected to use their expert and independent judgment concerning early termination. It is recognized that there are a number of considerations in determining whether a trial should be stopped early. For that reason, we propose guidelines to the

DSMB, not rules. This line of thinking was summed up well by Canner in his reflections on the Coronary Drug Project: "…no single statistical decision rule or procedure can take the place of the well-reasoned consideration of all aspects of the data by a group of concerned, competent, and experienced persons with a wide range of scientific backgrounds and points of view".[47]

6 Protection of Human Subjects & Other Ethical Considerations

6.1 Local Review of Protocol and Informed Consent

Prior to the initiation of the study at each clinical research site, the protocol, all informed consent forms and the participant Information materials will be submitted to and approved by the site's IRB or IEC. Likewise, any future amendments to the study protocol will be submitted and approved by each site's IRB or IEC. After IRB/IEC approval, sites must register for START before screening potential participants, and must register for any protocol amendments. Protocol registration procedures are described in the START *Protocol Instructions Manual*.

6.2 Ethical Conduct of the Study

The study will be conducted according to the Declaration of Helsinki in its current version (2004); the requirements of Good Clinical Practice (GCP) as defined in Guidelines, EU Clinical Trials Directive (2001/20/EC), and EU GCP Directive (2005/28/EC); Human Subject Protection and Data Protection Acts; the US Office for Human Research Protections (OHRP); or with the local law and regulation, whichever affords greater protection of human subjects.

6.3 Informed Consent of Study Participants

All study participants must sign all applicable approved informed consent forms (see samples in Appendix A) prior to any study-related procedures.

6.4 Confidentiality of Study Participants

The confidentiality of all study participants will be protected in accordance with GCP Guidelines and national regulations.

7 Other Important Documents and Policies

7.1 Reference Documents

Study procedures are described in detail in the *START Protocol Instructions Manual.*

7.2 Data Collection and Monitoring

Study data will be collected on standardized case report forms. It is assumed that most data will be collected during participant visits to health-care providers. In some instances, it may be necessary to obtain and abstract hospital records. Written permission from the participant is generally required. Study data and case report forms will be made available to site monitoring personnel. Monitoring may be performed by staff from the INSIGHT International Coordinating Centers or Site Coordinating Centers or by contractors of the primary funder.

At a minimum, all items referenced in the protocol as being relevant to the research study will be recorded in the participant's research record in accordance with standard procedures. In addition, all items specifically required by the protocol will be recorded on case report forms. Items that are recommended but not required by the protocol may or may not be recorded on case report forms.

7.3 Publications and Presentations

Publications and presentations related to data obtained from this study will adhere to the INSIGHT Publications and Presentations Policy on the INSIGHT website (see Appendix H).

8 Substudies

This section describes three substudies. Not all sites will be participating in these substudies. At the sites conducting the Genomics and/or Neurology substudies, participant participation in either substudy is optional and requires a separate consent. At sites conducting the Informed Consent substudy, all participants are part of the substudy by virtue of going through the informed consent process at that site. Participants are informed that, by signing the consent form for START, they are also participating in this substudy.

8.1 Genomics Substudy

8.1.1 Background and Rationale

Despite progress in the treatment of HIV/AIDS and greater understanding of the pathophysiology of HIV infection, clinicians and researchers continue to observe unexplained differences among individuals in both the progression of untreated HIV infection and response to ART. Some of these differences can be attributed to individual participant behavior (e.g., adherence to therapy) and/or diversity of the infecting virus (e.g., viral replicative capacity), but a significant portion is likely related to individual (host) differences that are determined by individual genetics.

One example of this is the 32 base-pair deletion in the CCR5 gene that in a homozygous state confers protection against HIV infection.[48,49,50] Another example is the association of human leukocyte antigen, HLA B*5701, with abacavir hypersensitivity reaction.[51] While these findings require further understanding before routine clinical application, the first example shows how knowledge of host genetics may lead to a targeted treatment strategy based on a subject's individual risk of disease progression, and the second example shows how knowledge of genetics can lead to targeted therapy to maximize efficacy and minimize toxicity.

These examples illustrate the promise of genetic knowledge in the management of HIV/AIDS, but the next step is to refine current genetic associations while continuing to discover new associations. In order to utilize current and future advances in genetic technology to better understand the role of host genetics in the pathophysiology and treatment of HIV infection, there is a need to link genetic data with clinical outcomes data in a large, well-characterized, HIV-infected population.

8.1.2 Purpose

The purpose of this study is to obtain a whole blood sample from which DNA will be extracted to study validated (present and future) genetic variants that determine the risk of the various primary and secondary outcomes assessed in START. Known genetic variants include for example HLA/killer cell immunoglobulin-like receptors (KIRs), genomic factors controlling viral replication, and genetic associates of dyslipidemia. Also, the material may be used to assess validated (present and future) genetic variants associated with tolerability of all the various medical interventions used in the study. Finally, the material will be used, via genomewide analysis, for appropriate control for population structures in this trial. These analyses may be done in participants included in START specifically or may be merged with similar studies from other sources.

8.1.3 Study Design

This nonrandomized multicenter protocol is designed to obtain a whole blood sample from participants who are planning to participate in START. The specimen will be archived for currently unspecified human genetic and other related analyses.

8.1.4 Sample Size and Statistical Considerations

All of the participants in START may participate in this substudy, so the potential sample size is the same as that for the main protocol.

8.1.5 Participant Selection

Enrollment is open to all participants in START. Consent to participation in START is required to provide a source of follow-up data to use in conjunction with the human genetic or other related assays in investigating questions of interest.

Inclusion Criteria

- Consent to randomization in START.
- Signed informed consent for the substudy (see Appendix A4).

Exclusion Criteria

None.

8.1.6 Enrollment and Data Collection

Following consent to the START study, a participant will be asked to sign the START Genomics substudy informed consent. It is strongly encouraged that participants consent to the Genomics substudy and provide a blood sample prior to randomization in START. It is, however, acceptable for participants to enroll in the Genomics substudy any time after consent and randomization in the main study.

Participants who consent to the START Genomics substudy will have the following collected:

- Nine (9) mL of whole blood, frozen and shipped as directed in the *START Genomics Substudy Protocol Instructions Manual.*

Participation in the START Genomics substudy is considered complete when the START Genomics substudy enrollment form is received at the INSIGHT Statistical and Data Management Center (SDMC). This form will collect the official date of enrollment, which is the date the blood is drawn.

8.1.7 Follow-up

There are no follow-up requirements for this substudy.

8.1.8 Specimen Collection

Sites will ship frozen whole blood specimens to a central specimen repository designated in the *START Genomics Substudy Protocol Instructions Manual* for long-term storage. At a future point, DNA will be extracted from either a subset of specimens or all specimens. Additional future plans might include separation of plasma on samples for either a subset of participants or all participants.

8.1.9 Human Subjects

This protocol must receive the approval of the participating site's IRB or IEC prior to implementation. All participants must sign an informed consent form (see sample in Appendix A4).

Participants who wish to withdraw consent for the storage and use of specimens collected in this protocol may do so at any time by contacting a member of the research staff and expressing their intention to withdraw consent in writing. If a participant withdraws consent, the specimen and any products derived from the specimen will not be used in further analysis and every effort will be made to destroy them. However, results of laboratory tests that have been performed prior to the withdrawal of consent will continue to be available for future analyses and will not be destroyed. A participant who has withdrawn consent and wishes to reconsider may reenroll in the protocol by repeating the informed consent and enrollment process.

In some situations, it may be important for a participant who has already provided one blood sample under this protocol to provide an additional sample (for example, in the event that specimen transport problems or labeling errors render the original specimen unacceptable). If a new specimen must be drawn, participants must repeat the informed consent and enrollment process.

8.1.10 Confidentiality

The confidentiality of all study participants will be protected in accordance with standard IRB/IEC policies and procedures in addition to those procedures specified in this protocol document.

The privacy of individual participants will be protected by the data management system described in the *START Genomics Substudy Protocol Instructions Manual.* After approval of a concept or study requiring use of genetic material, whole blood collected under this protocol will be processed to yield host DNA or other products, hereafter referred to as human biological material (HBM). Processed HBM will be labeled with a generated identification number (GID) assigned by the INSIGHT SDMC. This GID will be used in place of the START participant identification number (PID) in all data transactions concerning individual results of tests on the processed HBM. Thus, results of genetic tests on individual participants cannot be connected to a particular participant without access to both the participant's GID and the participant's PID. Data linking the PID to the GID will be stored only at the SDMC in an encrypted file. Data security will be maintained by minimizing the number of individuals at the SDMC with access to both identifiers.

Results of laboratory tests performed on HBM obtained in connection with this protocol will be stored in databases at the SDMC, indexed by the GID. These databases will not contain the participant PID or any other clinical data. The table for linking PIDs with GIDs will be accessible to a minimal number of individuals at the SDMC.

Further details of the data management practices intended to safeguard individual participant genetic information are given in the *START Genomics Substudy Protocol Instructions Manual.*

The following paragraph concerning the Certificate of Confidentiality applies only to sites located within the United States:

In addition, a Certificate of Confidentiality has been obtained for START to help protect the privacy of participants by withholding their names and other identifying characteristics, including processed DNA and results of genetic testing, from all persons not connected with the conduct of research.

8.1.11 Access to Stored Specimens Collected for the START Genomics Substudy

Stored specimens, including HBM processed from the donated whole blood specimens, will not be sold to third parties.

Tests using stored samples that are consistent with the purpose of this substudy may be proposed by any START investigator who follows established INSIGHT policies and procedures. Before testing is initiated, the proposal must be approved by the INSIGHT Executive Committee and the IRB governing the SDMC.

Whether in the form of whole blood or blood products (e.g., extracted host DNA), the stored HBM will be used for studies relevant to the health of persons with HIV infection, including its epidemiology, diagnosis, pathogenesis, complications, treatment and prognosis. As previously stated, tests will include validated (present and future) genetic variants that determine the risk of the various primary and secondary outcomes assessed in START. Known genetic variants include, for example, HLA/KIR, genomic factors controlling viral replication, and genetic associates of dyslipidemia. Also, the material may be used to assess validated (present and future) genetic variants associated with tolerability of the various medical interventions used in the study. Finally, the material will be used, via genomewide analysis, for appropriate control for population structures in this trial. These analyses may be done in participants included in START specifically or may be merged with similar studies from other sources.

8.1.12 Access to Individual Test Results

Due to the experimental nature of the assays involved and the inherent difficulty of interpreting their clinical significance in individual cases, individual test results will not be provided to participants, investigators, clinical site research staff or health-care providers. Summaries of clinically relevant findings from genetic tests will be disseminated to all study participants through individual clinical units and the INSIGHT website. In extraordinary circumstances when knowledge generated from genetic tests on HBM may have profound and unequivocal implications for the health of study participants, every reasonable effort will be made to offer study participants the genetic test repeated outside of the study. However, original study results cannot be provided to participants or clinicians under any circumstances.

Data generated from HBM for START will not be made available to employers, insurance companies, or other third parties that are not directly involved in the clinical research.

8.2 Neurology Substudy

8.2.1 Background and Rationale

The impact of HIV infection upon neurocognitive function in participants who have early HIV disease with high CD4+ cell counts is notable for the paucity of studies that directly address this issue, and more particularly the issue of whether ART during this period can prevent or reverse the development or progression of neurocognitive impairment during this phase of HIV infection. However, the conflation of data from studies of primary HIV infection (PHI) and studies of neurocognitive performance, cerebrospinal fluid (CSF) and histopathological findings in patients with early HIV disease indirectly provide important evidence that HIV infection involves the brain early in the course of the disease, establishes a state of chronic central nervous system (CNS) immunoactivation, and may be detrimental to brain integrity. The following discussion is largely confined to those studies that include HIV-infected patients with early disease and CD4+ cell counts of 350 cells/mm^3 or greater.

PHI may be associated with a number of different neurological presentations including meningoencephalitis, meningitis, seizures and cranial neuropathies.[52,53,54] During PHI, HIV is detected in the CSF [55] and the CSF HIV viral load is highest in those patients with neurological symptoms.[55] An important marker that suggests that early damage occurs within the CNS during PHI is neurofilament protein (NFL), which is elevated in some patients during PHI.[56] Similarly, CSF levels of quinolinic acid, an endogenous neurotoxin, are elevated in asymptomatic disease.[57]

Following PHI, HIV remains detectable in the CSF throughout the course of the disease in most patients[58] including those with CD4+ cells > 500 cells/mm.[54,59,60] The source of ongoing HIV replication within the CNS may result from infected CD4+ T cells trafficking through the brain and/or from productive infection of perivascular and meningeal macrophages, given the respective appellations “transitory” and “autonomous” CNS infection by Spudich et al. The distinction between the two proposed mechanisms is important as transitory CNS infection appears to occur in early disease with associated rapid CSF decay kinetics following treatment with ART, similar to those seen in plasma. Patients with more advanced HIV disease are likelier to have more autonomous CNS infection with slower associated decay kinetics following ART.[60,61]

There is evidence to show that the CNS adaptive immune response is integral to the control of HIV infection in the brain following PHI: data support that important contributions are made by both humoral[62]and cellular immunity.[63,64,65,66] McCrossan et al recently proposed that control is conferred largely by cellular immunity principally involving CD8+ cells. In their histopathological study of patients with early HIV disease (including patients with CD4+ cell counts as high as 824 cells/mm^3) they reported that there was a significant correlation between the proportion of CD8+ cells and the HIV proviral load in brain tissues suggesting that the level of immune surveillance increases as the level of HIV proviral DNA increases. McCrossan further hypothesized that with

progressive *peripheral* immunosuppression the integrity of the CNS surveillance afforded by cellular immunity wanes, and hence the brain is vulnerable to upregulated local CNS infection and increasing numbers of infected monocyte/macrophages trafficking into the CNS.[66] This hypothesis is supported by a number of studies wherein patients do achieve improved neurocognitive function despite receiving highly active antiretroviral therapy (HAART) regimens that do not penetrate the CSF well.[67,68,69,70,71,72] A commonality among some of these studies is that following commencement of ART, neurocognitive performance improved in association with significant decreases in plasma HIV viral load[68,71] or a significant decrease in both HIV viral load and an increase in CD4+ cell counts.[69]

Predictors of clinical neurocognitive disease progression in early HIV disease have been described in one longitudinal study from the pre-HAART era of 76 patients who seroconverted a median of 11 months (range, 1 month to 4 years) prior to study entry.[73] Within 5 years of estimated seroconversion, approximately 10% of those patients whose study baseline CD4+ cell counts were > 400 cells/mm^3 had developed HIV-related neurocognitive impairment (NCI). Conversely, 40% of those patients whose CD4+ cell counts were < 400 CD4 cells/mm^3 counts at study baseline were neurocognitively impaired over the same time period. Plasma viral loads greater than 30,000 copies/mL also independently predicted more rapid neurocognitive decline. The few patients treated with two drugs were significantly less likely to become impaired.[73]

Accurate estimates of the prevalence of impairment of neurocognitive function in otherwise healthy untreated patients with ≥ 500 CD4+ cell counts/ mm^3 are not available. Notably, however, studies of patients who were stratified according to HIV disease stage, rather than by CD4+ cell count, show that asymptomatic patients *per se* do experience mild neurocognitive deficits, [74,75,76] compared to HIV negative controls. Nonetheless, even mild neurocognitive impairment may be associated with diminished functioning in day-to-day life and an increased risk of unemployment.[77]

In summary, these findings demonstrate that the CNS is involved very early in the course of HIV infection. Control of CNS infection appears largely predicated upon intact peripheral immunity, and the decline of peripheral immunity would be delayed by early ART. Patients with early disease and high CD4+ cell counts may have mild neurocognitive impairment that, in turn, may affect aspects of their everyday life including vocational capacity. Predictors of neurocognitive decline in early HIV disease are a high plasma viral load and CD4+ cell counts < 400 cells/mm^3. The anticipated fall in HIV viral load and concomitant rise in CD4+ cell counts afforded by early ART would theoretically improve neurocognitive performance in this early disease patient group or, at the least, prevent neurocognitive decline.

Purpose

The purpose of the study is to determine whether immediate initiation of ART in ART-naïve persons with a CD4+ count > 500 cells/mm^3 is superior with respect to neurocognitive function to deferring ART initiation until CD4+ counts decline to below 350 cells/mm^3.

Study Hypothesis
We hypothesize that early ART results in improved neurocognitive function compared to deferred ART.

8.2.2 Methodology

8.2.2.1 Study Design

This is a substudy of START. It is planned to co-enroll a total of 600 participants over 3 years at selected, geographically diverse sites. Randomization (1:1 ratio) to the early or deferred treatment groups will be determined by the START study. Participants will be followed to the common closing date of START. This is estimated to be 6 years after the beginning of enrollment (3 years of enrollment and a minimum of 3 years follow-up for each participant). Before enrollment ends, sample size of the main START study will be re-assessed and sample size recalculated. At the same time, sample size for the Neurology substudy will be re-calculated.

START Neurology Substudy Schematic

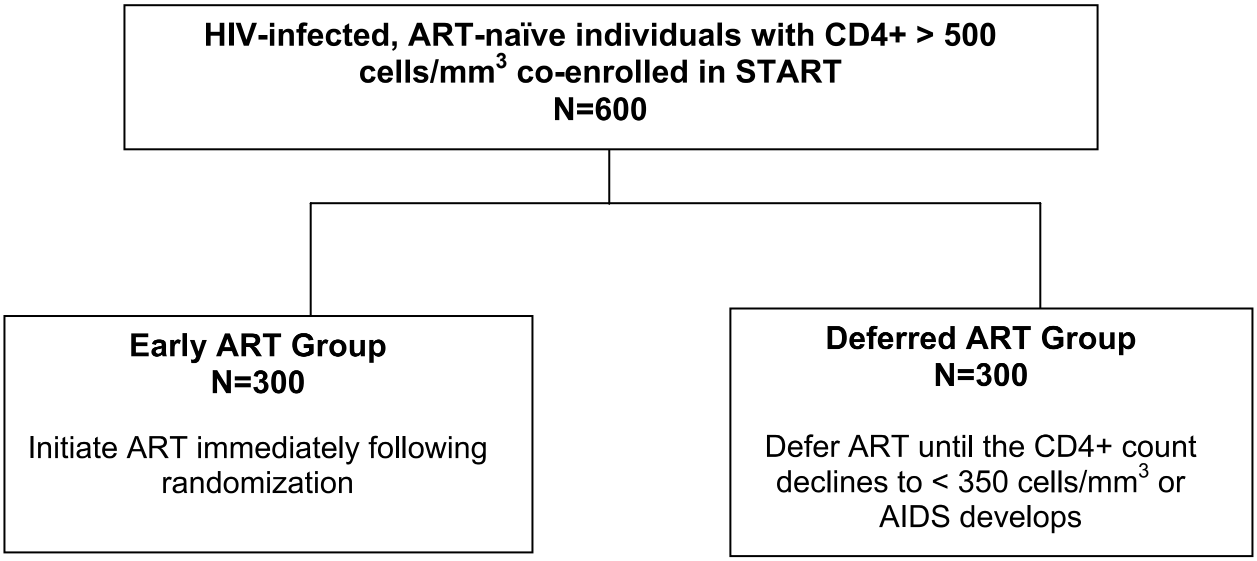

Data collection: Baseline, months 4, 8, 12, and annually thereafter.

8.2.2.2 Data Collection

To be collected at baseline (within 60 days prior to randomization), months 4, 8, and 12, and annually thereafter:

a. The quantitative neurocognitive performance (QNPZ-8) score, derived from a test battery consisting of
   - Grooved Pegboard test
   - Color Trails 1 test

- Color Trails 2 test
- WAIS-III Digit Symbol test
- Finger Tapping test
- Hopkins Verbal Learning test – revised (HVLT-R), Learning and Delayed Recall
- Semantic Verbal Fluency test (category fluency)

b. Center for Epidemiologic Studies Depression Scale (CES-D), a tool to screen for depression.

Additionally, to be collected at baseline:

- Extended demographics, including years of education, occupation, employment status, income, and area of residence (urban or rural)

8.2.2.3 Study Objectives

Primary Objective

To compare the early group with the deferred group for changes in neurocognitive function, as measured by the QNPZ-8 score.

Major Secondary Objectives

a. To compare the early and deferred ART groups for:
   - Changes in QNPZ-8 scores through the first year
   - Changes in neurocognitive function, as measured by each of the neuropsychological tests in the QNPZ-8 test battery, through the first year and overall
   - Proportions of participants with neurocognitive impairment
   - Changes in neurocognitive deficits as measured by the average deficit score (ADS)
   - Proportions of participants with depression as measured by the CES-D, and for changes in CES-D scores

b. To compare the early and deferred ART groups for changes in neurocognitive function in subgroups of participants defined by demographics (age, gender, education), geographic location, CD4+ cell count, HIV RNA level, estimated level of CNS penetration of the planned baseline ART regimen, drug classes in the planned baseline ART regimen, level of neurocognitive function at baseline, and other baseline characteristics, and to assess homogeneity of the treatment difference with respect to these baseline characteristics.

c. To determine the association of changes in neurocognitive function with baseline characteristics, including demographics (age, gender, education, occupation, employment status, income, urban or rural area of residence), geographic location, CD4+ cell count, HIV RNA level, perceived general health, estimated level of CNS penetration of the planned baseline ART regimen, drug classes in the planned

baseline ART regimen, level of neurocognitive function at baseline, dominant versus non-dominant hand, and others.

d. To determine whether changes in neurocognitive function are associated with CD4+ and HIV RNA levels through follow-up, perceived general health, duration of exposure to certain drug classes, estimated level of CNS penetration of the ART regimen, and other time-varying factors.

e. To study the association of factors measured at baseline with neurocognitive function at baseline. These factors include demographics, geographic location, CD4+ cell count, HIV RNA level, health history, perceived general health, and others.

f. To describe associations between the neuropsychological tests at baseline, and associations between the tests when measuring change in neurocognitive functioning through follow-up.

8.2.2.4 Study Endpoints and Outcome Measures

Neuropsychological Test Battery

The neuropsychological quantitative performance test battery to be used is comprised of eight neuropsychological tests that assess neurocognitive functioning across a range of cognitive abilities, shown in Table 1. Tests are described in more detail below.

Table 1. Neuropsychological Tests

Domain	Test
Attention/speed of information processing	Color Trails 1
Executive function	Color Trails 2
Verbal learning	HVLT-R, Learning
Verbal memory	HVLT-R, Delayed Recall
Language fluency	Semantic Verbal Fluency
Fine motor skills/ complex perceptual	Grooved Pegboard
Motor speed	Finger Tapping
Speed of information processing	WAIS-III Digit Symbol

Grooved Pegboard Test (Both Hands)[78]

The purpose of the test is to assess psychomotor speed and coordination. The Grooved Pegboard is a manipulative dexterity test that consists of 25 holes with randomly positioned slots in a 5 x 5 matrix. Pegs, which have a key along one side, must be rotated to match the hole before they can be inserted. This test requires more complex visual-motor coordination than most pegboards.
Score: Time to completion in seconds.

Color Trails Test: Color Trails 1 and Color Trails 2[79]
These are tests of speed for attention, sequencing, mental flexibility, and visual search and motor function. The tests are paper and pencil based. Color Trails 1 requires the participant to rapidly connect encircled numbers scattered on a page in sequence from 1 to 25. In Color Trails 2, each number is presented twice, once with a pink background and once with a yellow background, and on this part the participant rapidly connects encircled numbers in sequence, but alternates between pink and yellow colors.
Score: Time to completion in seconds, for each part.

WAIS-III Digit Symbol Test[80]
This is a test of psychomotor speed, concentration, and graphomotor abilities that requires the respondent to match symbols to numbers as quickly as possible, using a visual reference.
Score: Number correct in 120 seconds.

Hopkins Verbal Learning Test – Revised (HVLT-R): Learning and Delayed Recall [81]
The HVLT-R provides information on the ability to learn and immediately recall verbal information across trials, as well as the ability to recall this information after a delay. In the learning phase, a list of 12 words (four words from each of three semantic categories) is presented to the participant over three trials. In each trial, the same list is presented, and the participant is asked to recall as many items as possible from the list in any desired order. A 20-minute delay follows the administration of the three trials, after which the subject is asked to recall the list (delayed recall phase). In order to minimize practice effects that may result from repeated administrations, six alternate forms of the test are available.
Score, Total Learning: Number correct across the three learning trials (sum).
Score, Delayed Recall: Number of words correctly recalled after the 20 minute delay.

Finger Tapping Test (Both Hands)[82]
This is a test of motor speed. Using a specially adapted tapper, the participant is instructed to tap as rapidly as possible using the index finger. The number of taps is counted in five consecutive 10-second trials.
Score: Average number of taps across five trials excluding highest and lowest.

Semantic Verbal Fluency Test[83]
This test assesses speed of processing, memory, initiation, and inhibition, without requiring motor skills. It is a timed task requiring the production of words in a specific category in the subject's native language. Subjects are given 60 seconds to produce as many words as possible within a specific category (e.g. Animals).
Primary score: Number of correct words within 60 seconds.

Composite Outcome Measures

QNPZ-8 Score
The QNPZ-8 test battery consists of the eight neuropsychological tests described above. First, test scores will be standardized to *z-scores*, by subtracting the mean and

dividing by the standard deviation of test scores in reference populations. Best available normative data will be used, matched for each participant by age, education, sex, and race/ethnicity, when appropriate, and as available for each test. For the Finger Tapping and Grooved Pegboard tests, the average of the z-scores for the dominant and non-dominant hands will be used in calculating the QNPZ-8 score. For the HVLT-R, the z-scores for Learning and for Delayed Recall will be entered separately when calculating the QNPZ-8 score. The participant's eight individual z-scores will then be averaged to produce a quantitative neurocognitive performance z-score (QNPZ-8 score).

QNPZ-8 scores above zero denote above-average neurocognitive function and scores below zero denote below-average neurocognitive function compared to the reference population.

Neurocognitive Impairment

For the purpose of this study, neurocognitive impairment is defined as:

a. decrease in the QNPZ-8 score from the participant's baseline by at least 0.5,
   or
b. decrease in z-scores in two or more independent tests to at least 1 below baseline. The two tests can not be both Grooved Pegboard and Finger Tapping, since these tests both measure fine motor skills.

ADS

For each of the eight neuropsychological tests, the deficit score is calculated as follows:

Table 2. Calculation of Deficit Scores Based on Z-Scores

**Deficit Score**	**Z-Score**
0 (normal)	$\geq$ -1
1 (mild)	-1.5 to less than -1
2 (mild-moderate)	-2 to less than -1.5
3 (moderate)	-2.5 to less than -2
4 (moderate-severe)	-3 to less than -2.5
5 (severe)	< -3

The ADS is the average of the deficit scores for the eight neuropsychological tests. Higher scores denote more impairment. Assuming that the reference populations standardize the z-scores correctly to mean 0 and standard deviation 1, then participants who score more than 1 standard deviation below the population average on two of the eight tests would have an ADS of at least 2/8. In calculating the ADS, low performance on some tests is not cancelled out by high performance on other tests, as could be the case with the QNPZ-8.

CES-D

The CES-D[84] is a screening tool for depression. It is a 20-item pen and paper questionnaire designed to assess the frequency and severity with which symptoms of

depression are experienced. Questions refer to the past week. Participants rate each item on a 4-point scale. The CES-D score is obtained by summing the scores for the 20 items (scores for items 4, 8, 12, and 16 are reversed). CES-D scores range from 0 to 60. Scores of 16 to 26 are considered indicative of mild depression and scores of 27 or more indicative of major depression. The CES-D has been widely used in research and has been translated into a number of different languages.

CNS Penetration

The level of CNS penetration for a given ART regimen will be calculated as the CNS penetration effectiveness score (CPE) by Letendre et al.[85] In this algorithm, CNS penetration scores are assigned to each antiretroviral drug and the CPE score for a regimen is calculated as the sum of scores for the individual drugs. If newer algorithms are published, the best available will be used.

General Health

The participants' perception of their general health will be assessed by two items on a questionnaire, on a 5-point Likert scale and on a visual analog scale. These data are part of the QOL assessments in the main START study, and will be collected at baseline, month 4, and annually.

Primary Endpoint

Change in QNPZ-8 score

Secondary Endpoints

- Change in test scores for each of the eight neuropsychological tests – Grooved Pegboard, Color Trails 1 and 2, Finger Tapping, HVLT-R Learning, HVLT-R Delayed Recall, Semantic Verbal Fluency, and WAIS-III Digit Symbol
- Change in ADS
- Proportion of participants with neurocognitive impairment

8.2.2.5 Sample Size Calculations

A total sample size of 600 is considered sufficient to detect an average treatment difference of 0.13 in mean change in the QNPZ-8 scores from baseline, where the treatment difference is averaged over the 4, 8, and 12 month visits in the first year and annual visits thereafter, with a power of 80% at a 5% significance level. The difference of 0.13 in mean change in the QNPZ-8 scores would not be clinically meaningful for an individual participant but would be clinically meaningful for a participant group. Table 3 displays the sample sizes necessary under several scenarios. The sample size of 600 assumes a standard deviation of 0.7 for change in QNPZ-8 scores from baseline to any follow-up visit and is inflated by 10% to account for missed visits and loss to follow-up. With this sample size, a treatment difference of 0.1 would be detected with 58% power.

The sample size calculations were performed for a balanced design, assuming that the QNPZ-8 scores follow a longitudinal mixed model with exchangeable within-participant correlation structure and normally distributed random effects. The correlation structure implies that the between-participant standard deviations of change in QNPZ-8 scores from baseline to a given follow-up visit are equal through follow-up within each treatment group. For simplicity, the SD in Table 3 is expressed as standard deviation of change from baseline to any follow-up visit. The model used for the sample size calculations assumes that every participant completes the month-48 visit, as an approximation for the projected mean follow-up of 4.5 years in the main START study.

Table 3. Total Sample Size for Detecting Average Treatment Differences Between the Early and Deferred Groups in Mean Change in QNPZ-8 Scores from Baseline, Averaged over Months 4, 8, 12, 24, 36, and 48; power 80 %, significance level 5%

Difference Early vs. Deferred	Total N (N inflated by 10%)			
	SD=0.5	SD=0.6	SD = 0.65	SD = 0.7
Δ = 0.10	474 (521)	682 (750)	798 (878)	926(1019)
Δ = 0.13	282 (310)	404 (444)	474 (521)	**550 (605)**
Δ = 0.15	212 (233)	304 (334)	356 (392)	414 (455)

The assumption of SD=0.7 for the standard deviation of the *change in QNPZ-8 scores* from baseline was based on the following considerations.

a. In the SMART study, QNPZ-5 scores were obtained at baseline and month 6 for 258 participants. The QNPZ-5 scores were based on four of the QNPZ-8 tests, excluding the HVLT-R, Semantic Verbal Fluency, and the WAIS-III Digit Symbol tests, plus the Timed Gait test. The standard deviation of change in QNPZ-5 scores (pooled across the two treatment groups) was 0.52, with a 95% confidence interval of 0.48 – 0.57. The value of SD=0.7 was chosen conservatively to guard against model misspecification, including misspecification of the within-participant correlation structure. Also, the somewhat different composition of the neuropsychological test battery compared to the QNPZ-5 may result in a different, possibly higher SD of change for the QNPZ-8.

b. In a different study, four neuropsychological tests were administered at baseline and month 12, including the Grooved Pegboard, Finger Tapping, Timed Gait, and the Digit Symbol Modalities tests. QNPZ-4 scores were calculated from the standardized test results. The standard deviation of changes in QNPZ-4 scores from baseline to month 12 was 0.7, 95% confidence interval of 0.6 – 0.9 in 108 HIV-infected patients (Richard Price, personal communication).

The protocol team will monitor the standard deviation of change in QNPZ-8 scores and the within-participant correlation structure, pooled across treatment groups. Sample size will be recalculated concurrently with the sample size recalculation for the main START study.

8.2.2.6 Sample Size, Enrollment and Site Selection

Enrollment and Site Selection

Enrollment into the Neurology substudy is planned to start concurrently with the main START study. The Neurology substudy aims to co-enroll participants from a limited number of sites and to co-enroll a high proportion of eligible participants at these sites. Limiting the number of participating sites makes it easier to standardize the administration of the neuropsychological tests and also is more cost effective.

It is planned to assess feasibility and re-calculate sample size for the Neurology substudy concurrently with the main START study, taking into account the observed standard deviation of change in QNPZ-8 scores and the expected duration of the main study.

8.2.2.7 Participant Selection

A total of 600 participants will be enrolled at selected sites that are geographically diverse and ensure a demographically diverse population. At these sites, it is planned to co-enroll a high proportion of the eligible START study participants.

Inclusion Criteria

- Simultaneous co-enrollment in the START study
- Signed informed consent (see Appendix A6)
- Age $\geq$ 18 years

Exclusion Criteria

Participant is unable to perform necessary tests in the protocol, in the clinician's judgment.

8.2.3 Clinical Management Issues

8.2.3.1 Administration of the Neuropsychological Tests

The research staff who will administer the neuropsychological tests will be well trained in the standardized format in which these tests should be given. Research staff will participate in follow-up training to ensure correct administration of the tests as necessary. The tests are simple to administer and the total time per study visit will take about 50 minutes.

Table 4. Expected Time Needed for Tests and the CES-D

Test	Time
Grooved Pegboard (both hands)	8 min
Color Trails 1 and 2	3-8 min
WAIS-III Digit Symbol	5 min
HVLT-R, Learning	10 min
HVLT-R, Delayed Recall	3-5 min
Semantic Verbal Fluency	3 min
Finger Tapping (Both Hands)	8 min
CES-D	5 min
**Total**	**45 - 52 min**

The ethos that will underpin the training of the clinicians who will administer these tests is that the tests are serving as measures of the health of the participants' neurocognitive and motor performance. Thus, any discussions about these tests with the participants should impart the notion that we are seeking to define their neurocognitive health rather than their neurocognitive impairment.

All efforts will be made to make the participants feel comfortable and relaxed during the testing. If participants become fatigued or anxious during the testing they will be given an opportunity to rest and the testing may be ceased prematurely at the participant's request. During the administration of the tests, participants will be positively encouraged irrespective of their performance on the tests.

The test scores for the individual tests will be collected on standardized case report forms. The z-scores, ADS scores, and CES-D scores will be calculated at the INSIGHT statistical center. Calculated test scores will not be available in real-time, and will not be returned to the clinical sites. Participants in the Neurology substudy should continue to receive screening for cognitive impairment as per local standard of care. If concerns arise during the administration of tests or the CES-D questionnaire, it is recommended that participants be referred for diagnosis and treatment according to local guidelines.

8.2.3.2 Study Withdrawal

Participants may withdraw from the study at any time at their request, as described in section 4.5 of the START protocol. A participant can withdraw from the Neurology substudy and still be followed in the START study. If a participant withdraws from the main START study, the participant will be withdrawn from the Neurology substudy.

Otherwise, participants should be followed according to protocol, even if a participant chooses not to adhere to the treatment assignment or data collection schedule.

8.2.4 Evaluation

8.2.4.1 Data Analysis

All analyses comparing the treatment groups will be by intent to treat. Comparisons between treatment groups will be stratified by geographical area of enrollment, unless sample sizes are too small or stratification is inappropriate for other reasons.

Primary Analysis
For the primary objective, mean changes in QNPZ-8 scores from baseline will be compared between the early and deferred groups using all measurements through follow-up in a longitudinal mixed-effects model, stratified by geographical area of enrollment. The model will also include “month of visit” as categorical covariate, to adjust for a possible learning effect that would occur equally in both treatment groups.

Secondary Analyses
For each of the component tests of the QNPZ-8, mean changes from baseline in unadjusted test scores and in z-scores will be compared between the early and deferred groups using all measurements through follow-up in longitudinal mixed-effects models. The slopes in these endpoints through year 1 and through follow-up will also be compared between treatment groups, using longitudinal mixed-effects models.

Additionally to the standard longitudinal mixed-effects models that assume Gaussian distributions for the random effects, changes in tests scores through follow-up will be compared between treatment groups using O’Brien’s rank-sum test for multiple endpoints.[86] The two treatment groups will also be compared for changes in test scores from baseline to last visit, and for area under the curve from baseline through follow-up for the QNPZ-8 and each of the component tests.

The early and deferred ART arms will be compared for changes in test scores (QNPZ-8, z-scores, and unadjusted test scores) within subgroups of participants defined by demographics (age, sex, race/ethnicity, education, occupation, employment status, income, urban or rural area of residence), geographic location, CD4+ cell count, HIV RNA level, and other baseline characteristics using longitudinal mixed models. Presence of differential treatment effects across subgroups will be assessed by tests for interaction between subgroup and treatment group indicators in these longitudinal models.

In order to assess presence of a lateral effect on neurocognitive functioning, for finger tapping and grooved pegboard, the interaction between treatment group indicator and indicator for dominant versus non-dominant hand will be assessed in longitudinal models with double-repeated measures (within participant, measures are repeated over time, and across hands). If there is evidence for such an interaction, it will be investigated as to whether it changes over time, for example, by fitting an additional slope parameter. Change in test scores will also be compared between the dominant and non-dominant hands within the treatment groups.

The effect of classes of antiretroviral drugs on neurocognitive functioning will be assessed by comparing the early versus deferred ART treatment effect between subgroups of participants: those whose planned initial ART regimen (determined prior to randomization) contains the drug class under investigation, and those whose planned initial regimen does not contain the drug class. This means, the effect of the drug class will be assessed through the interaction effect between treatment and subgroup indicator. Also, ART regimens will be classified by their estimated level of CNS penetration. To test the hypothesis that higher CNS penetration protects neurocognitive functioning, the interaction effect between CNS penetration and treatment group indicator will be assessed in longitudinal models.

The early and deferred groups will be compared for proportions of participants with neurocognitive impairment using longitudinal models for binary data, for example, generalized estimating equations (GEE). The ADS is an alternative measure of neurocognitive impairment. The early and deferred groups will be compared for changes in ADS using longitudinal mixed models for ordinal data. Subgroup analyses will be performed if sample size is sufficient.

The association of changes in neuropsychological test scores with baseline factors and time-varying factors will be assessed by including these factors as independent variables in longitudinal mixed models. The association will be assessed within the early and deferred groups separately and also pooled across treatment groups if there is no evidence for differential opposite-treatment effects. Follow-up time spent at various levels of the time-varying factors will be tabulated to support the interpretation of the above analyses. For example, to help interpret the association of changes in neurocognitive functioning with the estimated CNS penetration levels of the ART regimens, the CNS penetration level will be included as a time-updated covariate in longitudinal models, and also exposure time to ART regimens of different CNS penetration levels (or no ART) will be tabulated.

Additionally, changes in test scores will be summarized through follow-up (e.g., as area under the curve), and linear regression methods will be used to investigate predictors that can be identified at baseline.

Linear regression methods will be used to assess the association of baseline factors with neuropsychological test scores at baseline in cross-sectional analyses.

Alcohol use, recreational drug use, and depression influence neurocognitive functioning. Alcohol use and recreational drug use (collected in the main START study), and depression scores will be tabulated through follow-up to provide supporting information for the treatment comparisons and to describe the substudy population. Baseline values will be assessed as explanatory factors for changes in neurocognitive functioning. Treatment groups will be compared for changes in CES-D scores using longitudinal mixed models.

8.2.4.2 Data Monitoring

Data for the Neurology substudy will be monitored by the DAIDS DSMB, and study results will be reviewed simultaneously with the main START study.

The protocol team will monitor the standard deviation of change in QNPZ-8 scores and the within-participant correlation structure of the QNPZ-8 scores, pooled across treatment groups, to adjust the sample size if necessary. Additionally, the standard deviation of change in test scores, pooled across treatment groups, will be monitored for each of the component tests of the QNPZ-8, as one measure to control the quality of test administration and to identify need for re-training. The protocol team will be blinded to all other follow-up data until the closing of the START study.

8.2.5 Human Subjects

This protocol must receive the approval of the participating site's IRB or IEC prior to implementation. All study participants must sign an informed consent form (see sample in Appendix A6).

8.3 Informed Consent Substudy

8.3.1 Background and Rationale

Informed consent is widely accepted as an integral part of ethical clinical research[87,88] and includes three distinct elements: (1) disclosure of information to prospective research participants, (2) participant understanding of the information, and (3) a voluntary decision by the participant to enroll in the research. Yet, the goal of well-informed individuals making voluntary choices about research participation is often imperfectly realized. Empirical data suggest that comprehension of important study information, especially side effects and randomization of treatment, although variable, can be unacceptably low.[89,90,91,92,93,94,95] In addition, some participants do not seem to understand that participation is their choice or that they can leave a study at any time.[94,96,97,98,99,100] Somewhat paradoxically, as more evidence emerges that comprehension is inadequate, written consent documents have become increasingly long and complex. Legal, ethical, regulatory, and risk reduction authorities have all had a hand in adding language to consent documents in the interests of "protecting" the human participant as well as the research institution or sponsor.

Many agree that long and complex written documents are unlikely to enhance understanding, a goal of informed consent. Some groups have attempted to improve the consent process by improving the readability of consent documents.[101,102,103,104,105,106] Studies that compared typical consent forms to more simplified designs (usually lower in reading level and sometimes shorter) have found either equivalency between the regular and simplified forms[101,102,103] or statistically and clinically significant improvements in comprehension with a simpler form.[104,105] Studies that measured satisfaction found that subjects clearly preferred the simplified forms.[102,103]

The INSIGHT START study presents an opportunity to compare participant understanding after being randomized to either a standard consent form or a more concise consent form, across a variety of languages and international research sites. The main outcome measure will be prospective participant comprehension of the study specific information, and secondary outcome measures will include participant satisfaction with the consent process, and the resources required for this process. Demonstrating that one form is superior to the other would suggest a new standard or reinforce the current approach to writing consent forms. A demonstration of equivalent results would permit investigators to use the most efficient and easiest of equally effective alternatives.

8.3.2 Purpose

The purpose of this substudy is to evaluate understanding of study information and satisfaction with the consent process among research participants of the START protocol, after receiving information from one of two different types of consent form: a standard consent and a concise consent.

8.3.3 Study Hypothesis

Comprehension of those in the concise consent group will be at least as good as for those in the standard consent group. Satisfaction with the process will be higher for the concise group.

8.3.4 Study Design

This is a randomized study to evaluate and compare the following two groups in START:

- A **standard group** in which participants receive the standard detailed consent form (see Appendix A1) and discuss the study with one or more members of the research team.
- A **concise group** in which participants receive a shorter, less-complex, consent form (see Appendix A7) written at a lower reading level, and discuss the study with one or more members of the research team.

Both the standard and the concise forms contain all of the required elements of informed consent according to the federal regulations found at 45CFR46.116 and 21CFR50.25, and include information needed to make a decision about participating in START.

Consent documents and the questionnaire assessing responses will be translated into the primary language(s) spoken at participating sites.

START Informed Consent Substudy Schematic

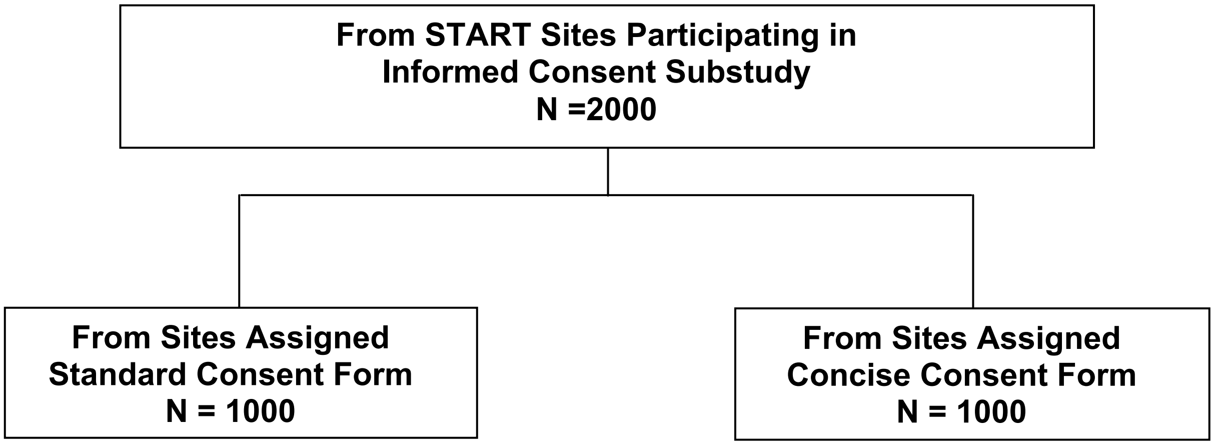

Data collection: Following consent and prior to randomization in main START study

8.3.5 Eligibility Criteria for Sites

- Sites will choose whether or not to participate in the Informed Consent substudy. Interested sites will complete a site questionnaire.

- Sites that choose to participate in the Informed Consent substudy must be registered for both the main START study and the Informed Consent substudy. They must also register to any amendments of either the main study or the substudy.
- There must be at least two willing sites whose primary language is the same.
- Sites may be excluded after review by substudy investigators in cases where the IRB/ IEC stipulates revisions to either consent document that significantly alter the length or readability, making comparison difficult.

8.3.6 Randomization of Participating Sites

For scientific reasons, to minimize contamination, the unit of randomization will be the site rather than the individual participant. Sites will have the option of participating in the consent substudy. Sites that choose not to participate in the substudy will use the standard consent template. Sites that choose to participate in the substudy will submit both the standard and concise forms to their IRB. All reviewing IRBs/IECs will be informed that the consent documents are part of a randomized assessment of understanding and asked to consider minimizing stipulated changes to the consent documents. After IRB/IEC approval, the site will register to START and the Informed Consent substudy and will submit both consents for approval, as per usual procedures. The Informed Consent substudy team will review any changes made to consent documents to ensure that the two documents remain sufficiently different. Similar processes will be followed for any amendments to the consent if the protocol changes.

After registration, the site will be randomized to either the standard form or the concise form. A stratified randomization scheme will be used. Eligible sites will be grouped by primary language of participants, and sites will be centrally assigned to use the concise or standard consent (1:1 allocation) by block randomization within stratum. The site will be notified of their consent form assignment when they receive notification from the SDMC that they are open to randomize participants to START.

The substudy will randomize by site in order to enhance the ability to compare the two consent documents and decrease the possibility that those obtaining consent will alter their usual process because of the substudy. Additionally, randomization by site will minimize the logistical burden at the site because the same consent form and process will be utilized for each participant.

8.3.7 Data Collection

Each randomized site will only use the consent document to which it was assigned for all participants at that site.

Each participant will complete a self-administered questionnaire immediately after giving consent to START but before START randomization. The questionnaire will take approximately 15 to 20 minutes to complete. Consent substudy objectives will be measured using responses to this questionnaire. Research participants' comprehension, especially of study purpose, risks and benefits, randomization, and their right to withdraw will be measured by answers to a series of knowledge questions.

The secondary outcomes will be measured using questions related to satisfaction with the consent process and voluntariness of participation decisions.

The number of individuals completing the questionnaire and the number who refused or failed to complete it will be summarized. Basic information available for those individuals who sign consent at each site (e.g., CD4+ cell count, limited demographic information, whether or not eligible, and whether or not randomized) will be tabulated to assess any differences between the two groups.

For each consented participant, site staff will record the approximate amount of time spent in the consent process, who obtained consent (e.g., the site leader, study coordinator, etc.) and the extent to which the person who obtained consent used the written document. In addition, the site leader will be asked to complete a brief questionnaire describing general site and consent process characteristics.

In addition, the number and type of changes made to the final IRB/IEC approved consent document from each participating site will be compared for length and readability to the sample consent distributed to each site.

8.3.8 Differences between Standard Consent and Concise Consent for START

Both the standard and the concise consent forms present information needed to make a decision about participating in START and contain all of the required elements of informed consent according to the federal regulations found at 45CFR46.116 and 21CFR50.25. These required elements include: statement that activity is research, the purpose and procedures, anticipated risks, possible benefits, alternatives, whom to contact for information, how confidentiality will be protected, how research injury will be handled, and that participation is voluntary. Both forms also include “additional” elements related to unforeseeable risks, circumstances under which participation would be terminated, additional costs, approximate number of participants, and that significant new findings will be provided to participants.

The concise form attempts to present this information in a way that reduces repetition, simplifies explanations and terminology, reduces the reading level, shortens the form, and uses formatting, tables, bullets, etc. to facilitate understanding. It is approximately one-third the length of the standard form.

8.3.9 Study Outcome Measures

The primary endpoint will be the proportion of participants giving correct answers to questions about randomization. An important secondary endpoint will be based on mean scores on the comprehension section of the instrument. Comprehension will include questions on study purpose and procedures, randomization, risks and side effects, and right to refuse. A comprehension score will be based on the number of correct answers to the knowledge questions.

Other secondary endpoints will be measured by a composite score of the satisfaction questions, a composite score of the voluntariness questions, and an evaluation of IRB/IEC required changes to the consent documents.

8.3.10 Sample Size Estimation

Sample size was estimated taking into account the between-site variance in the proportion responding correctly to questions about randomization, the number of participants to be enrolled at each site, the expected percent of participants given the standard consent who will understand the randomization plan, and the expected difference in the proportion understanding the randomization plan between the standard and concise consent form groups.

Cluster randomized trials require a larger sample size than trials with individual randomization. Compared to an individual randomized trial, the sample size must be inflated by an amount that depends on the cluster size and the intra-site correlation coefficient. The intra-site correlation is the amount of site to site variability compared to the total variability.

There is limited information available on site to site variability in the comprehension of randomization, the primary response variable for the study. Data from a consent substudy of a large international trial, Evaluation of Subcutaneous Proleukin® in a Randomized International Trial (ESPRIT), conducted in 2003 was used to estimate sources of variability in the comprehension of randomization. The percent of participants responding correctly to two questions about randomization for sites that enrolled at least ten participants was assessed. Seventeen sites enrolled at least ten participants and on average 50% of the 426 participants at these sites answered both questions correctly. There was considerable variability in the response among sites with the percent of participants responding correctly ranging from 18% to 87%. The estimated coefficient of variation for true proportions between sites was 0.30; the estimated intra-site correlation coefficient was 0.09 (personal communication, Deborah Wentworth).

Sample size for the START substudy is estimated using an approach described by Hayes and Bennett.[107] They use the between-site coefficient of variation (standard deviation divided by mean) to estimate sample size. Their approach is equivalent to use of the intra-site correlation coefficient. If a coefficient of variation of 0.30 is assumed and each site enrolls 25 participants, then 58 sites (29 assigned to use the concise consent and 29 assigned to use the standard consent) are required to detect a difference of 15% of participants responding accurately to the questions concerning randomization (50% versus 65%) with power of 0.80 at the two-sided level of significance of 0.05 (i.e., a total of 1,450 participants). If the coefficient of variation is 0.20, then a total of 34 sites are needed with these assumptions (850 participants). For comparison, if participants were individually randomized, a total of 340 participants would be required.

Sample size assumptions were assessed after approximately 500 participants were enrolled by 70 sites; the coefficient of variation was 0.17. Most of the participants enrolled had a primary language of English, Spanish or German. Since there is interest in obtaining data for other primary languages (e.g., Thai, Greek, Portuguese), for sites in developing countries, and for other subgroups, and since the substudy has proven to be very acceptable to participants and site staff, the Informed Consent substudy decided to offer new sites in START the opportunity to participate in the substudy. This will enhance the generalizability of the substudy.

Given the current site participation and enrollment per site, it is estimated that 125-150 sites will participate in the substudy with an average enrollment of 15 participants (an estimated 2,000 participants). This will provide greater than 90% power for the primary endpoint comparison related to comprehension of randomization and allow reliable comparisons between the standard and concise groups for other measures of comprehension that are being assessed as well as subgroups of interest.

8.3.11 Data Analysis

The number and characteristics of sites assigned to use the standard and concise consent will be summarized. Baseline site characteristics will include primary language of participants and site staff and the background of the person administering the consent (e.g., physician, nurse, coordinator). Interested sites for which the Informed Consent substudy or sample informed consents were not approved by local ethics committees will also be summarized.

For sites participating in the Informed Consent substudy, demographic characteristics (e.g., age, gender, ethnicity) of participants consented will be compared. In addition, the number of participants at each site who refused to complete the post-consent questionnaire will be tabulated. Median (IQR) site size (number of participants consented with completed questionnaires) for each treatment group will be summarized.

Logistic regression appropriate for cluster randomized trials (hierarchical models) will be used to compare the standard and concise consent groups for the primary outcome. Odds ratios (concise versus standard consent) and 95% confidence intervals will be cited. Mean levels of continuous secondary outcomes will be compared using methods that account for the within-site correlation. The intra-site correlation coefficient will be cited for the primary and each secondary outcome.

The primary analysis will include all participants who completed the post-consent questionnaire. In a secondary analysis, participants who were consented and did not complete the questionnaire will be considered. In these analyses, participants will be counted as if they did not comprehend the consent questions.

Subgroup analyses will be conducted by primary language, the nature of differences between the standard and concise consent as judged by the Informed Consent Substudy team, age, gender, race/ethnicity, education, and previous participation in

research studies. Tests of heterogeneity will be performed to document differences among subgroups.

8.3.12 Administration of the Questionnaire

Individual participants will complete the questionnaire immediately after consenting to START, and return it to the research team when completed.

8.3.13 Confidentiality of the Questionnaire Responses

Questionnaires will be coded with the study PID. No other identifying information will be included on the questionnaire. If copies of the questionnaire are kept at the site after data are transmitted, they will be aggregated and kept separate from the participant records.

8.3.14 Study Alternative for Sites and START Participants

A site can choose not to participate in the Informed Consent substudy. Participants at each participating site will be asked to complete a questionnaire after they give consent; however, individual participants can refuse to complete the questionnaire.

8.3.15 Human Subjects

The Informed Consent substudy will be approved by the participating site's IRB/IEC prior to implementation.

Participants will be informed in the consent document that their site is participating in a consent substudy. They will be asked to complete the questionnaire as part of that study, and will be informed that they can decline without affecting their participation in START.

8.3.16 Benefits

Participants will not benefit directly from participation in this substudy, but may contribute to a better understanding of the informed consent process and development of improvements for the future.

8.3.17 Risks

There are no anticipated risks to the participants from completing the substudy questionnaire.

APPENDIX A: SAMPLE CONSENT FORMS

A1. START Main Study Sample Consent

A2. START Study Specimen Storage (Plasma & Urine) Sample Consent

A3. START Study Specimen Storage (Biopsy Tissue & Blood) at Diagnosis of New Malignancy Sample Consent

A4. START Genomics Substudy Sample Consent

A5. START Genomics Substudy Sample Withdrawal Letter

A6. START Neurology Substudy Sample Consent

A7. START Informed Consent Substudy: Sample Concise Consent for Main Study

START MAIN STUDY SAMPLE CONSENT

University of Minnesota: SPONSOR
NIAID: PRIMARY FUNDER

Protocol Title:
Strategic Timing of AntiRetroviral Treatment
(START)

A Multicenter Study of the
International Network for Strategic Initiatives in Global HIV Trials
(INSIGHT)

Short Title of the Study: START

CONSENT FOR PARTICIPATING IN AN NIH-FUNDED RESEARCH TRIAL

SITE LEADER: ___________________________________ PHONE: __________

ALL SITE INSTRUCTION THAT IS INCLUDED IN A TEXT BOX SHOULD BE REMOVED FROM THE SITE'S INFORMED CONSENT FOR SUBJECTS

OHRP Requirements to be read by the sites:

PLEASE NOTE THAT THIS SAMPLE LANGUAGE DOES NOT PREEMPT OR REPLACE LOCAL IRB REVIEW AND APPROVAL. INVESTIGATORS ARE REQUIRED TO PROVIDE THE LOCAL IRB WITH A COPY OF THIS SAMPLE LANGUAGE ALONG WITH THE LANGUAGE INTENDED FOR LOCAL USE. LOCAL IRBS ARE REQUIRED TO WEIGH THE UNIQUE RISKS, CONSTRAINTS, AND POPULATION CONSIDERATIONS AS A CONDITION OF ANY APPROVAL. ANY DELETION OR SUBSTANTIVE CHANGE OF INFORMATION CONCERNING RISKS OR ALTERNATIVE TREATMENT MUST BE JUSTIFIED BY THE INVESTIGATOR, APPROVED BY THE LOCAL IRB, AND NOTED IN THE IRB MINUTES. JUSTIFICATION AND IRB APPROVAL OF SUCH CHANGES MUST BE FORWARDED TO THE INTERNATIONAL COORDINATING CENTER.. SPONSOR-APPROVED CHANGES IN THE PROTOCOL MUST BE APPROVED BY THE LOCAL IRB BEFORE USE UNLESS INTENDED FOR THE ELIMINATION OF APPARENT IMMEDIATE HAZARD. NEW INFORMATION SHALL BE SHARED WITH EXISTING SUBJECTS AT NEXT ENCOUNTER, WITH ALL NEW SUBJECTS PRIOR TO INVOLVEMENT, OR AS THE LOCAL IRB MAY OTHERWISE ADDITIONALLY REQUIRE.

INTRODUCTION

You are being asked to take part in the START study because you are infected with HIV, the virus that causes AIDS, and because you have never taken medicines to treat your HIV infection. This study is being done to find out the best time to start taking HIV medicines. The study will look at whether starting HIV medicines early when your CD4+ cell count is fairly high may help you stay healthy longer than waiting for your CD4+ to drop. Before you can decide whether or not to take part in this study, we would like to explain the purpose of the study, how it may help you, any risks to you, and what is expected of you.

YOUR PARTICIPATION IS VOLUNTARY
This consent form gives you information about the study that will be discussed with you. Once you understand the study, and if you agree to take part, you will be asked to sign this consent form. You will be given a copy to keep.

Before you learn about the study, it is important that you know the following:
- Your participation is entirely voluntary;
- You may decide not to take part or to withdraw from the study at any time without losing the benefits of your routine medical care.

This study is being funded primarily by the National Institute of Allergy and Infectious Diseases (NIAID), U.S. National Institutes of Health (NIH), through a grant to the University of Minnesota, which is the lead institution in the INSIGHT group. The University of Minnesota is the sponsor of this study. This study is also being conducted with additional funding from the Agence Nationale de Recherches sur le SIDA et les Hépatites Virales (ANRS, France); Bundesministerium für Bildung und Forschung (BMBF, Germany); the Australian National Health and Medical Research Council (NHMRC), the Department of Bioethics at The Clinical Center at NIH, NEAT - European AIDS Treatment Network; National Heart, Lung, and Blood Institute; National Institute of Mental Health (NIMH); National Institute of Neurological Disorders and Stroke (NINDS); and the Division of Clinical Research (NIAID). Additional support is being provided by Abbott Laboratories, Inc.; Bristol-Myers Squibb; Gilead Sciences, Inc.; GlaxoSmithKline, Inc.; Merck & Co., Inc, and Tibotec Pharmaceuticals, Ltd.

[The following language should be inserted at sites which are participating in the Informed Consent substudy of START.]

We are trying to learn which of two types of consent form is best to help people understand what the START study is about and what they have to do if they join the study. By reading this consent, if you choose to sign it, you are also taking part in a substudy about the consent form itself. Everybody at this site gets the same consent form that you are reading now. Several sites are doing this substudy. Some of them will use this consent form, and some of them will use the other consent being studied. You will be asked to complete a questionnaire about how well you understand the START study and how you felt about the consent process. You can refuse to complete this questionnaire and still be in the START study.

Site instruction:<br>*If your site is participating in the Informed Consent substudy, please work with your IRB to modify the consent language as little as possible from this template. This will strengthen the validity of the comparison of the two consents.*

WHY IS THIS STUDY BEING DONE?
Most guidelines agree that if the number of your CD4+ cells (cells in your blood which help fight infection) drops below 350 cells/mm^3, or if you have symptoms of AIDS, you

should start taking HIV medicines. There are randomized trials that support this recommendation. (Randomized trials are usually considered the strongest form of evidence to support treatment decisions. Other studies, like observational studies, provide evidence too, but the evidence is often considered to be weaker than evidence from randomized trials. A randomized trial gives the most certain information about how well a treatment works because randomization makes sure each group is similar except for the treatment they receive.) Some experts believe that HIV treatment should be started even when the number of CD4+ cells is above 350 cells/mm^3. For example, guidelines issued in the US in December 2009 include a new recommendation for starting HIV medicines if your CD4+ cell count is between 350 and 500 cells/mm^3. However, this recommendation is based on information from observational studies, not randomized trials.

It is unusual to develop symptoms of AIDS when your CD4+ cell count is above 350, although it does happen in some people. Studies have shown that other serious illnesses, like heart attacks, liver disease, or kidney failure, also happen less often in people whose CD4+ cell count is higher. In these studies, the chance of having one of these serious illnesses appears to increase as your CD4+ cell count drops. In some studies, people who took HIV medicines and kept their CD4+ cell counts high had fewer of these serious illnesses than people not taking HIV medicines. We do not completely understand why this happens.

We are doing this study to find out if the chances of getting one of these serious illnesses or of getting AIDS are less if you start taking HIV medicines at a time when your CD4+ cell count is still fairly high, instead of waiting to take HIV medicines at a CD4+ count where there is good evidence for starting medicines.

We will also try to learn more about how this strategy of starting HIV medicines early might affect other things, such as your chances of developing other illnesses or resistance to HIV medicines (where the HIV virus changes so that some medicines no longer work against it), how often you need to see a doctor, the cost of your medical care, and your general health and satisfaction with your life.

HOW MANY PEOPLE WILL TAKE PART IN THE STUDY?
We expect that we will need about 4,000 people from around the world to answer this question.

HOW LONG WILL YOU BE IN THE STUDY?
Right now we think it will take about 3 years to enroll the study, and at least 3 years of follow-up after that to complete the study. You will continue to be followed in the study group to which you were assigned at the beginning, until the study comes to an end. An independent Data and Safety Monitoring Board (DSMB) will review results of the study at least once per year. The DSMB is made up of doctors and other people with a good understanding of HIV and clinical trials, and they are responsible for reviewing the study results to make sure the study continues to be safe and likely to answer the study

question. The DSMB may decide to stop the study when they review it if they do not think the study will be able to reach an answer.

HOW WILL THE STUDY WORK?
If you qualify for this study, you will be assigned by chance (like flipping a coin) to one of two treatment groups. You will have an equal (1 to 1) chance of being assigned to either of the two groups. These are the groups:

1) If you are assigned to the EARLY treatment group, you will start taking HIV medicines right away after enrollment. This is the experimental (research) group.

2) If you are assigned to the DEFERRED treatment group, you will wait to start HIV medicines until your CD4+ cell count drops to below 350 cells/mm^3, or until you develop AIDS or other symptoms of HIV infection. There is strong evidence from randomized trials to begin HIV medicines at that time. This is also called the "control group."

However, if you and your doctor agree that there is a good reason to start HIV medicines before your CD4+ cell count drops to below 350 cells/mm^3, you can begin taking HIV medicines at any time during the study and still be in the study. You may develop medical problems that might be related to the HIV virus or your general health. It may be better for you to start HIV medicines for these reasons.

No matter which group you are assigned to, the specific HIV medicines you take will be decided by you and your doctor. Your first HIV medicine regimen will be chosen from a list of the best regimens that doctors currently recommend for people starting treatment. If you need to change HIV medicines during the study, you and your doctor will decide what HIV medicines are best to take next.

The University of Minnesota, the sponsor of this research, gets royalties (payments) from the use of abacavir, one of the HIV medicines that can be used in this study. The sponsor will not tell you or your doctor if you should take abacavir. Only you and your doctor will decide which HIV medicines you will take.

[If applicable: While you are taking part in this study, you may be asked to take part in some smaller related research studies. You may refuse to take part in these smaller studies and still be in this main study.]

WHAT DO YOU HAVE TO DO IF YOU ARE IN THIS STUDY?

Screening

Site Instruction:<br>*It is suggested that you tell potential participants how long screening and follow-up visits are likely to take.*

You may need two or more clinic visits within 60 days before you can be randomized, to see if you are eligible to be in the study. You must have two CD4+ cell counts at least two weeks apart to be eligible for the study. Both of these must be higher than 500 cells/mm^3.

Your study doctor or nurse will ask questions about your health and your HIV infection and will give you a short medical examination. You will have a total of about 65-85 mL (about 5-6 tablespoons) of blood drawn. Some of the blood will be used to measure the amount of HIV in your blood, CD4+ cell counts, CD8+ cell count (another type of blood cell that is affected by HIV), and other routine tests. Some blood will be tested to see if you are infected with hepatitis B or hepatitis C (liver diseases caused by viruses), if you have not already had these tests. We will also do tests to check how your liver and kidneys are working, along with testing the amount of sugar and the types of lipids (fats) in your blood. For the blood tests for sugar and lipids done at this second visit we will ask that you not have anything to eat and only water to drink for at least 8 hours before the visit. You will also be asked for a urine sample, which will be tested to see if it has proteins in it which may indicate you have a kidney problem. These results will be shared with you at your next study visit or when they become available.

Site instruction:
If your site does not have or will not be receiving a study-supplied ECG machine and a certified technician, please remove the text in italics in the next paragraph. If your site ***will*** *be doing ECGs, please change the text to not be in italics.*

Y*ou will also have an electrocardiogram (ECG), a routine test that allows the doctor to look at the rhythm of your heart. This involves lying on a table and having 10 small electrodes stuck to your skin for at least 5 minutes while the test is done.* You will be asked what medicines you are taking. You will be asked questions about how often you see the doctor or go to the hospital. You will be asked to answer a questionnaire about how you feel physically and emotionally (a "quality of life" questionnaire). You will be asked about sexual or other behaviors that may infect other people with HIV, and you will receive counseling about how to avoid infecting others. You will be asked whether and how much you smoke, and about your use of alcohol and other drugs. Your answers to these questions will not affect your medical care or your participation in the study.

Your doctor will discuss HIV medicines with you. You and your doctor will decide which specific HIV medicines you will take if you are randomized to start treatment right away. Your doctor will go over the possible side effects of your treatment and how you should take your HIV medicines.

If you and your doctor decide you should take a medicine containing abacavir (Ziagen®, Epzicom®, Kivexa®, or Trizivir®), you will have another blood test to see if you carry the HLA-B*5701 marker. People who have this marker are more likely to have a bad

reaction to abacavir. Your doctor will need to have the result of this test before your start taking abacavir, and if you have the marker you will not take abacavir.

If you have had a test showing what medicines your HIV virus might not respond to ("resistance testing"), the results of this test will be recorded for the study.

If you are a woman who can get pregnant, you will have a pregnancy test done using either blood (about 1 teaspoon) or a urine sample. This will be done at the clinic within 14 days before you are randomized. If you are pregnant or breastfeeding you cannot be in this study, as it would be unsafe for you to do so.

If you consent, you will give an additional 15-30 mL (1-2 tablespoons) of blood from a vein and a small urine sample, to be stored and used for additional tests in the future, looking at information important to HIV research.

Follow-up visits
No matter which group you are assigned to, you will return to the clinic at about 1 month and 4 months after you are randomized, and at least every 4 months after that until the end of the study. At these visits, your study doctor or nurse will ask you about your general health and whether you are having any side effects from your HIV medicines (if you are taking these). You will have a brief physical exam and about 5-10 mL (about 1-2 teaspoons) of blood will be drawn. This blood will be used to measure the amount of HIV in your blood and your CD4+ cell count. You will also be asked for a urine sample to be tested for protein. These results will be shared with you at your next study visit or when they become available. If you are taking HIV medicines, you will also be asked about how much of your prescribed medicine you have taken.

If you have had a test showing what medicines your HIV virus might not respond to ("resistance testing"), the results of this test will be recorded for the study.

If you consent to have blood and urine stored for this study, at each study visit you will have 15-30 mL (1-2 tablespoons) of blood stored. At visits in the first year of the study, and once a year after that, you will also have a small urine sample stored.

At your Month 4 visit and at every annual visit, you will be asked to answer the "quality of life" questionnaire about how you feel physically and emotionally. You will also be asked about sexual or other behaviors that may infect other people with HIV and receive counseling about how to avoid infecting others.

For visits in the first year of the study, and once a year after that, you will have another 10-15 mL (about a tablespoon) of blood drawn to see how your kidneys are working.

Once a year at your study visit you will also have an extra 30-45 mL (2-3 tablespoons) of blood drawn to test how your liver is working and to look at the amount of sugar and types of lipids in your blood. You will be asked to not eat anything for at least 8 hours

before these tests for sugar and lipids, and to not drink anything except water during that time.

> *Site instruction:*
> *If your site does not have or will not be receiving a study-supplied ECG machine and a certified technician, please remove the text in italics in the next paragraph. If your site* ***will*** *be doing ECGs, please change the text to not be in italics.*

At this annual visit, *you will have another ECG*. You will be asked what medicines you are taking. You will be asked about your smoking status. If you are a woman who can get pregnant, you will be asked if you have been pregnant, and if so, how that pregnancy turned out. You will be asked questions about how often you see the doctor or go to the hospital. You will be asked about the use of alcohol and other drugs. Your answers to these questions will not affect your medical care or your participation in the study.

Throughout the study
It is very important to let your study doctor or nurse know right away if you are sick or injured or in the hospital. This is important for your safety, and also for the study to learn more about illnesses that happen to people with HIV who are treated early (or defer treatment) for their HIV infection. Your study doctor or nurse will ask you for permission to get medical records from your other doctors or from the hospital (if you were in the hospital). You will be asked to sign a “Release of Medical Information” form to allow other doctors or hospitals to share this information with the study team.

You should tell your study nurse or doctor before you take any other medicines or dietary supplements or enroll in other clinical trials. This is important because some medicines should not be taken together. Your study doctor or nurse will help figure out what medicines and supplements are safe for you to take.

You doctor may decide to see you more often than required for the study based on your needs.

> *Site Instruction:*
> *Please remove the Stored specimens section that follows if your IRB requires a separate consent for stored specimens*

Stored specimens for future research
As part of the START study, you will be asked to have blood and urine samples stored in a safe and secure laboratory in the United States for use in future research related to HIV infection, its complications, and the immune system. You and your doctor will not receive any results from tests done on these stored samples. No tests of your genes (DNA) will be done on these samples. These samples will not have any information on them that can identify you by name. There is no time limit on how long your samples

will be stored. You can still be in the START study even if you do not want to have samples stored.

If you consent to have specimens stored for this study, we will collect an additional 15-30 ml sample of blood (about 1-2 tablespoons) at screening and at each study visit. We will also collect urine at baseline and at study visits at months 1, 4, 8, and 12, and every 12 months afterward. We will also collect a 10-mL sample of blood (about 2 teaspoons) if you change HIV medicines because of the amount of HIV virus in your blood or because resistance mutations have been detected. You will be asked for this sample before you start taking your new HIV medicines. This blood will be used sometime in the future to confirm whether your HIV virus had stopped responding to the HIV medicines you were taking.

HOW WILL YOU GET HIV MEDICINES FOR THE STUDY?
When it is time for you to take HIV medicines – right away if you are in the early group, or later on in the deferred group – your study doctor or nurse will help you find out if you can get HIV medicines through your insurance or some other program or if the study will provide HIV medicines to you.

WHAT IF YOU MOVE?
If you move or transfer your medical care to another doctor, the study staff would like to continue to collect information about your health. If you give permission, your study doctor or nurse will contact your new doctor and ask him or her to provide information about your health. The types of information your new doctor will be asked for are routine things, such as results of laboratory tests (for example, CD4+ cell count and viral load), what medicines you are taking, and whether you have been sick. When you move, you will be asked to sign a release of medical information form to allow your new doctor to share this information with the study team. Your new doctor may also ask you to sign a release form.

WHAT ARE THE RISKS AND/OR DISCOMFORTS OF THIS STUDY?
This section describes risks or discomforts that you may experience if you decide to be in this study. There may be additional risks to you (or to your baby, if you are a woman and become pregnant while on the study) that we have no way of knowing about right now. If additional risks or benefits are found during the study, your study doctor or nurse will let you know about them right away.

Possible risks of both ways of treating HIV in the study
Each way of treating HIV disease in this study may be associated with possible benefits and risks. It is not known in the long run which of these strategies will be less risky.

<u>Early group</u>: The long-term risks of using HIV medicines are not clear, especially in people with higher CD4+ cell counts like those joining this study. People who take HIV medicines over many years will probably have more side effects than people who take HIV medicines for shorter periods of time. Also, you may find it hard to take HIV medicines according to your doctor or nurse’s directions for many years, which may

lead to your HIV virus becoming resistant to some medicines used to treat it. Because of this, you may have fewer HIV medicines you can use when the risk of disease is high.

Deferred group: The long-term risks of **not** using HIV medicines are not clear. People who do not take HIV medicines will probably have a drop in their CD4+ cell count. There may be a greater chance of developing symptoms of AIDS or other serious illnesses if the CD4+ cell count drops too much. People who do not take HIV medicines may also have an increase in the amount of HIV virus in their blood ("viral load"). People with high viral loads may be more likely to be able to pass the virus to others.

Risks of HIV medicines
All HIV medicines can cause side effects. Your doctor or nurse will discuss with you the risks of the specific HIV medicines that you take. These risks are not specific to this study; they are associated with taking these medicines whether you are in the study or not.

It is also possible for the HIV virus to develop resistance to any anti-HIV drug. It is not known if either way of treating HIV in this study will lead to resistance to more HIV medicines over time.

It is possible that someone could inadvertently find out that you are infected with HIV if you are taking HIV medicines and someone in your household or at work notices you taking them.

Risks of medicine interactions (where one medicine affects how another works)
For your safety, you must tell your doctor or nurse about all medicines, including prescription, over-the-counter (non-prescription), herbal or alternative medicines, and dietary supplements you are taking. This is because there may be serious side effects when other medicines are taken with HIV medicines. Also, please let your nurse or doctor know before you enroll in any other studies while on this study.

Risk of transmitting HIV
Using HIV medicines does not necessarily affect your ability to transmit HIV to other people. You should continue to use precautions to make sure you do not infect someone else. Your study doctor or nurse will tell you about how to protect yourself and other people.

Risks of blood drawing
The risks of having blood taken include pain, bleeding, bruising, lightheadedness, anxiousness, and in rare cases fainting or infection or a blood clot where the needle enters the body. You may feel some anxiety while waiting for your test results to be available. You will have blood tests like those in this study done as part of your usual care, even if you decide not to be in this study.

WHAT ABOUT PREGNANCY AND BREASTFEEDING?

If you are a woman who can become pregnant, you should know that there are some HIV medicines that are risky to take during pregnancy. If your doctor or nurse thinks that one of these medicines is the best choice for you to treat your HIV infection, she or he will talk to you about these risks and about contraception and other options for HIV medicines. In order to be in this study, you must be willing to use appropriate birth control (contraception) if you are taking HIV medicines. For some medicines, this may mean you need to use two types of birth control at the same time.

Pregnant women cannot join the study since we do not know whether these treatment strategies are safe for them or their unborn babies. If you are a woman who is able to become pregnant, you must have a negative pregnancy test before you join this study. If you become pregnant during the study, tell your study doctor or nurse right away. You may go to another doctor to help manage your pregnancy if you choose to do so. You will be asked to continue your study visits.

Women who are breastfeeding cannot join the study since the effects on the baby from the HIV medicines that are in breast milk are not known.

WHAT ARE THE BENEFITS OF THIS STUDY?

If you take part in this study, there may be a direct benefit to you, but no guarantee can be made. It is also possible that you may receive no benefit from being in this study. What we learn from this study may help us to improve the treatment of other people who are infected with HIV.

Benefits of taking HIV medicines

While not every doctor necessarily agrees with all of the treatment guidelines, very few doubt that when properly used, HIV medicines can bring about an increase in CD4+ cell count and decrease in viral load in most people. So, if you are taking HIV medicines in this study, you may have an increase in your CD4+ cell count and a decrease in your viral load. Because of this, you might stay well for a longer time, be less likely to develop symptoms of HIV infection or infections associated with HIV, and may be less likely to be able to transmit HIV to other people.

Benefits of not taking HIV medicines

If you are not taking HIV medicines, you will not have any of the side effects that HIV medicines can cause. You also will not have to cope with any of the other issues of HIV medicines, like timing when to eat so you can take your medicines.

Benefits of being in a research study

By being in a research study, you may find out sooner about treatments, services, or other things that could help you live with your HIV infection than you would if you were not in a research study. You might have access to tests or treatments that would not otherwise be available to you. You might also feel good about yourself for participating in research that might improve not only your own health but also the health of other people, including people who are close to you.

Benefit to others
In addition to any direct benefit you may receive, your participation in this research may lead to benefits for other people infected with HIV. By finding out the best way to use HIV medicines, this research could lead to better treatment for HIV.

WILL HIV MEDICINES BE PROVIDED AFTER THE STUDY?
After you complete this study, if you and your doctor decide it is best for you to take HIV medicines, this is how you will get your medicines: If you got your medicines through your insurance or national health program during the study, you will need to continue to get your medicines this way after the study. If the study gave you medicines, you will continue to get HIV medicines through the study for up to 6 months after the study is completed. After this time, neither the primary study funder of this study (DAIDS) nor the drug companies that make HIV medicines will provide HIV medicines to you. This 6-month time period will give your doctor time to arrange for you to get your medicines another way.

Site Instruction:<br>*Please include specific information about how your site will provide ART to participants after the study ends.*

WHAT IF THERE ARE NEW FINDINGS?
You will be told about any new information learned during the study that might cause you to change your mind about staying in the study. At the end of the study, you will be told when study results may be available and how to learn about them.

WHAT IF YOU DON'T WANT TO BE IN THE STUDY ANY LONGER?
If you enroll in this study, you may decide to stop participating at any time. Withdrawing from this study will not affect the benefits of your regular medical care. However, if you are receiving HIV medicines provided by the study, you will not continue to be given HIV medicines through the study after you withdraw. Your doctor or nurse will help you find another way to get HIV medicines.

CAN YOUR STUDY PARTICIPATION BE STOPPED WITHOUT YOUR CONSENT?
You may be taken off of HIV medicines or started on HIV medicines during the study before you normally would be if your doctor believes it is the best thing for you. You may be taken off the entire study without your consent if:

- Your study doctor decides that continuing in the study would harm you;
- The study is cancelled by the sponsor (the University of Minnesota), the National Institute of Allergy and Infectious Diseases (NIAID), regulatory authorities in your country, or your site's Institutional Review Board (IRB)/Ethics Committee(EC);
- You are in jail or prison; or
- Other administrative reasons.

WHAT OTHER CHOICES DO YOU HAVE BESIDES THIS STUDY?
Before you decide to take part in this study, the study doctor or nurse will talk with you about the other options that are available to you. Possible options include:

- Not taking any treatment for your HIV infection at this time;
- Taking HIV medicines using current standards and guidelines.

Site Instruction:
In the next paragraph, insert specific information about what HIV medicines will be provided for free, if any. If any information in this paragraph is not correct for your country/site, please revise the text to explain to the subject any costs to them that may result from participating in the study and obtaining their ART.

WHAT ARE THE COSTS TO YOU?
During the study, *<site should insert specific information about what HIV medicines will be provided for free, if any>.* You, your insurance company, or some other third-party payer must pay for all other medicines, including HIV medicines not listed above and medicines needed to prevent or treat other illnesses. We will provide all clinical and professional services, lab work, and other tests that are part of this study and not part of your regular care at no cost to you.

HOW IS YOUR PRIVACY PROTECTED?
Researchers will take every reasonable step to protect the privacy of your health information and to prevent misuse of this information. You will not be identified by name or any other way in any publication about this study. You will be identified only by a code, and personal information from your records will not be released without your written permission. We will collect dates of your study visits and of hospitalizations and certain illnesses so that we can answer the study questions as accurately as possible. We will use your initials as a check on the number code assigned to you to make sure all of your information stays together. If you wish, you may ask your study doctor or nurse to use any 3-letter code instead of your initials and this will not affect your participation in the study or your regular medical care.

[The following paragraph must be included at U.S. sites only]
In addition to these efforts to keep your information private, the START study and its substudies are covered by a Certificate of Confidentiality from the U.S. Department of Health and Human Services. This certificate means that researchers cannot be forced to give information collected as part of this study to people who are not involved with the study, such as the court system. However, this certificate has limited protection rights. You should know that it does not stop the doctor in charge of this study from taking appropriate steps to prevent serious harm to yourself or others.

[The following paragraph must be included at international sites only]
Efforts will be made to keep your personal information private, but we cannot guarantee complete confidentiality. Your personal information may be released if required by law. Any publication of this study will not use your name or identify you personally.

Your medical and research records may be reviewed by the *[insert the name of the site]* ethics committee (institutional review board, IRB), the U.S. National Institutes of Health (NIH), the U.S. Office for Human Research Protections (OHRP), and the research staff and monitors, and their designees. Also, the research staff at *[insert the name of the site]* is required to make sure that people not involved with this study do not have access to your research and medical records while collecting personal information about you. They will keep your files in a locked cabinet in a safe place and will handle your personal information very carefully. This will also help to protect your privacy.

Site Instruction:
If the information in the next paragraph is not correct for your country/site, please revise the information to inform the subject of the following: 1. what treatment will be provided to the subject; 2. who will pay for the treatment; 3. if there is any plan for compensation for research-related injury issues, such as lost wages, etc.

WHAT IF YOU ARE INJURED?
If you are injured because of being in this study, *[insert the name of the clinic]* will give you immediate necessary treatment for your injuries. The cost for this treatment will be charged to you or your insurance company. You will then be told where you may receive additional treatment for injuries. There is no program for monetary compensation. You do not give up any of your legal rights by signing this form.

WHAT IF YOU HAVE PROBLEMS OR QUESTIONS?
If you ever have questions about this study or in case of research-related injuries, you should contact *[insert the name of the study doctor at your site]* at *[insert the telephone number]*. If you have questions about research subject's rights you can call *[insert the name and title of the appropriate country- or site-specific person]* at *[insert the telephone number]*.

SIGNATURE PAGE FOR CONSENT TO PARTICIPATE IN START MAIN STUDY

If you have read the informed consent (or if you have had it explained to you) and understand the information, and you voluntarily agree to join this study, please sign your name below.

____
Participant's name
(typed or printed)

____ ________
Participant's signature Date

OR

____
Participant's legal guardian or representative name
(typed or printed)

____ ________
Legal guardian/representative's signature Date

____
Witness's name
(typed or printed)

____ ________
Witness's signature Date

NOTE: This consent form with the original signatures MUST be retained on file by the principal investigator. A copy must be given to the participant. A copy should be placed in the participant's medical record.

A witness to the participant's signature is strongly encouraged.

> *Site Instruction:*
> *If your IRB/EC requires a separate consent document for specimen storage, do not use this signature page. Instead, use the START STUDY SPECIMEN STORAGE (PLASMA AND URINE) SAMPLE CONSENT (Appendix A-2).*

SIGNATURE PAGE FOR CONSENT TO STORE BLOOD AND URINE SPECIMENS

If you have read the information about stored specimens for future research in the informed consent (or if you have had it explained to you) and understand the information, please mark your choice in one of the boxes below and sign or initial as asked.

You can still be in the START study event if you do not want to have samples stored.

Please mark your choice:

☐ YES. I agree to have blood and urine samples collected and stored and used for future research. *Please sign below.*

☐ NO. Do not collect and store samples. *Please put your initials and today's date here →*

______________________________ *Do **NOT** sign below.*

____
Participant's name (typed or printed)

____ __________
Participant's signature Date

OR

____
Participant's legal guardian or representative name (typed or printed)

____ __________
Legal guardian/representative's signature Date

NOTE: This consent form with the original signatures MUST be retained on file by the principal investigator. A copy must be given to the participant. A copy should be placed in the participant's medical record.

START STUDY SPECIMEN STORAGE (PLASMA AND URINE) SAMPLE CONSENT

Site instruction:
Use this consent only if your IRB/IEC requires a separate consent document for specimen storage

University of Minnesota: SPONSOR
NIAID: PRIMARY FUNDER

Protocol Title:
Strategic Timing of AntiRetroviral Treatment
(START)

A Multicenter Study of the
International Network for Strategic Initiatives in Global HIV Trials
(INSIGHT)

Short Title of the Study: START

CONSENT FOR THE STORAGE OF SPECIMENS OBTAINED WHILE PARTICIPATING IN AN NIH-FUNDED RESEARCH TRIAL

SITE LEADER: ___________________________________ PHONE: __________

ALL SITE INSTRUCTION THAT IS INCLUDED IN A TEXT BOX SHOULD BE REMOVED FROM THE SITE'S INFORMED CONSENT FOR SUBJECTS

INTRODUCTION

You have decided to take part in START, a research study being funded primarily by the National Institute of Allergy and Infectious Diseases (NIAID), US National Institutes of Health (NIH), through a grant to the University of Minnesota, which is the lead institution in the INSIGHT group. The University of Minnesota is the sponsor of START. While you are in this research study, we would like to take some samples of blood and urine from you that might be useful for future research. You are being asked to agree to the storage of these samples and to their use for future research. This consent form gives you information about the collection, storage, and use of your samples. The study staff will talk with you about this information. Please ask if you have any questions.

If you agree to the storage of your samples for future research, you will be asked to sign this consent form. You will get a copy to keep. You do not have to agree to storing samples or sign this consent form in order to be in the main START study.

HOW WILL THE STUDY GET THE SAMPLES FROM YOU?
If you agree to allow the researchers to take additional samples for storage, you will have 15-30 mL (1 to 2 tablespoons) of blood drawn from a vein by a needle at baseline and each study follow-up visit (at 1 month, 4 months, and every 4 months after you start the study). You will also give a small urine sample at baseline and at 1, 4, 8, and 12 months, and every 12 months thereafter. You will give this sample by urinating into a container that your study doctor or nurse will give you. These additional samples will be kept and used for future research.

You will also be asked for a 10-mL (about 2 teaspoons) blood sample if you change HIV medicines because of the amount of HIV virus in your blood or because resistance mutations have been detected. This blood will be used sometime in the future to confirm whether your HIV virus had stopped responding to the HIV medicines you were taking. You and your doctor or nurse will not get the results of these tests.

HOW WILL YOUR SAMPLES BE USED?
Your samples will be used to learn more about HIV infection and its complications. The research may include studies to understand how HIV causes disease and complications and how to best treat or prevent HIV infection and its complications. Samples may also be used to study other problems that are very important to people with HIV infection, such as liver disease, diabetes, or heart disease. Testing may include studies of HIV, studies of other infections that affect people with HIV (for example, hepatitis viruses), studies of your cells, proteins, and other chemicals in your body.

The researchers do not plan to contact you or your doctor or nurse with any results from these studies done on your stored samples. This is because research tests are often done with experimental procedures, and, in general, results from only one research study should not be used to make a decision on how to treat your disease. Your samples will not be sold or used directly to produce commercial products. Research studies using your samples will be reviewed by the National Institutes of Health and a special committee at the institution where the researcher wants to test your samples (an Institutional Review Board or Ethics Committee).

WILL YOUR SAMPLES BE USED FOR STUDIES OF YOUR GENES (DNA)?
These samples will not be used to study your genes (DNA). Tests could possibly be done on the genes of the HIV virus you are infected with.

HOW LONG WILL YOUR SAMPLES BE KEPT?
There is no time limit on how long your samples will be stored.

HOW WILL YOUR SAMPLES BE STORED?
Your samples will be stored at special facilities in the United States that are designed to store samples safely and securely. The storage facilities are designed so that only researchers approved by the study team and the National Institutes of Health can use the samples for future testing. The employees at these facilities who will store and track your specimens will not have information that identifies you by name. An Institutional

Review Board (Ethics Committee) will oversee the storage facilities to protect you and other research participants from harm.

DOES STORAGE OF YOUR SAMPLES BENEFIT YOU?
There are no direct benefits to you. The benefit of doing research on stored samples includes learning more about HIV infection and its complications, in order to help people who have HIV.

WHAT ARE THE RISKS?
There are no risks to your health related to storing your samples. Possible risks of having blood drawn include pain, bleeding, bruising, lightheadedness, fainting, and rarely, infection or a blood clot where the needle enters the body. Possible risks to your privacy are described in the next section.

HOW WILL THE STUDY PROTECT YOUR PRIVACY?
In order to keep your information private, your samples will be labeled with a code that can be traced back only to your research clinic, not to you personally. Your personal information (name, address, phone number) will be protected by the research clinic. When researchers are given your stored samples to study, they will not be given your personal information. The results of future tests will not be included in your health records unless you are notified of a special test result and you ask that your test result be sent to another doctor or nurse. Every effort will be made to keep your personal information confidential.

Site Instruction:
If there are any other country/site-specific organizations or personnel that might have access to your subjects' research records, please add them to the above text.

For sites in the US only:
In addition to the efforts of the study staff to help keep your personal information private, we have gotten a Certificate of Confidentiality from the U.S. Federal Government. This certificate means that researchers cannot be forced to tell people who are not connected with this study, such as the court system, about your participation. However, this certificate has limited protection rights. You should know that it does not stop the doctor in charge of this study from taking appropriate steps to prevent serious harm to yourself or others.

WHAT ARE YOUR RIGHTS?
Allowing your samples to be stored is voluntary. You may decide not to have samples stored and still be in the START study. If you decide now that your samples can be stored for future research, you may change your mind at any time. You must contact your study doctor or nurse and let him/her know that you do not want your samples used for future research. Your samples will then no longer be used.

WHAT DO YOU DO IF YOU HAVE QUESTIONS?
For questions about the storage of your samples, contact *[insert the name of the investigator]* at *[insert telephone number]*. For questions about your rights related to the storage of your samples for research, contact *[insert the name or title of person on the Institutional Review Board]* at *[insert telephone number].*

SIGNATURE PAGE FOR CONSENT FOR STORAGE OF PLASMA AND URINE SPECIMENS OBTAINED WHILE PARTICIPATING IN START

If you have read the information about stored specimens for future research in this consent (or if you have had it explained to you) and understand the information, please mark your choice in one of the boxes below and sign or initial as asked.

You can still be in the START study event if you do not want to have samples stored.

Please mark your choice:

☐ YES. I agree to have blood and urine samples collected and stored and used for future research. *Please sign below.*

☐ NO. Do not collect and store samples. *Please put your initials and today's date here →*

____________________________ *Do **NOT** sign below.*

____
Participant's name (typed or printed)

____ _________
Participant's signature Date

OR

____
Participant's legal guardian or representative name (typed or printed)

____ _________
Legal guardian/representative's signature Date

Participant's legal guardian or representative name (typed or printed)

____ _________
Legal guardian/representative's signature Date

NOTE: This consent form with the original signatures MUST be retained on file by the principal investigator. A copy must be given to the participant. A copy should be placed in the participant's medical record.

START STUDY SPECIMEN STORAGE (BIOPSY TISSUE [*US sites only*: AND BLOOD]) AT DIAGNOSIS OF NEW MALIGNANCY SAMPLE CONSENT

University of Minnesota: SPONSOR
NIAID: PRIMARY FUNDER

Protocol Title:
Strategic Timing of AntiRetroviral Treatment
(START)

A Multicenter Study of the
International Network for Strategic Initiatives in Global HIV Trials
(INSIGHT)

Short Title of the Study: START

CONSENT FOR THE STORAGE OF BIOPSY TISSUE [*US sites only*: AND BLOOD] OBTAINED AT DIAGNOSIS OF NEW MALIGNANCY WHILE PARTICIPATING IN AN NIH-FUNDED RESEARCH TRIAL

SITE LEADER: ___________________________________ PHONE: __________

ALL SITE INSTRUCTION THAT IS INCLUDED IN A TEXT BOX *OR IN ITALICS* SHOULD BE REMOVED FROM THE SITE'S INFORMED CONSENT FOR SUBJECTS

INTRODUCTION

You have decided to take part in START, a research study being funded primarily by the National Institute of Allergy and Infectious Diseases (NIAID), U.S. National Institutes of Health (NIH), through a grant to the University of Minnesota, which is the lead institution in the INSIGHT group. The University of Minnesota is the sponsor of this study. If you develop a cancer or malignancy during the course of the START study, you are being asked now to agree to donate biopsy tissue [*US sites only:* and a blood sample] for future research. You are being asked to agree to the storage of these samples and to their use for future research. This consent form gives you information about the collection, storage, and use of your samples. The study staff will talk with you about this information. Please ask if you have any questions.

If you agree to the storage of your samples for future research, you will be asked to sign this consent form. You will get a copy to keep. You do not have to agree to storage of samples or sign this consent form in order to be in the main START study.

WHEN WILL YOUR TISSUE AND BLOOD BE OBTAINED?

If you have a tissue biopsy to determine if you have a cancer or malignancy we are asking for permission to have some of the tissue taken and stored for future research.

Only tissue in excess of that required for decision-making will be stored. If it is determined that your doctor needs more of your tissue for additional studies, the tissue that is stored may be given back to your doctor.

[*US sites only:* You are also being asked to donate a blood sample of approximately 20 mL (about 4 teaspoons) for storage so that scientists will also be able to look for any reasons that may explain the cancer. It is best to draw the blood sample before you have received treatment for your cancer or malignancy.]

If, during the course of treatment by your doctor, it is necessary to perform additional biopsy procedures for diagnostic reasons, you will be asked at that time to consent to having a portion of that sample sent for storage. [*US sites only:* No additional blood will be drawn.]

Information about you, such as your sex and age, that is not unique to you will be stored along with your sample. This information may help scientists better understand any tests they may do on your stored samples.

HOW WILL YOUR TISSUE AND BLOOD BE STORED?
Your samples will be stored at special facilities in the United States that are designed to store samples safely and securely. The name of these facilities is the AIDS Cancer Specimen Resource (ACSR). This bank has been set up by the National Cancer Institute (NCI) to store tissues and biological fluids from HIV-positive and HIV-negative persons. ACSR specimens are available for scientists studying cancers associated with HIV disease.

HOW WILL YOUR TISSUE AND BLOOD BE USED?
Your samples will be used to learn more about HIV infection, cancer, and other complications. The research may include studies to understand how HIV causes disease and complications and how to best treat or prevent HIV infection and its complications.

The researchers do not plan to contact you or your doctor or nurse with any results from studies done on your stored samples. This is because research tests are often done with experimental procedures, and, in general, results from only one research study should not be used to make a decision on how to treat your disease. Your samples will not be sold or used directly to produce commercial products.

Research studies using your samples will be reviewed by the National Cancer Institute. The studies will also be reviewed by a special committee (an Institutional Review Board or Ethics Committee) at the institution where the researcher wants to test your samples.

WILL YOUR SAMPLES BE USED FOR STUDIES OF YOUR GENES (DNA)?
Studies to detect certain genes associated with cancers or the development of cancers may be done on your stored specimens with appropriate approval by the

National Cancer Institute and the Institutional Review Board or Ethics Committee at the institution where the researcher wants to test your samples. The results of these studies will not identify you by name, but may be shared with other investigators and published in scientific journals.

HOW LONG WILL YOUR SAMPLES BE KEPT?
There is no time limit on how long your samples will be stored.

DOES STORAGE OF YOUR SAMPLES BENEFIT YOU?
It may be that there will be no direct benefit to you by participating in storing your tissue [*US sites only*: and blood samples]. However, there may be possible benefit to medical knowledge and it is hoped that the information gained from these procedures will help in the treatment of HIV-infected individuals in the future.

WHAT ARE THE RISKS?
There are no risks to your health in storing your samples. [*US sites only:* Possible risks of having blood drawn include pain, bleeding, bruising, lightheadedness, fainting, and rarely, infection or a blood clot where the needle enters the body.]

WHAT ARE THE COSTS?
There are no additional costs to you for storage of your tissue and blood samples.

HOW WILL THE STUDY PROTECT YOUR PRIVACY?
In order to keep your information private, your samples will be labeled with a code that can be traced back only to your research clinic, not to you personally. Your personal information (name, address, phone number) will be protected by the research clinic. When researchers are given your stored samples to study, they will not be given your personal information. The results of future tests will not be included in your health records. Every effort will be made to keep your personal information confidential.

Site Instruction:
If there are any other country/site-specific organizations or personnel that might have access to your subjects' research records, please add them to the above text.

For sites in the US only:
In addition to the efforts of the study staff to help keep your personal information private, we have gotten a Certificate of Confidentiality from the U.S. Federal Government. This certificate means that researchers cannot be forced to tell people who are not connected with this study, such as the court system, about your participation. However, this certificate has limited protection rights. You should know that it does not stop the doctor in charge of this study from taking appropriate steps to prevent serious harm to yourself or others.

WHAT ARE YOUR RIGHTS?

Allowing your samples to be stored is voluntary. You may decide not to have samples stored and still be in the START study. If you decide now that your samples can be stored for future research, you may change your mind at any time. You must contact your study doctor or nurse and let him/her know that you do not want your samples used for future research. Your samples will then no longer be used.

WHAT DO YOU DO IF YOU HAVE QUESTIONS?
For questions about the storage of your samples, contact *[insert the name of the investigator]* at *[insert telephone number]*. For questions about your rights related to the storage of your samples for research, contact *[insert the name or title of person on the Institutional Review Board]* at *[insert telephone number].*

SIGNATURE PAGE FOR CONSENT TO THE STORAGE OF BIOPSY TISSUE [*US sites only:* AND BLOOD] OBTAINED AT DIAGNOSIS OF NEW MALIGNANCY WHILE PARTICIPATING IN START

Site Instruction:<br>*This is only a suggested signature page. Sites may use their own signature page.*

Please read the statements below carefully, and think about your choice. Write your initials on the line in front of your choice for each statement. No matter what you decide, it will not affect your health care or your participation in the START study.

If, during the START study, I develop a new cancer or malignancy, I agree to donate a tissue sample from any biopsy that is taken [*US sites only:* and a blood sample (20 mL [4 teaspoons])] to be stored and to be used for future research related to HIV infection and its complications. I understand that I will not receive these results. I understand that this agreement may include tests of my genes (DNA).

_____Yes

_____No *Please write your initials and today's date here →* _____________ Do **NOT** sign this form.

If you have read this informed consent (or if you have had it explained to you) and understand the information, and you voluntarily agree to have biopsy tissue [*US sites only:* and blood] stored for this study, please print and sign your name below.

________________________<br>Participant's name<br>(typed or printed)	____________________________<br>Participant's signature	_________<br>Date
	**OR**	
________________________<br>Participant's legal<br>guardian or representative	____________________________<br>Legal guardian's signature	_________<br>Date

________________________ ____________________________ _________

Witness's name (typed or printed) — Witness's signature — Date

NOTE: This consent form with the original signatures MUST be retained on file by the site investigator. A copy must be given to the participant. A copy should be placed in the participant's medical record, if applicable.

A witness to the participant's signature is strongly encouraged.

START GENOMICS SUBSTUDY SAMPLE CONSENT

University of Minnesota: SPONSOR
NIAID: PRIMARY FUNDER

Protocol Title:
Genomics: A Substudy of

Strategic Timing of AntiRetroviral Treatment
(START)

A Multicenter Study of the
International Network for Strategic Initiatives in Global HIV Trials
(INSIGHT)

Short Title of the Study: START Genomics Substudy

CONSENT FOR PARTICIPATION IN A SUBSTUDY OF AN NIH-FUNDED RESEARCH TRIAL

SITE LEADER: ___________________________________ PHONE: __________

ALL SITE INSTRUCTION THAT IS INCLUDED IN A TEXT BOX SHOULD BE REMOVED FROM THE SITE'S INFORMED CONSENT FOR SUBJECTS

INTRODUCTION

You are being asked to take part in this substudy because you are infected with HIV, the virus that causes AIDS, and because you have joined the START study. The START study is being funded primarily by the National Institute of Allergy and Infectious Diseases (NIAID), U.S. National Institutes of Health (NIH), through a grant to the University of Minnesota, which is the lead institution in the INSIGHT group. The University of Minnesota is the sponsor of this study. This substudy is being done to look at the genetics of people with HIV and of the specific HIV virus infecting them, to see how these might affect the health or treatment of people with HIV.

YOUR PARTICIPATION IS VOLUNTARY

This consent form gives you information about the substudy that will be discussed with you. Once you understand the substudy, and if you agree to take part, you will be asked to sign this consent form. You will be given a copy to keep.

Before you learn about the substudy, it is important that you know the following:

- Your participation is entirely voluntary;
- You can refuse to take part in this substudy and still be in the main START study;
- If you agree to this substudy and then decide to stop participating in the substudy, you may do so at any time for any reason. If you stop participating in this substudy,

you can still be in the main START study, and you will not lose any of the benefits of your regular medical care.

WHY DO A GENOMICS SUBSTUDY IN START?

Research from other medical problems like diabetes, high blood pressure, and hepatitis C infection has shown that people may react differently to either illness or treatment, based on their genes. Genes are inherited and control things like hair color and height. Everyone's genes are a little different. The purpose of this substudy is to collect one blood specimen and store it for researchers who will do genetic testing (testing on your genes) and other related tests in the future. These tests, when linked with your health information from the START study, will help us find out how the genetic make-up of people affects HIV infection and its treatment. For example, some people who carry a specific version of a gene may have serious side effects after taking abacavir (Ziagen®) while others who have a different version of the gene will probably not have that side effect. If your doctor or nurse wants to prescribe abacavir for you, you will have a test for this gene done as part of being in the START study, since we know that this can make a difference in what HIV medicines your study doctor or nurse prescribes to you.

By studying your DNA or genes, this substudy may help scientists learn more about people's ability to fight off HIV infection and its complications. It may also help them learn about how people respond differently to HIV treatment, to find out why some people get different illnesses and side effects of medicines, and how to best treat HIV and other HIV-related conditions. Any future research done on the blood collected for this substudy will be related to HIV disease, the treatment of HIV, or the infections and other health problems common to people who are infected with HIV.

HOW MANY PEOPLE WILL TAKE PART IN THE GENOMICS SUBSTUDY?

Any person enrolled in START may take part in this substudy. We expect about 4,000 people from around the world to enroll in START.

HOW LONG WILL YOU BE IN THE SUBSTUDY?

Your participation in this substudy will end when your blood specimen arrives at the storage facility in usable condition. If it does not arrive in usable condition, we will ask you to sign another consent form and donate another blood specimen.

Your blood will be used for research to be done in the future. If you later decide that you do not want your blood to be used for future research, it will not be used, and every effort will be made to destroy it. Test results from the blood will not be given to you in any case.

Your participation in the START study will continue until the START study ends or you withdraw your consent to be in it. Any health information from the START study linked to your blood specimen for this substudy will only be used for research.

WHAT DO YOU HAVE TO DO IF YOU ARE IN THE GENOMICS SUBSTUDY?

The research staff will collect one blood specimen of about 9 mL (a little less than 2 teaspoons). This will be done by using a needle to get blood from a vein in your arm. This procedure will only take a few minutes of your time. This will happen only one time.

HOW WILL YOUR BLOOD BE USED?
Your blood will be used only to learn more about HIV infection and about health problems common to people who are infected with HIV. This may include tests to better understand why some people with HIV get sicker faster than others and why medicines might work better in one group of HIV-infected people compared with other groups.

Researchers involved with this blood collection project do not know yet exactly which tests will be done.

You and your study doctor or nurse will not get any results from the tests done on your blood collected in this substudy. These tests will only be used for research and may not apply to your clinical care. If these tests show a promising result that has a strong and clear effect on the health condition of other research participants, every reasonable effort will be made to provide you with additional genetic testing outside of this substudy.

Your blood sample collected for this substudy will become the property of the START study. Your blood will not be sold or used to make commercial products. It will not be tested for any specific research study unless the plan for using your blood is approved by the INSIGHT Scientific Steering Committee, the DAIDS (National Institutes of Health/NIH), and a special committee at the researcher's institution (an Institutional Review Board or Ethics Committee).

Researchers will write reports about new findings and results that they learn about from doing future tests on your blood. These reports will be shared with participating sites. These findings will also be submitted for publication in scientific or medical journals to share with you and the public. Any publications about this research will not use your name or identify you personally.

HOW LONG WILL YOUR BLOOD BE KEPT?
Your blood specimen will be stored as long as funding is available for storage and testing.

HOW WILL YOUR BLOOD BE STORED?
Your blood specimen will be stored safely and securely at a special facility in the United States called a specimen repository. This facility follows strict procedures so that only approved researchers can use the stored specimen for future testing. The employees at this facility who will store and track your blood specimen will not have information that identifies you by name.

[Alternative to Previous Paragraph for International Sites Only]

Your blood specimen will be stored safely and securely at a special facility called a specimen repository. The repository may be located within the United States. This facility follows strict procedures so that only approved researchers can use the stored specimen for future testing. The employees at this facility who will store and track your blood specimen will not have information that identifies you by name.

HOW IS YOUR PRIVACY PROTECTED?
Researchers will take every reasonable step to protect the confidentiality of your health information and to prevent misuse of this information. They will also make sure your blood sample is handled with care at the storage facility and that your privacy is protected. For example, your research records will be identified by a code. Your blood sample and results of any genetic testing will be identified by a second code. Only a few statisticians associated with the START study will have access to both codes in order to analyze the test results. These statisticians will not have access to any information that can identify you.

[For U.S. Sites Only]
In addition to these efforts to keep your information confidential, the START study is covered by a Certificate of Confidentiality from the U.S. Department of Health and Human Services. This certificate means that researchers cannot be forced to give information collected as part of this substudy to people who are not involved with the substudy, such as the court system. However, this certificate has limited protection rights. You should know that it does not stop the doctor in charge of this substudy from taking appropriate steps to prevent serious harm to yourself or others. Federal and state laws also help protect research participants and others who have genetic testing done.

[For International Sites Only]
Efforts will be made to keep your personal information confidential, but we cannot guarantee complete confidentiality. Your personal information may be released if required by law. Any publication of this substudy will not use your name or identify you personally.

[For All Sites]
Your medical and research records may be reviewed by the *[insert the name of the site]* ethics committee (institutional review board, IRB), the U.S. National Institutes of Health (NIH), the U.S. Office for Human Research Protections (OHRP), and the research staff and monitors, and their designees. Also, the research staff at *[insert the name of the site]* is required to make sure that people not involved with this substudy do *not* have access to your research and medical records while collecting personal information about you. They will keep your files in a locked cabinet in a safe place and will handle your personal information very carefully. This will also help to protect your privacy.

WHAT ARE THE BENEFITS OF THE GENOMICS SUBSTUDY?
There are no benefits to you for participating in this substudy. Information learned from the blood collected as a part of this substudy may help others with HIV in the future.

WHAT ARE THE RISKS OF THE SUBSTUDY?
There are few risks involved with your participation in this project. Drawing blood may result in a little pain and slight bruising where the needle goes into your skin. You may also faint, feel lightheaded, bleed, or develop a small blood clot where the needle goes into your skin. Very rarely, your skin may get infected. Another small but unlikely risk is the possibility of others finding out about your participation in this substudy.

CAN YOUR GENOMICS SUBSTUDY PARTICIPATION BE STOPPED WITHOUT YOUR CONSENT?
Your study doctor or nurse may need to take you off the substudy early without your permission if the substudy is cancelled by the sponsor (the University of Minnesota), the National Institute of Allergy and Infectious Diseases (NIAID), regulatory authorities in your country, or your site's Institutional Review Board (IRB)/Ethics Committee(IEC);

WHAT OTHER CHOICES DO YOU HAVE BESIDES THIS SUBSTUDY?
You can choose not to be in this substudy and still be in the START study. Please talk to your study doctor or nurse about this and other choices available to you.

WHAT IF YOU ARE INJURED?
If you are injured from having your blood drawn for this substudy, you will receive proper medical care. The cost for such medical care will be paid by you or by another party. There is no program for compensation through this substudy. You will not be giving up any of your legal rights by signing this consent form.

Site Instruction:
If the information is not correct for your country/site, please revise the information to inform the subject of the following: 1. what treatment will be provided to the subject; 2. who will pay for the treatment; 3. if there is any plan for compensation for research-related injury issues, such as lost wages, etc.

WHAT ARE THE COSTS TO YOU?
There is no cost to you to be in this substudy. The substudy will cover all costs for storage of your blood and for future tests.

WHAT ARE YOUR RIGHTS AS A RESEARCH PARTICIPANT?
Allowing your blood to be collected, stored, and tested at a later date is voluntary. Your decision will not affect your right to take part in the main START study or affect your receipt of medical care.

If you sign the consent that your blood can be stored for research to be done at a later date, including studies of your genes, you can change your mind at any time. If you change your mind, you must write a letter to *[insert the name of the principal investigator*] at the *[insert the name and address of the site]* to let them know that you

do not want your blood specimen used for future research. A sample letter will be given to you as a guide to help you express your request in writing.

When *[insert the name of the principal investigator]* receives your letter, the research staff will contact you to come to the clinic to sign and date in this original informed consent to verify your decision. A second copy of this consent will be given to you as proof that we received your request. If we do not hear from you within 30 days after getting your letter to withdraw from this plan, we will send your request to the storage facility.

If you decide to withdraw consent for this substudy, your blood sample and any parts separated from it will not be used. Every effort will be made to destroy your blood sample and any parts separated from it. If some testing has already been done on your blood sample, the results from this testing will remain as part of this research. The research staff at the *[insert the name of the site]* will notify you of the date your blood specimen and any of its parts were destroyed. You have the right to change your mind and to re-enroll in this substudy. If you change your mind, you must sign a new consent form and donate another blood specimen.

WHOM DO YOU CALL IF YOU HAVE QUESTIONS OR PROBLEMS?
For questions about this substudy or about the storage or use of your blood, and in case of any research-related injury, contact:
- *[name of the investigator or other study staff]*
- *[telephone number of above]*

For questions about your rights as a research participant contact:
- *[name or title of person on the ethics committee (Institutional Review Board, IRB) or other organization appropriate for the site]*
- *[telephone number of above]*

SIGNATURE PAGE FOR START <u>GENOMICS SUBSTUDY CONSENT</u>

Site Instruction:<br>*This is only a suggested signature page. Sites may use their own signature page.*

You agree to donate a blood sample for the START Genomics Substudy to be stored and used for future genetic research related to HIV infection, its complications, and related illnesses. You understand that the testing and research on your blood sample will be done at a later date. You also understand that you and your study doctor or nurse will not get results from any testing done on your blood sample. Further, you understand that your blood sample may be stored for a long time.

Unless you decide at some point in the future to submit a request in writing to no longer take part in this substudy, you agree to let the START study researchers use your blood sample for approved HIV-related genetic testing and research whether or not you are still alive. You also agree to give permission/authorization for the use and disclosure of your personal health information as described in this consent form for the purposes of this research.

If you have read this consent form (or had it explained to you) and all of your questions were answered, and you agree to take part in this substudy, please print and sign your name, and give the date of your signature below. By doing so, you also confirm receiving the *sample study withdrawal letter*.

______________________<br>Participant's name<br>(typed or printed)	______________________<br>Participant's signature	__________<br>Date
	**OR**	
______________________<br>Participant's legal<br>guardian or representative	______________________<br>Legal guardian's signature	__________<br>Date

______________________<br>Witness's name<br>(typed or printed)	______________________<br>Witness's signature	__________<br>Date

NOTE: This consent form with the original signatures MUST be retained on file by the site investigator. A copy must be given to the participant. A copy should be placed in the participant's medical record, if applicable.

A witness to the participant's signature is strongly encouraged.

START GENOMICS SUBSTUDY SAMPLE WITHDRAWAL LETTER

SAMPLE STUDY WITHDRAWAL LETTER
FOR STUDY PARTICIPANTS WHO CHOOSE AT A LATER DATE
TO STOP TAKING PART IN THE START GENOMICS SUBSTUDY AND
NOT TO HAVE THEIR BLOOD SPECIMEN USED FOR FUTURE GENETIC TESTING

NOTE FOR THE STUDY PARTICIPANT: PLEASE KEEP A COPY OF THIS LETTER FOR YOUR RECORDS ALONG WITH A COPY OF YOUR SIGNED CONSENT FORM.

Date of Letter

Name of Site Leader at the Participating Site
Name of Facility
Name of Department
Address (Street Number and Name)
Address (City, State, Country, Postal Code)

Dear Dr. (Name of Site Leader):

I am a study participant for the START Genomics Substudy. I have decided to stop participating in this research. Therefore, I would like to have my blood sample destroyed and not used for future genetic testing.

I understand that any information collected from testing my blood sample before you received this request will remain a part of the research study and kept confidential. I also understand that if I decide to be a part of this study again, I must sign a new consent form and donate another blood sample.

Please let me know when my blood sample is destroyed.

Thank you for respecting my decision.

Cordially,

Your signature

START NEUROLOGY SUBSTUDY SAMPLE CONSENT

University of Minnesota: SPONSOR
NIAID: PRIMARY FUNDER

Protocol Title:
Neurology: A Substudy of
Strategic Timing of AntiRetroviral Treatment
(START)

A Multicenter Study of the
International Network for Strategic Initiatives in Global HIV Trials
(INSIGHT)

Short Title of the Study: START Neurology Substudy

CONSENT FOR PARTICIPATION IN A SUBSTUDY OF AN NIH-FUNDED RESEARCH TRIAL

SITE LEADER: ___________________________________ PHONE: __________

ALL SITE INSTRUCTION THAT IS INCLUDED IN A TEXT BOX SHOULD BE REMOVED FROM THE SITE'S INFORMED CONSENT FOR SUBJECTS

INTRODUCTION

You are being asked to take part in this substudy because you are infected with HIV, the virus that causes AIDS, and because you have joined the START study. This substudy is being done to look at whether there are changes in the function of the brain depending on whether you start HIV medicines early or wait until the guidelines suggest doing so.

YOUR PARTICIPATION IS VOLUNTARY

This consent form gives you information about the study that will be discussed with you. Once you understand the study, and if you agree to take part, you will be asked to sign this consent form. You will be given a copy to keep.

Before you learn about this substudy, it is important that you know:

- Your participation is completely voluntary;
- You can refuse to take part in this substudy and still be in the main START study;
- If you agree to this substudy and then decide to stop participating in the substudy, you may do so at any time for any reason. If you stop participating in this substudy, you can still be in the main START study, and you will not lose any of the benefits of your regular medical care.

If you agree to take part in this substudy, you will be asked to sign this consent form. The doctor in charge of this substudy at this location is *[insert the name of the Site Leader]*. He or she will keep the original copy of this consent to place in your medical record. You will also receive a copy to keep.

This study is being funded by DAIDS, National Institute of Allergy and Infectious Diseases (NIAID); the National Institute of Neurological Disorders and Strokes (NINDS); and the National Institute of Mental Health (NIMH), all in the U.S. National Institutes of Health (NIH), through a grant to the University of Minnesota, which is the lead institution in the INSIGHT group. The University of Minnesota is the sponsor of this study.

WHY IS THE NEUROLOGY SUBSTUDY BEING DONE?
HIV can affect how well the brain functions and may cause problems like poor concentration and memory: this is called "HIV-related cognitive impairment." HIV-related cognitive impairment usually only happens when a person's CD4+ count is less than 200 cells/mm^3, and HIV medicines have been proven to help to reverse memory and concentration problems in this situation.

When CD4+ counts are much higher, like over 500 cells/mm^3, HIV may cause changes in brain function in some people that are so small that the people don't even notice them. We don't know whether starting HIV medication early helps to treat or prevent these small changes; it may be that it's perfectly safe to wait until your CD4+ cells drop to the point at which most guidelines recommend that you start treatment (350 cells/mm^3 or fewer). There is very little information to help answer this question. Therefore this substudy will look at whether starting HIV medicines earlier or waiting until the CD4+ cell count drops below 350 cells/mm^3 makes any difference to brain function in people with HIV.

HOW MANY PEOPLE WILL TAKE PART IN THE NEUROLOGY SUBSTUDY?
We expect that we will need about 600 people to answer this question.

HOW LONG WILL YOU BE IN THE SUBSTUDY?
You will continue to be followed in the substudy until the main START study comes to an end. Right now we think it will take about 6 years to complete the study.

WHAT DO YOU HAVE TO DO IF YOU ARE IN THIS SUBSTUDY?

Screening
After you consent to the main START study and this substudy, your study doctor or nurse will do eight tests that measure your memory, speed of thinking, concentration, movement, and coordination. You will be asked to fill out a short questionnaire about any depression you may be having. The tests and questionnaire will take about an hour for you to complete.

The tests include:
- Placing pegs in holes on a board – this tests your speed of thinking and coordination

- Connecting colored circles on a piece of paper (two versions) – these test your movement and coordination, your speed of thinking, and your concentration
- Tapping your index fingers – this tests your movement and coordination
- Matching symbols and numbers – this tests your movement and coordination, your speed of thinking, and your concentration
- Remembering a list of words that will be read out loud to you – this tests your concentration, your memory, and your speed of thinking
- To list words of the same kind, for example, words describing food – this tests your speed of thinking, and your concentration

At this first visit only, you will also be asked about how many years of schooling you have had, whether you live in a town or a rural area, and about your occupation and income level.

Follow-up visits
You will return to the clinic for study visits at months 4, 8, and 12, and every 12 months after that. At each of these visits, your doctor or study nurse will do exactly the same tests that were done at your first visit, as described above. You will be asked to fill out the same short questionnaire about any depression you may be having. At each study visit, the tests and questionnaire will take about an hour for you to complete.

WILL YOU GET THE RESULTS OF THE TESTS DONE IN THIS SUBSTUDY?
The results of the tests done in this substudy will not be available to you. This is because these tests are never used alone to diagnose HIV-related cognitive impairment or other brain disorders. Your study doctor or nurse will explain and recommend available treatment options, should you have or develop serious brain problems.

Your study doctor or nurse will go over your responses to the depression questionnaire to make sure you are getting appropriate care if you are having any depression.

WHAT ARE THE RISKS AND/OR DISCOMFORTS OF BEING IN THIS SUBSTUDY?
You may become tired during the tests of brain function. If this happens, it's fine for you to take a break for a while and then continue.

WHAT ARE THE BENEFITS OF BEING IN THIS SUBSTUDY?
If you take part in this substudy, there may be a direct benefit to you from having your brain function looked at regularly, but no guarantee can be made. It is also possible that you may receive no benefit from being in this study. Information learned from this study may help others who have HIV.

WHAT ABOUT PREGNANCY AND BREASTFEEDING?
There are no risks related to pregnancy or breastfeeding.

WHAT IF THERE ARE NEW FINDINGS?
You will be told about any new information learned during this substudy that might cause you to change your mind about staying in it. At the end of the main START

study, you will be told when substudy results may be available and how to learn about them.

WHAT IF YOU WANT TO WITHDRAW FROM THE NEUROLOGY SUBSTUDY?
If you enroll in this substudy, you may decide to stop participating at any time. Withdrawing from this study will not affect your medical care, and you can continue to be in the main START study.

CAN YOUR SUBSTUDY PARTICIPATION BE STOPPED WITHOUT YOUR CONSENT?
You may be taken off the Neurology substudy without your consent if:

- Your study doctor decides that continuing in the substudy would harm you;
- The substudy is cancelled by the sponsor (the University of Minnesota), the National Institute of Allergy and Infectious Diseases (NIAID), regulatory authorities in your country, or your site's Institutional Review Board (IRB)/Ethics Committee(IEC);
- You are in jail or prison
- Other administrative reasons.

WHAT ARE THE ALTERNATIVES TO BEING IN THIS SUBSTUDY?
You can choose not to be in this substudy. Please talk to your study doctor or nurse about this and other choices available to you.

ARE THERE ANY COSTS TO YOU?
There is no cost to you to be in this substudy. The substudy will cover all costs for doing the tests described earlier in the consent.

WHAT IF YOU ARE INJURED?
There is very little chance that you would be injured by participating in this substudy. If you are injured as a result of participating in this substudy, you will receive proper medical care. The cost for such medical care will be paid by you or by another party. There is no program to compensate you through this substudy. You will not be giving up any of your legal rights by signing this consent form.

Site Instruction:<br>*If the information is not correct for your country/site, please revise the information to inform the subject of the following: 1. what treatment will be provided to the subject; 2. who will pay for the treatment; 3. if there is any plan for compensation for research-related injury issues, such as lost wages, etc.*

HOW IS YOUR PRIVACY PROTECTED?
Researchers will take every reasonable step to protect the privacy of your health information and to prevent misuse of this information. For example, your research records will be identified by a code. You will not be identified by name or any other way in any publication about this substudy.

[The following paragraph is for U.S. sites only]

In addition to these efforts to keep your information private, the START study and its substudies are covered by a Certificate of Confidentiality from the U.S. Department of Health and Human Services. This certificate means that researchers cannot be forced to give information collected as part of this substudy to people who are not involved with the substudy, such as the court system. However, this certificate has limited protection rights. You should know that it does not stop the doctor in charge of this substudy from taking appropriate steps to prevent serious harm to yourself or others.

[The following paragraph is for international sites only]
Efforts will be made to keep your personal information private, but we cannot guarantee complete confidentiality. Your personal information may be released if required by law. Any publication of this substudy will not use your name or identify you personally.

[The following paragraph is for all sites}
Your medical and research records may be reviewed by the *[insert the name of the site]* ethics committee (institutional review board, IRB), the U.S. National Institutes of Health (NIH), the U.S. Office for Human Research Protections (OHRP), and the research staff and monitors, and their designees. Also, the research staff at *[insert the name of the site]* is required to make sure that people not involved with this substudy do not have access to your research and medical records while collecting personal information about you. They will keep your files in a locked cabinet in a safe place and will handle your personal information very carefully. This will also help to protect your privacy.

WHOM DO YOU CALL IF YOU HAVE QUESTIONS OR PROBLEMS?
For questions about the Neurology substudy or in case of a research-related injury contact:

- *insert name of the investigator or other study staff*
- *insert telephone number of above*

For questions about your rights as a research participant, contact:

- *insert name or title of person on the ethics committee (Institutional Review Board, IRB) or other organization appropriate for the site*
- *insert telephone number of above*

SIGNATURE PAGE FOR THE START NEUROLOGY SUBSTUDY CONSENT

Site Instruction:<br>*This is only a suggested signature page. Sites may use their own signature page.*

You have already agreed to join the main START study, and you can still be in the main START study even if you do not want to join this substudy.

If you have read this informed consent (or if you have had it explained to you) and understand the information, and you voluntarily agree to join ***the START Neurology Substudy***, please sign your name below.

Participant's name (typed or printed)	Participant's signature	Date
	**OR**	
Participant's legal guardian or representative	Legal guardian's signature	Date

Witness's name (typed or printed)	Witness's signature	Date

NOTE: This consent form with the original signatures MUST be retained on file by the principal investigator. A copy must be given to the participant. A copy should be placed in the participant's medical record, if applicable.

A witness to the participant's signature is strongly encouraged.

START INFORMED CONSENT SUBSTUDY: SAMPLE CONCISE CONSENT FOR MAIN STUDY

University of Minnesota: SPONSOR
NIAID: PRIMARY FUNDER

Protocol Title:
Strategic Timing of Anti-Retroviral Treatment
(START)

A Multicenter Study of the
International Network for Strategic Initiatives in Global HIV Trials
(INSIGHT)

Short Title of the Study: START

CONSENT FOR PARTICIPATION IN AN NIH-FUNDED RESEARCH TRIAL

SITE LEADER: ___________________________________ PHONE: __________

ALL SITE INSTRUCTION THAT IS INCLUDED IN A TEXT BOX SHOULD BE REMOVED FROM THE SITE'S INFORMED CONSENT FOR SUBJECTS

OHRP Requirements to be read by the sites:

PLEASE NOTE THAT THIS SAMPLE LANGUAGE DOES NOT PREEMPT OR REPLACE LOCAL IRB REVIEW AND APPROVAL. INVESTIGATORS ARE REQUIRED TO PROVIDE THE LOCAL IRB WITH A COPY OF THIS SAMPLE LANGUAGE ALONG WITH THE LANGUAGE INTENDED FOR LOCAL USE. LOCAL IRBS ARE REQUIRED TO WEIGH THE UNIQUE RISKS, CONSTRAINTS, AND POPULATION CONSIDERATIONS AS A CONDITION OF ANY APPROVAL. ANY DELETION OR SUBSTANTIVE CHANGE OF INFORMATION CONCERNING RISKS OR ALTERNATIVE TREATMENT MUST BE JUSTIFIED BY THE INVESTIGATOR, APPROVED BY THE LOCAL IRB, AND NOTED IN THE IRB MINUTES. JUSTIFICATION AND IRB APPROVAL OF SUCH CHANGES MUST BE FORWARDED TO THE INTERNATIONAL COORDINATING CENTER. SPONSOR-APPROVED CHANGES IN THE PROTOCOL MUST BE APPROVED BY THE LOCAL IRB BEFORE USE UNLESS INTENDED FOR THE ELIMINATION OF APPARENT IMMEDIATE HAZARD. NEW INFORMATION SHALL BE SHARED WITH EXISTING SUBJECTS AT NEXT ENCOUNTER, WITH ALL NEW SUBJECTS PRIOR TO INVOLVEMENT, OR AS THE LOCAL IRB MAY OTHERWISE ADDITIONALLY REQUIRE.

Site instruction:
If your site is participating in the Informed Consent Substudy, please work with your IRB to modify the consent language as little as possible from this template. This will strengthen the validity of the comparison of the two consents.

We invite you to join this HIV/AIDS research study. It is up to you whether or not you want to join this study. Please ask questions and take as much time as you need to decide.

WHY ARE WE DOING THIS RESEARCH?

We want to find out whether it is better for people with HIV infection to start taking HIV medicines as soon as they know they have HIV or to wait until their CD4 cell count is less than 350 cells/mm^3. CD4 cells are immune cells that normally fight infection, but fall over time in HIV infection.

Randomized trials have shown that people with *less* than 350 CD4 cells/mm^3 or with HIV symptoms benefit from taking HIV medicines. It is less certain whether someone with *more* than 350 CD4 cells/mm^3 should start HIV medicine and that is why we are doing this study. US guidelines from December 2009 recommend starting HIV medicines in people with more than 350 CD4 cells/mm^3. This recommendation is based on observational information and not on a randomized clinical trial. A randomized trial gives the most certain information about how well a treatment works because randomization makes sure each group is similar except for the treatment they receive.

Our study is a randomized trial. People like you who are HIV infected but have not taken HIV medicine will be randomized – like flipping a coin – into one of two groups. If you join, you have an equal chance of being in either group:

- The **EARLY** group (half of those who join the study) will start taking HIV medicines right away.

- The **DEFERRED** group (half of those who join the study) will be watched very closely but will wait to start HIV medicines until their CD4 cells fall below 350 cells/mm^3 or HIV symptoms or AIDS are seen.

Whether you are in the DEFERRED or EARLY group, you and your doctor will decide which specific HIV medicines to take from a list of standard HIV medicines.

The University of Minnesota, the sponsor of this study, gets royalties (payments) from the use of abacavir, one of the HIV medicines that can be used in this study. The sponsor will not tell you or your doctor if you should take abacavir. Only you and your doctor will decide which HIV medicines you will take.

We will also study whether the cost of medical care, general health, and satisfaction with life differs between the DEFERRED and EARLY group. And, we will study whether the virus changes and becomes resistant to some HIV medicines. We are testing two different forms of written information to find out which is easier to understand. You will get one of these written forms.

We plan to enroll 4000 people with HIV infection and follow them for 3 to 6 years.

WHAT WILL HAPPEN DURING THE RESEARCH?

Before the study begins
During 2 clinic visits, we will do some tests to see if you qualify:

- Visit 1: Draw a small amount of blood to measure how many CD4 cells you have.

- Visit 2: (at least 2 weeks later)
    - Ask questions about your health, medical history, sexual behaviors, use of alcohol and drugs, and quality of life.
    - Check to see how healthy you are by doing a physical examination, taking a small amount of blood and urine, *and doing a routine test of your heart (an ECG)*. You should not eat or drink anything except water for 8 hours before this clinic visit. We will give you the results of these tests when they are ready.
    - If you are a woman, test your blood or urine to see if you are pregnant.
    - Optionally, take a small amount of blood and urine to store for later tests on HIV and health problems associated with HIV. You will be asked to sign to indicate whether you agree to this.

Site instruction:
If your site does not have or will not be receiving a study-supplied ECG machine and a certified technician, please remove the text "and doing a routing test of your heart (an ECG)" in the second bullet of the preceding list.

After the study starts
Whether you are in the DEFERRED or EARLY group, you will come to the clinic 1 and 4 months after you start the study and then about every 4 months until the study ends. Each time we will ask questions about your health, medicines, and side effects.

At the beginning of the Study	At 1 and 4 months, and every 4 months after	Additional tests done once a year
• *Optional* Consent Questionnaire<br>• Assigned to either the **DEFERRED** or the **EARLY** group.	• Blood tests for HIV viral load and CD4 count<br>• Physical exam<br>• *Optional* blood to store for future research<br>• Urine test for general health and *optional* urine to store for future research (once a year after the first year)	• Blood tests to check general health<br>• ECG of your heart<br>• Health-related questionnaires (also at month 4)

Site instruction:
If your site does not have or will not be receiving a study-supplied ECG machine and a certified technician, please remove "ECG of your heart" from the preceding table.

At any time during the study - Please talk to your study doctor or nurse *as soon as possible if*:

- *You are sick or hurt or in the hospital for any reason.*
- *You want to join any other research study*
- *You take any medicines, including over-the-counter, herbal, or alternative medicines.*
- *You move or transfer your care to another doctor.*
- *You become pregnant*.

With your permission, we will contact your doctor(s) to ask about any medicines you are taking or any illnesses, hospitalizations, or pregnancies you have had.

FOR WOMEN: PREGNANCY AND BREASTFEEDING

You cannot join if you are pregnant or breastfeeding. We will test to see if you are pregnant. To be in the study you must be willing to use appropriate birth control when you are taking HIV medicines. Please discuss this with your doctor. If you do become pregnant during the study, please tell the study doctor or nurse *right away* because your HIV medicines may need to be changed. We will ask you to continue your study visits.

WHAT ARE THE RISKS OF BEING IN THIS STUDY?

All HIV medicines have some side effects. No one knows whether people in the DEFERRED or EARLY group will have fewer risks overall. There may also be risks that we do not know about now. We will tell you if we learn about new risks or any other information that might be important to you.

Possible risks of being in the DEFERRED group:

- A drop in CD4 cell count that could increase the chance of developing HIV symptoms or AIDS.
- An increased chance of infecting others with HIV because of virus in your blood.

Possible risks of being in the EARLY group:

- More side effects than people who take medicines for a shorter time.
- Difficulty sticking to a schedule when taking medicines for a long time.
- Increased chance that the HIV virus will become resistant to the HIV medicines you are on.

Other possible study risks are:

- Side effects because of an interaction of HIV medicines with other medicines you might be taking, including herbal or alternative medicines.
- Pain, bleeding, bruising, feeling lightheaded, anxious, or in rare cases fainting or an infection or blood clot when blood is drawn. Some people feel anxious while waiting for test results.
- Discomfort from some of the questions we ask you.

WHAT ARE THE BENEFITS OF BEING IN THIS STUDY?
Although taking HIV medicines can help people with HIV infection, we do not know whether you will benefit more from starting HIV medicines EARLY or taking them at the DEFERRED time**.** This study will help us learn when to treat future patients with HIV.

WHAT CHOICES DO YOU HAVE OTHER THAN BEING IN THIS STUDY?
You do not have to join this research study if you do not want to. If you join, you can quit at any time. If you choose not to join or to quit, it will not affect your regular medical care. If you decide not to join, please talk with your doctor about whether or not to take HIV medicines.

CAN YOUR STUDY PARTICIPATION BE STOPPED EVEN IF YOU DON'T AGREE?
The study doctor can take you out of this study if continuing in the study would harm you, if you go to prison, or if the study is stopped by the study funder, sponsor, review committees (IRB/REC) or government authorities, or for other administrative reasons. Once a year, an expert group will review the study and will recommend stopping it if the risk is higher than expected in either the DEFERRED or EARLY group.

WHAT WILL WE PAY FOR?
We will pay for the services, lab work, and other tests that are part of this study but not part of your regular care. During the study, your HIV medicines will either be paid for through your insurance company or national program or the study will provide them.

WHAT WILL HAPPEN AT THE END OF THE STUDY?
If you stop participating or the study ends, you and your doctor will decide whether you should take HIV medicines. If you stop early, the study will not be able to continue to provide your medicines. If you finish the study and we are providing your HIV medicines, we will continue to provide them for up to 6 months while you and your doctor find another way to get your medicines.

WHO WILL BE ABLE TO SEE YOUR MEDICAL INFORMATION?
We will protect the privacy of your medical information as much as legally possible, and release your records only with your written permission. We will label your study records with a code number and three letters, and you will not be identified in any publications about this research. However, your records may be seen by:

- People in the US government agencies that fund or oversee this research, for example, the U.S. National Institutes of Health (NIH).
- Study monitors who make sure the study is being conducted correctly.
- Independent groups (IRBs) that make sure the study is ethically acceptable.

[Include the following paragraph at US sites only]
"We have a Certificate of Confidentiality from the US Government. This means that law enforcement officers, the courts, and others cannot force us to give them information about you. However, this does not prevent the study team from taking appropriate steps to prevent serious harm to you or to others.

WHAT IF YOU ARE INJURED AS PART OF THE STUDY?
We will provide treatment right away if you are hurt because of the research. The costs may be charged to you or your insurance company. We will give you information about where you can get additional treatment. You do not give up any of your legal rights by signing this form.

Site Instruction:
If the information is not correct for your country/site, please revise the information to inform the subject of the following: 1. what treatment will be provided to the subject; 2. who will pay for the treatment; 3. if there is any plan for compensation for research-related injury issues, such as lost wages, etc.

WHO CAN YOU TALK TO ABOUT THIS STUDY?
Please contact (*site PI and contact information*) if you have any questions or concerns about this research study or contact *(name and contact info)* if you have concerns about your rights as a research participant or you are injured as part of this study.

SIGNATURE PAGE FOR CONSENT TO PARTICIPATE IN START MAIN STUDY

If you have read the informed consent (or if you have had it explained to you) and understand the information, and you voluntarily agree to join this study, please sign your name below.

____
Participant's name
(typed or printed)

____ ________
Participant's signature Date

OR

____
Participant's legal guardian or representative name
(typed or printed)

____ ________
Legal guardian/representative's signature Date

____
Witness's name
(typed or printed)

____ ________
Witness's signature Date

NOTE: This consent form with the original signatures MUST be retained on file by the principal investigator. A copy must be given to the participant. A copy should be placed in the participant's medical record.

A witness to the participant's signature is strongly encouraged.

Site Instruction:
If your IRB/EC requires a separate consent document for specimen storage, do not use this signature page. Instead, use the START STUDY SPECIMEN STORAGE (PLASMA AND URINE) SAMPLE CONSENT (Appendix A-2).

SIGNATURE PAGE FOR CONSENT TO STORE BLOOD AND URINE SPECIMENS

If you have read the information about stored specimens for future research in the informed consent (or if you have had it explained to you) and understand the information, please mark your choice in one of the boxes below and sign or initial as asked.

You can still be in the START study event if you do not want to have samples stored.

Please mark your choice:

☐ YES. I agree to have blood and urine samples collected and stored and used for future research. *Please sign below.*

☐ NO. Do not collect and store samples. *Please put your initials and today's date here →*

______________________________ *Do **NOT** sign below.*

____
Participant's name (typed or printed)

____ ____________
Participant's signature Date

OR

____
Participant's legal guardian or representative name (typed or printed)

____ ____________
Legal guardian/representative's signature Date

NOTE: This consent form with the original signatures MUST be retained on file by the principal investigator. A copy must be given to the participant. A copy should be placed in the participant's medical record.

APPENDIX B: SUMMARY OF BACKGROUND DATA FOR START DESIGN

CD4+ Cell Count, HIV RNA Level, and Risk of AIDS
Findings from the Multicenter AIDS Cohort Study (MACS) established that single measurements of HIV RNA and CD4+ count were important determinants of AIDS or death and that HIV RNA level predicted the rate of CD4+ decline. Data from larger epidemiological studies like the CASCADE Collaboration extended these findings by estimating 6-month risk of AIDS according to current CD4+ and HIV RNA levels. Predicted 6-month risk of AIDS among untreated patients aged 45 years with a CD4+ count of 500 cells/mm^3 ranged from 0.5% (HIV RNA = 3000 copies/mL) to 1.9% (HIV RNA = 300,000 copies/mL). Likewise, for patients in the same age group with an HIV RNA level of 100,000 copies/mL, the predicted 6-month risk of AIDS ranged from 1.0% (CD4+ = 350 cells/mm^3) to 0.5% (CD4+ = 500 cells/mm^3). Both current CD4+ count and current HIV RNA level were significant determinants of 6-month risk of AIDS.[8]

Other studies have provided evidence of a graded relationship between latest (or current) levels of CD4+ count and risk of AIDS diseases even among ART-naïve patients with CD4+ cell count ≥ 350 cells/mm^3.[3,108,109,110,111] Data for 17,609 ART-naïve individuals from the United Kingdom Collaborative HIV Cohort Study (UK CHIC) suggest that the graded relationship between AIDS or death and CD4+ count is evident among those with latest CD4+ cell counts ≥ 500/mm^3. In that cohort, estimates of absolute rates of AIDS or death are 24.5 (95% CI: 21.1 – 27.9), 15.5 (95% CI: 12.2 – 18.8) and 9.7 (95% CI: 7.0 – 12.4) per 1,000 person-years for patients with latest CD4+ count 350-499, 500-649 and ≥ 650/mm^3, respectively.

The risks for these groups with CD4+ counts above the current threshold for initiating ART are not negligible for individuals for whom there is the realistic hope of living close to a normal lifespan with use of ART. Based on these data, it is reasonable to hypothesize that for those with a CD4+ count > 500 cells/mm^3 (the target population for START), early initiation of ART will lead to a reduced risk of AIDS as compared to deferral of ART to CD4+ counts < 350 cells/mm^3 where AIDS risk is increased.

Furthermore, for a given CD4+ count, the risk of AIDS or death is lower in patients who have started ART than in those who are ART-naïve. Among individuals in the UK CHIC cohort, the corresponding rates of AIDS or death for those who started ART are 13.8 (95% CI: 11.7-15.9), 10.0 (95% CI: 7.8 – 12.2) and 9.1 (95% CI: 7.0-11.2) per 1,000 person-years for patients with latest CD4+ cell counts of 350-499, 500-649 and ≥ 650/mm^3, respectively. Overall, for those in the UK CHIC cohort, there was a reduced risk of AIDS or death for those on ART compared to those who were not (HR=0.58; 95% CI: 0.51-0.68) after adjustment for current CD4+ count (personal communication, Andrew Phillips). This reduced risk with ART was also observed in a EuroSIDA investigation. The lower risk of AIDS at high CD4+ counts among those on ART is also consistent with the finding from the MACS that AIDS events that occurred at higher CD4+ levels were associated with higher viral load levels.[112]

In the SMART trial, most (~80%) patient-time during follow-up was spent at CD4+ cell counts ≥ 350/mm^3.[11,113] Despite the relatively high CD4+ counts in the SMART study, there was a highly significant increased risk of AIDS or death in the patients assigned episodic ART (the DC group) compared to the viral suppression (VS) group (HR=2.6; 95% CI: 1.9-3.7). The difference in risk between the DC and VS groups was greater for fatal or non-fatal AIDS events (HR=3.6; 95% CI: 2.2-5.5) than for AIDS or death from any cause. The greater risk of AIDS or death in the DC compared to the VS group was explained in part by their lower CD4+ counts and higher HIV-RNA levels during follow-up.

These data suggest, but do not prove, that initiation of ART might reduce the rate of AIDS or death by an amount greater than would be predicted by the CD4+ cell count rise alone. The lower rates of AIDS or death on ART may be related to the lower viral load and the resulting reduction in immune activation,[114,115] other factors such as qualitative aspects of immunodeficiency not encapsulated in the CD4+ count,[116] and/or immunosuppression.[117] The effects of generalized immune activation and dysfunction induced by HIV infection are not well understood, and it is possible that these factors could be related to the development of non-AIDS events as well as AIDS events.

Relationship of CD4+ Count and Risk of Non-AIDS Events
While the potential reduction in risk of AIDS events is an important motivation for a trial of early ART, serious morbidity and mortality among patients with CD4+ count greater than 350 cells/mm^3 is dominated by conditions other than AIDS.[118,119,120,121,122,123] Until recently, non-AIDS conditions have been associated with other established risk factors and use of ART, but not HIV. Lau et al have found that in patients with CD4+ count > 200 cells/mm^3, risk of death from non-AIDS causes is greater than risk of death from AIDS causes.[124] The difference is more marked the higher the CD4+ count. Consistent with these data, of the 85 deaths that occurred in SMART, only 7 (8%) were from AIDS diseases. The risk of experiencing a pre-defined composite endpoint consisting of cardiovascular, hepatic, or renal disease outcomes was higher in the DC group compared with the VS group -- HR (DC/VS) = 1.7 (95% CI: 1.1-2.5).[11] The HR of non-AIDS death was 1.9 (95% CI: 1.1 – 2.9).[113] Further evidence that untreated HIV may result in an increased risk of death from non-AIDS diseases comes from EuroSIDA. In that large epidemiological investigation, the rate of non-AIDS-related death declined over the calendar time period corresponding to the introduction of effective ART across Europe.[121,125]

In D:A:D, the rates of death from hepatic causes, non-AIDS-defining malignancies[126] and deaths from non-AIDS causes were higher among those with lower levels of latest CD4+ cell counts. These relationships appear to be present even in those with a CD4+ cell count ≥ 350 cells/mm^3. Thus, the higher risk is not limited to those with very severe immunodeficiency. Similar findings have been reported from the CASCADE cohort, where cause-specific relative hazards per 100 /mm^3 higher CD4+ count of 0.89 (95% CI: 0.85 – 0.94) for risk of death from non-AIDS malignancy and 0.89 (95% CI: 0.83 - 0.92) for death from hepatic causes have been reported.[122] These relationships of risk

with CD4+ count are not so marked as for AIDS diseases, e.g., HR for death from AIDS is 0.76 (95% CI: 0.74 – 0.77) in CASCADE, but nevertheless are appreciable and attain high levels of statistical significance. Similarly, in the FIRST study, a study of initial ART in 1,397 patients, a 100-cell higher CD4+ cell count was associated with a 0.56 relative hazard for AIDS events (95% CI: 0.50-0.62) and a relative hazard of 0.86 (95% CI: 0.77-0.96) for non-AIDS morbidity and mortality.[127]

Cancer incidence has also been found to be increased in HIV-uninfected, immunosuppressed patients after kidney transplant.[128] A recent meta-analysis of HIV/AIDS studies and transplant studies concluded that both populations had an increased risk of many cancers and risk was similar.[129] Similarly, a report from the large Adult and Adolescent Spectrum of Disease and HIV Outpatient Study concluded that the incidence of many types of non-AIDS defining cancer were higher in HIV-infected persons compared to the United States general population.[130] Further, risk of lung cancer has been found to be higher in HIV infected, compared with uninfected, individuals even after adjustment for smoking.[131,132,133,134] A large study in U.S. veterans reported that hepatocellular carcinoma and non-Hodgkin's lymphoma risk was greater in HIV-positive than HIV-negative veterans. For hepatocellular carcinoma, the higher risk appears to be explained in part by co-infection with hepatitis C and alcohol abuse/dependence.[135] In SMART, the rate of non-AIDS malignancies was 30% higher among DC as compared to VS participants but this increased hazard was not significant (HR=1.3; 95% CI:0.7-2.1).[12]

Possible Positive and Negative Effects of ART on Non-AIDS Diseases
Liver disease has been reported to be the most common non-AIDS cause of death in HIV-infected patients receiving ART.[16] The balance in risks between known increased risk induced by some antiretroviral drugs[136,137] and the decreased risk of progressive liver diseases that may be associated with a reduction in immunodeficiency is not clear. While the impact of co-infection with hepatitis B and hepatitis C viruses on this risk/benefit is also unclear, data from SMART indicate that the potential for benefit is great. Risk of non-AIDS death in SMART was three- to fourfold higher among co-infected patients.[138]

The risk/benefit of early ART for CVD is also uncertain. In the large D:A:D cohort, an increased risk of myocardial infarction with increasing cumulative use of protease inhibitors was found (relative rate of 1.16 per year of protease inhibitor exposure).[22] This increased risk was estimated with high precision due to the 345 myocardial infarctions observed (95% CI: 1.10-1.23). Some of this effect appears to be explained by lipid levels. In D:A:D, the relative rate of myocardial infarction per year of protease inhibitor exposure was reduced to 1.10 (95% CI: 1.04-1.18) after adjustment for lipids. An increased risk of myocardial infarction or CVD associated with protease inhibitors has also been reported in other cohort studies.[139,140] Likewise, in two studies that compared HIV-infected individuals with the general population, myocardial infarction incidence was higher among those who were HIV infected.[141,142] In one of these studies, the increased risk was associated with protease inhibitors. The SMART and D:A:D group have also reported an increase risk of myocardial infarction associated wit use of abacavir.[23,24]

In SMART, there was a higher risk of CVD in the DC compared to the VS arm that was of borderline statistical significance (HR= 1.6; 95% CI 1.0 – 2.5; p=0.05). There is no evidence to suggest that this is related to the generally lower CD4+ counts in these patients. Consistent with this, the current CD4+ cell count does not show a strong association with risk of myocardial infarction in the D:A:D study. The higher risk in the DC arm in SMART could relate to changes in lipid profile resulting from stopping ART as there was an associated marked lowering in HDL cholesterol as well as in LDL cholesterol, resulting in an unfavorable total cholesterol-to-HDL ratio.[143] HDL cholesterol levels are reduced by HIV infection[144,145]. A possible mechanism involving impaired cholesterol efflux from macrophages by which the lower HDL cholesterol may increase risk of CVD in HIV-infected individuals has been described.[146] More generally, pro-atherogenic effects of HDL in the presence of inflammation have been the subject of several reviews.[147,148] This increased risk of CVD in the DC compared to the VS group was reduced when continuous ART was re-initiated (HR= 1.1; 95% CI: 0.7-1.8; p=00.64).

Impaired endothelial function may promote the development of atherosclerosis and other end-organ diseases through a number of mechanisms including its effects on the vascular wall, platelet and leukocyte adhesion, and coagulation.[149] Using stored specimens for SMART participants, four inflammatory and two coagulation markers were investigated as possible explanations for the increased risk of all-cause mortality, which was largely non-AIDS, and CVD in the DC compared to the VS group. Study entry levels of interluekin-6 (IL-6) and D-dimer were strongly related to all-cause mortality. Unadjusted ORs (highest versus lowest quartile) were 8.3 (95% CI 3.3-20.8; p<0.0001) and 12.4 (95% CI 4.2-37.0; p<0.0001), respectively. Furthermore, IL-6 and D-dimer levels increased one month after randomization by 30% and 16% in the DC group and by 0% and 5% in the VS group (p<0.0001 for both); the increase in the DC group was related to HIV-RNA levels at one month (p<0.0001).[150] IL-6 levels, an inflammatory marker produced in response to several factors including infection, are higher among HIV-infected than HIV-negative individuals.[151] D-dimer, a fibrin degradation product, has also been reported to be higher in HIV-infected as compared to HIV-negative individuals. In addition, D-dimer levels decreased in ART-naïve patients following the initiation of treatment.[152] HIV may increase risk of CVD and other end-organ diseases by activating inflammatory pathways in the vascular wall.[153]

HIV infection is associated with several types of renal dysfunction, including HIV-associated nephropathy (HIVAN), immune complex kidney disease and acute renal failure.[154,155] In a large cohort of women, the prevalence of proteinuria based on a urine dipstick examination was higher among women with higher HIV RNA levels and lower CD4+ count (≤ 200 versus > 200 cells/mm^3). Further, proteinuria was associated with an increased risk of a doubling of creatinine levels during follow-up.[156] A recent report in HIV-infected South Africans suggests that there may be a direct effect of HIV on renal disease as the prevalence of HIVAN was high among untreated individuals with HIV infection who did not have other risk factors for renal disease.[157] HIVAN is more commonly diagnosed in patients of Black African ethnic origin and appears to have

declined since the introduction of more potent ART regimens.[155,158,159,160] The impact of ART on renal disease other than HIVAN is less clear.[161] This may be due to associations with nephrotoxicity of some antiretroviral medications and with drugs used to prevent or treat complications of HIV infection.[154] Taken together, these studies indicate that HIV may increase the risk of renal disease. Whether early use of ART may prevent disease or slow progression is uncertain.

In summary, a clear picture on the likely risk/benefits of early ART in terms of hepatic, cardiovascular and renal disease does not emerge when considering together the data from epidemiological studies, laboratory studies, and SMART. A trial should provide definitive information on these risks and benefits.

CD4+ Cell Count and HIV RNA Response to ART

The majority of adults and children initiating ART now achieve a viral load < 50 copies/mL by 12 months.[162,163,164] Since the introduction of combination ART, this percentage has increased over time to a level (nearly 90% in some cohorts) at which future improvements will be difficult to achieve. On average, among patients achieving virologic suppression, there is a rapid rise in CD4+ cell count in the first several months of treatment followed by a continued slower rise.[165,166] With continued suppression of HIV RNA levels, CD4+ cell counts on average approach normal levels with long-term treatment.[167] However, achievement of normal levels likely requires several additional years for those starting ART with CD4+ counts < 350 compared to to > 350 cells/mm^3. In one report, median CD4+ counts after 7 years of uninterrupted ART were 660, 780, and 870 cells/mm^3, for those starting ART with counts 200-350, 350-500, and > 500 cells/mm^3, respectively.[168] Thus, if there is a continuum of risk between CD4+ count and AIDS and non-AIDS diseases as the epidemiological data indicate, patients who start ART at lower CD4+ counts will spend more time in CD4+ categories associated with a greater risk of morbidity and mortality. Further, about 15-20% of patients do not achieve a good immunological response (at least 25 cells) even with viral suppression after the first 6-9 months of ART,[169,170,171] and this poor immunologic response is associated with an increased risk of AIDS or death.[170,172] The consequences of this poor response on risk of AIDS and non-AIDS would be expected to be greater among patients who start ART at lower CD4+ counts. In addition, the immune response to immunization has been shown to be related to nadir CD4+ count, suggesting that immune competence may be compromised in those who defer ART to < 350 cells/mm^3.[173]

The CD4+ cell count response to different ART regimens is similar even though the virologic response varies. In three trials of protease inhibitor- and NNRTI-based regimens, CD4+ cell count increases were similar for the treatment groups even though NNRTI-based regimens (largely efavirenz) were superior in terms of virologic response to protease inhibitor-based regimens (largely nelfinavir and ritonavir-boosted regimens).[174,175,176,177] Similarly, a trial of lopinavir-ritonavir versus nelfinavir found that patients assigned lopinavir-ritonavir had a superior virologic response but a similar immunologic response as compared to patients assigned a nelfinavir-based regimen.[178] A recent review also noted that the recovery of CD4+ cell counts following the initiation

of ART appears independent of ART regimen but that comparisons over the long term were complicated by frequent changes in ART.[179]

These data indicate that a strategy of initiating ART at > 500 as compared to < 350 cells/mm^3 is likely to result in large CD4+ cell count differences between treatment groups over many years and beyond the planned duration of the study. During the initial years of follow-up, CD4+ cell counts will increase in the early ART group on average and decline in the deferred ART group. Even after those in the deferred group initiate ART, it will likely take several years to approach the average count achieved for a comparable patient (similar baseline count) in the early ART group. Also, the consequences of not achieving a robust immune response will be greater in terms of CD4+ count among those in the deferred ART arm.

Data indicate that new regimens that might be available to the deferred arm, and not the immediate ART arm, are unlikely to result in large differences in viral suppression or CD4+ cell count recovery compared with existing regimens. With the ART strategies to be studied, large differences in ART exposure, HIV RNA levels, and CD4+ count are expected over the entire follow-up period. These are ideal conditions to understand the risk and benefits of beginning ART at CD4+ counts > 500 versus waiting until the CD4+ declines to < 350 cells/mm^3.

CD4+ Decline among ART-Naïve Individuals
The CD4+ cell counts that define patient eligibility and the rate of CD4+ decline among those enrolled and randomized to the deferred group are important design parameters. Together, the entry CD4+ and rate of decline of CD4+ during follow-up define the average period of time the randomized groups differ with respect to use of ART. During this period of decline in CD4+ count among the deferred group before ART is initiated, it is hypothesized that risk of AIDS and non-AIDS conditions will increase. During this same time period, for the immediate ART group, it is hypothesized that risk of AIDS and non-AIDS diseases will decrease as a consequence of using ART that increases CD4+ cell count and suppresses HIV RNA levels. Any adverse effects of ART would also be more evident during this time period in the early ART group than later in follow-up when a larger percentage of those assigned to the deferred arm are also taking ART.

CD4+ declines among naïve patients depend on HIV RNA levels and are highly variable.[26] For example, a recent analysis of two cohorts indicated that the annual rate of decline was 55.9 cells (95% CI: 47.3-64.5) for those with HIV RNA levels at baseline of 10,001-40,000 copies/mL and 77.7 cells (95% CI: 68.2-87.3) for those with HIV RNA levels > 40,000 copies/mL.[27] This level and variability of CD4+ decline has been observed in other cohorts[180,181] and the decline has been found to be greater among older patients.[181] Of particular relevance to the proposed design, in the UK CHIC study, among patients with CD4+ cell count between 500 and 650 cells/mm^3 and with a median HIV RNA level of 15,800 copies/mL, the median time to use of ART or a CD4+ cell count < 350 cells/mm^3 was 2.5 years.

With consideration of the variability in the rate of decline, current guidelines indicate that the CD4+ cell count is usually the most important consideration in decisions to initiate ART and that counts among ART-naïve patients should be determined every 3 to 6 months. In START, CD4+ cell count will be monitored at least every 4 months for both the deferred and early ART groups.

Risk Factors for Non-AIDS Conditions

The etiology of each of the non-AIDS conditions – CVD, renal disease, liver disease, and non-AIDS-defining cancers – is multifactorial. For each disease, there are established risk factors, many of which are modifiable. HIV and ART may be additional risk factors for these diseases.

Older age is associated with an increased risk of each of these diseases. As HIV populations with access to ART age, these events will increase in absolute numbers. Smoking is an established risk factor for many diseases including cardiovascular, renal and liver disease and many cancers.[182] For CVD, the major risk factors are blood cholesterol level, blood pressure, smoking, overweight/obesity, diets high in fat and cholesterol, and diabetes mellitus.[183,184] These risk factors are additive in their effects on CVD risk, and for blood pressure and blood cholesterol, risk of a cardiovascular event is not restricted to those with hypertension or hypercholesterolemia. Risk equations for predicting long-term risk of CVD morbidity and mortality have been developed taking into account actual levels of blood pressure and blood cholesterol.[185]

Risk factors for renal disease are similar to those for CVD. Blood pressure and diabetes mellitus are major risk factors. Smoking and elevated blood cholesterol are also associated with an increased risk of renal disease.[186] African-Americans have a greater risk of kidney disease than other race groups.[187] Traces of protein on a casual urine dipstick and above average creatinine levels (e.g., 1.5-2.0 mg/dl) have been associated with long-term risk of ESRD.[188] The National Kidney Foundation in the United States has adopted a new classification scheme for chronic kidney disease as an estimated glomerular filtration rate (eGFR) < 60 mL/min per 1.73 m^2 or proteinuria.[189] Creatinine will be measured in START at baseline and all follow-up examinations in the first year and annually afterward in order to estimate GFR. The simplified Modification of Diet in Renal Disease equation will be used.[36]

Chronic hepatitis C infection is a major risk for cirrhosis, hepatocellular carcinoma, and death from liver disease.[190,191] The association between co-infection and risk of AIDS or death has not been consistent in different studies.[191,192,193] In SMART, approximately 15% of patients were co-infected with hepatitis C.[11] Risk of non-AIDS death was three- to fourfold higher among co-infected patients and the majority of these patients were co-infected with hepatitis C.[138] Chronic hepatitis B infection is associated with an increased risk of death from liver disease.[15] However, co-infection with hepatitis B virus is expected to be less common in the target population for START because of the location of sites.

If early ART is effective in reducing risk of these diseases, the public health impact may be much greater among individuals with established risk factors for each disease because a given proportional reduction in risk will have a much greater impact on absolute excess risk among individuals at higher risk. For this reason, subgroup hypotheses have been formulated that take into account risk of non-AIDS diseases.

Potential Loss of Future Drug Options with Early ART
As a consequence of incomplete HIV RNA level suppression with ART, replication in the presence of strong selective drug pressure occurs and resistance develops. The resistance that develops to a specific drug can confer cross-resistance to other drugs within the class.[194] While a second potent ART regimen can usually be constructed following virologic failure on the initial regimen, until recently subsequent durable regimens were more difficult to define. With the early potent ART regimens used, toxicities and poor adherence were common reasons for changing ART regimens.[195] Current ART is less toxic and more convenient to take, and it has proved to be highly successful in durably suppressing HIV.[196,197] Emergence of three-class resistance and exhaustion of all available active drugs has also proved to be a slow process.[198]

Nevertheless, ART is life-long, and excellent adherence is critical to reducing the risk of resistance. This is a major reason why treatment guidelines emphasize patient readiness. This will also be an important consideration for the START study – patients and clinicians must be prepared to initiate ART immediately if randomized to that arm. Since drug therapy is needed in order to select for resistance, the risk of developing resistance will be higher initially in patients randomized to early ART initiation compared with those deferring use of ART.

Effect of ART on HIV Transmission
An early study suggested that zidovudine reduced HIV transmission among serodiscordant couples.[199] Two studies in Africa found an association between plasma HIV RNA level and transmission risk.[31,32] In the Rakai study of 415 serodiscordant couples in which transmission risk increased with HIV RNA level of the infected partner, no transmission events occurred in those couples in which the infected partner had a plasma HIV RNA level < 1500 copies/mL.[31] Models predict that ART could decrease HIV transmission.[200,201] This however, may depend in part on changes in HIV risk behaviors following the initiation of ART.

The association between ART usage and HIV transmission risk behavior was evaluated in a substudy of the SMART study. Overall, transmission risk behavior was similar among participants randomized to continuous ART versus episodic ART. However, in the relatively small subgroup of participants (n = 194) off ART at baseline (and therefore, similar to participants in the START study), randomization to start ART was associated with a decrease in self-reported high-risk behavior (anal or vaginal sex without a condom, self-reported needle-sharing, or incident gonorrhea, chlamydia, or syphilis). These data suggest that earlier initiation of ART may decrease the risk of HIV transmission, both by decreasing viral load and by decreasing behaviors associated with HIV transmission.[202]

APPENDIX C: TIME AND EVENTS SCHEDULE

Requirement	Baseline (≤60 days **before** participant randomized unless noted below)	≤14 days **before** participant randomized	Follow-up visits in Y1				Follow-up visits **after** Y1 (every 4 months)	
			1	4	8	12	Annual (e.g. 24, 36, etc.)	Other q4 visits
Informed consent	X							
Demographics, including education	X							
Documentation of HIV infection[1]	X							
CD4+ cell count and CD4%	X[2]		X	X	X	X	X	X
Karnofsky score	X							
Serum or urine pregnancy test[3]		X[4]						
Targeted health history and clinical evaluation[5]	X		X	X	X	X	X	X
Nadir CD4+ cell count and CD4%, maximal HIV RNA documented in the medical record at any time in the past	X							
Up to 3 most recent additional CD4+ cell counts, CD4%s and HIV RNA levels available in the medical record	X		X	X	X	X	X	X

[1] By plasma HIV RNA viral load, a rapid HIV test or any licensed ELISA test; and confirmed by an ELISA, Western Blot, HIV culture, HIV antigen, HIV pro-viral DNA or a second antibody test by a method other than ELISA at any time prior to study entry

[2] Before randomization, two CD4+ cell count and % measurements are required at least 2 weeks apart, with the earlier occurring not more than 60 days before randomization.

[3] For women of child-bearing potential. Test must be done in the clinic and read by a clinician or laboratory technician.

[4] Also required anytime pregnancy is suspected or if a woman of child-bearing potential is being prescribed efavirenz or other Pregnancy Category D drug.

[5] To include weight, height, sitting blood pressure, pulse, and smoking status (smoking status at baseline and annually only). The following clinical events are reported as they occur: AIDS diagnoses (listed in Appendix E), non-AIDS events (listed in Appendix E), bacterial pneumonia, pulmonary embolism, deep vein thrombosis, new-onset diabetes mellitus, coronary artery disease requiring drug treatment, congestive heart failure, peripheral arterial disease, serious events, initiation of and changes of ART, and pregnancy outcomes.. In addition, for participants diagnosed with malignancy while on study, fresh frozen tissue or paraffin block, pathology slide(s) of the malignancy, and from US sites only, pre-treatment whole blood stored for future research will also be obtained, if possible.

Requirement	Baseline (≤60 days **before** participant randomized unless noted below)	≤14 days **before** participant randomized	Follow-up visits in Y1				Follow-up visits **after** Y1 (every 4 months)	
			1	4	8	12	Annual (e.g. 24, 36, etc.)	Other q4 visits
Findings from genotyping or other form of acceptable ART resistance testing, if available	X		X	X	X	X	X	X
Recording of selected concomitant medications	X					X	X	
Pregnancy status	X					X	X	
Quality of life assessment	X			X		X	X	
Use of alcohol and recreational drugs	X					X	X	
HIV transmission risk behaviors	X			X		X	X	
Health care utilization	X					X	X	
HIV RNA level	X		X	X	X	X	X	X
CBC: hemoglobin, hematocrit, white blood cell count (WBC) with differential and platelets	X					X	X	
CD8+ cell count and CD8%	X		X	X	X	X	X	X
Renal function measurement: serum creatinine	X		X	X	X	X	X	
Liver function measurements: ALT, AST, alkaline phosphatase, total bilirubin and albumin	X					X	X	
Glucose	X					X	X	
Lipids: total cholesterol, LDL, HDL, triglycerides	X					X	X	
Dipstick urinalysis for protein	X		X	X	X	X	X	

Requirement	Baseline (≤60 days **before** participant randomized unless noted below)	≤14 days **before** participant randomized	Follow-up visits in Y1				Follow-up visits **after** Y1 (every 4 months)	
			1	4	8	12	Annual (e.g. 24, 36, etc.)	Other q4 visits
Hepatitis B and C: hepatitis B surface antigen, core antibody, surface antibody; hepatitis C antibody; and, if available, hepatitis C genotype and viral load	X							
Resting ECG[6]	X					X	X	
ART regimen which participant will start if randomized to Early arm	X							
Current ART regimen			X	X	X	X	X	X
Self-reported adherence to ART			X	X	X	X	X	X
Stored plasma for future HIV-related research (consenting patients only) [7]	X		X	X	X	X	X	X
Stored urine for future HIV-related research(consenting patients only)	X		X	X	X	X	X	
Self-reported comprehension of study requirements **(Informed Consent substudy)**	X							

[6] At sites with a study-supplied ECG machine and a certified technician.
[7] Plasma for storage will also be collected each time the ART regimen is switched or stopped due to an elevated HIV RNA (or presence of resistance mutations). The sample will be used for HIV resistance testing to be done in batch mode at a later time.

APPENDIX D: START PROTOCOL TEAM

The **INSIGHT START Protocol Team** will oversee the implementation of the START study. The Protocol Team includes representatives from the community, from industry collaborators, and from different scientific and administrative disciplines. Members are:

- Co-Chairs: Abdel Babiker, Sean Emery, Fred Gordin, Jens Lundgren
- Blinded statistician: Abdel Babiker
- Unblinded statisticians: Birgit Grund, Andrew Phillips, Shweta Sharma
- NIAID statistician: Michael Proschan
- INSIGHT Executive Steering Committee representatives: H. Clifford Lane, James D. Neaton, Nick Paton
- Division of AIDS Medical Officer: Karin Klingman
- Division of AIDS Pharmacist: Ana Martinez
- Laboratory Representative: John Baxter
- ICC clinicians: Jose Arribas, Waldo Belloso, David Cooper, Gerd Fätkenheuer, Bernard Hirschel, Sally Hodder, Margaret Johnson, Ines Maria Otegui, Mauro Schechter
- ICC representatives: Daniela Gey, Nick Paton, Sarah Pett, Michael Vjecha
- Leadership CORE representatives: Eileen Denning, Gregg Larson, Sue Meger
- Community representatives: David Munroe, Claire Rappoport, Siegfried Schwarze
- Pharmaceutical company representatives: Guy De La Rosa, Eric Lefebvre, Jeff Tryon (Tibotec); Sandy Griffith (GlaxoSmithKline); Linda Fischer, Kristy Grimm (Bristol-Myers Squibb); Sandra Nusinoff Lehrman (Merck); Marisol Marinez-Tristani, Michael Norton, William Woodward (Abbott); Alexia Exarchos, James Rooney (Gilead)
- Other funders: Jean-Michel Molina and Bruno Hoen (ANRS); Gerd Fätkenheuer (BMBF); Richard Davey, Jr. (NIH Clinical Center); Brian Agan and Scott Wegner (Tri-Service AIDS Clinical Consortium/TACC, funded by NIAID/NIH).
- Other experts (including INSIGHT Interest Group representatives and substudy representatives not identified elsewhere): Jason Baker, Bruce Brew, Bill Burman, Andrew Carr, Matthew Dolan, Greg Dore, Daniel Duprez, Ezekiel Emanuel, Christine Grady, Jennifer Hoy, Ken Kunisaki, Alan Landay, Bruno Ledergerber, Ron Mitsuyasu, Amanda Mocroft, Deenan Pillay, Richard Price, Peter Reiss, Kevin Robertson, Jürgen Rockstroh, Michael Ross, Amalio Telenti, Edwina Wright

APPENDIX E: PRIMARY ENDPOINT DEFINITION

The primary composite endpoint for START is non-fatal serious AIDS events (or "AIDS*"), non-fatal serious non-AIDS (or "non-AIDS") events, and death from any cause. It includes the following components:

- Fatal AIDS or non-fatal AIDS* events
  These include opportunistic events consistent with the 1993 CDC expanded surveillance definition plus additional events associated with immunosuppression in the participant population targeted for enrollment. Esophageal candidiasis and chronic *Herpes simplex* infection will only be counted in the primary endpoint if fatal events.

  AIDS* events include:
  - Aspergillosis (invasive)
  - Bartonellosis
  - Candidiasis of the bronchi, trachea, or lungs
  - Invasive cervical cancer
  - Chagas disease (American trypanosomiasis) of the central nervous system (CNS)
  - Cytomegalovirus virus (CMV) disease (radiculomyelitis, meningoencephalitis, or other disease)
  - CMV retinitis
  - Extrapulmonary or disseminated coccidioidomycosis
  - Cryptosporidiosis with diarrhea > 1 month
  - Cryptococcosis, meningitis or extrapulmonary
  - HIV-related encephalopathy, including AIDS Dementia Complex
  - Disseminated *Herpes zoster*
  - Extrapulmonary or disseminated histoplasmosis
  - Isosporiasis with diarrhea > 1 month
  - Kaposi's sarcoma, mucocutaneous or visceral
  - Leishmaniasis (visceral)
  - Hodgkin's lymphoma
  - Non-Hodgkin's lymphoma, all cell types
  - Primary lymphoma of the brain
  - Tuberculosis, pulmonary and/or extrapulmonary
  - Microsporidiosis with diarrhea > 1 month
  - *Mycobacterium avium* complex (MAC), disseminated
  - Other nontuberculous species or unidentified species of *Mycobacterium*, disseminated
  - Nocardiosis
  - Penicilliosis, disseminated
  - Extrapulmonary *Pneumocystis jiroveci*
  - *Pneumocystis jiroveci* pneumonia
  - Recurrent bacterial pneumonia (2 episodes within 12 months)

- Progressive multifocal leukoencephalopathy (PML)
- *Rhodococcus equi* disease
- Recurrent *Salmonella* septicemia (2 episodes within 12 months)
- Toxoplasmosis of the brain
- Wasting syndrome due to HIV

AIDS events include:
- All conditions defined as AIDS* above
- Esophageal candidiasis
- Chronic *Herpes simplex*

- Non-fatal serious non-AIDS events ("non-AIDS")
    - Cardiovascular disease (CVD) (myocardial infarction, stroke, coronary revascularization)
    - End-stage renal disease (ESRD) (initiation of dialysis, renal transplantation)
    - Decompensated liver disease
    - Non-AIDS-defining cancers (excluding basal and squamous cell skin cancers)

- Deaths not attributable to AIDS

The INSIGHT Endpoint Review Committee (ERC) has established objective criteria for each event and its level of diagnostic certainty. These criteria are given in the *START Protocol Instructions Manual.* The ERC is responsible for reviewing each reported event to determine the level of diagnostic certainty. Events that are judged as confirmed or probable will be included in the primary analysis.

APPENDIX F: GUIDELINES FOR RESISTANCE TESTING

When initiating ART in either treatment group, resistance testing may be helpful to guide therapy; national or local treatment guidelines should be consulted.[203, 204] Resistance testing can also be used to guide treatment changes in participants experiencing virologic failure. The following provides background information and general comments regarding the use of resistance testing in managing ART.

There are two types of resistance tests: genotypic and phenotypic assays. A genotype detects resistance mutations to relevant viral genes and an interpretive algorithm is used to predict drug susceptibility. A phenotype measures the virus's ability to replicate in the presence of different drugs, with susceptibility reported as an IC_{50} fold change compared to a wild-type control. The genotypic assay is generally more sensitive in detecting early evidence of drug resistance, has a more rapid turn around time, and is usually less expensive than a phenotype.

Transmitted resistance, defined as resistance to at least one antiretroviral drug, has been reported to occur in 6-16% of newly infected patients from surveillance studies in the U.S. and Europe.[4] Resistance mutations may persist for prolonged periods in untreated chronically infected patients. Persistence of transmitted NNRTI resistance mutations is likely due to these mutations having little effect on viral fitness.

Studies suggest that suboptimal virologic responses may occur in patients with baseline resistance mutations. Recent data using highly sensitive resistance assays have demonstrated that for NNRTI-based regimens, the presence of NNRTI resistance prior to starting ART is predictive of virologic failure. Resistance testing in naive patients has also been demonstrated to be cost-effective based on a prevalence of at least 5% in an ART-naïve population, given specific assumptions about regimen failure rates and its consequences to future regimen outcomes.[205]

Guidelines for resistance testing in treatment-naïve patients
The decision to perform resistance testing immediately prior to initiating ART should consider: (1) the regimen to be prescribed; (2) availability of previous resistance tests (e.g., closer to the time of seroconversion); and (3) risk of having transmitted drug resistance based on local data. Additionally, the following general points regarding baseline resistance testing should be considered:

- Resistance testing is desirable to guide initial treatment if the prevalence of transmitted resistance is > 5% in the population where the patient is believed to have been infected, though it is reasonable to note that relying on geography and estimates of time of seroconversion may be imprecise for several reasons including the mobility of populations.
- In treatment-naïve patients, a genotype is the preferred assay to detect transmitted resistance.
- Ideally, resistance testing should be performed early after the diagnosis of HIV to increase the likelihood of detecting resistance. If a resistance test was done previously, then that test can be used to guide initial treatment.

- There are settings where resistance testing may not be necessary prior to starting treatment. These include regions of very low prevalence of transmitted drug resistance, as well as a reliance on regimens that are likely to be minimally impacted by typical transmitted resistance patterns.

Guidelines for resistance testing in treatment-experienced patients
There are no restrictions on the use of resistance testing for guiding treatment changes. Prospective studies have shown a benefit with drug resistance testing in patients with virologic failure.

Clinicians should refer to current guidance on switching ART because of virologic failure or suboptimal virologic response.[203, 204] Resistance testing is most helpful when performed while a patient is taking their failing regimen. A definitive resistance test result is more likely when the plasma viral load is > 1,000 copies/mL. In treatment-experienced patients, resistance testing is not as useful after patients have discontinued treatment, as drug resistant species may not be detected.

Resistance testing should not be the only consideration guiding treatment changes. Prior treatment history, adherence, drug intolerance, and pharmacokinetic issues should also be assessed. Furthermore, expert advice has been shown to be beneficial in patients with extensive prior ART experience and drug resistance.

APPENDIX G: SAMPLE SIZE JUSTIFICATION

Sample size has been estimated using data from several sources including the CASCADE Collaboration and the UK CHIC Cohort. To calculate event rates in START, the rate of CD4+ count change while ART naïve and after initiation of ART was modelled using data from CASCADE. The observed CD4+ counts, which were modelled in the square root scale (suggested by Box-Cox), were assumed to differ from the individual's true (unobserved) values by random measurement errors representing intra-patient variability. Before ART initiation, a person's true square root CD4+ trajectory incorporates a linear decline with slope varying between individuals plus true fluctuations represented by a Brownian motion process.[206] A similar model was used after ART initiation, except that the trajectory incorporated two rates of increase one up to 1 year after ART initiation and the other after 1 year.

Event rates are assumed to be determined by current true CD4+ count but initiation of ART in the deferred treatment group is determined by the observed CD4+ count. At baseline, it was assumed that 70% of patients would have CD4+ counts between 501 and 600 cells/mm^3, 20% between 601 and 700, and the remaining 10% would have CD4+ counts > 700 cells/mm^3. The estimated model parameters were used to calculate the predicted (true) CD4+ counts corresponding to the observed values.
Using the estimated slopes for CD4+, computer simulations were performed to estimate time spent in different CD4+ categories. Event rates within CD4+ categories were estimated using data from the CASCADE Collaboration [8] and from the UK CHIC Cohort.[34] CD4+ was assumed to be measured every 4 months; repeated after a month if the value crossed the CD4+ threshold for initiation of ART in the deferred treatment group. To allow for non-adherence to the deferred ART strategy, it was assumed that 70% of patients would not initiate ART until CD4+ count dropped to below 350 cells/mm^3 and 30 % would initiate ART earlier – 10% before the CD4+ declined to 400 cells/mm^3 and 20% while the CD4+ was between 350 and 400 cells/mm^3.

Based on this model and the assumptions cited, the percentage of patients initiating ART in the deferred arm after 1 year is estimated as 13%; by the end of the study, 75% of patients in the deferred arm will have started ART.

The event rates from the cohort studies that were used for the simulations are shown in Tables G-1 and G-2 below.

Table G-1 summarizes event rates for ART-naïve participants from UK CHIC and CASCADE. Rates of AIDS or death from any cause (AIDS/death) can be computed for both cohorts. Rates for AIDS* (serious AIDS events including all AIDS deaths) can only be estimated from CASCADE. AIDS/death rates are similar for the two cohorts. Serious AIDS event rates are much lower because non-AIDS deaths represent a large fraction of the deaths in each CD4+ category.

Table G-1: Event Rates Pre-ART by Latest Observed CD4+ Count

Endpoint	Cohort	CD4+	Person-years	Events	Rate
AIDS or death	UK-CHIC	<200	2,449	794	32.4
		200-349	7,062	349	4.9
		350-499	8,643	212	2.5
		500-649	5,704	89	1.6
		≥ 650	5,464	53	1.0
	CASCADE	<200	1,592	448	28.1
		200-349	5,148	235	4.6
		350-499	8,242	172	2.1
		≥ 500	15,277	160	1.0
AIDS*	CASCADE	<200	1,604	371	23.1
		200-349	5,136	172	3.3
		350-499	8,235	111	1.3
		≥500	15,260	95	0.6
Non-AIDS death	CASCADE	<200	1,557	83	5.3
		200-349	5,086	42	0.8
		350-499	8,232	46	0.6
		≥ 500	15,295	48	0.3
All-cause death	CASCADE	<200	1,557	147	9.4
		200-349	5,086	47	0.9
		350-499	8,232	50	0.6
		≥ 500	15,295	57	0.4

Table G-2 summarizes event rates following the initiation of ART for participants in the CASCADE study. Similar to the data for ART-naïve participants in Table 1 rates for AIDS* are much smaller than AIDS or death because non-AIDS deaths represent a large fraction of all deaths. This fraction increases with increasing CD4+ cell count.

Table G-2: CASCADE Event Rates after Starting ART by Latest Observed CD4+ Cell Count

	Latest CD4+ Cell Count (cells/mm^3)			
**Endpoint**	**<200**	**200-349**	**350-499**	**≥500**
AIDS or Death	10.7	2.2	1.3	0.6
AIDS*	8.2	1.7	0.6	0.3
Non-OD death	2.5	0.8	0.4	0.3
All-cause death	4.3	0.8	0.4	0.3

The early and deferred ART groups are to be compared in terms to time to first occurrence of any of the components of the composite primary endpoint. It is assumed that 5% of patients with primary outcome would have multiple events of both AIDS* and non-AIDS. Rates of non-AIDS morbidity were not available and the events in the cohorts used were not adjudicated. We assumed that the rate of non-fatal non-AIDS events was 4 times the death rate from non-AIDS causes resulting in an assumed ratio of non-AIDS to AIDS* events of 3.3. We also assumed that the percentage of accepted events after adjudication was 65% for AIDS* and 90% for non-AIDS events. The sensitivity of sample size to the ratio of non-fatal non-AIDS to non-AIDS death and to different adjudication rates are illustrated below in Table G-3. Sample size is shown assuming 90% power and a type 1 error of 0.05 (2-sided). Based on this table, the target number of events was set at 370. If 275 events are achieved instead of 370, power is approximately 0.80 to detect a hazard ratio of 0.714 at the 0.05 level of significance.

Table G-3: Sensitivity of Sample Size and the Event Target to Assumptions Concerning the Ratio of Non-AIDS to AIDS* Events and Event Adjudication Estimates

Ratio: Non-fatal Non-AIDS/Non-AIDS Deaths	% AIDS* Events Adjudicated	Composite Event Rate per 100 Person Years (Deferred Arm)	Hazard Ratio (average over follow-up)	Combined Sample Size	Event Target
3	65	2.40	0.706	4,244	351
4	65	2.81	0.712	3,822	369
5	65	3.23	0.717	3.472	383
3	75	2.49	0.700	3,900	333
4	75	2.91	0.707	3,545	353
5	75	3.33	0.712	3,245	367

APPENDIX H: REFERENCES ON INSIGHT WEBSITE

The INSIGHT website (**www.insight-trials.org**) will maintain updated links to the following documents referenced in the START protocol and to other information pertinent to the study:

- The *START Protocol Instructions Manual*

- The table "Antiretroviral Components Required for the Initial Regimen in START" with a current list of preferred antiretroviral regimens for use as initial therapy in START.

- Current product information on antiretroviral drugs used in START

- Information on antiretroviral drug availability and distribution from the INSIGHT Central ART Repository

- The DAIDS Table for Grading the Severity of Adult and Pediatric Adverse Events, Version 1.0, December 2004 (or "DAIDS AE Grading Table"), as applicable for START

- *Antiretroviral Pregnancy Registry* for voluntary reporting of pregnancies that occur during START

- INSIGHT Publications and Presentations Policy

APPENDIX I: LIST OF ACRONYMS

ACSR	AIDS Cancer Specimen Resource
ADS	Average Deficit Score
AE	Adverse Event
AIDS	Acquired Immunodeficiency Syndrome (see Appendix E)
AIDS*	Modified definition of AIDS (see Appendix E)
ALT	Alanine aminotransferase
ANRS	Agence Nationale de Recherches sur le SIDA et les Hépatites Virales
ART	Antiretroviral Therapy
AST	Aspartate aminotransferase
AWP	Average Wholesale Price
BMBF	Bundesministerium für Bildung und Forschung (German Ministry)
BP	Blood Pressure
cART	Combination Antiretroviral Therapy
CASCADE	Concerted Action on Seroconversion to AIDS and Death in Europe
CBC	Complete Blood Count
CDC	Centers for Disease Control and Prevention (U.S.)
CES-D	Center for Epidemiologic Studies Depression Scale
CFR	Code of Federal Regulations (U.S.)
CI	Confidence Interval
CNS	Central Nervous System
CPE	CNS Penetration Score
CRF	Case Report Form
CSF	Cerebrospinal Fluid
CVD	Cardiovascular Disease
D:A:D	Data Collection for Adverse Events of Anti-HIV Drugs
DAIDS	The Division of AIDS, NIAID, NIH (U.S.)
DC	Drug Conservation (Arm in SMART Study)
DHHS	Department of Health and Human Services (U.S.)
DNA	Deoxyribonucleic Acid
DSMB	Data and Safety Monitoring Board
DVT	Deep Vein Thrombosis
EAE	Expedited Adverse Event
ECG	Electrocardiogram
eGFR	Estimated Glomerular Filtration Rate
ELISA	Enzyme-Linked Immunosorbent Assay
EMEA	European Medicines Agency
ERC	Endpoint Review Committee
ESPRIT	Evaluation of Subcutaneous Pro-leukin in a Randomized International Trial
ESRD	End-stage Renal Disease
EU	European Union
FDA	Food and Drug Administration (U.S.)
FIRST	Flexible Initial Antiretrovirus Suppressive Therapies
GCP	Good Clinical Practice

GEE	General Estimating Equations
GFR	Glomerular Filtration Rate
GID	Generated Identification Number
HAART	Highly Active Antiretroviral Therapy
HBM	Human Biological Material
HBV	Hepatitis B Virus
HCV	Hepatitis C Virus
HDL	High Density Lipoprotein
HIV	Human Immunodeficiency Virus Type 1
HIVAN	HIV-Associated Nephropathy
HLA	Human Leukocyte Antigen
HR	Hazard Ratio
HVLT-R	Hopkins Verbal Learning Test
ICC	International Coordinating Center (INSIGHT)
IEC	Institutional Ethics Committee
IL-2	Interleukin-2
INSIGHT	International Network for Strategic Initiatives in Global HIV Trials
IRB	Institutional Review Board
LDL	Low Density Lipoprotein
MACS	Multicenter AIDS Cohort Study
mL	Milliliter
mm	Millimeter
NCI	National Cancer Institute, NIH (U.S.)
NFL	Neurofilament Protein
NIAID	National Institute of Allergy and Infectious Diseases, NIH (U.S.)
NIH	National Institutes of Health (U.S.)
NIMH	National Institute of Mental Health, NIH (U.S.)
NINDS	National Institute of Neurological Disease and Stroke, NIH (U.S.)
NNRTI	Non-nucleoside Reverse Transcriptase Inhibitor
Non-AIDS	Serious Non-AIDS Conditions (see Appendix E)
NRTI	Nucleoside/Nucleotide Reverse Transcriptase Inhibitor
OHRP	Office for Human Research Protections (U.S.)
PHI	Primary HIV Infection
PHS	Public Health Service (U.S.)
PI	Protease Inhibitor
PID	Participant Identification Number
PIM	Protocol Instructions Manual
QNPZ	Quantitative Neurocognitive Performance Z Score
RNA	Ribonucleic Acid
SAE	Serious Adverse Event
SD	Standard Deviation
SDMC	Statistical and Data Management Center (INSIGHT)
SF-12	Medical Outcomes Study Short-Form-12 Item Survey
SMART	Strategies for Management of Antiretroviral Therapy
START	Strategic Timing of Antiretroviral Treatment
SUSAR	Suspected Unexpected Serious Adverse Reactions

TACC	Tri-Service AIDS Commission, Department of Defense (U.S.)
U.K. CHIC	United Kingdom Collaborative HIV Cohort Study
U.S.	United States of America
VS	Viral Suppression (Arm in SMART Study)
WAIS-III	Wechsler Adult Intelligence Scale-III
WBC	White Blood Cell Count
WHO	World Health Organization

REFERENCES

[1] Severe P, Juste MAJ, Ambroise A, et al. Early versus standard antiretroviral therapy for HIV-infected adults in Haiti. N Engl J Med 2010; 363:257-265.

[2] World Health Organization. Antiretroviral therapy for HIV infection in adults and adolescents. 2010 revision. Available at http://www.who.int/hiv/pub/arv/adult2010/en/index.html. Accessed 13 September 2010.

[3] European AIDS Clinical Society (EACS). Guidelines for the clinical management and treatment of HIV infected adults in Europe. Version 5, November 2009 (http://www.eacs.eu/)

[4] Gazzard BG on behalf of the BHIVA Treatment Guidelines Writing Group. British HIV Association guidelines for the treatment of HIV-1 infected adults with antiretroviral therapy 2008. *HIV Medicine* 2008. 9:563-608.

[5] Addendum to BHIVA Treatment Guidelines. British HIV Association. 2009. Available at http://www.bhiva.org/AddendumtoTreatmentGuidelines.aspx. Accessed 24 Aug 2010.

[6] Panel on Antiretroviral Guidelines for Adults and Adolescents. Guidelines for the use of antiretroviral agents in HIV-1-infected adults and adolescents. Department of Health and Human Services. December 1, 2009; 1-161. Available at http://www.aidsinfo.nih.gov/ContentFiles/AdultandAdolescentGL.pdf. Accessed 24 Aug 2010

[7] Thompson MA, Aberg JA, Cahn P, et al. Antiretroviral treatment of adult HIV infection. 2010 recommendations of the International AIDS Society – USA Panel. JAMA 2010 304: 321-333.

[8] CASCADE Collaboration. Short-term risk of AIDS according to current CD4 cell count and viral load in antiretroviral drug-naïve individuals and those treated in the nonotherapy era. *AIDS* 2004;18:51-58.

[9] Egger M, May M, Chene G, et al and the ART Cohort Collaboration. Prognosis of HIV-1 infected patients starting highly active antiretroviral therapy: a collaborative analysis of prospective studies. *Lancet* 2002;360:119-129.

[10] May M, Sterne JAC, Sabin C, et al and the ART Cohort Collaboration. Prognosis of HIV-1 infected patients after initiation of HAART: collaborative analysis of prospective studies. *AIDS* 2007;21:1185-1197.

[11] The Strategies for Management of Antiretroviral Therapy (SMART) Study Group. CD4+ count - guided interruption of antiretroviral treatment. *N Engl J Med* 2006; 355:2283-2296.

[12] Silverberg, MJ, Neuhaus J, Bower M, et al. Risk of cancers during interrupted antiretroviral therapy in the SMART Study. *AIDS* 2007;21:1-7.

[13] The Strategies for Management of Antiretroviral Therapy (SMART) Study Group. Clinical outcomes in the SMART study after recommendation to re-initiate antiretroviral therapy for participants randomized to the CD4+ guided interruption strategy. *Ann Intern Med* 2008; 149:289-299.

[14] Emery S, Neuhaus J, Phillips A, et al. Clinical outcomes in antiretroviral naïve patients and those not taking antiretroviral therapy at entry to SMART *J Infect Dis* 2008;197:1133-1144.

[15] Mocroft A, Soriano V, Rockstroh J, et al. Is there evidence for an increase in the death rate from liver-related disease in patients with HIV? *AIDS* 2005; 19:2117-2125.

[16] The D:A:D Study Group. Liver-related deaths in persons infected with the human immunodeficiency virus. *Arch Intern Med* 2006;166:1632-1641.

[17] Qurishi N, Kreuzberg C, Lüchters G, et al. Effect of antiretroviral therapy on liver-related mortality in patients with HIV and hepatitis C virus infection. *Lancet* 2003; 362:1708-1713.

[18] Phillips AN, Neaton J, Lundgren J. The role of HIV in serious diseases other than AIDS. *AIDS* 2008;22:2409-2418. .

[19] Kitahata MM, Gange SJ, Abraham AG, et al. Effect of early versus deferred antiretroviral therapy for HIV on survival. N Engl J Med 2009; 360:1815-1826.

[20] When to Start Consortium. Timing of initiation of antiretroviral therapy in AIDS-free HIV-1 infected patients: a collaborative analysis of 18 HIV cohort studies. Lancet 2009; 373:1352-1363.

[21] The D:A:D Study Group. Combination antiretroviral therapy and the risk of myocardial infarction. *N Engl J Med* 2003;349:1993-2003.

[22] The D:A:D Study Group. Class of antiretroviral drugs and risk of myocardial infarction. *N Engl J Med* 2007;356:1723-1735.

[23] D:A:D Study Group. Use of nucleoside reverse transcriptase inhibitors and risk of myocardial infarction in HIV-infected patients enrolled in the D:A:D study: a multi-cohort collaboration. *Lancet* 2008;371:1417-1426..

[24] The SMART/INSIGHT and D:A:D Study Groups. Use of nucleoside reverse transcriptase inhibitors and risk of myocardial infarction in HIV-infected patients. *AIDS* 2008;22:F17-F24.

[25] The UK Collaborative HIV Cohort (CHIC) Study Steering Committee. HIV diagnosis at CD4 count above 500/mm^3 and progression to below 350/mm^3 without antiretroviral therapy. *J Acquir Immune Defic Syndr* 2007;46:275-278.

[26] Mellors JW, Muñoz A, Giorgi JV, et al. Plasma viral load and CD4+ lymphocytes as prognostic markers of HIV-1 infection. *Ann Intern Med* 1997; 126:946-954.

[27] Rodŕiguez B, Sethi AK, Cheruvu VK, et al. Predictive value of plasma HIV RNA level on rate of CD4 T-cell decline in untreated HIV infection. *JAMA* 2006; 296:1498-1506.

[28] Ware J, Jr., Kosinski M, Keller SD. A 12-Item Short-Form Health Survey: construction of scales and preliminary tests of reliability and validity. *Med Care.* Mar 1996;34:220-233.

[29] Wu AW, Huang IC, Gifford AL, et al. Creating a crosswalk to estimate AIDS Clinical Trials Group quality of life scores in a nationally representative sample of persons in care for HIV in the United States. *HIV Clin Trials.* 2005;6:147-157.

[30] Burman WJ, Grund B, Roediger MP et al. The impact of episodic CD4+ cell count-guided antiretroviral therapy on quality of life. *J Acquir Immune Defic Syndr* 2008;47:185-93.

[31] Quinn TC, Wawer MH, Sewankambo N, et al. Viral load and heterosexual transmission of human immunodeficiency virus type 1. Rakai Project Study Group. *N Engl J Med* 2000;342;921-929.

[32] Fideli US, Allen SA, Musonda R, et al. Virologic and immunologic dterminants of heterosexual transmission of human immunodeficiency virus type 1 in Africa. *AIDS Res Hum Retroviruses* 2001;17:901-910.

[33] Johnson VA, Brun-Vézinet F, Clotet B, et al. Update of the drug resistance mutations in HIV-1 : 2007. *Topics in HIV Medicine* 2007;15:119-124.

[34] Phillips AN, Gazzard B, Gilson R, et al. Rate of AIDS diseases or death in HIV infected antiretroviral therapy-naïve individuals with high CD4 count. *AIDS* 2007;21:1717-1721.

[35] Neaton JD, Grund B. Earlier initiation of antiretroviral therapy in treatment naive patients: implications of results of treatment interruption trials. *Current Opinion in HIV and AIDS* 2008;3:112-117.

[36] National Kidney Foundation: K/DOQI clinical practice guidelines for chronic kidney disease: evaluation, classification, and stratification. *Am J Kidney Dis* 2002;39: S1-S266.

[37] Hirsch MS, Brun-Vezinet F, D'Aquila R, et al. Antiretroviral drug resistance testing in adult HIV-1 infection. Recommendations of International AIDS Society-USA Panel. *JAMA* 2000;283:2417-2426.

[38] Vandamme AM, Sönnerborg A, Ait-Khaled M, et al. Updated European recommendations for the clinical use of HIV drug resistance testing. *Antivir Ther* 2004;9:829-848.

[39] Dybul M, Nis-Kraske E, Daucher M, et al. Long-cycle structured intermittent vesus continuous highly active antiretroviral therapy for the treatment of chronic infection with human immunodeficiency virus: effects on drug toxicity and on immunologic and virologic parameters. *J Infect Dis* 2003;188: 388-396.

[40] Fox Z, Phillips A, Cohen C, et al. Viral re-suppression and detection of drug resistance following interruption of a suppressive non-nucleoside reverse transcriptase inhibitor-based regimen. *AIDS* 2008;22:2279-2289.

[41] World Health Organization. Antiretroviral drugs for treatment of pregnant women and preventing HIV infection in infants: Towards Universal Access. Recommendations for a public health approach. August 7, 2006. (http://www.who.int/hiv/pub/guidelines/en/).

[42] Public Health Service. Recommendations for use of antiretroviral drugs in pregnant HIV-1 infected women for maternal health and interventions to reduce perinatal HIV-1 transmission in the United States. October 12, 2006. (http://AIDSinfo.nih.gov).

[43] Australian Antiretroviral Guidelines Panel. Australian commentary to the USA Department of Health and Human Services Antiretroviral Guidelines. October 10, 2006. (http://www.ashm.org.au/aust-guidelines/).

[44] Kalbfleisch JD, Prentice RL. The statistical analysis of failure time data. 2nd ed., New York, NY, John Wiley, 2002.

[45] O'Brien PC, Fleming TR. A multiple testing procedure for clinical trials. *Biometrics* 1979;35:549-56.

[46] Lan KKG, DeMets DL. Discrete sequential boundaries for clinical trials. *Biometrika* 1983;70:659-63.

[47] The Coronary Drug Project Research Group. 1981. Practical aspects of decision making in clinical trials: the Coronary Drug Project as a case study. *Cont Clin Trials* 1:363-376.

[48] Dean M, Carrington M, Winkler C, et al. Genetic restriction of HIV-1 infection and progression to AIDS by a deletion allele of the CKR5 structural gene. Hemophilia Growth and Development Study, Multicenter AIDS Cohort Study, Multicenter Hemophilia Cohort Study, San Francisco City Cohort, ALIVE Study. *Science* 1996; 273(5283):1856-1862.

[49] Liu R, Paxton WA, Choe S, et al. Homozygous defect in HIV-1 coreceptor accounts for resistance of some multiply-exposed individuals to HIV-1 infection. *Cell* 1996; 86(3):367-377.

[50] Samson M, Libert F, Doranz BJ, et al. Resistance to HIV-1 infection in caucasian individuals bearing mutant alleles of the CCR-5 chemokine receptor gene. *Nature* 1996; 382(6593):722-725.

[51] Mallal S, Nolan D, Witt C, et al. Association between presence of HLA-B*5701, HLA-DR7, and HLA-DQ3 and hypersensitivity to HIV-1 reverse-transcriptase inhibitor abacavir. *Lancet* 2002;359:727-732.

[52] Brew BJ. HIV Neurology. First ed. New York: Oxford University Press; 2001.

[53] Carne CA, Tedder RS, Smith A, et al. Acute encephalopathy coincident with seroconversion for anti-HTLV-III. *Lancet* 1985;2(8466):1206-8.

[54] Ho DD, Sarngadharan MG, Resnick L, et al. Primary human T-lymphotropic virus type III infection. *Ann Intern Med* 1985;103:880-883.

[55] Tambussi G, Gori A, Capiluppi B, et al. Neurological symptoms during primary human immunodeficiency virus (HIV) infection correlate with high levels of HIV RNA in cerebrospinal fluid. *CID* 2000;30(6):962-5.

[56] Abdulle S, Mellgren A, Brew BJ, et al. CSF neurofilament protein (NFL) - a marker of active HIV-related neurodegeneration. *J Neurol* 2007;254(8):1026-32.

[57] Martin A, Heyes MP, Salazar AM, et al. Progressive slowing of reaction time and increasing cerebrospinal fluid concentrations of quinolinic acid in HIV-infected individuals. *J Neuropsychiatry Clin Neurosci* 1992;4(3):270-9.

[58] Chiodi F, Keys B, Albert J, et al. Human immunodeficiency virus type 1 is present in the cerebrospinal fluid of a majority of infected individuals. *J Clin Microbiol* 1992;30(7):1768-71.

[59] Garcia F, Niebla G, Romeu J, et al. Cerebrospinal fluid HIV-1 RNA levels in asymptomatic patients with early stage chronic HIV-1 infection: support for the hypothesis of local virus replication. *AIDS* 1999;13(12):1491-6.

[60] Spudich SS, Nilsson AC, Lollo ND, et al. Cerebrospinal fluid HIV infection and pleocytosis: relation to systemic infection and antiretroviral treatment. *BMC Infect Dis* 2005;5:98.

[61] Staprans S, Marlowe N, Glidden D, et al. Time course of cerebrospinal fluid responses to antiretroviral therapy: evidence for variable compartmentalization of infection. *AIDS* 1999;13(9):1051-61.

[62] Cepok S, von Geldern G, Nolting T, et al. Viral load determines the B-cell response in the cerebrospinal fluid during human immunodeficiency virus infection. *Ann Neurol* 2007.

[63] An SF, Groves M, Gray F, Scaravilli F. Early entry and widespread cellular involvement of HIV-1 DNA in brains of HIV-1 positive asymptomatic individuals. *J Neuropathol Exp Neurol* 1999;58(11):1156-62.

[64] Gray F, Scaravilli F, Everall I, et al. Neuropathology of early HIV-1 infection. *Brain Pathol* 1996;6(1):1-15.

[65] Sinclair E, Gray F, Ciardi A, Scaravilli F. Immunohistochemical changes and PCR detection of HIV provirus DNA in brains of asymptomatic HIV-positive patients. *J Neuropathol Exp Neurol* 1994;53(1):43-50.

[66] McCrossan M, Marsden M, Carnie FW, et al. An immune control model for viral replication in the CNS during presymptomatic HIV infection. *Brain* 2006;129(Pt 2):503-16.

[67] Sacktor N, Tarwater PM, Skolasky RL, et al. CSF antiretroviral drug penetrance and the treatment of HIV-associated psychomotor slowing. *Neurology* 2001;57(3):542-4.

[68] Robertson KR, Robertson WT, Ford S, et al. Highly active antiretroviral therapy improves neurocognitive functioning. *J Acquir Immune Defic Syndr* 2004;36(1):562-6.

[69] Cohen RA, Boland R, Paul R, et al. Neurocognitive performance enhanced by highly active antiretroviral therapy in HIV-infected women. *AIDS* 2001;15(3):341-5.

[70] Price RW, Yiannoutsos CT, Clifford DB, et al. Neurological outcomes in late HIV infection: adverse impact of neurological impairment on survival and protective effect

of antiviral therapy. AIDS Clinical Trial Group and Neurological AIDS Research Consortium study team. *AIDS* 1999;13(13):1677-85.

[71] Tozzi V, Balestra P, Galgani S, et al. Positive and sustained effects of highly active antiretroviral therapy on HIV-1-associated neurocognitive impairment. *AIDS* 1999;13(14):1889-97.

[72] Ferrando S, van Gorp W, McElhiney M, et al. Highly active antiretroviral treatment in HIV infection: benefits for neuropsychological function. *AIDS* 1998;12(8):F65-70.

[73] Marcotte TD, Deutsch R, McCutchan JA, et al. Prediction of incident neurocognitive impairment by plasma HIV RNA and CD4 levels early after HIV seroconversion. *Arch Neurol* 2003;60(10):1406-12.

[74] White DA, Heaton RK, Monsch AU. Neuropsychological studies of asymptomatic human immunodeficiency virus-type-1 infected individuals. The HNRC Group. HIV Neurobehavioral Research Center. *J Int Neuropsychol Soc* 1995;1(3):304-15.

[75] Heaton RK, Grant I, Butters N, et al. The HNRC 500--neuropsychology of HIV infection at different disease stages. HIV Neurobehavioral Research Center. J *Int Neuropsychol* Soc 1995;1(3):231-51.

[76] Reger M, Welsh R, Razani J, Martin DJ, Boone KB. A meta-analysis of the neuropsychological sequelae of HIV infection. *J Int Neuropsychol Soc* 2002;8(3):410-24.

[77] Heaton RK, Velin RA, McCutchan JA, et al. Neuropsychological impairment in human immunodeficiency virus-infection: implications for employment. HNRC Group. HIV Neurobehavioral Research Center. *Psychosom Med* 1994;56(1):8-17.

[78] Instruction/Owner's Manual. Grooved Pegboard. In: Lafayette Instruments 1989.

[79] D'Elia LF, Satz P, Uchiyama CL, et al. Color Trails Test. Odessa, FL. In: Psychological Assessment Resources, Inc; 1996.

[80] The Psychological Corporation. Wechsler Adult Intelligence Scale-Revised. San Antonio, TX: The Psychological Corporation; 1981.

[81] Benedict RH, Schretlen D, Groninger L, Brandt J. Hopkins Verbal Learning Test-Revised: Normative data and analysis of inter-from and test-retest reliability. *The Clinical Neuropsychologist* 1998;12(1):43-55.

[82] Reitan RM. Manual for administration of neuropsychological test batteries for adults and children. Bloomington. In: Indiana University Press; 1969.

[83] Spreen O, Strauss E. A compendium of neuropsychological tests :administration, norms, and commentary. 2nd ed., New York, Oxford University Press: 1998

[84] Radloff LS. The CES-D scale: A self-report depression scale for research in the general population. *Applied Psychological Measurement* 1977;1:385-401.

[85] Letendre S, Marquie-Beck J, Capparelli E, et al for the CHARTER Group. Validation of the CNS penetration-effectiveness rank for quantifying antiretroviral penetration into the central nervous system. *Arch Neurol* 2008;65(1):65-70.

[86] O'Brien PC. Procedures for comparing samples with multiple endpoints. *Biometrics* 1984; 40:1079-1087.

[87] Faden R, Beauchamp TA. A History and Theory of Informed Consent. Oxford University Press; 1986.

[88] Emanual EJ, Wendler D, Grady C. What makes clinical research ethical? *JAMA* 2000; 283(20):2701-11.

[89] Bergler JH, Pennington AC, Metcalfe M, Freis ED. Informed consent: how much does the patient understand? *Clin Pharmacol Ther* 1980;27(4):435-39.

[90] Howard JM, DeMets D, BHAT Research Group. How informed is informed consent? The BHAT experience. *Control Clin Trials* 1981;2:287-303.

[91] Harrison K, Vlahov D, Jones K, Charon K, Clements ML. Medical eligibility, comprehension of the consent process, and retention of ijection drug users recruited for an HIV vaccine trial. *J Acquir Immune Defic Syndr* 1995;10:386-90.

[92] van Stuijvenberg M, Suur MH, de Vos S, et al. Informed consent, parental awareness, and reasons for participating in a randomised controlled study. *Arch Dis Child* 1998;79(2):120-25.

[93] Pace C, Emanuel EJ, Chuenyam T, et al. The quality of informed consent in a clinical research study in Thailand. *IRB* 2005;27:9-17.

[94] Pace C, Talisuna A, Wendler D, et al. Quality of parental consent in a Ugandan malaria study. *Am J Public Health* 2005;95:1184-89.

[95] Moodley K, Pather M, Myer L. Informed consent and participant perceptions of influenza vaccine trials in South Africa. *J Med Ethics* 2005;3:727-32.

[96] Karim QA, Karim SSA, Coovadia HM, Susser M. Informed consent for HIV testing in a South African hospital: Is it truly informed and truly voluntary? *Am J Pub Health* 1998;488:637-40.

[97] Leach A, Hilton S, Greenwood BM, et al. An evaluation of the informed consent procedure used during a trial of a *Haemophilus influenzae* type B conjugate vaccine undertaken in The Gambia, West Africa. *Soc Sci Med* 1999;48:139-48.

[98] Lynoe N, Hyder Z, Chowdhury M, Ekstrom L. Obtaining informed consent in Bangladesh. *N Engl J Med* 2001;344:460-61.

[99] Joubert G, Steinberg H, van der Ryst E, Chikobvu P. Consent for participation in the Bloemfontein vitamin A trial: How informed and voluntary? *Am J Public Health* 2003;93:582-84.

[100] Marshall PA, Adebamowo CA, Adeyemo AA, et al. Voluntary participation and informed consent to international genetic research. *Am J Public Health* 2006;96:1989-95.

[101] Taub HA, Baker MT, Sturr JF. Informed consent for research. Effects of readability, patient age, and education. *J Am Geriatr Soc* 1986;34(8):601-6.

[102] Davis TC, Holcombe RF, Berkel HJ, Pramanik S, Divers SG. Informed consent for clinical trials: A comparative study of standard versus simplified forms. *J Natl Cancer Inst* 1998;90(9):668-74.

[103] Coyne CA, Xu R, Raich P, et al. Randomized, controlled trial of an easy-to-read informed consent statement for clinical trial participation: A study of the Eastern Cooperative Oncology Group. *J Clin Oncol* 2003;21(5):836-42.

[104] Dresden GM, Levitt MA. Modifying a standard industry clinical trial consent form improves patient information retention as part of the informed consent process. *Acad Emerg Med* 2001;8(3):246-52.

[105] Young DR, Hooker DT, Freeberg FE. Informed consent documents: Increasing comprehension by reducing reading level. *IRB* 1990;12(3):1-5.

[106] NCI Template Consent Form, available at http://www.cancer.gov/clinicaltrials/understanding/simplification-of-informed-consent-docs/page3

[107] Hayes RJ, Bennett S. Simple sample size calculation for cluster-randomized trials. *Int J Epid* 1999;28:319-326.

[108] Olsen CH, Gatell J, Ledergerber B, et al. Risk of AIDS and death at given HIV-RNA and CD4 cell counts, in relation to specific antiretroviral drugs in the regimen. *AIDS* 2005; 19 (3): 319-330.

[109] Podlekareva D, Mocroft A, Dragsted UB, et al. Factors associated with the development of opportunistic infections in HIV-1-infected adults with high CD4(+) cell counts: A EuroSIDA study. *J Infect Dis* 2006; 194 (5): 633-641.

[110] Bogaards JA, Weverling GJ, Geskus RB, et al. Low versus high CD4 cell count as starting point for introduction of antiretroviral treatment in resource-poor settings: a scenario-based analysis. *Antiviral Therapy* 2003; 8 (1): 43-50.

[111] Weber R, Friis-Moller N, Sabin C et al. HIV and non-HIV related deaths and their relationship to immunodeficiency: The D:A:D Study. Abstract 106LB, 12th Conference on Retroviruses and Opportunistic Infections. Boston, USA, 2005.

[112] Hennessey KA, Giorgi JV, Kaplan AH, et al. AIDS onset at high CD4+ cell levels is associated with high viral load. *AIDS Research and Human Retroviruses* 2000; 16:103-107.

[113] The Strategies for Management of Antiretroviral Therapy (SMART) Study Group. Inferior clinical outcome of the CD4+ count - guided antiretroviral treatment interruption strategy in SMART: role of CD4+ counts and HIV-RNA levels during follow-up. *J Infect Dis* 2008;197:1145-1155.

[114] Lempicki RA, Kovacs JA, Baseler MW, et al. Impact of HIV-1 infection and highly active antiretroviral therapy on the kinetics of CD4+ and CD8+ T cell turnover in HIV-infected patients. *Proc Natal Acad Sci* 2000;97:13778-13783.

[115] Brenchley JM, Price DA, Schacker TW, et al. Microbial translocation is a cause of systemic immune activation in chronic HIV infection. *Nature Med* 2006;12:1365-1371.

[116] Lange CG, Valdez H, Medvik K, et al. CD4+ T-lymphocyte nadir and the effect of highly active antiretroviral therapy on phenotypic and functional immune restoration in HIV-1 infection. *Clin Immunol* 2002;102:154-161.

[117] Day CL, Kaufmann DE, Kiepiela P, et al. PD-1 expression on HIV-specfiic T cells is associated with T-cell exhaustion and disease progression. *Nature* 2006;443:350-354.

[118] Sackoff JE, Hanna DB, Pfeiffer MR and Torian LV. Causes of death among persons with AIDS in the era of highly active antiretroviral therapy: New York City. *Ann Intern Med* 2006;145:397-406.

[119] Crum NF, Riffenburgh RH, Wegner S, et al. Comparisons of causes of death and mortality rates among HIV-infected persons: analysis of the pre-, early, and late HAART (highly active antiretroviral therapy) eras. *J Acquir Immune Defic Syndr* 2006;41:194-200.

[120] Reisler RB, Han C, Burman WJ, Tedaldi EM, Neaton JD. Grade 4 events are as important as AIDS events in the era of HAART. *J Acquir Immune Defic Syndr* 2003;34:379-386.

[121] Mocroft A, Brettle R, Kirk O, et al. Changes in the cause of death among HIV positive subjects across Europe: results from the EuroSIDA study. *AIDS* 2002;16:1663-71.

[122] Smit C, Geskus R, Walker S, et al. Effective therapy has altered the spectrum of cause-specific mortality following HIV seroconversion. AIDS 2006;20:741-9.

[123] Palella FJ, Jr., Baker RK, Moorman AC, et al. Mortality in the highly active antiretroviral therapy era: changing causes of death and disease in the HIV outpatient study. *J Acquir Immune Defic Syndr* 2006;43:27-34.

[124] Lau B, Gange S, Moore RD. Risk of non-AIDS related mortality may exceed risk of AIDS-related mortality among individuals enrolling into care with CD4+ counts greater than 200 cells/mm^3. *J Acquir Immune Defic Syndr* 2006 (epublication).

[125] Mocroft A, Ledergerber B, Katlama C, et al. Decline in the AIDS and death rates in the EuroSIDA study : an observational study. *Lancet* 2003;362:22-29.

[126] The Data Collection on Adverse Events of Anti-HIV Drugs (D:A:D) Study Group. HIV-induced immunodeficiency and mortality from AIDS-defining and non-AIDS-defining malignancies. *AIDS* 2008;22:2143-2153.

[127] Baker JV, Peng G, Rapkin J, et al. CD4+ cell count and risk of non-AIDS diseases following initial treatment for HIV infection. *AIDS* 2008;22:841-848.

[128] Vajdic CM, McDonald SP, McCredie MRE, et al. Cancer incidence before and after kidney transplantation. *JAMA* 2006; 296:2823-2831.

[129] Grulich AE, van Leeuwen MT, Falster MO, Vajdic C. Incidence of cancers in people with HIV/AIDS compared with immunosuppressed transplant recipients: a meta-analysis. *Lancet* 2007;370:59-67.

[130] Patel P, Hanson DL, Sullivan PS, et al. Incidence of types of cancer among HIV-infected persons compared with the general population in the United States, 1992-2003. *Ann Intern Med* 2008;148:728-736.

[131] Clifford GM, Polesel J, Rickenbach M, et al. Cancer Risk in the Swiss HIV Cohort Study: Associations with immunodeficiency, smoking, and highly active antiretroviral therapy. *J Natl Cancer Inst* 2005;97:425 – 432.

[132] Engels EA, Brock MV, Chen J, et al. Elevated incidence of lung cancer among HIV-infected individuals. *J Clinical Oncology* 2006;24(9):1383-1388.

[133] Kirk GD, Merlo C, O'Driscoll P, et al. HIV infection is associated with an increased risk of lung cancer, independent of smoking. *CID* 2007;45:103-110.

[134] Chaturvedi A, Pfeiffer RM, Chang L, et al. Elevated risk of lung cancer among people with HIV. *AIDS* 2007; 21:207-213.

[135] McGinnis KA, Fultz SL, Skanderson M, et al. Hepatocellular carcinoma and non-Hodgkin's lymphoma: the roles of HIV, hepatitis C infection, and alcohol abuse. *J Clin Oncol* 2006;24:5005-5009.

[136] Nunez MJ, Martin-Carbonero L, Moreno V, et al. Impact of antiretroviral treatment-related toxicities on hospital admissions in HIV-infected patients. *AIDS Research and Human Retroviruses* 2006; 22: 825-829.

[137] van Leth F, Phanuphak P, Ruxrungtham K, et al. Comparison of first-line antiretroviral therapy with regimens including nevirapine, efavirenz, or both drugs, plus stavudine and lamivudine: a randomised open-label trial, the 2NN Study. *Lancet* 2004; 363:1253-1263.

[138] Tedaldi E, Peters L, Neuhaus J, et al. Opportunistic disease and mortality in patients co-infected with hepatitis B or C virus in the Strategic Management of Antiretroviral Therapy(SMART) study. *CID* 2008;47:1468-1475.

[139] Holmberg SD, Moorman AC, Williamson JM, et al. Protease inhibitors and cardiovascular outcomes in patients with HIV-1. *Lancet* 2002; 360:1747-1748.

[140] Iloeje UH, L'Italien G, Mauskoph J, et al. Protease inhibitor exposure and increased risk of cardiovascular disease in HIV-infected patients. *HIV Medicine* 2005; 6:37-4.

[141] Mary-Krause M, Cotte L, Simon A, Partisani M, Costagliola D. Increased risk of myocardial infarction with duration of protease inhibitor therapy in HIV-infected men. *AIDS* 2003; 17:2479-2486.

[142] Triant VS, Lee H, Hadigan C, Grinspoon SK. Increased acute myocardial infarction rates and cardiovascular risk factors among patients with HIV disease. *J Clin Endocrin Metab* 2007 (epublication).

[143] SMART CVD Risk Writing Group. Interruption of antiretroviral therapy and risk of cardiovascular disease in persons with HIV-1 infection: exploratory analyses from the SMART trial *Antivir Ther* 2008;13:177-187.

[144] Riddler SA, Smit E, Cole SR, et al. Impact of HIV infection and HAART on serum lipids in men. *JAMA* 2003;289:2978-2982.

[145] Rose H, Woolley I, Hoy J, et al. HIV infection and high-density lipoprotein: the effect of the disease versus the effect of treatment. *Metabolism Clinical and Experimental* 2006;55:90-95.

[146] Mujawar Z, Rose H, Morrow MP, et al. Human immunodeficiency virus impairs reverse cholesterol transport from macrophages. *PLoS Biology* 2006;4:1970-1983.

[147] Navab M, Anantharamich GM, Reddy, et al. Mechanism of disease: proatherogenic HDL – an evolving field. *Nature Clinical Practice* 2006;2:504-511.

[148] Chait A, Han CY, Oram JF, Heinecke JW. Lipoprotein-associated inflammatory proteins: markers or mediators of cardiovascular disease. *J Lipid Research* 2005;46:389-403.

[149] Cines DB, Pollak ES, Buck CA, et al. Endothelial cells in physiology and in the pathophysiology of vascular disorders. *Blood* 1998;91:3527-3561.

[150] Kuller LH, Tracy R, Belloso W, et al. Inflammatory and coagulation biomarkers and mortality in patients with HIV infection. *PLoS Medicine* 2008 5(10):e203.

[151] De Larrangas GF, Petroni A, Deluchi G, Alonso BS, Benetucci JA. Viral load and disease progression as responsible for endothelial activation and/or injury in human immunodeficiency virus-1-infected patients. *Blood Caogul Fibrinolysis* 2003;14:15-18.

[152] Wolf K, Tsakiris DA, Weber R, et al. Antiretroviral therapy reduces markers of endothelial and coagulation activation in patients with human immunodeficiency virus type 1. *JID* 2002;185:456-462.

[153] Fisher SA, Miller TL, Lipshultz SE. Impact of HIV and highly active antiretroviral therapy on leukocyte adhesion molecules, arterial inflammation, dyslipidemia, and atherosclerosis. *Atherosclerosis* 2006;185:1-11.

[154] Gupta, SK, Eustace JA, Winston JA, et al. Guidelines for the management of chronic kidney disease in HIV-infected patients: recommendations of the HIV Medicine Association of the Infectious Diseases Society of America. *Clin Infect Dis* 2005; 40:1559-1585.

[155] Kimmel PL, Barisoni L, Koop JB. Pathogenesis and treatment of HIV-associated renal disease: lesson from clinical and animal studies, molecular pathologic correlations, and genetic investigations. *Annal Intern Med* 2003;139:214-226.

[156] Szczech LA, Gange S, van der Horst C, et al. Predictors of proteinuria and renal failure among women with HIV infection. *Kidney International* 2002;61:195-202.

[157] Han TM, Naicker S, Ramdial PK, Assounga AG. A cross-sectional study of HIV-seropositive patients with varying degrees of proteinuria in South Africa. *Kidney Int'l* 2006;69:2243-2250.

[158] Cantor ES, Kimmel PL, Bosch JP. Effect of race on expression of acquired immunodeficiency syndrome-associated nephropathy. *Arch Intern Med* 1991;151:125-128.

[159] Lucas GM, Eustace JA, Sozio S, et al. Highly active antiretroviral therapy and the incidence of HIV-1-associated nephropathy: a 12-year cohort study. *AIDS* 2004; 18:541-546.

[160] Atta MG, Gallant JE, Rahman MH, et al. Antiretroviral therapy in the treatment of HIV-associated nephropathy. *Nephrol Dial Transplant* 2006;21:2809-2813.

[161] Szczech LA, Gupta SK, Habash R, et al. The clinical epidemilogy and course of the spectrum of renal diseases associated with HIV infection. *Kidney Int'l* 2004; 66:1145-1152.

[162] Moore RD, Keruly JC, Gebo KA, et al. An improvement in virologic response to highly active antiretroviral therapy in clinical practice from 1996 to 2002. *J Acquir Immune Defic Syndr* 2005; 39:195-198.

[163] Lampe FC, Smith CJ, Madge S, et al. Success of clinical care for human immunodeficiency virus infection according to demographic group among sexually infected patients in a routine clinic population, 1999 to 2004. *Arch Intern Med* 2007;167: 692-700.

[164] The Antiretroviral (ART) Collaboration. HIV treatment response and prognosis in Europe and North America in the first decade of highly active antiretroviral therapy: a collaborative analysis. *Lancet* 2006; 368:451-458.

[165] Smith CJ, Sabin CA, Lampe FC, et al. The potential for CD4 cell increases in HIV-positive individuals who control viraemia with highly active antiretroviral therapy. *AIDS* 2003; 17:963-969.

[166] Moore RD, Keruly JC. CD4+ cell count 6 years after commencement of highly active antiretroviral therapy in persons with sustained virologic suppression. *CID* 2007; 44:441-446.

[167] Mocroft A, Phillips A, Gatell J, et al. Normalization of CD4 counts in patients with HIV-1 infection and maximum virological suppression who are taking combination antiretroviral therapy: an observational cohort study. *Lancet* 2007; 370:407-413.

[168] Gras L, Kesselring AM, Griffin JT, et al. CD4 cell counts of 800 cells/mm^3 or greater after 7 years of highly active antiretroviral therapy are feasible in most patients starting with 350 cells/mm^3 or greater. *J Acquir Immune Defic Syndr* 2007;45:183-192.

[169] Pakker NG, Kroon ED, Roos MT, et al. Immune restoration does not invariably occur following long-term HIV-1 suppression during antiretroviral therapy. *AIDS* 1999;13(2):203-212.

[170] Moore DM, Hogg RS, Chan K, et al. Disease progression in patients with virological suppression in response to HAART is associated with the degree of immunological response. *AIDS* 2006;20:371-377.

[171] Moore DM. Regimen dependent variations in immunologic responses to HAART in the presence of virologic suppression. 11th International Workshop on HIV Observational Databases, Monte Carlo, 2007 (Abstract 23).

[172] The Antiretroviral Therapy (ART) Cohort Collaboration. Prognostic importance of initial response in HIV-1 infected patients starting potent antiretroviral therapy: analysis of prospective studies. *Lancet* 2003;362:679-686.

[173] Lange CG, Lederman MM, Medvik K, et al. Nadir Cd4+ T-cell count and numbers of CD28+ CD4+ T-cells predict functional responses to immunizations in chronic HIV-1 infection. *AIDS* 2003;17:2015-2023.

[174] Robbins GK, DeGrutolla V Schafer RW, et al. Comparison of sequential three-drug regimens as initial therapy for HIV-1 infections. *N Engl J Med* 2003; 249:2293-2303.

[175] INITIO Trial International Coordinating Committee. Virological and immunological outcomes at 3 years after starting antiretroviral therapy with regimens containing non-nucleoside reverse transcriptse inhibitor, protease inhibitor, or both in INITIO: open-label randomized trial. *Lancet* 2006;368:287-299.

[176] MacArthur RD, Novak RM, Peng G, et al. A comparison of three highly active antiretroviral treatment strategies consisting of non-nucleoside reverse transcriptase inhibitors, protease inhibitors, or both in the presence of nucleoside reverse

transcriptase inhibitors as initial therapy (CPCRA 058 FIRST Study): a long-term randomized trial. *Lancet* 2006;368:2125-2135.

[177] Rajesh G, Spritzler J, Chan E, et al. Effect of baseline- and treatment-related factors on immunologic recovery after initiation of antiretroviral therapy in HIV-1 positive subjects: results from ACTG 384. *JAIDS* 2006;42:426-434.

[178] Walmsley S, Bernstein B, King M, et al. Lopinavir-ritonavir versus nelfinavir for the initial treatment of HIV infection. *N Engl J Med* 2002;346:2039-2046.

[179] Battegay M, Nüesch R, Hirschel B, Kaufmann GR. Immunological recovery and antiretroviral therapy in HIV-1 infection. *Lancet Infect Dis* 2006;6:280-287.

[180] Lang W, Perkins H, Anderson RE, et al. Patterns of T lymphocye changes with human immunodeficiency virus infection: from seroconversion to the development of AIDS. *J Acquir Immune Defic Syndr* 1989;2:63-69.

[181] CASCADE Collaboration. Differences in CD4 cell counts at seroconversion and decline among 5739 HIV-1 infected individuals with well-estimated dates of seroconversion. *J Acquir Immune Defic Syndr* 2003;34:76-83.

[182] Peto R, Lopez AD, Boreham J, Thun M, Heath C. Mortality from smoking in developed countries. indirect estimation from national vital statistics. *Lancet* 1992;339:1268-1278.

[183] Stamler J. Established major coronary risk factors: historical overview. In: Marmot MG, Elliott P (eds) Coronary Heart Disease Epidemiology: From Aetiology to Public Health, Oxford University Press, June 2005.

[184] Stamler J, Neaton J, Garside DB, Davigulus ML. The major adult cardiovascular diseases: a global historical perspective. In: Lauer R, Burns TL, Daniels S (eds.). Pediatric Prevention of Atherosclerotic Cardiovascular Disease. Oxford University Press, 2006.

[185] Anderson, K.M., Odell PM, Wilson PW, Kannel WB. Cardiovascular disease risk profiles. *Am Heart J* 1991;121:293-298.

[186] Klag MJ, Whelton PK, Randall BL, et al. Blood pressure and end-stage renal disease. *N Engl J Med* 1996;334:13-18.

[187] Klag MJ, Whelton PW, Randall BL, et al. End-stage renal disease in African-American and White men. 16-year MRFIT findings. *JAMA* 1997;277:1293-1298.

[188] Ishani A, Grandits GA, Grimm RH, et al. Association of single measurements of dipstick proteinuria, estimated glomerular filtration rate, and hematocrit with 25-year incidence of end-stage renal disease in the Multiple Risk Factor Intervention Trial. *J Am Soc Nephrol* 2006;17:1444-1452.

[189] National Kidney Foundation: K/DOQI clinical practice guidelines for chronic kidney disease: evaluation, classification, and stratification. *Am J Kidney Dis* 2002;39: S1-S266.

[190] Pawlotsky JM. Pathophysiology of hepatitis C virus infection and related liver disease. *TRENDS in Microbiology* 2004;12:96-102.

[191] Rockstroh JK, Mocroft A, Soriano V, et al. Influence of hepatitis C virus infection on HIV-1 disease progression and response to highly active antiretroviral therapy. *J Infect Dis* 2005;192: 992-1002.

[192] Greub G, Ledergerber B, Battegay M, et al. Clinical progression, survival and immune recovery during antiretroviral therapy in patients with HIV-1 and hepatitis C coinfection. *Lancet* 2000; 356:1800-1805.

[193] Sulkowski MS, Moore RD, Mehta SH, Chaisson RE, Thomas DL. Hepatitis C and progression of HIV disease. *JAMA* 2002; 288:199-206.

[194] Hirsch MS, Brun-Vézinet F, Clotel B, et al. Antiretroviral drug resistance testing in adults infected with human immunodeficiency virus type 1: 2003 recommendations of an International AIDS Society – USA Panel. *CID* 2003;37:113-128.

[195] D'Arminio MA, Cozzi Lepri A, Rezza G, et al. Insights into the reasons for discontinuation of the first highly active antiretroviral theapy (HAART) regimen in a cohort of antiretroviral naïve patients. *AIDS* 2000;14:499-507.

[196] Moore RD, Keruly JC, Gebo KA, Lucas GM. An improvement in virologic response to highly active antiretroviral therapy in clinical practice from 1996 through 2002. J *Acquir Immune Defic Syndr* 2005;39:195-198.

[197] Lampe FC, Gatell JM, Staszewski S, et al. Changes over time in risk of initial virological failure of combination antiretroviral therapy. *Arch Intern Med* 2006;166:521-528.

[198] UK HIV Drug Resistance Database. Long term probability of detection of HIV-1 drug resistance after starting antiretroviral therapy in routine clinical practice. *AIDS* 2005;19:487-494.

[199] Musicco M, Lazzarin A, Nicolosi A, et al. Antiretroviral treatment of men infected with human immunodeficiency virus type 1 reduces the incidence of heterosexual transmission. Italian Study Group on HIV Heterosexual Transmission. *Arch Intern Med* 1994;154:1971-1976.

[200] Law MG, Prestage G, Grulich A, Van de Ven P, Kippax S. Modeling the effect of combination antiretroviral treatments on HIV incidence. *AIDS* 2001;15:1287-1294.

[201] Montaner JSG, Hogg R, Wood E, et al. The case for expanding access to highly active antiretroviral therapy to curb the growth of the HIV epidemic. *Lancet* 2006;368:531-536.

[202] Burman W, Grund B, Neuhaus J, et al. Episodic antiretroviral therapy increases HIV transmission risk compared to continuous therapy: Results of a randomized controlled trial. *J Acquir Immun Defic Synd* 2008; 49:142-150.

[203] Hirsch MS, Brun-Vezinet F, D'Aquila R, et al. Antiretroviral drug resistance testing in adult HIV-1 infection. Recommendations of International AIDS Society-USA Panel. *JAMA* 2000;283:2417-2426.

[204] Vandamme AM, Sönnerborg A, Ait-Khaled M, et al. Updated European recommendations for the clinical use of HIV drug resistance testing. *Antivir Ther* 2004; 9:829-848.

[205] Sax PE, Islam R, Walensky RP, et al. Should resistance testing be performed for treatment-naïve HIV-infected patients? A cost-effectiveness analysis. *Clin Inf Dis* 2005; 41:1316-1323.

[206] Taylor JMG, Cumberland WG, Sy JP. A stochastic model for analysis of longitudinal AIDS data. *J Amer Stat Assoc* 1994; 89:727-736.

Changes to START protocol, Version 1.0 (09 December 2008) to Version 2.0 (25 October 2010):

- Throughout: Removed references to pilot and definitive phase
- Throughout: Changed references to deferring ART strategy per “current guidelines” to per “current practice”.
- Throughout: Removed “consecutive” from CD4 eligibility criterion in all instances per Letter of Amendment #1
- Throughout: Clarified Inclusion Criterion 2 (“HIV infection documented by a plasma HIV RNA viral load, rapid HIV test or any licensed ELISA test; and confirmed by another test using a different method including but not limited to ~~such as~~ a rapid HIV test, Western Blot, HIV culture, HIV antigen, or HIV pro-viral DNA at any time prior to study entry”).
- Throughout: Clarified Exclusion Criterion 3 (“Presence of HIV progression such as oral thrush, unexplained weight loss, or unexplained fever at randomization”).
- Throughout: Clarified Exclusion Criterion 7 (“Diagnosis ~~History~~ of decompensated liver disease before randomization”).
- Throughout: Added “Fractures” as a secondary outcome. NOTE: This is not a new data collection item, simply adding an already-collected event to the list of secondary outcomes.
- Throughout: Took out references to substudy data collection (e.g., in Section 3.7.3, “In a subset of participants, neuropsychological tests…”), as these are in the substudies themselves.
- Section 1 (Synopsis/Rationale), Section 2.2.1 (previously “Treatment Guidelines”, now “Current evidence and practice on the timing of ART initiation”) and following: Rewritten to highlight where evidence from randomized trials is available to inform practice and de-emphasize dependence on guidelines. References added.
- Section 3.7.4 (Participant Follow-up): minor changes to better reflect data being collected at follow-up time points.
- Section 3.8.4 (Reporting Serious Events) and Section 4.1.8 (Abacavir Hypersensitivity Reaction): Removed references to a separate CRF for reporting abacavir hypersensitivity reactions to GSK. GSK have agreed to accept our Form 400 (Serious Event), on which all ABC hypersensitivity reactions must be reported, as sufficient for their purposes.
- Section 4.2 (CD4+ Cell Count and Viral Load Monitoring): Incorporated language regarding appropriate clinical management for participants in the deferred group whose CD4+ cell count is declining, per Jan 2010 communication to investigators.
- Sections 8.3.10 and 8.3.11 (Sample Size Estimation and Data Analysis, respectively, for the Informed Consent Substudy): Modified after performing the planned sample site re-estimation at the end of the pilot phase.
- Main study consents (Appendices A-1 and A-7): Incorporated language regarding updated US guidelines in consents per Jan 2010 communication to investigators.
- Main study consents: Modified language to permit sites to use a single consent for the main study that includes permission to collect and store specimens. A separate consent for specimen storage is still available (Appendix A-2); its signature page has been updated.

- Genomics Substudy consent, “What do you have to do if you are in the Genomics Substudy?”: corrected error in amount of blood to be drawn (9 mL instead of 6 mL; 6 mL is the amount being *stored*).
- Other consents: Incorporated Clarification Memos in regard to malignancy specimen collection (Appendix A-3), Genomics substudy involuntary withdrawal (Appendix A-4), and contraceptive language in main study Concise consent (Appendix A-7).
- Appendix C (Time & Events Schedule): Removed substudy data collection items, as there are now substudies open which are not included in the main protocol document. A Time & Events Schedule which includes data collection items for all substudies will be maintained on the INSIGHT website in a format that will allow sites to download it and delete items for those substudies in which they are not participating.
- Appendix D: Deleted INSIGHT Executive Committee (no longer exists). Updated START protocol team membership.

Summary of additional changes to START protocol Version 2.0, dated 25 October 2010

1. References to international regulatory documents (Declaration of Helsinki, EU clinical trials directives) modified to refer to “current version” rather than a specific version that may have since been updated. (Clarification Memo, 17 February 2011)
2. As planned, the sample size was re-estimated before enrollment was completed. Section 5.3 is added to the protocol to detail the updated sample size and primary event target. The inclusion criterion for age is changed to 35 years or older after 4,000 participants are enrolled to increase the likelihood of primary endpoints. (Letter of Amendment 2, 14 February 2013)
3. To comply with changes in EU guidance and individual country laws, language is added requiring reporting of serious adverse events within 24 hours following investigator knowledge of the event, for sites in EU member states. (Letter of Amendment 2.1, 13 May 2013)

Analysis Plan Informed Consent Substudy (26 Oct 2013)

INSIGHT START

Purpose of the study: to evaluate understanding of study information and satisfaction with the consent process among participants of the START study after receiving information from one of two different consent forms: a standard or a concise consent form.

Hypothesis: comprehension for those receiving the concise consent form will be at least as good as for those who received the standard form. Satisfaction will be higher in the concise group.

Unit of randomization: site
Stratified randomization- grouped by primary language, and centrally assigned by block randomization within stratum

Primary endpoint- proportion of participants who answered the randomization questions correctly

Secondary endpoints-
Comprehension score (based on # of correct answers to knowledge questions)
Composite score of satisfaction
Composite score of voluntariness

There is limited info available on site to site variability in understanding randomization. Based on data from the ESPRIT study, estimated coefficient of variation for true proportions between sites was 0.30 and estimated intra-site correlation coefficient 0.09. Based on this, an estimated 58 sites are needed to show a difference of 15% between participants receiving each consent who respond accurately to questions about randomization with a power of 0.80 at the two sided level of significance of 0.05 (a total of 1450 participants). With 179 sites and 4150 participants in the substudy, our power is substantially increased, and we should be able to readily identify differences between consents in the level of understanding of randomization, even if the site-to-site variability is higher than that found in ESPRIT.

Logistic regression appropriate to cluster randomized trials will be used to compare groups for the primary outcome. Odds ratios and confidence intervals. Primary analysis will include all participants who completed the post-consent questionnaire.

Mean levels of continuous secondary outcomes accounting for within-site correlation, and using the intrasite correlation coefficient. Secondary analyses will include those who did not complete the questionnaire and count incomplete responses as incorrect responses.

Statistical analysis

Descriptive statistics by:

- two-way tables with counts and proportions for categorical variables. P-values by chi-square or exact tests
- Median (IQR) (or mean-SD in a few cases) for continuous variables. P-values by Mann-Whitney/Kruskal-Wallis tests (or t-test/one way ANOVA respectively)
- Results shown by arm (i.e. consent assignment)

Univariable/multivariable analysis– *Correct answer to randomization question, Question A.6 (somewhat/very satisfied vs. other), Question A.26 (Could have refused to join START yes vs. no)*

- Multi-level logit models with random site effects (Stata's xtmelogit)
- Results of univariable models shown only if global Wald test was significant at the 0.20 level
- Model results shown as Odds Ratios and as %Risk differences
- In both cases, all comparisons are relative to the respective baseline category (usually the most prevalent).
- Continuous variables centered at the mean of their overall distribution thus risk differences
- in the case of continuous variables are relative to a typical person with the covariate of interest at the mean level.
- Risk differences and respective 95% CIs estimated through the Delta method (i.e. Stata's nlcom) and based on the estimate beta coefficients of the fixed effects and their variance-covariance matrix

Univariable/multivariable analysis– *Comprehension Score (0-16) and Satisfaction Score (0-8)*

- Quantile (median) regression with corrected SEs for clustering within sites (Stata's qreg2 - Parente, P.M.D.C. and Santos Silva, J.M.C. (2013), Quantile regression with clustered data, Department of Economics, University of Essex, Discussion Paper No 728.)
- Results of univariable models shown only if global Wald test was significant at the 0.20 level
- Model results shown as beta coefficients. All comparisons are relative to the respective baseline category (usually the most prevalent).

Univariable/multivariable analysis– *Voluntariness Score (0-4)*

- Ordinal logistic regression with corrected SEs for clustering within sites (Stata's ologit)
- Results of univariable models shown only if global Wald test was significant at the 0.20 level
- Model results shown as Odds Ratios (for higher scores)
- All comparisons are relative to the respective baseline category (usually the most prevalent).
- Due to violations of the proportionality of odds assumption, the dependent variable was recoded into a binary one (4 vs. <4) and logistic models were fitted (only for the multivariable models identified using the ordinal logistic regression)
