# Supplementary material for: A randomized trial comparing concise and standard consent forms in the START trial
Source: PLoS One. 2017 Apr 26;12(4):e0172607. doi: 10.1371/journal.pone.0172607 (PMC5406127; doi:10.1371/journal.pone.0172607)
Supplement: S2 File — List of approving IRBs/RECs with institution and FWA numbers. (DOCX) [file pone.0172607.s002.docx]

| **LSite Number** | **ORGANIZATION** | **FWA_NO** | **EC/IRB Name** |
| --- | --- | --- | --- |
| 003-011 | Harlem Family Center/Columbia University | FWA00002636 | Columbia University Medical Center |
| 007-004 | Tulane University Medical Center | FWA00002055 | Tulane Institutional Review Board |
| 008-012 | University of Illinois at Chicago | FWA00000083 | University of Illnois at Chicago |
| 009-004 | Washington DC VA Medical Center | FWA00002475 | Human Studies Subcommittee Institutional Board |
| 009-013 | George Washington Medical Faculty Associates | FWA00005945 | George Washington University |
| 012-004 | The Research + Education Group - Portland | FWA00009467 | Legacy Clinical Research and Technology, Regulatory Specialist Casey Bush |
| 014-001 | Henry Ford Health System | FWA00005846 | Henry Ford Health System |
| 016-010 | Wayne State University | FWA00002460 | Wayne State University Institutional Review Board Admin. Office |
| 017-004 | Denver Public Health | FWA00004689 | Colorado Multiple Institutional Review Board |
| 018-001 | Virginia Commonwealth University | FWA00005287 | Office of Research Subjects Protection Virginia Commonwealth University |
| 019-001 | Cooper University Hospital | FWA00000211 | Cooper University Hospital IRB |
| 023-001 | Bronx-Lebanon Hospital Center | FWA00001632 | Bronx-Lebanon Hospital Center |
| 023-002 | Montefiore Medical Center | FWA00000140 | Albert Einstein College of Medicine Committee on Clinical Investigations |
| 024-003 | Houston AIDS Research Team | FWA00000667 | Committee for the Protection of Human Subjects |
| 025-001 | Yale University School of Medicine | FWA00002571 | Yale University Human Investigation Committee |
| 025-004 | Community Research Initiative of New England | FWA00002933 | New England Institutional Review Board |
| 027-001 | Hennepin County Medical Center | FWA00006047 | Human Subject Research Committee |
| 028-001 | NJMS Adult Clinical Research Center | FWA00003913 | RBHS Newark IRB |
| 030-001 | VA Greater Los Angeles Healthcare System | FWA00000734 | VA Greater Los Angeles Healthcare System |
| 031-001 | Orlando Immunology Center | FWA00003356 | Sterling Institutional Review |
| 032-001 | Hillsborough County Health Deptment/University of South Florida | FWA00001669 | Institutional Review Board Florida Department of Health |
| 034-001 | Wake Forest University Health Sciences | FWA00001435 | Wake Forest university Health Sciences |
| 036-001 | AIDS Research and Treatment Center of the Treasure Coast | FWA00003859 | Sterling Institutional Services |
| 037-001 | University of Florida, Jacksonville | FWA00005790 | University of Florida, Jacksonville |
| 040-001 | Temple University | FWA00004964 | Temple University Institutional Review Board |
| 042-001 | University of North Texas Health Science Center | FWA00005755 | University of North Texas, Health Science Center at Fort Worth IRB |
| 050-001 | Florida Department of Health in Orange County/Sunshine Care Center | FWA00004682 | State Public Health Ethics and Human Research Protection Program, Florida Department of Health |
| 052-001 | Medical College of Wisconsin | FWA00000820 | Medical College of Wisconsin/Froedtert Hospital IRB |
| 052-003 | AIDS Resource Center of Wisconsin | FWA00013849 | Medical College of Wisconsin/Froedter Hospital IRB |
| 054-001 | Mayo Clinic | FWA00005001 | Mayo Clinic Institutional Review Board |
| 057-001 | Infectious Diseases Associates NW FL, PA | FWA00007033 | Sacred Heart Clinical Investigator Review Board CIRB |
| 065-001 | Cornell Clinical Trials Unit | FWA00000093 | Weill Cornell Medical College Institutional Review Board |
| 067-001 | Georgetown University | FWA00001080 | GU Institutional Review Board |
| 081-001 | Boston Medical Center | FWA00023612 | Boston University Medical Center, Institutional Review Board |
| 082-001 | UNC AIDS Clinical Trials Unit | FWA00004801 | Office of Human Research Ethics |
| 082-002 | Regional Center for Infectious Disease | FWA00004507 | The University of North Carolina at Chapel Hill Office of Human Research Ethics |
| 082-003 | Wake County Human Services | FWA00004801 | The University of North Carolina at Chapel Hill |
| 083-001 | The Ohio State University Wexner Medical Center | FWA00006378 | Office of Responsible Research Practices |
| 084-001 | UT Southwestern Medical Center | FWA00005087 | UT Southwestern Medical Center at IRB at Dallas |
| 085-001 | Duke University Health System | FWA00009025 | Duke University Health System |
| 086-001 | Puerto Rico-AIDS Clinical Trials Unit | FWA00005561 | UPR Medical Sciences Campus |
| 090-001 | University of Florida Health Services Center | FWA00005790 | University of Florida Health Science Center |
| 093-001 | St. Jude Children's Research Hospital | FWA00004775 | St Jude Children Research Hospital IRB |
| 095-001 | Texas Children's Hospital | FWA00000286 | Institutional Review Board for Human Subject Research, Baylor College of Medicine |
| 096-001 | David Geffen School of Medicine at UCLA | FWA00004642 | University of California Los Angeles Medical Institutional Review Board 1 |
| 097-001 | University of Southern California | FWA00005906 | USC Institutional Review Board Health Science Campus |
| 098-001 | UCSD Mother-Child-Adolescent Program | FWA00004495 | UCSD Human Research Protections Program |
| 099-001 | University of Puerto Rico Pediatric Research Site | FWA00005561 | UPR MSC University of Puerto Rico Medical Sciences Campus |
| 100-001 | San Juan Hospital | FWA00009049 | San Juan Hosptial |
| 104-001 | Ann & Robert H. Lurie Children's Hospital | FWA00001011 | Institutional Review Board Ann & Robert h. Lurie Children’s Hospital of Chicago |
| 104-002 | Mt. Sinai Hospital | FWA00005088 | Mount Sinai Hospital Institutional Review Board |
| 611-001 | Hospital General de Agudos JM Ramos Mejia | FWA00001767 | Comision de Bioetica/comite de Docencia e Investiation del Hospital General de Agudos |
| 611-002 | Hospital Italiano de Buenos Aires | FWA00004992 | Hospital Italiano de Buenos Aires |
| 611-003 | FUNCEI | FWA00004574 | Comite Etica Funcei-Helios Salud |
| 611-008 | Hospital Nacional Profesor Alejandro Posadas | FWA00003926 | Comite de Bietica Hospital Nacional |
| 611-009 | CAICI (Instituto Centralizado de Assistencia e Investigación Clínica Integral) | FWA00004569 | Comite de Etica-CAICI-CIAP |
| 611-011 | Hospital General de Agudos Teodoro Alvarez | FWA00001575 | Comite de Bioetica Hospital General de Agudos |
| 611-012 | Hospital Rawson | FWA00001702 | C.I.E.S del Nino y del Adulto Polo Hospitalario |
| 611-016 | Hospital Interzonal General de Agudos Dr. Diego Paroissien | FWA00018065 | Hospital Interzonal General de Agudos Dr Diego, Paroissien |
| 611-017 | CEIN | FWA00017458 | 1)Comite Indepeniente de Docencia CIDEL 2) Comision Asesora CAIBSH |
| 611-023 | Fundación IDEAA | FWA00017997 | Fundacion IDEAA IRB Neurguen |
| 611-101 | Fundación Arriarán | FWA00008315 | Comite Etico Cientifico del Salud Metropolitano Central |
| 611-302 | INCMNSZ (Instituto Nacional de Ciencias Médicas y Nutrición Salvador Zubirán) | FWA00014416 | INCMNSZ Instituto Nacional de Ciencias Medicas y Nutricion Salvador Zubiran |
| 612-001 | Taylor Square Private Clinic | FWA00002358 | St Vincent’s Hospital |
| 612-002 | St. Vincent's Hospital | FWA00002546 | St Vincent’s Hosptail Transiational Research Centre |
| 612-003 | Dr. Doong's Surgery | FWA00002512 | St Vincent’s Hospital |
| 612-006 | Holdsworth House Medical Practice | FWA00002513 | St Vincent’s Hospital |
| 612-007 | Royal Perth Hospital | FWA00000575 | Royal Perth Hospital |
| 612-017 | The Alfred Hospital | FWA00002341 | The Alfred Hospital |
| 612-018 | Melbourne Sexual Health Centre | FWA00003021 | The Alfred Hospital |
| 612-022 | Sexual Health and HIV Service - Clinic 2 | FWA00002614 | Prince Charles Hosp IRB |
| 612-024 | Prahran Market Clinic | FWA00002356 | St Vincent’s Hospital |
| 612-026 | Royal Adelaide Hospital | FWA00002233 | Royal Adelaide Hospital |
| 612-029 | Centre Clinic | FWA00002545 | St Vincent’s Hospital |
| 612-036 | East Sydney Doctors | FWA00002357 | St Vincent’s Hospital |
| 612-058 | Westmead Hospital | FWA00004983 | St Vincent’s Hospital |
| 612-402 | YRGCARE Medical Centre VHS, Chennai CRS | FWA0000672 | YRGCARE, YR Gaitonde Medical |
| 612-403 | Institute of Infectious Diseases | FWA00012951 | RAO Nursing Home, Survey No 691A-1A-1 CTB No 1897-B |
| 612-501 | University Malaya Medical Centre | FWA00007865 | U Malaya Med CTR IRB |
| 612-601 | Institute of Human Virology-Nigeria (IHVN) | FWA00014857 | 1)Institute of Human Virology 2) University of Abuja Teaching Hospital |
| 612-701 | 1 Military Hospital | FWA00006113 | The South African National Defence Force Institutional Review Board (SANDF) |
| 613-001 | Chulalongkorn University and The HIV-NAT | FWA00000943 | Chulalongkorn University The Institute Review Board of the Faculty of Medicine |
| 613-002 | Siriraj Hospital | FWA00002882 | Siriraj Hospital Mahidol University |
| 613-003 | Khon Kaen University, Srinagarind Hospital | FWA00003418 | The Khon Kaen University |
| 613-004 | Chonburi Regional Hospital | FWA00001604 | Institute for the Development of Human Research Protection Dept of Medical Science |
| 613-005 | Chiangrai Prachanukroh Hospital | FWA00001607 | 1)Chiangrai Prachanukroh Hospital 2) Institue for Development of Human Research Protection |
| 613-006 | Sanpatong Hospital | FWA00008410 | The Office of Secratory, Ethical Review Committee for Research in Human Subjects |
| 613-007 | Bamrasnaradura Infections Diseases Institute | FWA00008374 | 1)Institue for Development of Human Research 2)Bamrasnardua Infections Disease Institute |
| 613-008 | Ramathibodi Hospital | FWA00002882 | Ramathibodi Hospital Mahidol University |
| 613-009 | Research Institute for Health Sciences (RIHES) | FWA00005355 | Chiang Mai University |
| 614-003 | Tel Aviv Sourasky Medical Center | FWA00005013 | Tel Aviv Sourasky Medical Center |
| 614-004 | Rambam Medical Center | FWA00004980 | Helsinki Committee Ramban Medical Center |
| 621-001 | Centre Hospitalier Universitaire St. Pierre (C.H.U. St. Pierre) | FWA00006623 | Universitaire Ziekenhuis Antwerpen |
| 621-003 | Institute of Tropical Medicine | FWA00003251 | Ethisch Comitie Universitair Ziekenhuis Antwerpen |
| 621-006 | Universitaire Ziekenhuizen Gent | FWA00010368 | Local EC: Ethics Committee UZ Gent De Pintelaan Central EC: Ethics Committee UZ A, Wilrijkstraat |
| 621-007 | Universitair Ziekenhuis Gasthuisberg | FWA0000212 | Local EC: UZ Leuven, Commissie Medische Ethiek Central EC: Ethisch Comite, UZ Antwerpen, Wilrijkstraat |
| 621-101 | Centre Hospitalier de Luxembourg | FWA00008457 | CNER Comite National d Ethique de Recherche |
| 622-001 | Johann Wolfgang Goethe - University Hospital, Infektionsambulanz CRS | FWA00003677 | Ethikkommission der Medizinchen Fakultat der Universtat zu Koln |
| 622-004 | Universitätsklinikum Heidelberg | FWA0005282 | Ethik-Kommission der Medizinchen Fakultat der Universtat zu Koln |
| 622-005 | Universitätsklinikum Würzburg, Medizinische Klinik und Poliklinik II, Schwerpunkt Infektiologie CRS | FWA00004742 | Ethik-Kommission der Medizinshen Fakultat der Universtat zu Koln |
| 622-008 | Klinik I für Innere Medizin, Klinikum der Universität zu Köln | FWA00005638 | Ethik-Kommission der Medicinchen Fakultat der Universtat zu Koln |
| 622-010 | Medizinische Universitätsklinik - Bonn, Immunologische Ambulanz CRS | FWA00001907 | 1)Ethikkomission der Universitat zu Koln, 2) Ethikkommission an der Medizinischen Fakultat der Rheinischen |
| 622-016 | Klinikum der Universität München | FWA00001699 | Ethik-Kommission der Medizinischen Fakultat der Universitat zu Koln |
| 622-020 | Ifi - Studien und Projekte GmbH | FWA00006581 | Ethik-Kommission der Medizinischen Fakultat der Universitat zu Koln |
| 622-021 | Klinik fur Dermatologie, Venerologie, Allergologie | FWA00002340 | Ethik-Kommission der Medizinischen Fakultat der Universitat zu Koln |
| 622-030 | Universitätsklinikum Düsseldorf | FWA00000829 | Ethik-Kommission der Medizinischen Fakultat der Universitat zu Koln |
| 622-031 | Klinikum Dortmund gGmbH | FWA00015258 | 1)Ethik-Kommission der Medizinischen Fakultat der Universitat zu Koln  2)Ethik-Kommision der Arztekammer Westfalen-Lippe und der Medizinischen Fakultat der Westfalischen Wilhelms-Universitat Munster |
| 622-032 | Universitätsklinikum Regensburg | FWA00007450 | Ethik-Kommission der Medizinischen Fakultat der Universitat zu Koln |
| 622-033 | Universitätsklinikum Hamburg-Eppendorf | FWA00007534 | Ethik-Kommission der Medizinischen Fakultat der Universitat zu Koln |
| 622-034 | Gemeinschaftspraxis Jessen-Jessen-Stein | FWA00015891 | Ethikkommission der Universitat zu Koln |
| 622-352 | Universitätsklinikum Erlangen | FWA00007030 | 1)Ethik-Kommission der Medizinischen Fakultat der Universitat zu Koln  2)Ethik-Kommission der Medizinischen Fakultat der Friedrich-Alexander-Universitat Erlangen-Nurnberg |
| 622-355 | EPIMED-Gesellschaft für epidemiologische und klinische Forschung in der Medizin mbH | FWA00006365 | Ethik-Kommission der Medizinischen Fakultat der Universitat zu Koln |
| 622-357 | Medizinische Hochschule Hannover | FWA00006242 | Ethik-Kommission der Medizinischen Fakultat der Universitat zu Koln |
| 622-358 | ICH Study Center | FWA00006244 | Ethik-Kommission der Medizinischen Fakultat der Universitat zu Koln |
| 624-001 | Hospital de Santa Maria | FWA00005662 | CEIC-Parque da Saude de Lisboa |
| 624-003 | Hospital de Egas Moniz | FWA00005368 | CEIC-Parque de Saude de Lisboa |
| 624-012 | Centro Hospitalar do Porto | FWA00017401 | CEIC-Parque de Saude de Lisboa |
| 624-013 | Hospital Curry Cabral | FWA00016854 | CEIC-Parque de Saude de Lisboa |
| 625-001 | Hvidovre University Hospital, Department of Infectious Diseases | FWA00005693 | De Videnskabsetiske Komiteer for Region Hovedstaden |
| 625-002 | Aarhus Universitetshospital, Skejby | FWA00006033 | De Videnskabsetiske Komiteer for Region Hovedstaden |
| 625-003 | Rigshospitalet, Infektionsmedicinsk ambulatorium 8622 | FWA00006199 | De Videnskabsetiske Komiteer for Region Hovedstaden |
| 625-004 | Odense University Hospital | FWA00004088 | De Videnskabsetiske Komiteer for Region Hovedstaden |
| 625-101 | Oslo University Hospital, Ullevål | FWA00006596 | Regional Medical Research Ethics Committee South-East D |
| 625-203 | Sahlgrenska University Hospital | FWA00008826 | Regionala Etikprovningsnamnden i Goteborg |
| 625-204 | Skane University Hospital | FWA00018017 | Rigionala Etikprovningsnamnden i Goteborg |
| 625-301 | EMC Instytut Medyczny SA | FWA00015145 | Komisja Bioetyczna Centrum Ksztalcenia Podyplomowego |
| 625-302 | Wojewodzki Szpital Zakazny | FWA00001290 | Komisja Bioetyczna Centrum Medycznego Ksztalcenia Podyplomowego |
| 625-307 | Uniwersytecki Szpital Kliniczny | FWA00016885 | Komisja Bioetyczna Centrum Ksztalcenia Podyplomowego |
| 625-401 | Otto-Wagner-Spital SMZ /Baumgartner Hoehe | FWA00003025 | Ethikkommission der Medizinischen Universitat Wien |
| 625-402 | University Vienna General Hospital | FWA00003129 | Ethikkommission der Medizinischen Universitat Wien |
| 625-501 | Helsinki University Central Hospital, Dept of Infectious Diseases | FWA00004167 | Hospital District of Helsinki and Uusimaa, Coordinating Ethics Committee |
| 625-602 | University Hospital Plzen, CZ | FWA00016817 | 1)Ethics Committee of Faculty Hospital Plzen  2)Multicentric Ethics Commission, Hospital Liberec |
| 625-603 | Faculty Hospital Na Bulovce, Prague, Czech Rep. | FWA00006851 | 1)Multicentric Ethics Commission, Hospital Liberec  2)Ethics Commision, Faculty Hospital Bulovka |
| 625-701 | West Tallinn Central Hospital Infectious Diseases | FWA00007269 | Tallinn Medical Research Ethics Committee |
| 626-003 | Hospital Universitari Germans Trias i Pujol | FWA00001930 | CEIC-R de la Comunidad de Madrid |
| 626-004 | Hospital Clínic de Barcelona | FWA00000738 | CEIC-R de la Comunidad de Madrid |
| 626-009 | Hospital Universitario y Politécnico La Fe | FWA00013168 | 1)CEIC-R de la CAM  2)CEIC(Hosp. Universitario y Politecnico La Fe) |
| 626-012 | Hospital Universitario La Paz | FWA00006276 | CEIC-R de la CAM |
| 626-017 | Hospital Clínico San Carlos | FWA00001929 | CEIC-R de la CAM |
| 626-018 | Hospital Universitario Príncipe de Asturias | FWA00006277 | CEIC-R de la CAM |
| 626-019 | Hospital Universitario Doce de Octubre | FWA00006692 | 1)CEIC Hospital Doce de Octubre  2)CEIC Regional de la Comunidad de Madrid |
| 626-021 | Hospital La Princesa, Internal Medicine and Infectious Disease Service CRS | FWA00006275 | Comite Etico Regional de la Comunidad de Madrid |
| 626-023 | Hospital de la Santa Creu i Sant Pau | FWA00008876 | CEIC-R de la CAM |
| 626-024 | Hospital General Universitario de Valencia | FWA00006517 | CEIC-R de la CAM |
| 626-025 | Hospital del Mar | FWA00006198 | 1)CEIC-R de la CAM  CEIC-Parc de Salut Mar |
| 626-027 | Hospital Universitari Mutua Terrassa | FWA00006503 | CEIC-R de la CAM |
| 626-028 | Hospital Alvaro Cunqueiro | FWA00023682 | 1)Comite Etico de Investigacion Clinica Regional de la Comunidad de Madrid  2)CEIC Servixio Gallego de Salud – Conserjeria de Sanida |
| 631-001 | Hôpital Européen Georges Pompidou | FWA00006428 | CPP – Est II |
| 631-002 | Groupe Hospitalier Pitié-Salpêtrière | FWA00008088 | CPP – Est II |
| 631-003 | Hôpital de Bicêtre | FWA00001158 | CPP – Est II |
| 631-008 | Hôpital Henri Mondor | FWA00006430 | CPP – EST II |
| 631-016 | Hôpital Hôtel Dieu | FWA00009189 | CPP Est II |
| 631-018 | Hôpital Foch | FWA00006431 | CPP – Est II |
| 631-019 | Hôpital Saint-Louis | FWA00008207 | CPP – Est II |
| 631-020 | Hôpital Antoine Béclère | FWA00005921 | CPP – EST II |
| 631-024 | CHU de Besançon - Hôpital Jean-Minjoz | FWA00004248 | CPP – Est II |
| 631-027 | CHU Côte de Nacre - CAEN | FWA00008074 | CPP – EST II |
| 631-031 | Hôpital Saint-Antoine | FWA00006425 | CPP – EST II |
| 631-038 | Centre Hospitalier - Hôpital Gustave Dron | FWA00007060 | CPP – Est II |
| 632-007 | Ospedale San Raffaele S.r.l. | FWA00018939 | Comitato Etico dell’ Ospedale San Rafafele S.r.l. |
| 632-018 | Lazzaro Spallanzani IRCSS | FWA00006014 | Lazzaro Spallanzani IRCCS |
| 634-001 | Chelsea and Westminster Hospital | FWA00006205 | Yorkshire and the Humber – Leeds East Research Ethics Committee |
| 634-002 | Barts and the Royal London | FWA00006782 | Yorkshire and the Humber – Leeds East REC, Research Ethics Committee (REC) Centre |
| 634-003 | Royal Sussex County Hospital | FWA00003448 | NRES Committee Yorkshire & The Humber – Leeds East |
| 634-004 | University College London Medical School | FWA00003159 | Yorkshire & Humber – Leeds East REC |
| 634-005 | St. George's Hospital | FWA00001024 | Leeds East Research Ethics Committee, Yorkshire & the Humber REC Centre |
| 634-006 | Royal Free Hospital | FWA00001407 | Leeds East Research Ethics Committee, Yorkshire & Humber REC Centre Office |
| 634-008 | Sheffield Teaching Hospitals | FWA00004333 | Leeds East REC Yorkshire & Humber REC Centre Office |
| 634-009 | Leicester Royal Infirmary | FWA00000811 | Yorkshire and the Humber Leeds East REC, Research Ethics Committee (REC) Centre |
| 634-011 | St. Thomas' Hospital | FWA00005785 | Research Ethics Committee, Jarrow Centre |
| 634-016 | St. Mary's Hospital | FWA00006505 | Leeds (East) Research Ethics Committee, Yorkshire & Humber REC Office |
| 634-017 | Birmingham Heartlands Hospital | FWA00011794 | Leeds East Research Ethics Committee, Yorkshire & the Humber REC Centre Office |
| 634-019 | North Manchester General Hospital | FWA00017305 | Yorkshire and the Humber Leeds East, Leeds East Research Ethics Committee |
| 634-030 | Southmead Hospital | FWA00008107 | 1) NRES Committee Yorkshire & The Humber Leeds East 2) Jarrow Business Centre 3) Rolling Mill Road 4) Jarrow 5) Tyne and Wear |
| 634-038 | The James Cook University Hospital | FWA00003071 | NRES Committee, Yorkshire & The Humber Leeds East, Jarrow Business Centre |
| 634-040 | Queen Elizabeth Hospital Birmingham | FWA00005179 | Room 001, Jarrow Business Centre |
| 634-041 | Royal Bournemouth Hospital | FWA00016782 | NRES Committee Yorkshire & The Humber Leeds East, Jarrow Business Centre |
| 634-042 | Coventry and Warwickshire NHS Partnership Trust | FWA00017509 | Leeds East Research Ethics Committee, Yorkshire & The Humber REC Central Office |
| 634-043 | Gloucestershire Royal Hospital | FWA00017953 | NRES Committee Yorkshire & The Humber Leeds East, Jarrow Business Centre |
| 634-044 | Queen Elizabeth Hospital Woolwich | FWA00016874 | 1)Department of Cellular Pathology, Barts and the London NHS Trust The Royal London Hospital 2) Leeds (East) Research Ethics Committee Yorkshire & Humber REC Office |
| 634-045 | Royal Berkshire Hospital | FWA00017260 | NRES Committee, Yorkshire & The Humber Leeds East, Jarrow Business Centre |
| 634-102 | Mater Misericordiae University Hospital | FWA00005234 | Mater Misericordiae University Hospital Committee |
| 634-201 | Royal Victoria Hospital | FWA00012164 | 1)Alison Murphy, Research Manager, Belfast Health and Social Care Trust, Risk and Governance Research, 2) NRES Committee Yorkshire & The Humber Leeds East |
| 634-401 | University Hospital Centre Ibn Rochd | FWA00002898 | Comite d Ethique por la Recherche Biomedicale, Faculte de Medecine et de Casablanca, Centre Hospitalier Universitaire Bnou Rochd |
| 634-601 | MRC/UVRI Research Unit on AIDS | FWA00001354 | Uganda Virus Research Institute Research Ethics Committee |
| 634-602 | Joint Clinical Research Center (JCRC) | FWA00009772 | Uganda Virus Research Institute IRB Science and Ethics Committee |
| 635-005 | 5th Dept of Medicine & Infectious Diseases, Evangelismos General Hospital | FWA00006594 | 1)National Ethic Committee 2) National Organization for Medicines 3) Ethics Committee of National and Kapodistrian University of Athens |
| 635-006 | Korgialenio-Benakio Hellenic Red Cross | FWA00006445 | 1)National Ethics Committee; National Organization for Medicines 2) Scientific Council of Korgialenio-Benakio Hellenic Red Cross Hospital 3) IRB of National and Kapodistrian University of Athens Medical School |
| 635-009 | Attikon University General Hospital | FWA00006421 | 1)National Ethics Committee 2) National Organization for Medicine 3) Ethic Committee of National and Kapodistrian University of Athens |
| 635-010 | Andreas Syngros Hospital | FWA00006424 | 1)National Ethics Committee 2) National Organization for Medicicine |
| 635-012 | Hippokration University General Hospital of Athens | FWA00009087 | 1)National Organization for Medicines 2) National Ethics Committee 3) Ethics Committee of National and Kapodistrian University of Athens Medical School |
| 635-019 | AHEPA University Hospital | FWA00017089 | 1)National Organization for Medicines 2) National Ethic Committee 3) IRB of Aristotle University of Thessalonski |
| 636-001 | Unité VIH/SIDA Genèva | FWA00003804 | Comite dEthique de la Medicine Interne et de la Medicine Communautaire, Hospitaux Universitaire de Geneve |
| 636-003 | Bern University Hospital | FWA00000302 | Kantonale Ethikkommission Bern, Switzerland |
| 636-004 | University Hospital Basel | FWA00007946 | Ethikkommission beider Basel, EKBB, Hebelstrasse |
| 636-005 | University Hospital Zurich | FWA00002431 | 1)University Hospital Zurich Hematology Clinic 2) University Hospital Zurich Institute for Clinical Chemistry 3) University Zurich Institute for Medical Virology |
| 641-001 | Walter Reed Army Medical Center | FWA00000477 | COL Janine Babcock Chairman, Institutional Review Board Walter Reed Army Medical Center IRB |
| 641-002 | Walter Reed National Military Medical Center | FWA00000366 | 1)Timothy F. Donahue, CDR,MC,USN Chairman, Institutional Review Board National Naval Medical Center 2) Uniformed Services University of Health Sciences Institutional Review Board |
| 641-003 | Naval Medical Center Portsmouth | FWA00006001 | 1)USUHS IDIRB, Office of Research 2) Naval Medical Center Portsmouth Clinical Investigation Department |
| 641-005 | San Antonio Military Medical Center (SAMMC) | FWA00021471 | 1)Uniformed Services University of the Health Sciences Infectious Diseases Institutional Review Board 2) San Antonio Military Medical Center Institutional Review Board, Department of Clinical Investigations.MCHE-CI |
| 641-006 | Naval Medical Center San Diego | FWA00002342 | 1)Uniformed Services University of the Health Sciences 2) Naval Medical Center San Diego Clinical Investigation Department |
| 641-007 | Walter Reed National Military Medical Center | FWA00017749 | 1)Uniformed Services University of the Health Sciences Infectious Disease Institutional Review Board 2) Walter Reed National Military Medical Center Department of Research Programs |
| 644-001 | National Institutes of Health Clinical Center | FWA00005897 | NIAID IRB |
| 644-002 | SEREFO/CESAC MALI | FWA00012643 | SEREFO FMPOS, University of Barnako, |
| 648-001 | University of Miami | FWA00002247 | University of Miami Human Subject Research Office |
| 649-001 | Instituto de Infectologia Emílio Ribas - IIER | FWA00002035 | 1)Comite de Etica em Pesquisa do Instituto de Infectologia Emilio Ribas 2) Comissao Nacinal de Etica em Pesqueisa CONEP |
| 649-003 | Centro de Referência e Treinamento DST/Aids | FWA00000815 | Comite de Etica em Pesquisa CRT-DST/AIDS |
| 649-012 | Center for Infectious Diseases at the UFES | FWA00001406 | Comite de Etica em Pesquisa do Centro de Ciencias da Saude |
| 649-014 | LIM 56/HCFMUSP | FWA00001035 | Comissao de Etica para Analise de projetos de Pesquisas do Hopsital das Clinicas da Faculdade De Sao Paulo |
| 649-016 | SEI - Serviços Especializados em Infectologia | FWA00016940 | Comite de Etica em Pesquissa Maternidade Climerio de Oliveira/UFBA Rua do Limoeirco |
| 649-102 | Projeto Praça Onze Pesquisa em Saúde | FWA00000377 | 1)National IRB:CONCEP Esplanada dos Ministerios 2) Local IRB: Comite de Etica em Pesquisas da Faculdade de Medicina do Hospital Universitario Clementino Fraga Filho UFRJComite de Etica em Pesquisa do IPEC/FIOCRUZ |
| 649-106 | IPEC/FIOCRUZ | FWA00002548 | Comite Institucional de Bioetica de la Asociacion Civil Impacta Salud y Educacion |
| 651-001 | Asociación Civil Impacta Salud y Educación | FWA00001491 | Comite Institucional de Bioetica del Hospital Nacional Edgardo Rebagliati MartinsComite de Etica en Investigacion del Hospital Nacional Guillerno Almenara Irigoyen |
| 651-002 | Hospital Nacional Edgardo Rebagliati Martins | FWA00006688 | Comite Institucional de Bioetica del Hospital Nacional Edgardo Rebagliati Martins |
| 651-003 | Hospital Nacional Guillermo Almenara Irigoyen | FWA00006631 | Comite de Etica en Ivestigacion de Hospital Nacional Guillermo Almenara Irigoyen |
| 651-004 | Asociación Civil Impacta Salud y Educacion - Sede San Miguel | FWA00001491 | Comite Institucional de Bioetica de la Asociacion Civil Impacta Salud y Educacion |
| 651-006 | Via Libre | FWA00003081 | Comite Institucional de Bioetica de Via Libre |
| 652-001 | Desmond Tutu HIV Foundation Clinical Trials Unit | FWA00001637 | Prof M Blockman, Health Sciences Faculty Research Ethics Committee |
| 652-003 | Durban International Clinical Research Site | FWA00021425 | 1)Biomedical Research Ethics Administration Research Office University of KwaZulu Natal 2) South Africa Medicines Control Council Registrar of Mediciines |
| 652-004 | Clinical HIV Research Unit, University of Witswatersrand | FWA00000715 | 1)Ethics Committee: University of the Witswatersrand Human Research Ethics Committee (Medical) 2) Medicines Control Council: Medicines Control Council, Department of Health |
| 652-005 | Durban International Clinical Research Site (WWH) | FWA00021425 | 1)Biomedical Research Ethics Administration Research Office University of KwaZulu Natal 2) South Africa Medicines Control Council Registrar of Medicines |
