# Supplementary figures and images for: A randomized trial comparing concise and standard consent forms in the START trial
Source: PLoS One. 2017 Apr 26;12(4):e0172607. doi: 10.1371/journal.pone.0172607 (PMC5406127; doi:10.1371/journal.pone.0172607)

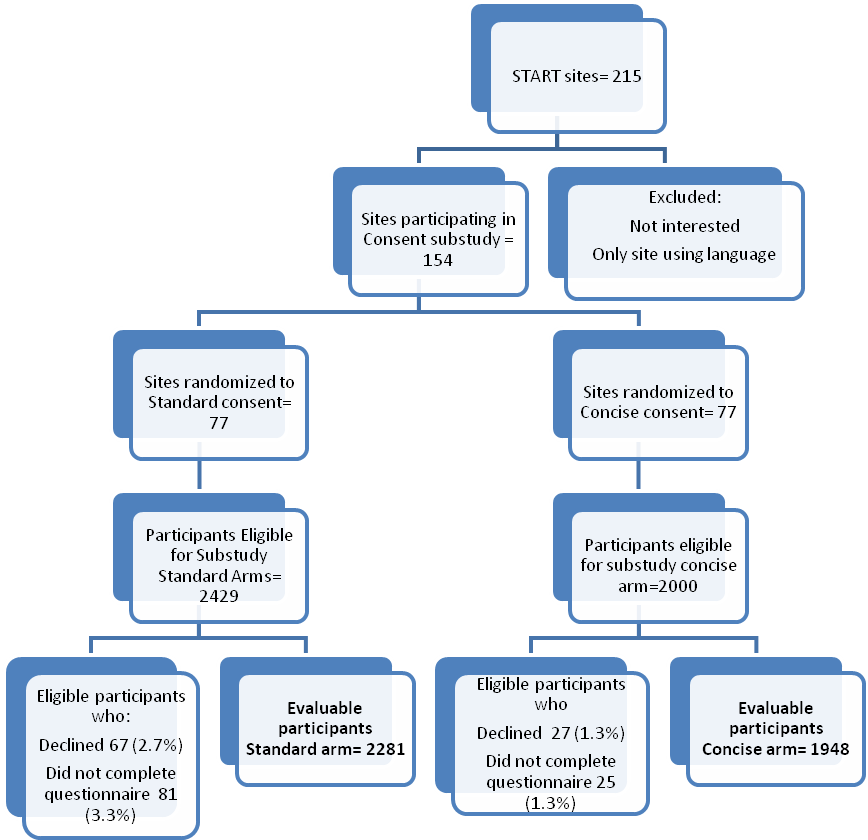


Figure 1. START Informed consent substudy participants

Supplement: S4 File — Participant Flow. (DOC) [file pone.0172607.s004.doc]
