# Supplementary material for: A randomized trial comparing concise and standard consent forms in the START trial
Source: PLoS One. 2017 Apr 26;12(4):e0172607. doi: 10.1371/journal.pone.0172607 (PMC5406127; doi:10.1371/journal.pone.0172607)
Supplement: S5 File — Denning et al. Reported consent processes and demographics: a substudy of the INSIGHT Strategic Timing of AntiRetroviral Treatment (START) trial. HIV Medicine 2015. (PDF) [file pone.0172607.s005.pdf]

DOI: 10.1111/hiv.12230
© 2015 British HIV Association
HIV Medicine (2015), **16** (Suppl. 1), 24–29

ORIGINAL RESEARCH

Reported consent processes and demographics: a substudy of the INSIGHT Strategic Timing of AntiRetroviral Treatment (START) trial

E Denning,[1] S Sharma,[1] M Smolskis,[2] G Touloumi,[3] S Walker,[4] A Babiker,[4] M Clewett,[5] E Emanuel,[6] E Florence,[7] A Papadopoulos,[8] A Sánchez,[9] J Tavel[10] and C Grady[11] for the International Network for Strategic Initiatives in Global HIV Trials (INSIGHT) START Study Group

[1]Coordinating Centers for Biometric Research, Division of Biostatistics, School of Public Health, University of Minnesota, Minneapolis, MN, USA, [2]National Institute of Allergy and Infectious Diseases (NIAID), Bethesda, MD, USA, [3]Department of Hygiene, Epidemiology and Medical Statistics, Athens University Medical School, Athens, Greece, [4]Medical Research Council (MRC) Clinical Trials Unit, London, UK, [5]Therapeutic and Vaccine Research Program, The Kirby Institute, Faculty of Medicine, University of New South Wales, Coogee, Australia, [6]Department of Medical Ethics and Health Policy, Perelman School of Medicine at the University of Pennsylvania, Philadelphia, PA, USA, [7]Institute of Tropical Medicine, Antwerp, Belgium, [8]4th Department of Internal Medicine, Athens Medical School, Attikon University General Hospital, Athens, Greece, [9]Institute for Clinical Research, Inc., at the Veterans Affairs Medical Center, Washington, DC, USA, [10]Genentech, South San Francisco, CA, USA and [11]Department of Bioethics, The Clinical Center, National Institutes of Health, Bethesda, MD, USA

Objectives

Efforts are needed to improve informed consent of participants in research. The Strategic Timing of AntiRetroviral Therapy (START) study provides a unique opportunity to study the effect of length and complexity of informed consent documents on understanding and satisfaction among geographically diverse participants.

Methods

Interested START sites were randomized to use either the standard consent form or the concise consent form for all of the site's participants.

Results

A total of 4473 HIV-positive participants at 154 sites world-wide took part in the Informed Consent Substudy, with consent given in 11 primary languages. Most sites sent written information to potential participants in advance of clinic visits, usually including the consent form. At about half the sites, staff reported spending less than an hour per participant in the consent process. The vast majority of sites assessed participant understanding using informal nonspecific questions or clinical judgment.

Conclusions

These data reflect the interest of START research staff in evaluating the consent process and improving informed consent. The START Informed Consent Substudy is by far the largest study of informed consent intervention ever conducted. Its results have the potential to impact how consent forms are written around the world.

Keywords: informed consent, START trial

Accepted 21 November 2014

Correspondence: Ms Eileen Denning, Coordinating Centers for Biometric Research, Division of Biostatistics, School of Public Health, University of Minnesota, 2221 University Ave SE, Suite 200, Minneapolis, MN, USA. Tel: 612-626-8049; fax: 612-624-2819; e-mail: eileen@ccbr.umn.edu

Introduction

Informed consent is widely accepted as an integral part of ethical clinical research [1,2] and includes three distinct elements: (1) disclosure of information to prospective research participants, (2) participant understanding of the information, and (3) a voluntary decision by the participant to enrol in the research. However, the goal of informed individuals making voluntary choices about research participation is often imperfectly realized. Data suggest that comprehension of important study information, especially side effects and randomization of treatment, varies considerably and can be unacceptably low [3–9]. In addition, some participants do not seem to understand that participation is their choice or that they can leave a study at any time [8,10–14].

Unfortunately, as more evidence emerges that comprehension is inadequate, written consent documents have become increasingly long and complex. Legal, ethical, regulatory, and risk reduction authorities have all had a hand in adding language to consent documents in the interests of 'protecting' the human participant as well as the research institution or sponsor.

Some groups have attempted to improve the consent process by improving the readability of consent documents [15–20]. Studies that compared typical consent forms with more simplified designs (usually lower in reading level and sometimes shorter) have found either equivalency between the regular and simplified forms [15–17] or statistically and clinically significant improvements in comprehension with a simpler form [18,19]. Studies that measured satisfaction found that participants clearly preferred the simplified forms [16,17].

In this report, we describe the characteristics of the sites and research participants that participated in the START Informed Consent Substudy, including consent procedures across sites.

Methods

The International Network for Strategic Initiatives in Global HIV Trials (INSIGHT) Strategic Timing of AntiRetroviral Therapy (START) study [21], a large international treatment strategy trial for antiretroviral-naïve HIV-positive individuals, provides an opportunity to compare participant understanding of a standard consent form with understanding of a more concise consent form, across a variety of languages and international research settings. Towards this end, participants consenting to START at sites participating in the START Informed Consent Substudy gave responses to questions regarding their satisfaction with and comprehension and voluntariness of the informed consent process. The primary endpoint is the proportion of participants giving correct answers to questions about randomization on a self-administered questionnaire. An important secondary endpoint will be based on composite scores on the comprehension section of the instrument that will include questions on study purpose and procedures, randomization, risks and side effects, and the right to refuse study participation. A comprehension score will be based on the number of correct answers to the knowledge questions. Other secondary endpoints will be measured by a composite score for the satisfaction questions, a composite score for the voluntariness questions, and a descriptive analysis of the changes made to the consent documents by each site's governing institutional review board (IRB)/ethics committee (EC).

All sites registering for START were invited to participate in the Informed Consent Substudy. The only criterion for a site's participation was that there be at least two sites participating in which individuals would be consenting primarily in the same language.

After the sample informed consent document (the 'standard' consent) for the START study was developed, the substudy protocol team prepared a second sample consent document in consultation with the main study protocol team. This 'concise' consent document contained all of the required and additional elements of informed consent [22], but was considerably shorter (1821 words versus 5927 in the standard consent) and at a slightly lower reading grade level (Flesch Kincaid Grade Level 9.2) than the standard template (Flesch Kincaid Grade Level 10.3). The concise version simplified sentences, reduced repetition, and made extensive use of tables and bulleted lists to convey information, while the standard version was written in a typical prose style. Both consents also included language informing potential START participants that by reading and signing the consent they were participating in a study of the informed consent process and would be asked to complete a questionnaire about their experience. Participants were told they could decline the questionnaire without consequence to their participation in START or their regular medical care.

Sites that chose to participate in the Informed Consent Substudy submitted both consent versions to their governing IRBs/ECs after making any necessary site-specific changes. Sites were asked to keep changes to a minimum and to ask their IRBs/ECs to do the same. Site-specific consent pairs (standard and concise) would undergo central textual analyses to assure that they remained different from one another after modification. Upon approval by the governing IRB/EC of both consents, each participating site was randomized to use either the standard or the concise consent for all participants. Randomization was stratified

© 2015 British HIV Association

by the primary language of consent at the site and done in blocks of two to assure balance between consent designs within each language.

The START Informed Consent Substudy is a cluster-randomized trial; the unit of randomization (between standard and concise consent documents) was the site rather than the individual participant. The cluster randomization strategy enhances the ability to compare the two consent documents and decreases the possibility that those obtaining consent will alter their usual process because of the substudy. Randomization by site also minimized the logistical burden at the site because the same consent form and process were utilized for each participant.

The substudy aim is to determine whether the concise consent is at least 'as good as' the standard consent. However, an odds ratio (OR) that indicates no statistically significant difference between groups does not necessarily mean that the response is similar in the two groups. A noninferiority approach will be taken in analysis to determine whether a nonsignificant OR indicates that the responses are similar enough to state that the concise consent provides sufficient understanding of study procedures compared with a standard consent. With a 7.5% noninferiority margin, the substudy has good power to make such a statement for the primary outcome (i.e. the proportion of participants understanding randomization in the concise consent group will not be more than 7.5% less than that in the standard consent group).

Sites were asked to have participants complete a self-administered questionnaire immediately after signing the consent form for START and before discussion of any other substudies or randomization to START. The questionnaire had 26 items addressing the participant's experience with the consent process (e.g. Did you feel adequately informed? Did you feel any pressure to join?) and relationship with site staff (e.g. How long have you known the staff? How well did they explain the study to you?) and 16 items to assess the participant's understanding of the information in the consent, including questions about the mechanism of randomization and possible risks and benefits of participating. Basic demographic information on each participant and screening CD4 measurements, if available, were collected on the screening form that was completed for each participant who signed a consent form for START.

The staff member who obtained the participant's signature on the consent form completed a seven-item questionnaire about how the consent form was used and how much person-time was used for the entire process for that individual participant. A clinical research staff member with primary involvement in the START study at each site also completed a one-time 14-item survey describing the general process for obtaining participant consent to START at their site. Questions included whether and what additional written information was provided to participants, whether participants received the consent form before the visit at which they signed it, and how the site typically assessed participant understanding of the study at the end of the consent process.

Results

Of the 221 sites registered to participate in START, 157 opened to the substudy, and 154 ultimately participated in the substudy by obtaining consent for at least one person to participate in START. Of the 64 START sites that did not open to the substudy, 23 did so because of a policy-level decision by their funding group that ruled out participation in START substudies. About one-third ($n = 14$) of the remaining 41 sites that did not participate in the Informed Consent Substudy did so because they did not have a language in common with another participating site (such as the single site in Nigeria, where the patient base speaks Hausa). However, this was not the only factor limiting participation. For example, in Sweden, where two sites enrolled participants into START, regulations strictly limit the length of informed consent documents to less than the length of our concise consent template. While 'participant information sheets' may be used to supplement the consent document, the length of these is also strictly limited, and so no meaningful difference between the standard and concise consents would be possible in Sweden. The START study's two-stage site start-up process (beginning enrolment at 101 pilot sites, then adding 120 new sites to complete the trial) was also a limiting factor as many of the sites that had not been part of the pilot did not want the delay in enrolment that may have occurred with participation in substudies.

Even though 14 of the 35 countries participating in START did not participate in the Informed Consent Substudy, the distribution of sites by geographical region and by number of participants consenting and randomized to START is similar to that for all sites participating in START [23]; most sites were located in Europe (43%), North America (28%) or South America/Mexico (14%). The geographical and language distribution of the 154 sites and 4473 individuals participating in the Informed Consent Substudy are shown in Table 1. Sites in South America and in Africa tended to be very high-enrolling sites in START, and together enrolled over half (53%) of the participants in the substudy, despite representing only 16% of the sites. This was also reflected in the primary language of consent identified for each site, with almost half of substudy enrolment (49%) occurring at sites that identified with a consent

© 2015 British HIV Association

Table 1 Geographical and language distribution of sites and individuals participating in the Strategic Timing of AntiRetroviral Treatment (START) Informed Consent Substudy

	Sites *n* (%)	Participants *n* (%)
Number participating	154	4473
Region		
Africa	3 (2.0)	816 (18.2)
Asia	9 (5.8)	257 (5.8)
Europe	66 (42.9)	1163 (26.0)
North America	43 (27.9)	589 (13.2)
Australia	11 (7.1)	113 (2.5)
South America and Mexico	22 (14.3)	1535 (34.3)
Primary language of consent at site		
English	74 (48.1)	1397 (31.2)
Spanish	25 (16.2)	920 (20.6)
Portuguese	9 (5.8)	800 (17.9)
Luganda	2 (1.3)	490 (11.0)
German	21 (13.6)	357 (8.0)
Thai	9 (5.8)	257 (5.8)
Greek	5 (3.3)	93 (2.1)
Flemish	3 (2.0)	81 (1.8)
French	2 (1.3)	51 (1.1)
Polish	2 (1.3)	25 (0.6)
Danish	2 (1.3)	2 (0.04)

language used in South America (Spanish or Portuguese) or Africa (Luganda).

The level of HIV research experience and aspects of the consent processes used at participating sites are described in Table 2. Sites with more HIV studies enrolled higher numbers of participants; the 24% of sites that have had more than 10 HIV studies ongoing in the past year enrolled 37% of the participants in the Informed Consent Substudy. The majority of sites (64%) provided written information in advance of the visit at which the participant signed a consent form for START. Among the sites providing written information in advance, 88% provided the consent document as part of that information. A majority (56%) of *all* sites provided the consent document to participants in advance of the visit at which the consent form was signed.

Site leaders, study investigators of record, coinvestigators, study coordinators, nurses, and other research staff were all active participants in the consent process, and in the majority of sites (58%), an investigator was the individual who obtained the participant's signature on the consent document. Sites spent anywhere from 15 min to 3 h in the consent process with each participant, with approximately half of the sites reporting less than 1 h spent and the other half reporting more than 1 h spent (Table 2). In the vast majority of sites (81%) the participant's understanding of the study was assessed interactively, with specific (8%) or general (73%) questions about understanding asked of the participant before the signature was obtained.

Table 2 Research experience and consent process at sites participating in the Strategic Timing of AntiRetroviral Treatment (START) Informed Consent Substudy

	n	%
Number of sites	154	
Number of other HIV studies ongoing or conducted in past year		
None	4	2.6
1–3	36	23.4
4–6	36	23.4
7–10	41	26.6
> 10	37	24.0
Site provided written information before the visit at which the individual gave consent	98	63.6
Among those, what information was provided?		
Consent document only	38	38.8
Consent document plus other information	48	49.0
Other information without consent document	12	12.2
Which research team members participated in the consent process (not mutually exclusive)?		
Site leader	77	50.0
Protocol investigator of record	55	35.7
Coinvestigator	100	64.9
Study coordinator	76	49.4
Study nurse	64	41.6
Others	17	11.0
Time typically spent in consent process		
≤ 15 min	6	3.9
> 15 min to < 1 h	74	48.1
≥ 1 h and < 3 h	69	44.8
> 3 h	5	3.3
How understanding was assessed before the individual was allowed to sign the consent form		
Formally (written test or specific questions)	12	7.8
Informally (nonspecific questions)	113	73.4
Clinical judgment	25	16.2
Other	4	2.6
Usually presented study information in a group setting prior to seeking individual consent	6	3.9

Demographic information and baseline CD4 cell count for participants in the substudy are found in Table 3. The proportion of individuals signing the consent form who were subsequently randomized to START (80%) was almost exactly the same in participants in the substudy as in all individuals consenting to participate in START, and demographic characteristics (age, race, gender and education) were similar to those of the entire randomized START cohort [23]. CD4 cell counts for participants in the Informed Consent Substudy were also similar to those for participants randomized to START.

Conclusions

These data indicate strong support to participate in this substudy within the INSIGHT network. Not only is this support evidence that the INSIGHT trial staff share a commitment to be involved actively in the consent process alongside potential trial participants, but it also reflects the

© 2015 British HIV Association

Table 3 Participants in the Strategic Timing of AntiRetroviral Treatment (START) Informed Consent Substudy

Substudy participants (all individuals **signing consent form** for START) (*n*)	4473
Age (years) [median (IQR)]	35 (28–44)
Gender female [*n* (%)]	1060 (23.7)
Race [*n* (%)]	
Asian	288 (6.4)
Black	1331 (29.8)
Latino/Hispanic	799 (17.9)
White	1870 (41.8)
Other	185 (4.1)
Formal education* [*n* (%)]	
Less than high school graduate or equivalent/year 12/'A' level equivalent	1298 (29.1)
High school graduate or equivalent/year 12/'A' level equivalent	952 (21.4)
Completed vocational training	404 (9.1)
Some college/some university	820 (18.4)
Bachelor's degree/university degree/TAFE degree	765 (17.2)
Any postgraduate education	219 (4.9)
CD4 count† (cells/μL) [median (IQR)]	628 (556–744)
Substudy participants subsequently **randomized** to START (%)	3584 (80.1)

IQR, interquartile range; TAFE, Technical and Further Education.
*Fifteen participants were missing education data. Percentages are of the 4458 participants with data available.
†Average of two screening values obtained at least 2 weeks apart within 60 days before randomization. This was available for 4470 substudy participants. Others were determined to be ineligible or otherwise not suitable for randomization before both screening CD4 count measurements were obtained.

concern shared by many researchers that the evolution of the consent document into its current form has not benefited potential research participants and may result in participants being less informed, not more, about the research in which they are being asked to take part.

IRBs/ECs were generally supportive of the Informed Consent Substudy and willing to keep changes to a minimum as allowed within their own institutional guidelines. All site-approved consent documents were reviewed by the START study sponsor (the University of Minnesota) before a site was randomized in the substudy, and there was rarely any concern that local changes had substantially reduced the difference between the standard and concise consent forms. A qualitative and quantitative description of the specific changes made at the local level is planned for a future paper.

The Informed Consent Substudy of START is by far the largest study of an informed consent intervention ever conducted [24]. As a study with broad international scope, the results of this substudy have the potential to impact how consent forms are written around the world. It may provide hard evidence for countries that have already regulated the length of consent documents because of concerns that the consent process was becoming less beneficial to participants as documents increased in length and complexity.

The START study is registered at clinicaltrials.gov (NCT00867048).

Funding

The START study is primarily funded by the National Institute of Allergy and Infectious Diseases of the National Institutes of Health under Award Number UM1-AI068641, the Department of Bioethics at the NIH Clinical Center and five NIH institutes: the National Cancer Institute, the National Heart, Lung, and Blood Institute, the National Institute of Mental Health, the National Institute of Neurological Disorders and Stroke and the National Institute of Arthritis and Musculoskeletal disorders. Financial support is also provided by the French Agence Nationale de Recherches sur le SIDA et les Hépatites Virales (ANRS), the German Ministry of Education and Research, the European AIDS Treatment Network (NEAT), the Australian National Health and Medical Research Council, and the UK Medical Research Council and National Institute for Heath Research. Six pharmaceutical companies (AbbVie, Inc., Bristol-Myers Squibb, Gilead Sciences, GlaxoSmithKline/ViiV Healthcare, Janssen Scientific Affairs, LLC, and Merck Sharp and Dohme Corp.) donate antiretroviral drugs to START.

Disclosures

The content is solely the responsibility of the authors and does not necessarily represent the official views of the National Institutes of Health. The University of Minnesota, the sponsor of START, receives royalties from the use of abacavir, one of the HIV medicines that can be used in START. The authors have no conflicts of interest to report.

Acknowledgements

We would like to thank the START participants without whom this work would not be possible. See the first article of this supplement [25] for a complete list of START investigators.

References

1 Faden R, Beauchamp TA. *A History and Theory of Informed Consent*. Oxford, Oxford University Press, 1986.
2 Emanuel EJ, Wendler D, Grady C. What makes clinical research ethical? *JAMA* 2000; **283**: 2701–2711.
3 Bergler JH, Pennington AC, Metcalfe M *et al.* Informed consent: how much does the patient understand? *Clin Pharmacol Ther* 1980; **27**: 435–439.

© 2015 British HIV Association

4 Howard JM, DeMets D, BHAT Research Group. How informed is informed consent? The BHAT experience. *Control Clin Trials* 1981; **2**: 287–303.

5 Harrison K, Vlahov D, Jones K *et al*. Medical eligibility, comprehension of the consent process, and retention of injection drug users recruited for an HIV vaccine trial. *J Acquir Immune Defic Syndr* 1995; **10**: 386–390.

6 van Stuijvenberg M, Suur MH, de Vos S *et al*. Informed consent, parental awareness, and reasons for participating in a randomised controlled study. *Arch Dis Child* 1998; **79**: 120–125.

7 Pace C, Emanuel EJ, Chuenyam T *et al*. The quality of informed consent in a clinical research study in Thailand. *IRB* 2005; **27**: 9–17.

8 Pace C, Talisuna A, Wendler D *et al*. Quality of parental consent in a Ugandan malaria study. *Am J Public Health* 2005; **95**: 1184–1189.

9 Moodley K, Pather M, Myer L. Informed consent and participant perceptions of influenza vaccine trials in South Africa. *J Med Ethics* 2005; **3**: 727–732.

10 Karim QA, Karim SSA, Coovadia HM *et al*. Informed consent for HIV testing in a South African hospital: is it truly informed and truly voluntary? *Am J Public Health* 1998; **488**: 637–640.

11 Leach A, Hilton S, Greenwood BM *et al*. An evaluation of the informed consent procedure used during a trial of a Haemophilus influenzae type B conjugate vaccine undertaken in The Gambia, West Africa. *Soc Sci Med* 1999; **48**: 139–148.

12 Lynoe N, Hyder Z, Chowdhury M *et al*. Obtaining informed consent in Bangladesh. *N Engl J Med* 2001; **344**: 460–461.

13 Joubert G, Steinberg H, van der Ryst E *et al*. Consent for participation in the Bloemfontein vitamin A trial: how informed and voluntary? *Am J Public Health* 2003; **93**: 582–584.

14 Marshall PA, Adebamowo CA, Adeyemo AA *et al*. Voluntary participation and informed consent to international genetic research. *Am J Public Health* 2006; **96**: 1989–1995.

15 Taub HA, Baker MT, Sturr JF. Informed consent for research. Effects of readability, patient age, and education. *J Am Geriatr Soc* 1986; **34**: 601–606.

16 Davis TC, Holcombe RF, Berkel HJ *et al*. Informed consent for clinical trials: a comparative study of standard versus simplified forms. *J Natl Cancer Inst* 1998; **90**: 668–674.

17 Coyne CA, Xu R, Raich P *et al*. Randomised, controlled trial of an easy-to-read informed consent statement for clinical trial participation: a study of the Eastern Cooperative Oncology Group. *J Clin Oncol* 2003; **21**: 836–842.

18 Dresden GM, Levitt MA. Modifying a standard industry clinical trial consent form improves patient information retention as part of the informed consent process. *Acad Emerg Med* 2001; **8**: 246–252.

19 Young DR, Hooker DT, Freeberg FE. Informed consent documents: increasing comprehension by reducing reading level. *IRB* 1990; **12**: 1–5.

20 NCI Template Consent Form, Available at http://ctep.cancer.gov/protocolDevelopment/informed_consent.htm (accessed 13 March 2014).

21 Babiker AG, Emery S, Fätkenheuer G *et al*. Considerations in the rationale, design and methods of the Strategic Timing of AntiRetroviral Treatment (START) study. *Clin Trials* 2013; **10** (Suppl 1): S5–S36.

22 U.S. National Archives and Records Administration. Code of federal regulations. 2005. Title 45. Public Welfare.

23 Sharma S, Babiker AG, Emery S *et al*. Demographic and HIV-specific characteristics of participants enrolled in the INSIGHT Strategic Timing of AntiRetroviral Treatment (START) trial. *HIV Med* 2015; **16** (Suppl 1): 30–36.

24 Flory J, Emanuel E. Interventions to improve research participants' understanding in informed consent for research: a systematic review. *JAMA* 2004; **292**: 1593–1601.

25 INSIGHT START Study Group. Why START? Reflections that led to the conduct of this large long-term strategic HIV trial. *HIV Med* 2015; **16** (Suppl 1): 1–9.

© 2015 British HIV Association
