# Supplementary material for: A randomized trial comparing concise and standard consent forms in the START trial
Source: PLoS One. 2017 Apr 26;12(4):e0172607. doi: 10.1371/journal.pone.0172607 (PMC5406127; doi:10.1371/journal.pone.0172607)
Supplement: S6 File — (DOCX) [file pone.0172607.s006.docx]

**Section 1. INSIGHT START Study Group**

We would like to thank the START participants without whom this work would not be possible.

In addition to writing group, the following committee members contributed to the conduct of the START trial:

Community Advisory Board: C. Rappoport (INSIGHT community liaison), P.D. Aagaard, S. Collins, G.M. Corbelli, N. Geffen, C. Kittitrakul, T. Maynard, M. Meulbroek, D. Munroe, M.S. Nsubuga, D. Peavey, S. Schwarze, M. Valdez.

Substudy Chairs: J.V. Baker, D. Duprez (arterial elasticity); A. Carr, J. Hoy (bone mineral density); M. Dolan, A. Telenti (genomics); C. Grady (informed consent); G. Matthews, J. Rockstroh (liver fibrosis progression); W.H. Belloso, J.M. Kagan (monitoring); E. Wright, B. Brew, R.W. Price, K. Robertson, L. Cysique (neurology); K.M. Kunisaki, J.E. Connett, D.E. Niewoehner (pulmonary).

Endpoint Review Committee: A. Lifson (chair), W.H. Belloso, R.T. Davey Jr., D. Duprez, J.M. Gatell, J. Hoy, C. Pedersen, R.W. Price, R. Prineas, J. Worley.

Central Drug Repository and Drug Distribution: K. Brekke, S. Meger, B. Baugh, J. Eckstrand, C. Gallagher, J. Myers, J. Rooney, J. Van Wyk.

Network Laboratory Group: J. Baxter, C. Carey, A. DuChene, E.B. Finley, M. George, J. Grarup, M. Hoover, R. Pedersen, C. Russell, B. Standridge.

Specimen Repositories: E. Flowers, M. Hoover, K. Smith (Advanced BioMedical Laboratories, LLC, Cinnaminson, NJ, United States); M. McGrath, S. Silver (AIDS and Cancer Specimen Resource, University of California, San Francisco, San Francisco, CA, United States).

Wake Forest ECG Reading Center, Winston-Salem, NC, United States: E.Z. Soliman, M. Barr, C. Campbell, S. Hensley, J. Hu, L. Keasler, Y. Li, T. Taylor, Z.M. Zhang.

Division of AIDS, National Institute of Allergy and Infectious Diseases, Bethesda, MD, United States: B. Alston-Smith, E. DeCarlo, K. Klingman, M. Proschan.

Data and Safety Monitoring Board: S. Bangdiwala (chair), R. Chaisson, A.R. Fleischman, C. Hill, J. Hilton, O.H.M. Leite, V.I. Mathan, B. Pick, C. Seas, P. Suwangool, G. Thimothe, F. Venter, I. Weller, P. Yeni.

Minnesota Coordinating Center, University of Minnesota, Minneapolis, MN, United States: J.D. Neaton, K. Brekke, G. Collins, E.T. Denning, A. DuChene, N.W. Engen, M. George, B. Grund, M. Harrison, K.H. Hullsiek, L.H. Klemme, E. Krum, G. Larson, S. Meger, R. Nelson, J. Neuhaus Nordwall, K. Quan, S.F. Quan, T. Schultz, S. Sharma, G. Thompson.

International Coordinating Centers**:** *Copenhagen HIV Programme, Rigshospitalet, University of Copenhagen,Denmark*: J.D. Lundgren, B. Aagaard, A.H.D. Borges, M. Eid, J. Grarup, P. Jansson, Z. Joensen, B. Nielsen, M. Pearson, R. Pedersen, A.N. Phillips; *The Kirby Institute, University of New South Wales, Sydney, Australia*: S. Emery, N. Berthon-Jones, C. Carey, L. Cassar, M. Clewett, D. Courtney-Rodgers, P. Findlay, S. Hough, S. Jacoby, J. Levitt, S.L. Pett, R. Robson, V. Shahamat, A. Shambrook; *Medical Research Council Clinical Trials Unit at UCL, London, United Kingdom:* A.G. Babiker, B. Angus, A. Arenas-Pinto, R. Bennett, N. Braimah, E. Dennis, N. Doyle, M. Gabriel, F. Hudson, B. Jackson, A. Palfreeman, N. Paton, C. Purvis, C. Russell; *Veterans Affairs Medical Center, Washington, DC, United States*: F. Gordin, D. Conwell, H. Elvis, E.B. Finley, V. Kan, L. Lynch, J. Royal, A. Sánchez, B. Standridge, D. Thomas, M. Turner, M.J. Vjecha.

The following investigators participated in the START study, listed by country (country lead, numbers of participants enrolled) and clinical site:

Argentina (M.H. Losso, n=216): *CAICI (Instituto Centralizado de Assistencia e Investigación Clínica Integral), Rosario Santa Fe*: S. Lupo, L. Marconi, D. Aguila; *FUNCEI, Buenos Aires*: G. Lopardo, E. Bissio, D. Fridman; *Fundación IDEAA, Buenos Aires*: H. Mingrone, E. Loiza, V. Mingrone; *Hospital General de Agudos JM Ramos Mejia, Buenos Aires*: M. Losso, J.M. Bruguera, P. Burgoa; *Hospital Interzonal General de Agudos Dr. Diego Paroissien, Buenos Aires*: E. Warley, S. Tavella; *Hospital Italiano de Buenos Aires, Buenos Aires*: W. Belloso, M. Sanchez; *Hospital Nacional Profesor Alejandro Posadas, Buenos Aires*: H. Laplumé, L. Daciuk; *Hospital Rawson, Cordoba:* D. David, A. Crinejo; *Argentinean SCC, Fundación IBIS, Buenos Aires*: G. Rodriguez-Loria, L. Doldan, A. Moricz, I. Otegui, I. Lanusse.

Australia (J. Hoy, n=109): *Burwood Road General Practice, Burwood, VIC*: N. Doong, S. Hewitt; *Centre Clinic, St Kilda, VIC*: B.K. Tee; *East Sydney Doctors, Darlinghurst, NSW:* D. Baker, E. Odgers; *Holdsworth House Medical Practice, Darlinghurst, NSW*: S. Agrawal, M. Bloch; *Melbourne Sexual Health Centre, Carlton, VIC*: T.R.H. Read, S.J. Kent; *Prahran Market Clinic, Prahran, VIC:* H. Lau, N. Roth; *Royal Adelaide Hospital, Adelaide, SA*: L. Daly, D. Shaw; *Royal Perth Hospital, Perth, WA*: M. French, J. Robinson; *Sexual Health & HIV Service - Clinic 2, Brisbane, QLD*: M. Kelly, D. Rowling; *St Vincent's Hospital, Fitzroy, VIC*: D.A. Cooper, A. J. Kelleher; *Taylor Square Private Clinic, Surry Hills, NSW*: C. Pell, S. Dinning; *The Alfred Hospital, Melbourne, VIC*: J. Hoy, J. Costa; *Westmead Hospital, Westmead, NSW*: D.E. Dwyer, P. King.

Austria (A. Rieger, n=7): *Otto-Wagner-Spital SMZ /Baumgartner Hoehe, Vienna*: N. Vetter; B. Schmied; *University Vienna General Hospital, Vienna*: A. Rieger, V.R. Touzeau.

Belgium (S. de Witt, n=102): *Centre Hospitalier Universitaire St. Pierre (C.H.U. St. Pierre), Brussels*: S. de Witt, N. Clumeck, K. Kabeya; *Institute of Tropical Medicine, Antwerp*: E. Cleve, E. Florence, L. van Petersen; *Universitair Ziekenhuis Gasthuisberg, Leuven*: H. Ceunen, E.H. van Wijngaerden; *Universitaire Ziekenhuizen Gent, Gent:* T. James, L. Vandekerckhove.

Brazil (L.C. Pereira Jr., M. Schechter, n=619): *Ambulatório de Imunodeficiências (LIM-56), Sao Paulo, SP*: J. Casseb, E. Constantinov, M.A. Monteiro; *Center for ID at UFES, Vitoria, ES*: L.N. Passos, T. Reuter; *Centro de Referência e Treinamento DST/AIDS, Sao Paulo, SP*: S.T. Leme, J.V.R. Madruga, R.S. Nogueira; *Hospital Escola Sao Francisco de Assis, Rio de Janeiro, RJ*: M. Barbosa Souza, C. Beppu Yoshida, M. Dias Costa; *Instituto de Infectologia Emilio Ribas, Sao Paulo, SP*: R. Castro, R.Cruz, S. Ito, T.N. Lobato Souza; *Instituto FIOCRUZ, Rio de Janeiro, RJ:* B. Grinsztejn, V.G. Veloso, S. Wagner Cardoso; *SEI Serviços Especializados em Infectología LTDA, Salvador, Bahia*: F. Bahia, C. Brites, J. Correia.

Chile (M.J. Wolff, n=76): *Fundación Arriarán, Santiago*: M. Wolff, R. Northland, C. Cortés.

Czech Republic (D. Sedlacek, n=13): *Faculty Hospital Na Bulovce, Prague:* D. Jilich; *University Hospital Plzen, Plzen*: D. Sedlacek.

Denmark (J. Gerstoft, n=33): *Hvidovre University Hospital, Hvidovre*: P. Collins, L. Mathiesen; *Odense University Hospital, Odense*: L. Hergens, C. Pedersen; *Rigshospitalet, Copenhagen*: J. Gerstoft, L.P. Jensen; *Århus Universitetshospital, Skejby, Århus*: I.R. Lofthiem, L. Østergaard.

Estonia (K. Zilmer, n=8): *West Tallinn Central Hospital Infectious Diseases, Tallinn*: K. Zilmer.

Finland (M. Ristola, n=23): *Helsinki University Central Hospital, Helsinki:* M. Ristola, O. Debnam.

France (B. Hoen, n=111): *CHU Côte de Nacre – Caen, Caen*: R. Verdon, S. Dargere; *CHU de Besançon -Hôpital Jean-Minjoz, Besancon*: B. Hoen, C. Chirouze; *Groupe Hospitalier Pitié-Salpêtrière, Paris*: C. Katlama, M-A. Valantin; *Hôpital Antoine Béclère, Clamart*: F. Boue, I. Kansau; *Hôpital de Bicêtre, Le Kremlin-Bicetre*: C. Goujard, C. Chakvetadze; *Hôpital Européen Georges Pompidou, Paris:* L. Weiss, M Karmochkine; *Hôpital Foch, Suresnes:* D. Zucman, C. Majerholc; *Hôpital Gustava Dron, Tourcoing*: O.Robineau, R. Biekre; *Hôpital Henri Mondor, Creteil*: Y. Levy, J.D. Lelievre; *Hôpital Hôtel Dieu, Paris*: J.P. Viard, J Ghosn; *Hôpital Saint-Antoine, Paris*: J. Pacanowski, B. Lefebvre; *Hôpital Saint-Louis, Paris*: J.-M. Molina, L. Niedbalski, M. Previlon; *French SCC, ANRS-Inserm SC10, Paris:* J.P. Aboulker, C. Capitant, B. Lebas, N. Leturque, L. Meyer, E. Netzer.

Germany (G. Fätkenheuer, n=312): *EPIMED, Berlin*: K. Arastéh, T. Meier; *Gemeinschaftspraxis Jessen-Jessen-Stein, Berlin:* C. Zedlack, H. Jessen*; ICH Study Center, Hamburg*: S. Heesch, C. Hoffmann; *Ifi - Studien und Projekte GmbH, Hamburg*: A. Plettenberg, A. Stoehr; *Johann Wolfgang Goethe - University Hospital, Frankfurt*: G. Sarrach, C. Stephan; *Klinik I für Innere Medizin der Universität zu Köln, Cologne*: G. Fätkenheuer, E. Thomas*; Klinikum der Universität München, Munich*: J.R. Bogner, I. Ott; *Klinikum Dortmund GmbH, Dortmund*: M. Hower, C. Bachmann; *Medizinische Hochschule Hannover, Hannover*: M. Stoll, R. Bieder; *Medizinische Universitätsklinik - Bonn, Bonn*: J. Rockstroh, B. Becker; *Universitätsklinikum Düsseldorf, Düsseldorf*: B. Jensen, C. Feind; *Universitätsklinikum Erlangen, Erlangen*: E. Harrer, T. Harrer; *Universitätsklinikum Essen, Essen*: S. Esser, H. Wiehler; *Universitätsklinikum Heidelberg, Heidelberg*: M. Hartmann, R. Röger; *Universitätsklinikum Regensburg, Regensburg*: B. Salzberger, E. Jäger; *Universitätsklinikum Würzburg, Würzburg*: H. Klinker, G. Mark; *Universitätsklinikum, Hamburg-Eppendorf*: J. van Lunzen, N. Zerche; *German SSC, Johann Wolfgang Goethe - University Hospital, Frankfurt*: V. Müller, K. Tillman.

Greece (G. Touloumi, n=101): *AHEPA University Hospital, Thessaloniki Central Macedonia*: S. Metallidis, O. Tsachouridou; *Attikon University General Hospital, Athens*: A. Papadopoulos, K. Protopapas; *Evangelismos General Hospital, Athens:* A. Skoutelis, V. Papastamopoulos; *Hippokration University General Hospital of Athens, Athens*: H. Sambatakou, I. Mariolis; *Korgialenio-Benakio Hellenic Red Cross, Athens*: M. K. Lazanas, M. Chini; *Syngros Hospital, Athens*: S. Kourkounti, V. Paparizos; *Greek SCC, National Kapodistrian University of Athens, Athens*: G. Touloumi*, V. Gioukari, O. Anagnostou.*

India (n=91): *Institute of Infectious Diseases, Pune Maharashtra:* A. Chitalikar, S. Pujari; *YRGCARE Medical Centre VHS, Chennai CRS*: F. Beulah, N. Kumarasamy, S. Poongulali.

Ireland (P. Mallon, n=7): *Mater Misericordiae University Hospital, Dublin*: P. Mallon, P. McGettrick.

Israel (E. Kedem, n=28): *Rambam Medical Center, Haifa*: E. Kedem, S. Pollack; *Tel Aviv Sourasky Medical Center, Tel Aviv*: D. Turner.

Italy (G. Tambussi, n=33): *Lazzaro Spallanzani IRCSS, Rome:* A. Antinori, R. Libertone; *Ospedale San Raffaele S.r.l., Milan*: G. Tambussi, S. Nozza, M.R. Parisi.

Luxembourg (T. Staub, n=5): *Centre Hospitalier de Luxembourg, Luxembourg*: T. Staub, C. Lieunard.

Malaysia (n=18): *University Malaya Medical Centre, Kuala Lumpur:* R.I.S.R. Azwa.

Mali (S. Dao, n=41): *SEREFO/ CESAC Mali, Bamako, Bamako*: B. Baya, M. Cissé, D. Goita.

Mexico (n=48): *INCMNSZ (Instituto Nacional de Ciencias Médicas y Nutrición), Tlalpan D.F.*: J. Sierra-Madero, M.E. Zghaib.

Morocco (K.M. El Filali, n=44): *University Hospital Centre Ibn Rochd, Casablanca*: K.M. El Filali, I. Erradey, H. Himmich.

Nigeria (n=50*): Institute of Human Virology-Nigeria (IHVN), Garki, Abuja FCT*: E. Ekong, N. Eriobu.

Norway (V. Ormaasen, n=15*): Oslo University Hospital, Ulleval, Oslo*: V. Ormaasen, L. Skeie.

Peru (A. La Rosa, n=215): *Hospital Nacional Edgardo Rebagliati Martins, Lima, Lima*: M. Espichan Gambirazzio, F. Mendo Urbina; *Hospital Nacional Guillermo Almenara Irigoyen, Lima, Lima:* R. Salazar Castro, J. Vega Bazalar; *IMPACTA Salud y Educación, Lima, Lima:* M.E. Guevara, R. Infante, J. Sanchez, M. Sanchez*; IMPACTA San Miguel, Lima, Lima*: R. Chinchay, J.R. Lama, M. Sanchez; *Via Libre, Lima, Lima*: E.C. Agurto, R. Ayarza, J.A. Hidalgo.

Poland (A.J. Horban, n=68): *EMC Instytut Medyczny SA, Wroclaw*: B. Knysz, A. Szymczak; *Uniwersytecki Szpital Kliniczny, Bialystok*: R. Flisiak, A. Grzeszczuk; *Wojewodzki Szpital Zakazny, Warsaw*: A.J. Horban, E. Bakowska, A. Ignatowska.

Portugal (L. Caldeira, n=67): *Hospital Curry Cabral, Lisbon*: F. Maltez, S. Lino; *Hospital de Egas Moniz, Lisbon*: K. Mansinho, T. Bapista; *Hospital de Santa Maria, Lisbon*: M. Doroana, A. Sequeira, L. Caldeira; *Hospital Joaquim Urbano, Oporto:* J. Mendez, R.S.E. Castro.

South Africa (R. Wood, n=518): *1 Military Hospital, Pretoria Gauteng*: S.A. Pitsi; *Desmond Tutu HIV Centre - Cape Town, Cape Town, Western Province*: R. Kaplan, N. Killa, C. Orrell, M. Rattley; *Durban International Clinical Research Site, Durban, KwaZulu Natal*: U.G. Lalloo, R. Mngqibisa, S. Pillay; *Durban International Clinical Research Site WWH, Durban, KwaZulu Natal*: J. Govender, M. John; *University of Witwatersrand, Johannesburg, Gauteng*: S. Badal-Faesen, N. Mwelase, M. Rassool.

Spain (J.R. Arribas, n=234): *Complejo Hospitalario Xeral Cies, Vigo Pontevedra:* A.O. Hermida, F. Warncke; *Hospital Clínic de Barcelona, Barcelona*: J.M. Gatell, A. Gonzalez; *Hospital Clínico San Carlos, Madrid*: V. Estrada, M. Rodrigo; *Hospital de la Santa Creu i Sant Pau, Barcelona*: P. Domingo, M. Gutierrez; *Hospital del Mar, Barcelona*: H.J. Knobel, A. Gonzalez; *Hospital La Paz, Madrid*: J.R. Arribas, M. Montes Ramirez; *Hospital La Princesa, Internal Medicine and Infectious Disease Service CRS, Madrid*: I. de los Santo Gil, J. Sanz Sanz; *Hospital Universitari Germans Trias i Pujol, Badalona*: B. Clotet, J.M. Llibre, P. Cobarsi; *Hospital Universitari Mutua Terrassa, Terrassa Barcelona*: D. Dalmau, C. Badia; *Hospital Universitario Doce de Octubre, Madrid*: R. Rubio, M.M. del Amo; *Hospital Universitario Príncipe de Asturias, Alcala de Henares Madrid*: J. Sanz Moreno; *Hospital Universitario y Politécnico La Fe, Valencia*: J. López Aldeguer, S. Cuellar; *Spanish SSC, Acoiba, Madrid*: P. López, B. Portas, P. Herrero.

Sweden (M. Gisslén, n=2): *Sahlgrenska University Hospital, Sweden*: M. Gisslén, L. Johansson; *Skåne University Hospital, Malmö*: C. Håkangård, K. Törqvist.

Switzerland (H. Furrer, n=31): *Bern University Hospital, Bern*: H. Furrer, A. Rauch; *Unite VIH/SIDA Genèva, Genèva*: A.L. Calmy, B. Hirschel (retd), T Lecompte; *University Hospital Basel, Basel:* M. Stoeckle; *University Hospital Zurich, Zürich*: N. Muller, M. Rizo-Oberholzer; *Swiss SCC, Bern University Hospital, Bern:* H. Furrer, C. Bruelisauer, A. Christen, M. Lacalamita.

Thailand (K. Ruxrungtham, n=248): *Bamrasnaradura Infections Diseases Institute, Nonthaburi*: W. Prasithsirikul, S. Thongyen; *Chiangrai Prachanukroh Hospital, Chiang Rai*: P. Kantipong, S. Khusuwan; *Chonburi Regional Hospital, Chonburi*: C. Bowonwatanuwong, U. Ampunpong; *Chulalongkorn University Hospital, Bangkok*: K. Ruxrungtham, A. Avihingsanon, W. Thiansanguankul; *Khon Kaen University, Srinagarind Hospital, Khon Kaen*: P. Chetchotisakd, P. Motsikapun, S. Anunnatsari; *Ramathibodi Hospital, Bangkok*: S. Kiertiburanakul, N. Sanmeema; *Research Institute for Health Sciences (RIHES), Chiang Mai*: K. Supparatpinyo, P. Sugandhavesa; *Sanpatong Hospital, Chiang Mai*: V. Klinbuayaem, Y. Siriwarothai; *Siriraj Hospital, Bangkok Noi*: W. Ratanasuwan, T Anekthananon; *Thai SCC, The HIV Netherlands Australia Thailand Research Collaboration (HIV-NAT), Bangkok*: W. Harnnapachewin, T. Jupimai, P. Rerksirikul.

Uganda (P. Mugyenyi, n=349): *Joint Clinical Research Center (JCRC), Kampala*: P. Mugyenyi, C. Kityo, H. Mugerwa; *MRC/UVRI Research Unit on AIDS, Entebbe*: P. Munderi, B. Kikaire, J. Lutaakome; *MRC/UVRI Research Unit on AIDS, Masaka – satellite site*: Z. Anywaine.

United Kingdom (M.A. Johnson, n=339): *Barts Health NHS Trust, London*: C. Orkin, J. Hand; *Belfast Health and Social Care Trust (RVH), Belfast Northern Ireland*: C. Emerson, S. McKernan; *Birmingham Heartlands Hospital, Birmingham West Midlands*: D. White, C. Stretton; *Brighton and Sussex University Hospitals NHS Trust, Brighton East Sussex*: M. Fisher, A. Clarke, A. Bexley; *Chelsea and Westminster Hospital, London*: B. Gazzard, C. Higgs, A. Jackson; *Coventry and Warwickshire NHS partnership Trust, Coventry West Midlands*: S. Das, A. Sahota; *Gloucestershire Royal Hospital, Gloucester*: A. de Burgh-Thomas, I. Karunaratne; *Guy's and St.Thomas' NHS Foundation Trust, London*: J. Fox, J.M. Tiraboschi; *Imperial College Healthcare NHS Trust, London*: A. Winston, B. Mora-Peris; *Leicester Royal Infirmary, Leicester Leicestershire*: M.J. Wiselka, L. Mashonganyika; *Lewisham and Greenwich NHS Trust, London*: S. Kegg, T. Moussaoui; *North Manchester General Hospital, Manchester*: E. Wilkins, Y. Clowes; *Queen Elizabeth Hospital Birmingham, Birmingham West Midlands*: J. Ross, J. Harding; *Royal Berkshire Hospital, Reading Berkshire*: F. Chen, S. Lynch; *Royal Bournemouth Hospital, Bournemouth Dorset*: E. Herieka, J. Ablorde; *Royal Free London NHS Foundation Trust, London*: M.A. Johnson, M. Tyrer, M. Youle; *Sheffield Teaching Hospital NHS Foundation Trust, Sheffield South Yorkshire*: D. Dockrell, C. Bowman; *Southmead Hospital, Bristol*: M. Gompels, L. Jennings; *St. George's Healthcare NHS Trust, London*: P. Hay, O. Okolo; *The James Cook University Hospital, Middlesbrough Cleveland*: D.R. Chadwick, P. Lambert; *University College London Medical School, London*: I. Williams, A. Ashraf.

United States (K. Henry, n=507): *Adult Clinical Research Center, Newark, NJ*: M. Paez-Quinde, S. Swaminathan; *Boston University Medical Center, Boston, MA*: I. Bica, M. Sullivan; *Bronx-Lebanon Hospital Center, Bronx, NY*: R.B. Cindrich, L.M. Vasco; *Community Research Initiative of New England, Boston, MA*: J. Green, H.B. Olivet; *Cooper University Hospital, Camden, NJ:* J. Baxter, Y. Smith; *Cornell CRS, New York, NY*: V. Hughes, T. Wilkin; *Denver Public Health, Denver, CO*: E.M. Gardner, J. Scott; *Duke University, Durham, NC*: J. Granholm, N. Thielman; *Florida Department of Health in Orange/Sunshine Care Center, Orlando, FL*: W.M. Carter, N.D. Desai; *George Washington University Medical Center, Washington, DC*: D.M. Parenti, G.L. Simon; *Georgetown University Medical Center, Washington, DC*: P. Kumar, M. Menna; *Hennepin County Medical Center, Minneapolis, MN*: J. Baker, R. Givot; *Henry Ford Hospital, Detroit, MI*: L.H. Makohon, N.P. Markowitz; *Hillsborough County Health Department, Tampa, FL*: M. Chow, C. Somboonwit; *Infectious Disease Associates of Northwest Florida, Pensacola, FL*: A.B. Brown, B.H. Wade; *Lurie Children's Hospital, Chicago, IL*: J. Jensen, A. Talsky; *Maternal, Child and Adolescent Center for ID/Virology USC, Alhambra, CA*: A. Kovacs, L. Spencer; *Mayo Clinic, Rochester, MN*: S. Rizza, Z. Temesgen; *Medical College of Wisconsin, Milwaukee, WI*: M. Frank, S. Parker; *Montefiore Medical Center, Bronx, NY*: C. Rosario, J. Shuter; *Mt Sinai Hospital, Chicago, IL*: K. Rohit, R. Yogev; *National Military Medical Center, Bethesda, MD*: I. Barahona, A. Ganesan; *Naval Medical Center Portsmouth NMCP, Portsmouth, VA*: S. Banks, T. Lalani; *Naval Medical Center San Diego NMCSD, San Diego, CA*: M.F. Bavaro, S. Echols; *NICE, Southfield, MI*: M. Farrough, R.D. MacArthur; *NIH, Bethesda, MD*: R.T. Davey Jr., R. McConnell; *Ohio State University, Columbus, OH*: H. Harber, S.L. Koletar; *Orlando Immunology Center, Orlando, FL*: E. DeJesus, A.F. Garcia; *Regional Center for Infectious Disease, Greensboro, NC*: K. Epperson, C.N. Van Dam; *San Antonio Military Health System, JBSA Fort Sam Houston, TX*: J.F. Okulicz, T.J. Sjoberg; *San Juan Hospital, San Juan, PR*: M. Acevedo, L. Angeli; *St. Jude Children's Research Hospital, Memphis, TN*: P.M. Flynn, N. Patel; *Temple University, Philadelphia, PA*: C. Geisler, E. Tedaldi; *Texas Children's Hospital- Baylor College of Medicine, Houston, TX:* C. McMullen-Jackson, W.T. Shearer; *The Research & Education Group, Portland, OR*: M.D. Murphy, S.M. Sweek; *Tulane University Health Sciences Center, New Orleans, LA*: D. Mushatt, C. Scott; *UCLA CARE 4 Families, Los Angeles, CA:* M. Carter, J. Deville; *UCSD Mother-Child-Adolescent HIV Program, San Diego, CA*: S.A. Spector, L. Stangl; *University of Florida, Department of Pediatrics, Jacksonville, FL*: M.H. Rathore, K. Thoma; *University of Florida, Jacksonville, FL*: M. Sands, N. Wilson; *University of Illinois at Chicago, Chicago, IL*: R.M. Novak, T. Pearson; *University of Miami, Miami, FL:* M.A. Kolber, T. Tanner; *University of North Carolina, Chapel Hill, NC*: M. Chicurel-Bayard, E. Hoffman; *University of North Texas Health Science Center, Fort Worth, TX*: I. Vecino, S.E. Weis; *University of Puerto Rico, San Juan, PR*: I. Boneta, J. Santana; *University of Texas Southwestern Medical Center, Dallas, TX:* M.K. Jain, M. Santos; *Veterans Affairs Greater LA Healthcare System, Los Angeles, CA:* M.B. Goetz, W.L. Rossen; *Virginia Commonwealth University, Richmond, VA*: D. Nixon, V. Watson; *Wake County Human Services, Raleigh, NC*: D. Currin, C. Kronk; *Wake Forest University Health Sciences, Winston-Salem, NC*: L. Mosley, A. Wilkin; *Washington DC Veterans Administration, Washington, DC*: A.M. Labriola, D.W. Thomas; *Yale University School of Medicine, New Haven, CT*: D. Chodkowski, G. Friedland.
